# A compendium of next-generation patient-derived models for diverse cancers

**DOI:** 10.1038/s41586-026-10806-y

**Published:** 2026-08-05

**Authors:** Dina ElHarouni, Mushriq Al-Jazrawe, Seongmin Choi, Merve Dede, Toshinori Hinoue, Sean A. Misek, Heeju Noh, Luca Zanella, Yuen-Yi Tseng, Hayley E. Francies, Dennis Plenker, Cindy W. Kyi, Julyann Perez-Mayoral, Megan J. Stine, Eva Tonsing-Carter, Rachana Agarwal, Jean Claude Zenklusen, James M. Clinton, Jennifer M. Shelton, Timothy R. Chu, William F. Hooper, Xavi Loinaz, Paula Keskula, Jordan Tagle, Peyton C. Kuhlers, Bahar Tercan, Sylvia F. Boj, Alessandro Vasciaveo, Lorenzo Tomassoni, James M. Crawford, Shawna Walsh, Claire Sinai, Sonam Bhatia, Priya Sridevi, Hardik Patel, Maria Antonietta Cerone, The HCMI Network, Dina ElHarouni, Mushriq Al-Jazrawe, Seongmin Choi, Merve Dede, Toshinori Hinoue, Sean A. Misek, Heeju Noh, Luca Zanella, Yuen-Yi Tseng, Hayley E. Francies, Dennis Plenker, Cindy W. Kyi, Julyann Perez-Mayoral, Megan J. Stine, Eva Tonsing-Carter, Rachana Agarwal, Jean Claude Zenklusen, James M. Clinton, Jennifer M. Shelton, Timothy R. Chu, William F. Hooper, Xavi Loinaz, Paula Keskula, Jordan Tagle, Peyton C. Kuhlers, Bahar Tercan, Sylvia F. Boj, Alessandro Vasciaveo, Lorenzo Tomassoni, James M. Crawford, Shawna Walsh, Claire Sinai, Sonam Bhatia, Priya Sridevi, Hardik Patel, Maria Antonietta Cerone, Mubarak Akadri, Andrew J. Aguirre, Rehan Akbani, Majd Al Assaad, Wael Al Zoughbi, Alyaa Al-Ibraheemi, Sahar Alkhairy, Nasser Altorki, Silvia Andreani, Joshua Araya, Gayatri Arun, Adel Atari, Stefanie Avril, Toby M. Baker, Metin Balaban, Michael Barnes, Caitlyn W. Barrett, Adam Bass, Alexandra E. Beck, Pascal Belleau, Christopher C. Benz, Bhavneet Bhinder, Shriram G. Bhosle, Julie Boerner, Jay Bowen, Lauren Brais, Bradley M. Broom, Catherine A. Bullen, Jonathan M. Buscaglia, Thomas Anthony Caiazza, Joshua D. Campbell, Evelyn Cantillo, Song Cao, Jared A. Capuano, Mauro A. A. Castro, Eloise Chapman-Davis, Kami Chiotti, Toni K. Choueiri, Kin-Hoe Chow, Wolu Chukwu, Alanna J. Church, Hans Clevers, Catherine Clinton, Isidro Cortes-Ciriano, Daniel B. Costa, Gregory M. Cote, Brian D. Crompton, Sabrina D’Agosto, Simona Dalin, Melissa Davis, Frederik De Smet, Rebecca Deasy, Mimoun Delmar, Paula I. Denoya, Astrid Deschênes, Li Ding, Elizabeth R. Duffy, Ruvimbo Dzvurumi, Kenneth Eng, Jose Espejo Valle-Inclan, Bishoy M. Faltas, Michelle Feenstra, Idhaliz Flores, Sharon B. Fox, Melissa K. Frey, Marina Frimer, Jack Geduldig, Veerle Geurts, Gad Getz, James G. R. Gilbert, Gary L. Goldberg, Sara Goodwin, Peter K. Gregersen, Evan Grossman, Akansha A. Gupta, Amber N. Habowski, William C. Hahn, Peter Hammerman, D. Neil Hayes, David I. Heiman, Elizabeth P. Henske, Carmen Herranz-Ors, Julian M. Hess, Kevin Holcomb, Andrew L. Hong, Christine Hudson, Victoria Hung, Lora Iliev, Katherine A. Janeway, Julia O. Japo, Grace Johnson, Jihang Ju, Troy J. Kane, Samuel J. Klempner, Melissa Kramer, Anneke Kramm, Alexander Krasnitz, Shwetha V. Kumar, Rita Teresa Lawlor, Gwo-Shu Mary Lee, Siyun Lee, Hongyu Li, Ellen Li, Madison Liistro, Jochen Lorch, Carolina Lucchesi, Claudio Luchini, Seth Malinowski, Jyothi Manohar, Laura A. Martello, Jennifer L. Marti, M. Laura Martin, R. Jay Mashl, W. Richard McCombie, Aaron K. McCormick, Brian W. McSteen, Christine N. Metz, Ana Molina, Juan Miguel Mosquera, Jenna E. Moyer, Anthony Murphy, Payal Naik, Indu Nair, David M. Nanus, Emon Nasajpour, Jones T. Nauseef, Lisa Newman, Kimmie Ng, Samuel Y. Ng, Allyson Ocean, Coyin Oh, Kentaro Ohara, Kadir Ozler, Kila Panchot, Nicole Pavao, Antonio Pea, Kristine Pelton, Anson Peng, Claudia K. Petritsch, Payal Pradhan, Sidharth V. Puram, Meifang Qi, Srivatsan Raghavan, Benjamin J. Raphael, David Requena, Phoebe L. Reuben, Esther Rheinbay, Arvind Rishi, Carmen Rios, A. Gordon Robertson, Peter Ronning, Ashley R. Ruehr, Suzanne Russo, Andrea Ruzzenente, Michael Ryan, Roberto Salvia, David Sandak, Shahab Sarmashghi, Ashish Saxena, Abeer Sayeed, Parul Shukla, Andrea Sboner, Aldo Scarpa, Douglas S. Scherr, Francesco Serafini, Hui Shen, Aniket Shetty, Manish Shah, Ewa Sicinska, Sabina Signoretti, Michael Sigouros, Amber R. Smith, Yizhe Song, Yingduo Song, Conrado T. Soria, Cora Sternberg, Joshua M. Stuart, Cheryl L. Thompson, Natalie Tsang, Aviad Tsherniak, Cristina Valente, Sara Valentini, Johan H. van Es, Barbara Van Hare, Shivakumar Vignesh, Jayne Vogelzang, Abigail Ward, Fiona Watkinson, Sophie Webster, John N. Weinstein, David M. Weinstock, Michael C. Wendl, Brian M. Wolpin, Christopher K. Wong, Maoxin Wu, Matthew A. Wyczalkowski, William P. Wysocki, Alexa Yeagley, Smitha Yerrum, Charles H. Yoon, Jenny Yuan, Brian Yueh, Zhenyu Zhang, Kyle Ellrott, Calvin J. Kuo, Olivier Elemento, Semir Beyaz, Vincenzo Corbo, David L. Spector, Rameen Beroukhim, Martin L. Ferguson, Andrew D. Cherniack, Peter W. Laird, Nicolas Robine, Andrew McPherson, Katherine A. Hoadley, Mathew J. Garnett, David A. Tuveson, Andrea Califano, Paul T. Spellman, Keith L. Ligon, Daniela S. Gerhard, Louis M. Staudt, Jesse S. Boehm, Kyle Ellrott, Calvin J. Kuo, Olivier Elemento, Semir Beyaz, Vincenzo Corbo, David L. Spector, Rameen Beroukhim, Martin L. Ferguson, Andrew D. Cherniack, Peter W. Laird, Nicolas Robine, Andrew McPherson, Katherine A. Hoadley, Mathew J. Garnett, David A. Tuveson, Andrea Califano, Paul T. Spellman, Keith L. Ligon, Daniela S. Gerhard, Louis M. Staudt, Jesse S. Boehm

**Affiliations:** 1 Broad Institute of MIT and Harvard, Cambridge, MA USA; 2 Department of Pathology, Dana-Farber Cancer Institute, Harvard Medical School, Boston, MA USA; 3 Koch Institute for Integrative Cancer Research, Massachusetts Institute of Technology, Cambridge, MA USA; 4 Computational Oncology, Department of Epidemiology and Biostatistics, Memorial Sloan Kettering Cancer Center, New York, NY USA; 5 The Halvorsen Center for Computational Oncology, Memorial Sloan Kettering Cancer Center, New York, NY USA; 6 Department of Bioinformatics and Computational Biology, University of Texas MD Anderson Cancer Center, Houston, TX USA; 7 Van Andel Institute, Department of Epigenetics, Grand Rapids, MI USA; 8 Departments of Cancer Biology and Medical Oncology, Dana-Farber Cancer Institute, Harvard Medical School Boston, Boston, MA USA; 9 Department of Systems Biology, Vagelos College of Physicians and Surgeons, Columbia University Irving Medical Center, New York, NY USA; 10 Institute for Systems Biology, Seattle, WA USA; 11 Wellcome Sanger Institute, Cambridge, UK; 12 Cold Spring Harbor Laboratory Cancer Center, Cold Spring Harbor, New York, NY USA; 13 Center for Cancer Genomics, NCI, Bethesda, MD USA; 14 Management Services, Calverton, MD USA; 15 Frederick National Laboratory for Cancer Research, Frederick, MD USA; 16 American Type Culture Collection, Manassas, VA USA; 17 New York Genome Center, New York, NY USA; 18 Krantz Family Center for Cancer Research, Boston, MA USA; 19 Oregon Health & Science University, Department of Biomedical Engineering, Portland, OR USA; 20 Department of Genetics, Lineberger Comprehensive Cancer Center, University of North Carolina at Chapel Hill, Chapel Hill, NC USA; 21 HUB Organoids, Merck, Utrecht, The Netherlands; 22 Center for Data Science and Artificial Intelligence, NCI-Designated Cancer Center, Sanford Burnham Prebys Medical Discovery Institute, La Jolla, CA USA; 23 DarwinHealth, New York, NY USA; 24 Northwell Health, New Hyde Park, NY USA; 25 Cancer Research Horizons, Therapeutic Innovation, Babraham Research Campus, Cambridge, UK; 26 Department of Medicine, Division of Hematology, Stanford University School of Medicine, Stanford, CA USA; 27 Englander Institute for Precision Medicine, Weill Cornell Medicine, New York, NY USA; 28 Department of Engineering for Innovation Medicine and ARC-Net Research Centre, University of Verona, Verona, Italy; 29 Biohub New York, New York, NY USA; 30 Herbert Irving Comprehensive Cancer Center, Columbia University, New York, NY USA; 31 Department of Biochemistry and Molecular Biophysics, Vagelos College of Physicians and Surgeons, Columbia University, New York, NY USA; 32 Department of Biomedical Informatics, Vagelos College of Physicians and Surgeons, Columbia University, New York, NY USA; 33 Department of Medicine, Vagelos College of Physicians and Surgeons, Columbia University, New York, NY USA; 34 Departments of Medicine and Human Genetics, University of California Los Angeles, Los Angeles, CA USA; 35 Department of Pathology, Brigham and Women’s Hospital, Harvard Medical School, Boston, MA USA; 36 Department of Pathology, Boston Children’s Hospital, Harvard Medical School, Boston, MA USA; 37 Lymphoid Malignancies Branch, Center for Cancer Research, NCI, Bethesda, MD USA; 38 SUNY Downstate Health Sciences University, Brooklyn, NY USA; 39 Dana-Farber Cancer Institute, Harvard Medical School, Boston, MA USA; 40 Boston Children’s Hospital, Harvard Medical School, Boston, MA USA; 41 University of Verona, Verona, Italy; 42 Case Western Reserve University, Cleveland, OH USA; 43 Department of Medicine, University of California Los Angeles, Los Angeles, CA USA; 44 Department of Computer Science, Princeton University, Princeton, NJ USA; 45 Milken Institute, Washington, DC USA; 46 Buck Institute for Research on Aging, Novato, CA USA; 47 Department of Oncology, Karmanos Cancer Institute, Wayne State University, Detroit, MI USA; 48 Abigail Wexner Research Institute, Nationwide Children’s Hospital, Columbus, OH USA; 49 Center for Translational Data Science, University of Chicago, Chicago, IL USA; 50 Stony Brook Medicine, Stony Brook, NY USA; 51 Boston University, Boston, MA USA; 52 Department of Medicine and McDonnell Genome Institute, Washington University in St Louis, St Louis, MO USA; 53 Bioinformatics and Systems Biology Laboratory, Federal University of Paraná, Curitiba, Brazil; 54 Hubrecht Institute, Royal Netherlands Academy of Arts and Sciences and UMC Utrecht, Utrecht, The Netherlands; 55 Oncode Institute, Utrecht, The Netherlands; 56 European Molecular Biology Laboratory, European Bioinformatics Institute, Cambridge, UK; 57 Beth Israel Deaconess Medical Center, Harvard Medical School, Boston, MA USA; 58 Massachusetts General Brigham Cancer Institute, Harvard Medical School, Boston, MA USA; 59 Institute of Translational Genomic Medicine, Morehouse School of Medicine, Atlanta, GA USA; 60 Ponce Health Science University, Ponce, PR USA; 61 Department of Pathology, Harvard Medical School, Boston, MA USA; 62 Departments of Medical Oncology and Cancer Biology, Dana-Farber Cancer Institute, Boston, MA USA; 63 UTHSC Center for Cancer Research, University of Tennessee Health Science Center, Memphis, TN USA; 64 Brigham and Women’s Hospital, Boston, MA USA; 65 Emory University and Children’s Healthcare of Atlanta, Atlanta, GA USA; 66 Rare Cancer Research Foundation, Durham, NC USA; 67 Departments of Neurology and Neurosciences, and Neurosurgery, Stanford Cancer Institute, Stanford, CA USA; 68 Department of Otolaryngology–Head and Neck Surgery, Washington University School of Medicine, St Louis, MO USA; 69 Department of Genetics, Washington University School of Medicine, St Louis, MO USA; 70 The Robert Ebert and Greg Stubblefield Head and Neck Tumor Center at Siteman, Washington University, St Louis, MO USA; 71 BC Cancer, Genome Sciences Centre, Vancouver, British Columbia Canada; 72 Department of Biomolecular Engineering, UC Santa Cruz Genomics Institute, Santa Cruz, CA USA

**Keywords:** Cancer models, Cancer genomics

## Abstract

The development of new therapeutics and the validation of pathogenetic cancer mechanisms require representative laboratory models^1,2^. However, existing collections represent only a fraction of the diversity observed in human cancer^2–4^. Recent technologies have enabled efficient in vitro model derivation (for example, tumour organoids)^5^. However, whether these maintain essential properties of patient tumours during long-term expansion has not been systematically investigated. Here we present results of a large-scale international programme—the Human Cancer Models Initiative—which involved the generation of a resource of 665 next-generation models from 2,780 donors with 25 cancer types and integrated tumour–model whole genome, exome, methylome and transcriptome analyses. The resource provides 522 models with comprehensive clinical data, 153 models of rare cancers and 71 models from participants with non-European ancestry. Analyses of 421 matched tumour–model pairs reveal high genetic (97.8%) and epigenetic (95%) concordance and define correlates of model discordance. Single-nucleus RNA sequencing of tumour–model pairs reveals subsets of models in which culture conditions significantly influence cell states. Finally, we characterize model preservation of extrachromosomal DNA and post-treatment mutational signatures to provide opportunities to study therapeutic resistance. This model repository is being made available to the community—including multimodal molecular profiling, clinical information and integrative software tools—thus providing a valuable resource for preclinical investigation of cancer pathogenesis and treatment response.

## Abstract (web summary)

The international collaboration of the Human Cancer Models Initiative presents a comprehensive resource of next-generation cancer models from 2,780 donors with 25 cancer types and integrated tumour–model genomic, transcriptomic and epigenomic analyses.

## Main

Major international projects have generated cancer atlases^3,6^ that have revealed many molecular underpinnings of human malignancies^7–9^. Provocative hypotheses about most types of cancer have emerged, with each requiring preclinical validation to support both basic science and drug discovery. Well-characterized cancer models that maintain the fidelity of diverse human tumour types and clinical states are required for such validation^10^.

Efforts to genomically characterize large cohorts of cancer models have produced comprehensively annotated compendia of over 1,000 cell lines^1,2,11,12^, which have enabled large-scale mapping of cancer dependencies^9,13–16^. However, existing cell lines typically exhibit limited phenotypic complexity, uncertain fidelity to the originating tumour specimen and inadequate clinical information^17^.

Advances in the technology of cell culture, including biologically informed selection of defined growth factors and new plating methods, have facilitated the development of new patient-derived models^5,18–25^. However, reports suggest that only a minority of such models could be indefinitely propagated and distributed.

Here we present the generation and comprehensive genomic, transcriptomic and epigenomic characterization of 665 fully accessible patient-derived organoid, neurosphere and cell line models, which were created as part of the international Human Cancer Models Initiative (HCMI). Companion papers^26,27^ demonstrate the utility of HCMI models for the systematic evaluation of gene essentiality and drug sensitivity.

## Generation of 665 cancer models

**Fig. 1:**
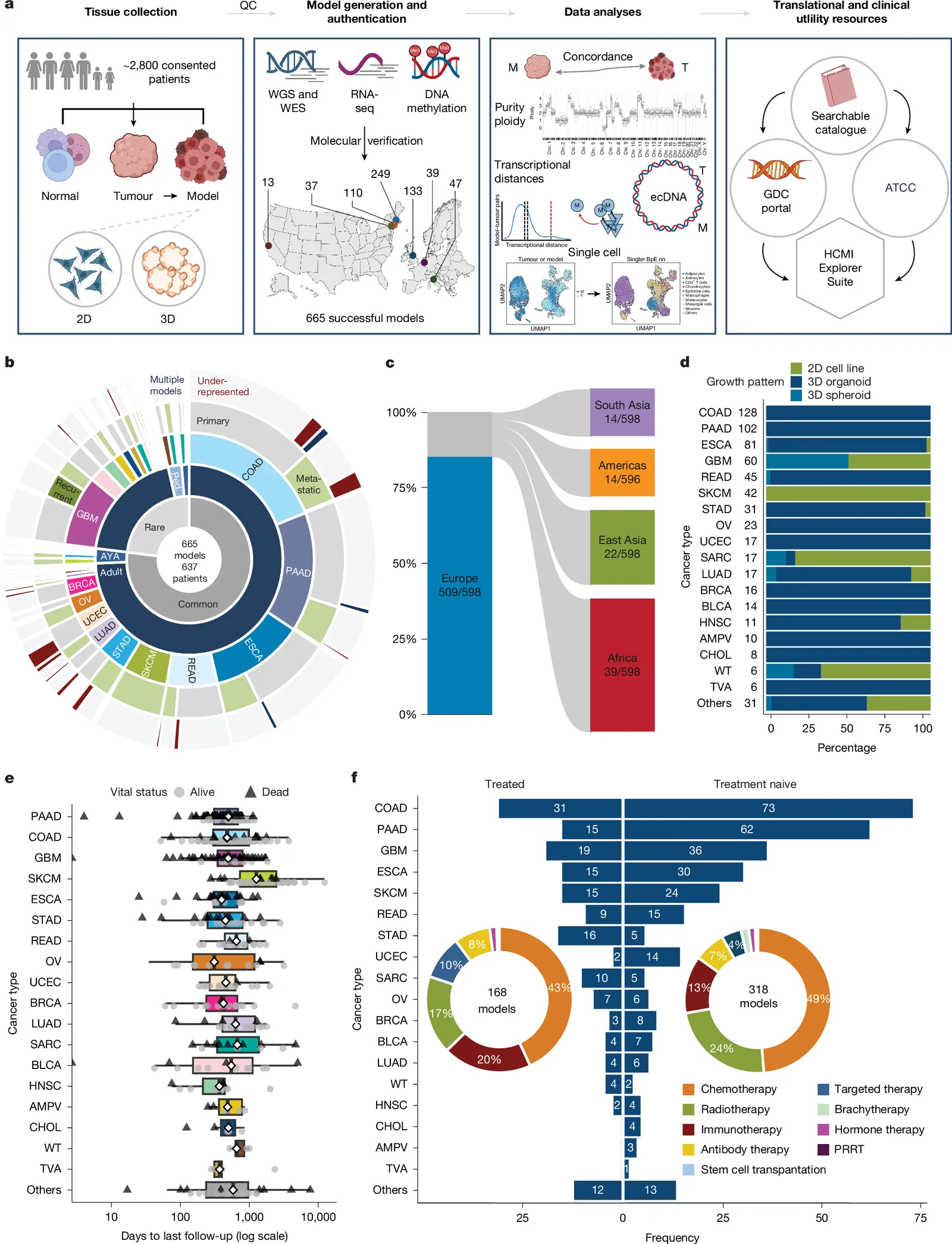
Overview of the HCMI resource and clinical demographics. **a**, Schematic of the HCMI workflow. **b**, Distribution of the 665 HCMI models derived from 637 patients by cancer type and patient demographics. The inner layer classifies HCMI donors by common versus rare cancers; the second layer groups patients by age category (paediatric, adolescent and young adult (AYA) and adult); the third layer groups tumours according to primary HCMI diagnosis, with cancer types coded as in Supplementary Table 1; the fourth layer maps the tumour status (primary, recurrent, metastatic or non-malignant); whereas the outer layer highlights underrepresented patients in red, and patients with multiple models in blue. **c**, Predicted continental ancestry of *n*= 664 HCMI models based on genomic data. Each model is represented by its majority ancestry component (see Supplementary Fig. 4 for full ancestry proportion assignments). **d**, Growth patterns of *n*= 665 HCMI models, showing proportions of 2D adherent, 3D organoid and 3D spheroid cultures across cancer types. **e**, Patient vital status and duration of follow-up across cancer types (log scale format). Box plots show the median (centre line), 25th–75th percentiles and 1.5× interquartile (IQR) whiskers. Individual data points are overlaid; circles indicate alive patients and triangles indicate deceased patients, *n* = 468 patients with available vital status follow-up data. **f**, Number of models for each cancer type, distributed by treatment status (left, treated, *n* = 168; right, treatment naive, *n* = 318 models), PRRT, peptide receptor radionuclide therapy. Pie charts show the percentage distribution of treatment types for both treatment status cohorts. Schematic in **a** created in BioRender; Bhatia, S. https://biorender.com/wq31pxj, https://biorender.com/i0a9q60 (2026).

Between 2016 and 2021, 2,780 patients from the United States, United Kingdom, Italy and the Netherlands consented to participate in the HCMI study (Fig. 1a and Supplementary Table 1). Active attempts were made to include diverse tumour types and patients from under-represented populations (Supplementary Methods, ‘Human subjects protocol’). Tissue samples were transferred to Cancer Model Development Centers at the Broad Institute, Cold Spring Harbor Laboratory, Weill Cornell Medicine, Stanford University, the Hubrecht Institute and the University of Verona ARC-Net Research Centre (Supplementary Table 1) for model generation, with additional models derived and contributed by the Wellcome Sanger Institute.

Model derivation techniques varied by tumour type, sample type and model-derivation centre (Supplementary Methods, ‘CMDC model development’, and Supplementary Table 1). After several sequential passages, cultures were subjected to genomic-based quality control, authentication and subsequently banked for distribution (Supplementary Note 2 and Supplementary Figs. 1 and 2).

**Extended Data Fig. 1:**
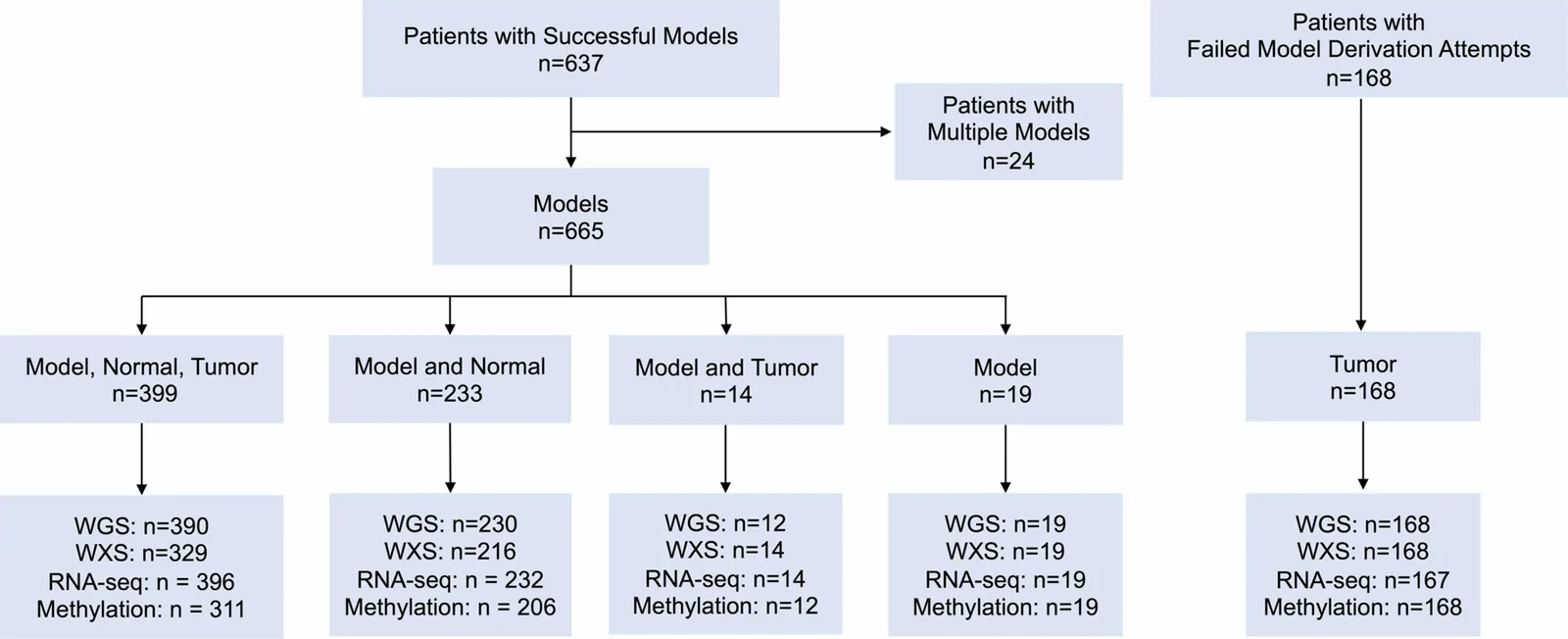
Consort diagram of HCMI model frequencies and molecular characterization. The diagram summarizes sequencing data generated for the HCMI cohort (n = 665 models). Counts are shown for models with available tumor, normal, and model samples, including complete trios (n = 399), pairs of model and normal (n = 233), pairs of model and tumor (n = 14), and models without matched tumor or normal samples (n = 19). For each group, the number of samples profiled by WGS, WXS, RNA-seq, and DNA methylation is indicated. In addition, 168 failed derivation attempts are included, with sequencing data available for tumor tissues (WGS, WXS, RNA-seq, and methylation).

This process generated 665 models from 637 patients representing 25 distinct human malignancies (Fig. 1b, Extended Data Tables 1 and 2 and Supplementary Note 2). Success rates varied by tumour type, clinical stage and tissue type, and were affected by sample quality. Among the successful models, most were derived from adult donors (*n* = 622; 93.5%), but paediatric and adolescent donors were also represented (*n* = 43; 6.5%). Moreover, 23% of the successful models (*n* = 153) were of rare cancer types (https://www.cancer.gov/pediatric-adult-rare-tumor/rare-tumors/about-rare-cancers). Models were established from primary tumours (*n* = 394; 59.2%) and metastatic lesions (*n* = 250; 37.6%) (Fig. 1b). Overall, 78% of models (*n* = 519) are three-dimensional (3D) organoid cultures, 6% are 3D spheroid cultures (*n* = 37) and 16% are two-dimensional (2D) adherent patient-derived cell lines (*n* = 109) (Fig. 1d, Supplementary Table 1). Patient-matched peripheral blood or normal tissue samples and parental tumour tissue samples were obtained for 95% and 62% of models, respectively (Fig. 1a and Extended Data Fig. 1). A total of 399 models had complete data trios (tumour, mode, and blood or normal samples) (Supplementary Table 1). Validated models were banked and made available for distribution through the American Type Culture Collection (ATCC).

We also analysed tumours from 168 failed derivation attempts (Extended Data Fig. 1). Specifically, we compared molecular features of successful and failed derivations across the three largest tumour cohorts (colon adenocarcinoma (COAD), oesophageal adenocarcinoma (ESCA) and pancreatic adenocarcinoma (PAAD)). Although tumour purity did not emerge as a significant covariate (Supplementary Fig. 3ab), failed tumour samples presented increased representation of states associated with DNA repair and the cell cycle, whereas successful tumour samples displayed greater representation of differentiation, stemness and epithelial–mesenchymal transition (EMT)-related programs (Supplementary Fig. 3c–f).

To improve the representation of under-represented populations, HCMI enabled enrolment through the network of Tissue Source Sites from the National Cancer Institute (Supplementary Methods, ‘Establishment of CMDCs’) and a direct-to-patient consent platform (https://pattern.org). We estimated genetic ancestry across five continental groups (Fig. 1c) (Methods, ‘Genetic ancestry estimation’). Across the cohort, the predominant ancestry groups were European (85%), African (6.5%), East Asian (3.7%), admixed American (2.5%) and South Asian (2.3%). Overall, 71 models are from primarily non-European ancestry donors. Some participants showed complex ancestry admixtures (Supplementary Fig. 4).

Previous modelling efforts lack systematic clinical annotations for each model. Here extensive clinical data, including treatment histories, were collected for 78% (*n* = 522) of the models (Supplementary Table 1 and Supplementary Fig. 2). Data included patient overall survival (74% of models, with overall survival range of 0–34 years), treatments received before and after tissue procurement and post-therapy surival (median follow-up of 1.5 years) (Fig. 1e). A total of 318 models were generated from treatment-naive tissue samples, and 168 were generated from patients who had received previous therapy (Fig. 1f and Supplementary Table 1).

## Evaluation of genomic fidelity

**Fig. 2:**
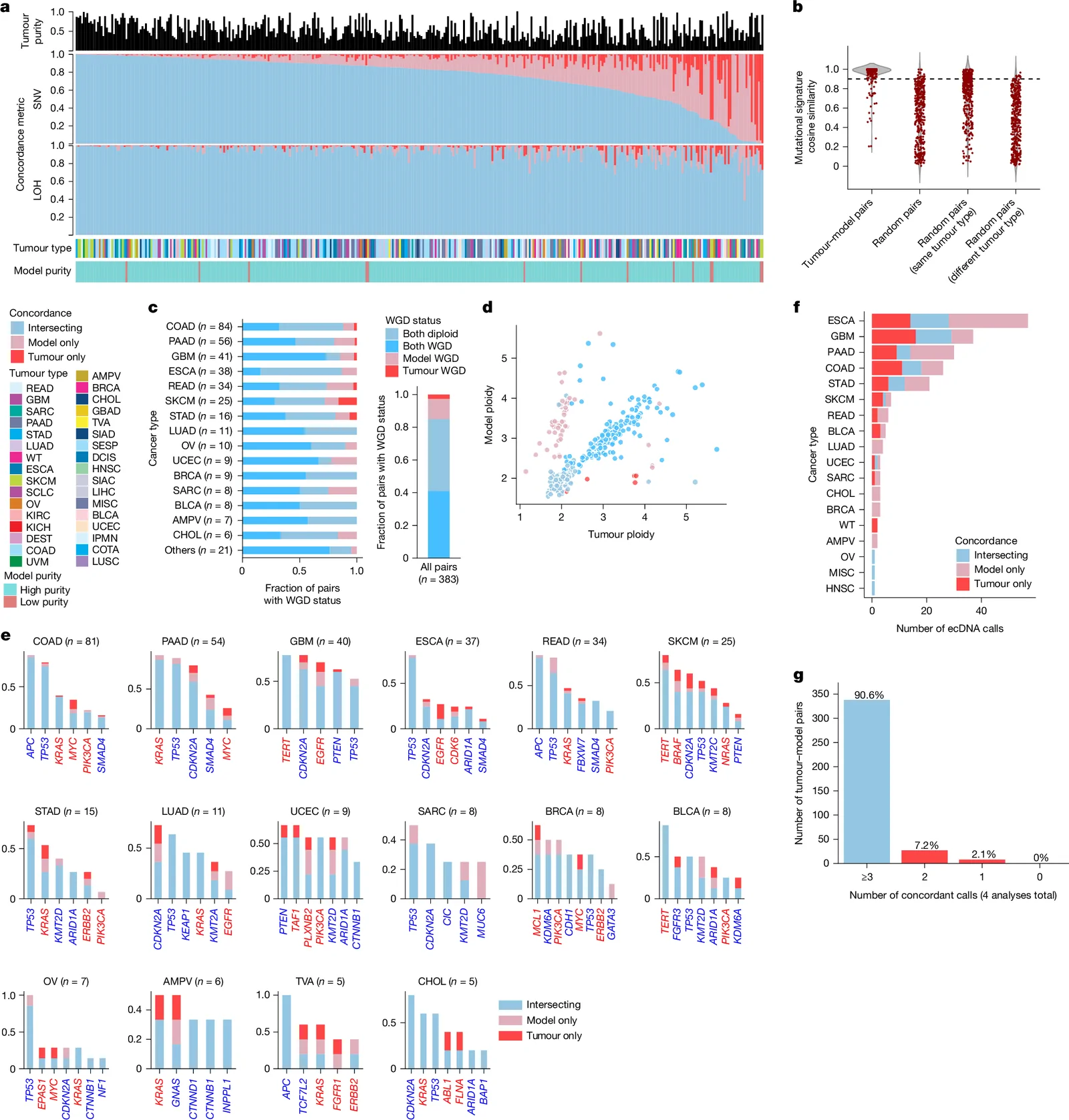
Tumour genomic alterations are recapitulated in cancer models. **a**, Concordance of SNVs and LOH across 388 tumour–model pairs. Top, tumour purity estimated from consensus copy number algorithms (Methods, ‘Consensus purity and ploidy’). Middle, fraction of variants shared between tumour and model (light blue), model-specific (light red) or tumour-specific (dark red). Bottom, analogous fractions for LOH events. **b**, Trinucleotide mutation signatures were inferred by non-negative matrix factorization (NMF). Similarity between tumour–model pairs was quantified by cosine similarity and compared with a background distribution from randomly paired samples. Pairs with cosine similarity > 0.9 were considered concordant. **c**, Whole-genome doubling (WGD) status across tumour–model pairs. Pairs were classified as both diploid (light blue), both WGD (dark blue), model-only WGD (light red) or tumour-only WGD (dark red). **d**, Comparison of ploidy estimates for tumour–model pairs. Each point represents one pair and is coloured as in **c**. **e**, Conservation of driver alterations across tumour–model pairs. Genes were selected on the basis of mutation frequency (SNVs or copy number alterations (CNAs)) in each tumour type. *TERT* alterations refer to canonical promoter mutations. Events were classified as shared (light blue), model-specific (light red) or tumour-specific (dark red). Genes are annotated as gain-of-function (red text) or loss-of-function (blue text). **f**, ecDNA events in tumour–model pairs. Events were categorized as shared (light blue), model-specific (light red) or tumour-specific (dark red). **g**, Fraction of tumour–model pairs concordant across up to four metrics: LOH, SBS signature similarity, WGD status and SNVs or indels.

**Extended Data Fig. 2:**
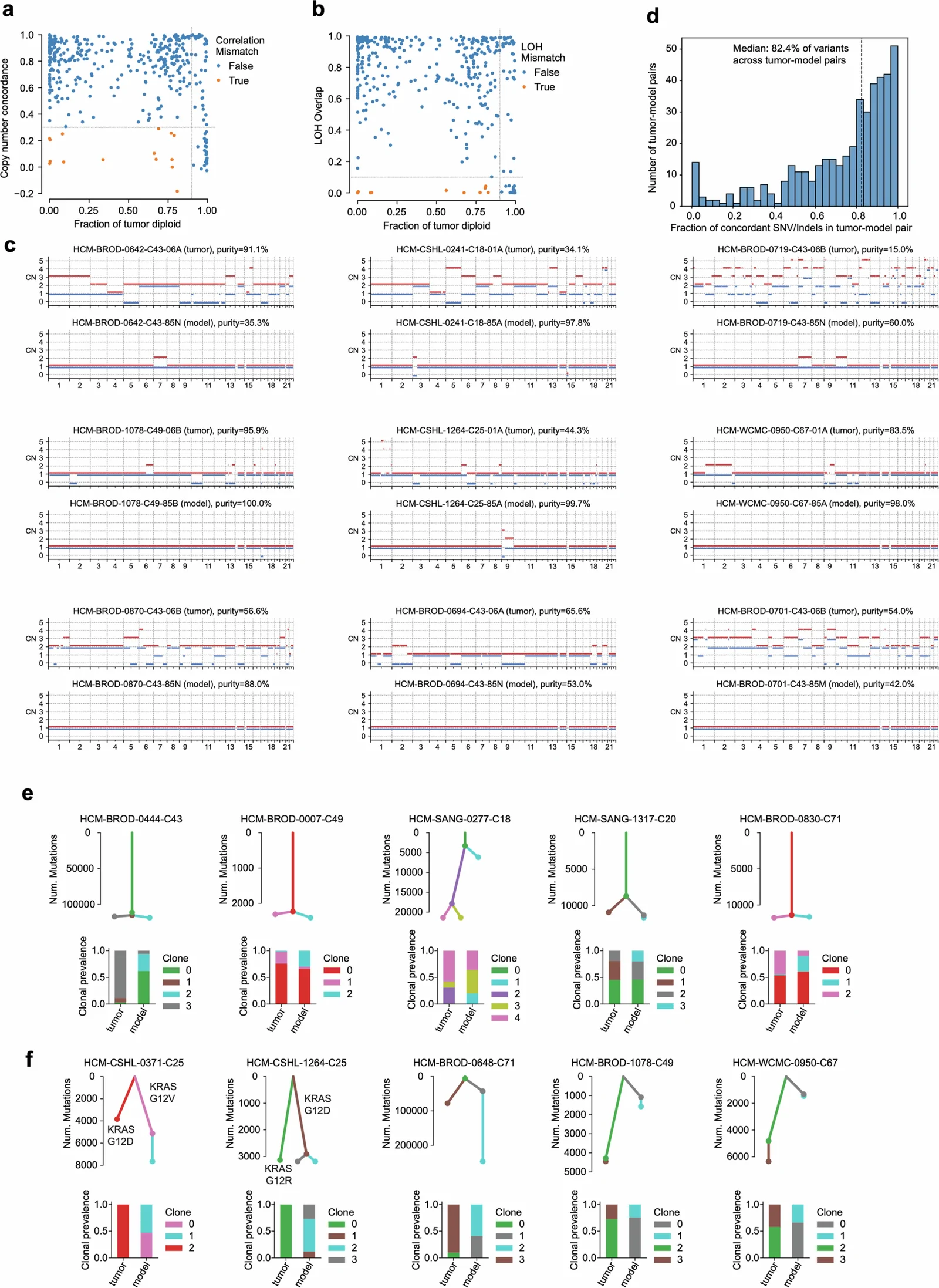
Clonal selection and copy number concordance in tumor/model pairs. (**a**) Pearson correlation between tumor and model total copy number (y-axis) by fraction of the tumor genome that is diploid (x-axis), with models that mismatched their respective tumors highlighted. (**b**) LOH concordance between tumor and model (y-axis) by fraction of the tumor genome that is diploid (x-axis), with models that mismatched their respective tumors highlighted. LOH concordance is calculated as the amount of LOH in both tumor and model as a fraction of LOH in either tumor or model. (**c**) Copy number for nine tumor/model pairs showing minor copy number in blue and major copy number in red. (**d**) Distribution of SNV/indel concordance values across tumor/model pairs. (**e**) Five models identified as highly discordant (**f**) Upper: predicted clonal phylogeny with branch lengths as the number of somatic mutations. Lower: prevalence of each clone in the tumor and sample. Clone-specific KRAS mutations are annotated for two divergent models in (**f**).

For the 421 models with matched parental tumours, we first evaluated DNA-level concordance. We compared ploidy and consensus copy number calls from tumour–model pairs using whole-genome sequencing (WGS) (Methods, ‘Genome sequencing: copy number’). For the 420 models for which purity values could be ascertained, 406 models had an estimated tumour purity of >80%, and very few (*n* = 9) models showed patterns of aneuploidy distinct from parental tumours. This result suggests that subclonal outgrowth is uncommon (Extended Data Fig. 2a–c). Moreover, 361 among 388 of the models with paired tumours exhibited a DNA loss of heterozygosity (LOH) concordance that exceeded 80% (average of 94%) (Fig. 2a).

Next, we evaluated concordance of single nucleotide variants (SNVs) and insertions and deletions (indels). A median of 82.4% of SNVs and indels were shared between tumour and normal pairs (Fig. 2a and Extended Data Fig. 2d). Several low-concordance cases were associated with low tumour purity or formalin-fixed paraffin-embedding (FFPE) of tumours producing sequencing artefacts (Fig. 2a and Methods (‘Biospecimen processing and quality control’)). Five tumour–model pairs stood out as markedly discordant (Extended Data Fig. 2e,f). These included two unique PAAD tumour–model pairs in which distinct *KRAS*^*G12*^ mutations were observed between the tumour and the model. This result probably reflects the multifocal and polyclonal nature of PAAD (Supplementary Note 3).

**Extended Data Fig. 3:**
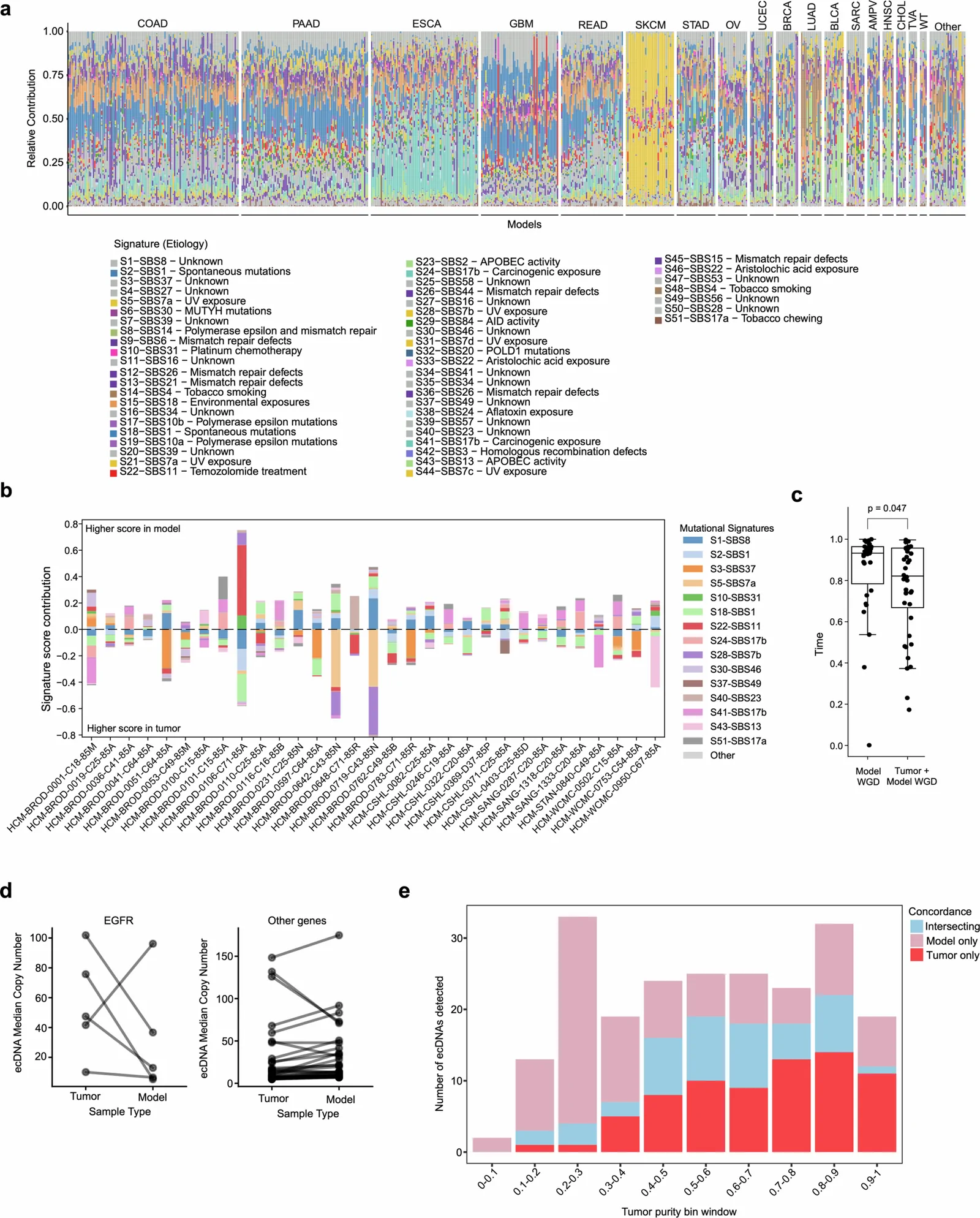
Levels of concordance of mutational signatures and ecDNA between tumor/model pairs. (**a**) Relative contribution of each SNV signature (y-axis) in each model (y-axis). (**b**) Mutational signature contribution (y-axis) in 31 discordant tumor/model pairs (x-axis). Positive signature contributions indicate that the signature is enriched in the model; negative signature contributions indicate the signature is enriched in the tumor. (**c**) Comparison of the estimated relative timing of WGD events between models exhibiting concordant WGD in both tumor and model (n=40, min=0.17, max=1.0, median=0.82) versus those with WGD detected only in the model (n=30, min=0.001, max=1.0, median=0.93). WGD events exclusive to models occur at significantly later relative mutational timepoints (*p* = 0.047, one-sided Wilcoxon rank-sum test; boxes and whiskers represent medians, and 25-75th percentiles, +/- 1.5 IQR). (**d**) Comparison of ecDNA levels (y-axis) for tumor model pairs for: EGFR (left, n = 5), and other ecDNAs (right, n = 30). (**e**) Number of ecDNAs detected in each tumor/model pair (y-axis) stratified by tumor purity (x-axis). Bars are colored by the fraction of ecDNAs that are unique to the tumor (dark red), unique to the model (light red), or are detected in both the tumor and model (blue).

We next assessed SNV mutational signatures by computing trinucleotide signature scores and cosine similarity between each tumour–model pair (Methods, ‘Analysis methods: Mutation signature’). We observed well-known signatures for specific tumour types (Extended Data Fig. 3a and Supplementary Table 2), which indicated that models retained relevant exposure signatures. The consistency was significantly higher than expected by chance (Fig. 2b). In the few pairs (*n* = 35; 8% of the total) with discordant signature profiles (cosine similarity < 0.9), no mutational signatures were consistently enriched in the models, which suggests that discordance was unlikely to arise from ex vivo culture (Extended Data Fig. 3b). These observations suggest that HCMI models faithfully recapitulate mutational signatures.

We next assessed whole-genome doubling (WGD) between tumour and model pairs. Most models recapitulated tumour WGD status (88%) (Fig. 2c), and the tumour–model ploidy was significantly correlated (Pearson’s correlation *r *= 0.53, *P* = 1.84 × 10^−29^; Fig. 2d). In 10% of the pairs, WGD was detected in the model but not the matched tumour, whereas WGD was seldom detected in the tumour but not the model (2% of cases) (Fig. 2c). SNV-based analyses indicated that WGD events detected only in models occurred later in the mutational timeline (*P* = 0.047, one-sided Mann–Whitney *U*-test). This finding is consistent with culture-associated clonal selection or spatial sampling variation. (Extended Data Fig. 3c), and provides support that tumour genomic structure is preserved.

Finally, we sought to confirm that well-known driver events were conserved. Across all tumour-specific driver events analysed, 81% of tumour events were retained in the models (Fig. 2e). Concordance was highest for canonical alterations in *BRAF* (skin cutaneous melanoma (SKCM)), *KRAS* (PAAD), *APC* (COAD and rectum adenocarcinoma (READ)) and *TP53* (ESCA). Consistent with the literature for glioblastoma (GBM)^28,29^, 80% of cases with extrachromosomal *EGFR* amplification showed higher *EGFR* copy number in the tumour than in the matched models (Extended Data Fig. 3d). On average, we detected a relatively lower level of concordance in extrachromosomal DNAs (ecDNAs) between tumour and model pairs, with 93 out of 212 (43.9%) of ecDNAs being model-specific and 69 out of 212 (32.5%) being tumour-specific (Fig. 2f). We attribute most of this discordance to low sequencing coverage (Extended Data Fig. 3e). However, differences in in vivo and ex vivo growth conditions may select for or against ecDNA amplification, as previously observed^30^.

In aggregate, 22 (5%) models were deprioritized for further interrogation owing to low purity. These models were excluded from subsequent analyses in this paper, which led to a collection of 643 prioritized HCMI models. In aggregate, our analyses confirmed that despite being passaged in culture for at least 1 year (Supplementary Methods, ‘Biospecimen collection and establishment of in vitro cultures’), most HCMI models (97.8%) retained at least two and often all four of the DNA-based features that we evaluated herein (driver mutations, mutational signatures, WGD and ploidy) (Fig. 2g).

## Epigenetic and transcriptomic fidelity

**Fig. 3:**
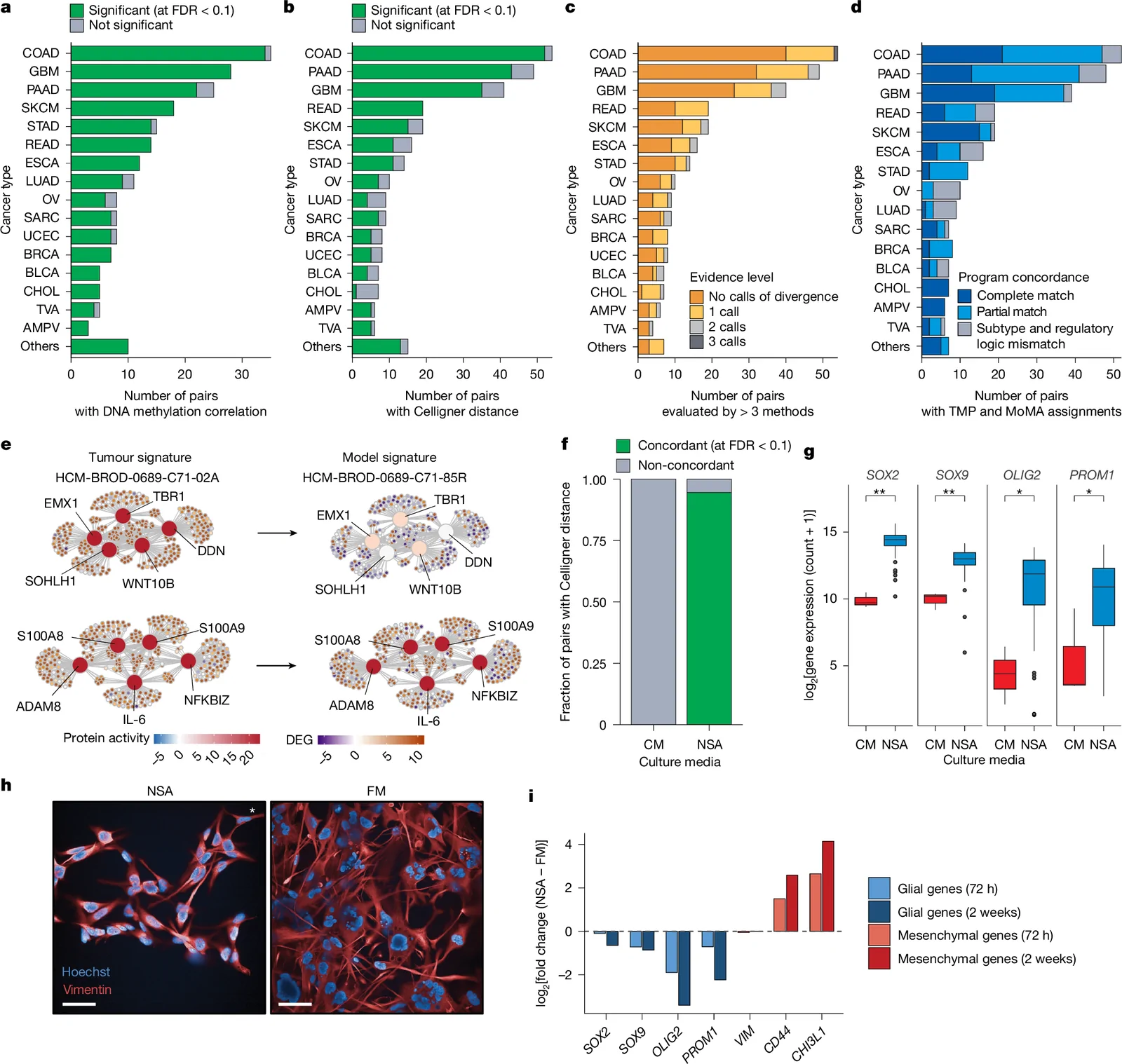
Transcriptional and epigenetic concordance between models and matched tumours. **a**, Similarity across 201 model–tumour pairs by DNA methylation distance, stratified by disease; ‘Others’ indicate <5 model cohorts. Bars indicate pair count; concordant pairs (FDR < 0.1; Methods, ‘Tumour–model hypermethylation similarity scores’) are in green. **b**, As in **a**, using Celligner distance (*n* = 297 pairs). **c**, Multimetric transcription divergence estimate agreement across transcriptional similarity methods (*n* = 286 models evaluated by ≥4 methods). Models not called divergent by any method are in orange (*n* = 178), one-method divergent in gold (*n* = 85) and those with divergence evidence from two (*n* = 22) or three (*n* = 1) in light or dark grey (Extended Data Fig. 4b shows models analysed by all). **d**, TMP subtype and MOMA regulatory logic concordance (*n* = 272 models). Matches by one analysis (*n* = 228) are in blue, and both in dark blue (*n* = 109). **e**, Top master regulators and target expression for the HCM-BROD-0689-C71 sample, with master regulator activity in red or blue (activation or inactivation, respectively), target expression in orange or purple (upregulated or downregulated, respectively) (mismatch example provided in Extended Data Fig. 4d). 02A denotes an aliquot of tissue from the primary tumour sample and 85R denotes an aliquot of cells from the corresponding derived model.** f**, Celligner similarity in GBM pairs by media: conditioned media (CM, *n* = 3 pairs) and NeuroCult NSA (*n* = 37 pairs). Two-sided Fisher’s exact *P* = 0.001. **g**, Expression of candidate markers by culture media (CM *n* = 3; NSA *n* = 56 models). Box plots show median (centre line), 25–75th percentiles and 1.5× IQR whiskers. Points are outliers. *P < 0.05, **P < 0.01 by a two-sided Wilcoxon rank-sum test (Extended Data Fig. 4e shows corresponding tumours). **h**, Immunofluorescence HCM-BROD-0416-C71 (GBM) under native media (NSA, asterisk) and after reconditioning in formulated-conditioned medium (FM) (*n* = 3 wells per condition). Scale bar, 50 µm. **i**, Marker expression log_2_ fold changes at 72 h and 2 weeks. Source data for **a**–**d** and **g** are provided in Supplementary Table 3.

**Extended Data Fig. 4:**
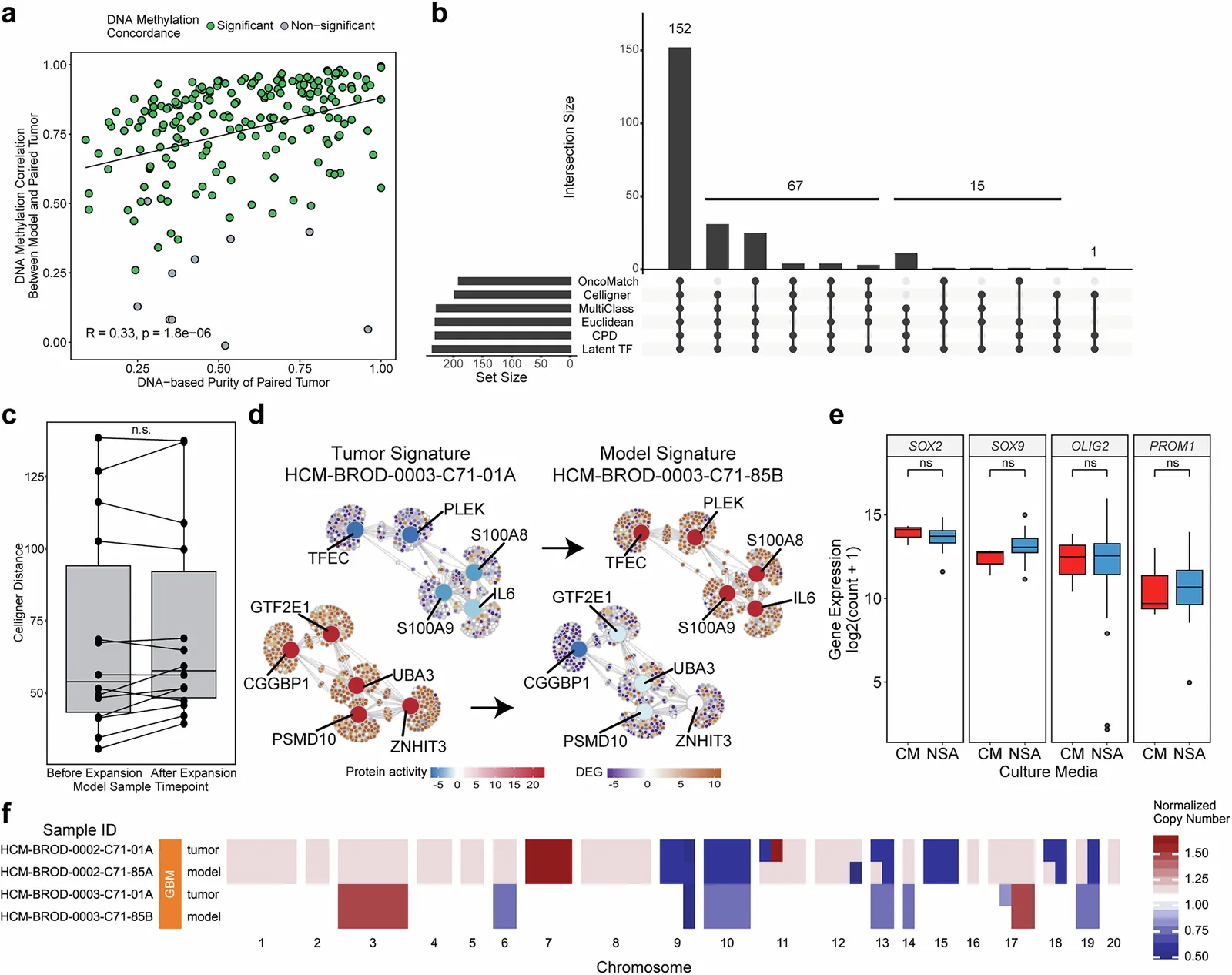
Transcriptional and epigenetic concordance between models and their matched tumors. (**a**) Correlation between epigenetic estimate of drift between model-tumor pairs and the starting tumor’s tissue purity. Each point represents a model-tumor pair (n = 201). The line shows the linear regression fit. The correlation was assessed using a two-sided Pearson correlation test (r = 0.33, P = 1.8 x 10^−6^). No multiple comparison correction was applied. DNA methylation fidelity (y-axis) is the similarity score (i.e. the Pearson correlation) between each model and its matched tumor. Tumor purity (x-axis) is the DNA-based consensus tumor purity estimate (see Methods). Gray dots indicate a similarity score probability that was no different from random chance (at FDR < 0.1, see Methods). (**b**) Intersection of transcriptional concordance methods. An UpSet plot of model-tumor pairs evaluated by all six independent transcriptional proximity methods (n = 235): Celligner, OncoMatch, MultiClass Pair Classification Distance (MultiClass), Canonical Parallel Direction (CPD), Euclidean Distance (Euclidean), and Latent Transcription Factor Distance (Latent TF). Set size indicates the number of model-tumor pairs that were considered concordant by each method. (**c**) Celligner-based transcriptional distance of tumors to their models, before and after *in vitro* expansion. Each point represents the Celligner distance for one model relative to its matched tumor at a given timepoint. Linked points indicate the same model evaluated before and after expansion to assess transcriptional drift over time (n = 14 biologically independent models; 14 paired comparisons). The y-axis is the Celligner-based distance between each model and its parent tumor (boxes and whiskers represent medians, and 25-75th percentiles, +/- 1.5 IQR, two-sided paired t-test P = 0.13, ns = not statistically significant). (**d**) MR activity of a divergent model-tumor pair. Visualization of top 10 candidate MR proteins and expression of known target genes for a model (HCM-BROD-0003-C71) that does not match its tumor’s MR activity profile. MR activation or deactivation are shown in red and blue, respectively. Differential expression of target genes (DEGs) for upregulated or downregulated genes is respectively orange and purple. (**e**) Gene expression of GBM markers in tumors, before model derivation. GBM tumors are grouped by the culture media that we used to derive their respective models. Each point represents a biologically independent tumor sample (CM n = 3 tumors; NSA n = 37 tumors). Box plots show the median (center line) and interquartile range (box; 25th-75th percentiles); whiskers extend to +/- 1.5 IQR, and points beyond this range are plotted as outliers. ns = not statistically significant by a two-sided Wilcoxon rank-sum test (Mann-Whitney U test). (**f**) Copy number concordance between model/tumor pairs. Blue and red regions indicate, respectively, copy number losses and gains. Cases where a model has transcriptionally diverged from its parent tumor (e.g. HCM-BROD-0002-C71) display retention of inferred copy number events, suggesting transcriptional state changes.

We next evaluated epigenetic and transcriptional fidelity (Methods (‘Transcriptional relatedness measurements’) and Supplementary Table 3). First, for the 201 tumour–model pairs for which DNA methylation profiles were available, we found that 190 paired distances (95%) were smaller than expected by random chance (false discovery rate (FDR) < 0.1; Fig. 3a and Supplementary Table 3). DNA methylation similarity was positively correlated with estimated tumour purity (Pearson’s correlation *r* = 0.33, *P* < 0.05; Extended Data Fig. 4a). Overall, 9 out of 11 models with apparent epigenetic discordance were derived from tumours for which tissue purity estimates were below 60%, which suggests that this result is due to artefactual rather than biological discordance.

We next evaluated transcriptional concordance in 297 tumour–model pairs using Celligner^31^ (Methods, ‘Transcriptional relatedness measurements: Celligner’). Celligner identified 242 pairs (81%) as significantly concordant (Fig. 3b and Supplementary Table 3). Five additional computational methods further confirmed concordance (Fig. 3c, Methods, Extended Data Fig. 4 and Supplementary Table 3). Of the 286 models assessed by at least 4 of the 6 independent similarity metrics, only 1 model (HCM-CSHL-0255-C18) was considered non-concordant by 3 methods. Overall, 263 (92%) models were identified as discordant by no more than 1 method (Fig. 3c), a result that supports their ability to recapitulate key transcriptional states. Subsequent RNA sequencing (RNA-seq) profiling of 14 additional models, expanded independently at ATCC, did not reveal significant differences (paired *t*-test, *P* = 0.13; Extended Data Fig. 4c). These data show that most HCMI models preserve the transcriptional state of their parental tumours.

Several pan-cancer and cohort-specific molecular subtype stratifications of tumour samples from The Cancer Genome Atlas (TCGA)^3^ have been proposed. These include a 106-subtype stratification based on subtypes defined in the original TCGA publications for each cohort (here, referred to as the tumour molecular pathology (TMP) subtypes^32^) and a 112-subtype stratification by multi-omics master-regulator analysis (MOMA)^33^, which is based on activities of master regulator proteins. Of all the tumour–model pairs in HCMI, a subset (*n* = 272 out of 297, 92%) could be classified on the basis of both TMP and MOMA subtypes (Supplementary Table 3).

Tumours and models from 160 (59%) and 177 (65%) of cases had identical TMP or MOMA classifications, respectively. In total, 228 tumour–model pairs (84%) were coherently classified by at least 1 of the 2 methods (Fig. 3d and Extended Data Fig. 4d). As expected, tumour–model pairs with high fidelity and identical TMP or MOMA subtypes generally displayed strong master regulator conservation (Fig. 3e). These results further confirm that most of the HCMI models are biologically relevant proxies.

Culture media have been shown to influence transcriptional states^34^. We therefore investigated this aspect and observed that GBM models propagated in NeuroCult NS-A basal (NSA) medium were significantly more similar to their parental tumours than those cultured in Propagenix conditioned medium (Fisher’s exact test, *P* = 1.5 × 10^−4^; Fig. 3f). Expression differences were driven by the differential expression of established mesenchymal and proneural GBM lineage markers^35^ (Fig. 3g, Extended Data Fig 4e and Supplementary Table 3). Consistent with these findings, gene set enrichment analysis^36^ identified EMT as differential, which suggests that the type of medium used for culture can influence the transition of GBM cells from proneural to mesenchymal^37^ (Extended Data Fig. 4f and Supplementary Table 3). We further evaluated these observed effects of medium conditions by single-nucleus transcriptional analysis (see below).

We performed medium-switching experiments (Supplementary Fig. 5a). When the GBM model HCM-BROD-0416-C71 was cultured in formulated-conditioned medium, it adopted new morphology (Fig. 3h), reduced nuclear SOX2 intensity (Supplementary Fig. 5b–d) and cell-state-related transcriptional changes (Fig. 3i and Supplementary Fig. 5e). Together, these findings indicate that serum-driven programs in GBM may exhibit plasticity. We did not observe major influences of media in other cancer model lineages beyond GBM (Supplementary Fig. 6a,b). As expected, we found that integrative master regulator and pathway analyses across seven core components of medium revealed modest transcriptional influences of serum, WNT and TGFβ inhibitor supplementation (Supplementary Fig. 6c,d).

Taken together, data from DNA-based and RNA-based analyses show that the majority of HCMI tumour–model pairs were concordant genetically (98%, 365 out of 373) and transcriptionally (92%, 263 out of 286). Consistent with previous reports^34^, media emerged as a potential contributor to occasional transcriptional discordance.

## HCMI expands model diversity

**Fig. 4:**
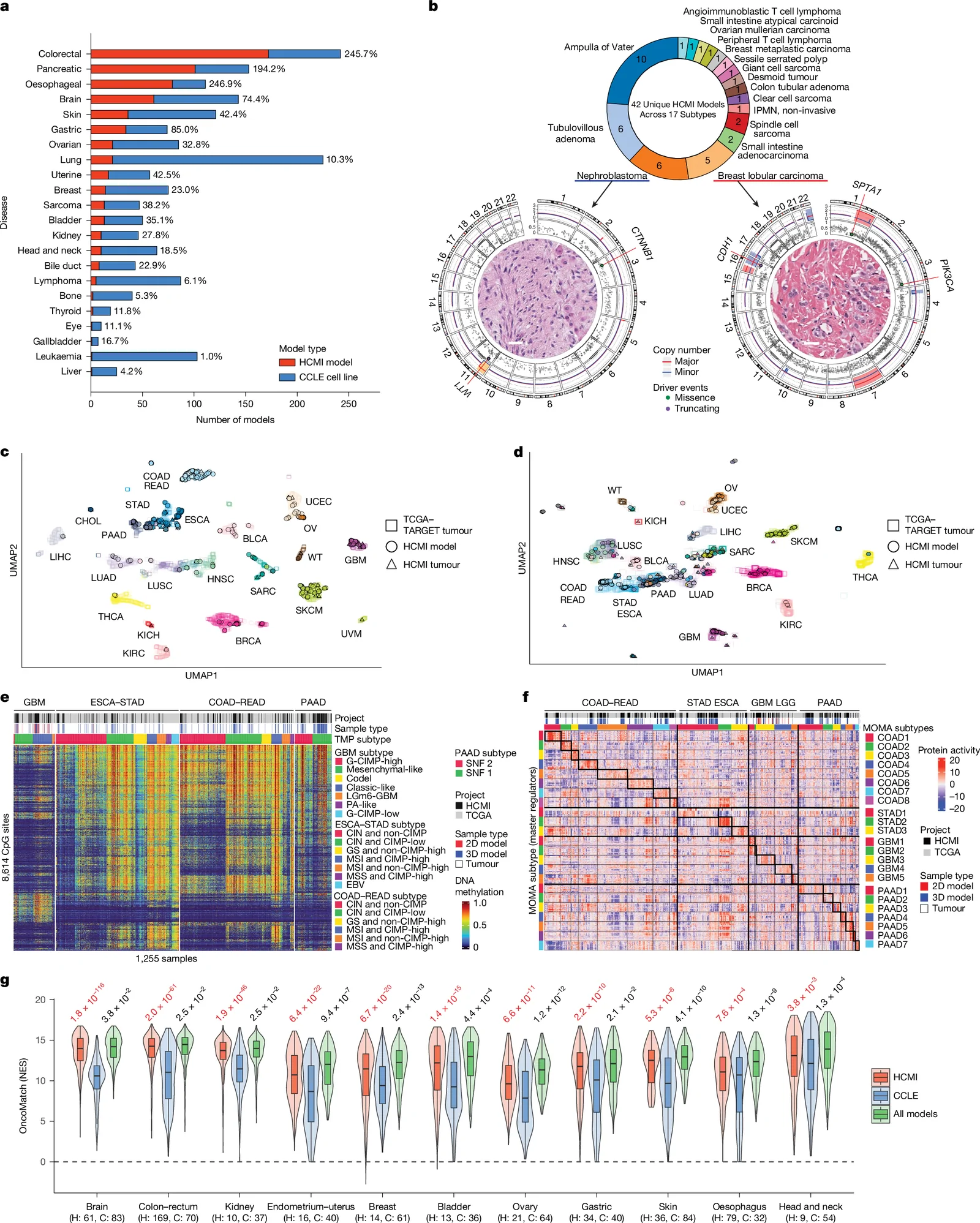
Comparison of HCMI, TCGA and CCLE. **a**, Stacked bar plot of disease-type distributions in HCMI (red) and CCLE (blue) models. Percentages indicate relative expansion achieved with HCMI versus CCLE. **b**, Top, donut chart summarizing 42 models across 17 rare subtypes exclusive to HCMI. Bottom, circos plots of rare tumour-derived models. From outer to inner: ideograms, copy number profiles and mutation patterns. Major or minor copy numbers are shown in red or blue, respectively; gains, losses and LOH in red, blue and orange, respectively. Inner tracks show variant allele frequencies (VAFs) with selected driver mutations. Central panels display corresponding histology. Histology images correspond to individual patient whole slide images. Scale bars, 50 µm. **c**, Uniform manifold approximation and projection (UMAP) plot of cancer-associated DNA hypermethylation for HCMI tumours (*n* = 155, triangles), HCMI models (*n *= 377, circles) and TCGA–TARGET tumours (*n* = 6,351, squares). Shared cancer types are shown; colours denote cancer type. **d**, UMAP of Celligner-aligned gene expression for the same samples and cancer types as in **c**, with identical shapes but for RNA. **e**, Heatmap of cancer-associated DNA hypermethylation in HCMI (89 tumours, 235 models) and TCGA (*n* = 931), grouped by TMP subtype and hierarchically clustered (*β* values: low, blue; high, red). **f**, Supervised heatmap of inferred protein activity (MOMA or OncoMatch) in HCMI (195 tumours, 439 models) and TCGA (*n* = 1,054). Columns denote samples (black, HCMI; grey, TCGA); rows show top 50 activated proteins per subtype. Sample types: tumour (white), 2D (red) or 3D (blue). **g**, Violin plots showing OncoMatch protein activity similarity (NES) of the best model in HCMI (red), CCLE (blue) or both (green). Box plots show median, 25–75th percentiles and 1.5× IQR whiskers; outliers are not shown but included in the analyses. See Extended Data Fig. 5f for remaining cohorts. Numbers on the *x* axis denote cohort sizes in the HCMI (H) and CCLE (C) datasets. Indicated *P* values represent HCMI versus CCLE and HCMI versus both (two-sided Mann–Whitney, Benjamini–Hochberg-corrected).

**Extended Data Fig. 5:**
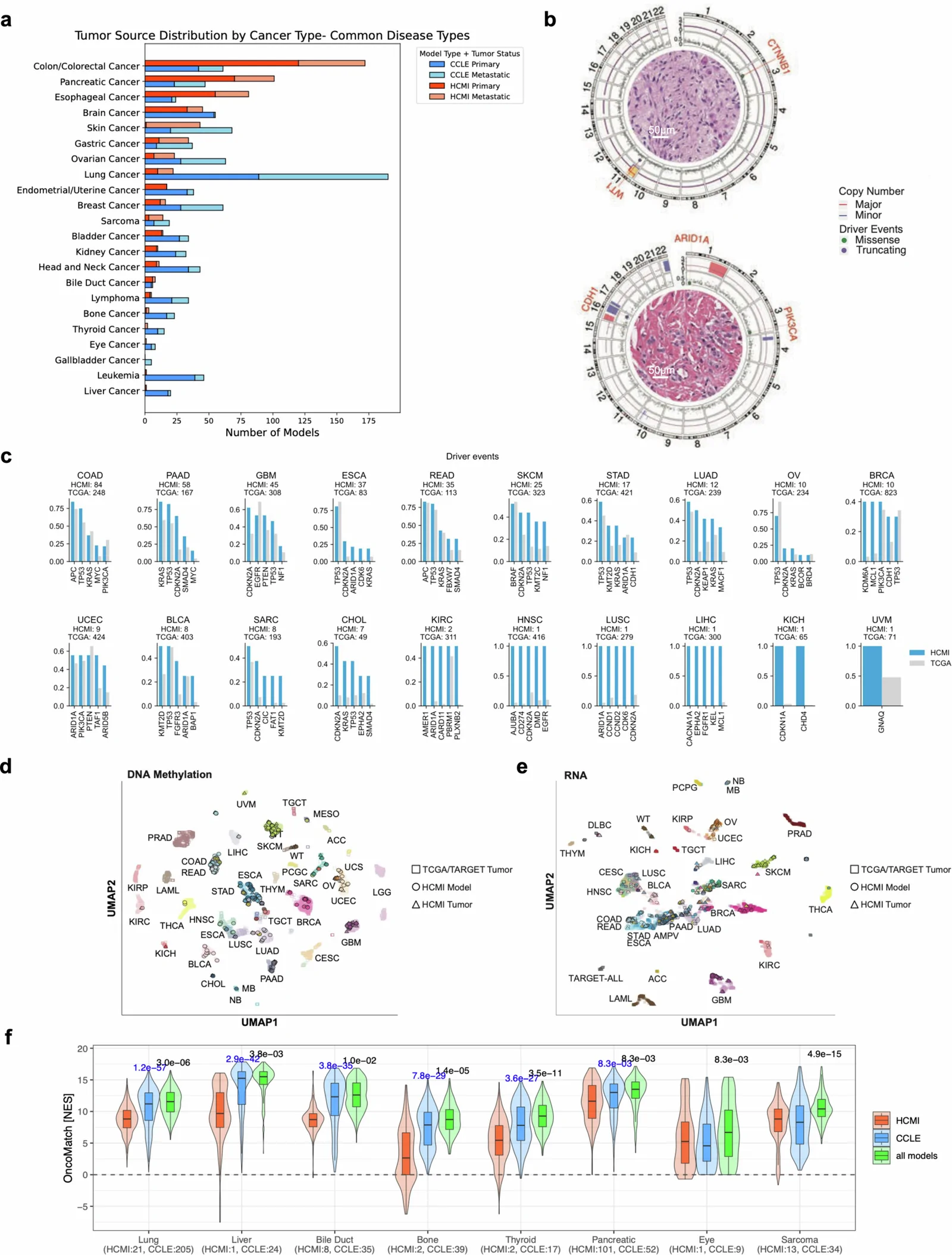
Comparison of HCMI, TCGA and CCLE. (**a**) Stacked bar plot showing the number of models across major disease types in the HCMI (red) and CCLE (blue) collections, stratified by tumor status (primary vs. metastatic). Bar lengths indicate total model counts. (**b**) Circos plots that illustrate the copy number and mutational landscapes of the matched tumors for the rare models shown in Fig. 4b (lower). From the outside moving inward, the tracks display: chromosome ideograms, copy number profiles, and mutational patterns that highlight both driver missense and truncating mutations. Major and minor copy numbers are represented respectively by red and blue lines, while copy number gains, losses, and loss-of-heterozygosity are emphasized with red, blue, and orange shadings. The innermost track shows variant allele frequencies (VAFs) of identified mutations, with key driver mutations labeled by their gene symbols. A histologic image of the tumor-of-origin is placed at the center. (**c**) Comparison of tumor-type relevant driver events in patient tumor samples for HCMI (blue) and TCGA (grey) cohorts. Extended Data Fig. 5b shows driver events, including pathogenic and likely-pathogenic SNVs, high-level amplifications, and homozygous deletions in each gene. For the HCMI cohort, patient tumor samples used to directly generate the matching model were used for the comparison. Genes associated with these driver events were curated from Bailey et al.^106^ and supplemented by expert opinion (Supplementary Table 10). (**d**) A UMAP layout of CpG sites for HCMI tumors, HCMI models, and TCGA/TARGET tumors showed cancer-associated DNA hypermethylation; all cancer types in HCMI and TCGA/TARGET projects are shown. Samples are colored according to cancer type. (**e**) UMAP projection of Celligner-aligned gene expression data for HCMI tumors, HCMI models and TCGA/TARGET tumors, colored by cancer type. All cancer types represented in HCMI and TCGA/TARGET cohorts are shown. Samples are colored according to cancer type and shapes indicate sample types. (**f**) Violin plots that display protein activity similarity, assessed by OncoMatch NES, of the best model in HCMI (red), CCLE (blue), and both (green), for each TCGA tumor across 8 cancer types (lung [nHCMI:21, nCCLE:205], liver [nHCMI:1, nCCLE:24], bile duct [nHCMI:8, nCCLE:35], bone [nHCMI:2, nCCLE:39], thyroid [nHCMI:2, nCCLE:17], pancreatic [nHCMI:101, nCCLE:52], eye [nHCMI:1, nCCLE:9], and sarcoma [nHCMI:13, nCCLE:34]) out of 19 common cancer types in both datasets. Box plots show median (center line), 25–75th percentiles, and 1.5 X IQR whiskers; data points beyond this range are considered outliers and are not shown but are included in statistical analyses. Higher NES values indicate greater similarities between a tumor and a model. Benjamini-Hochberg (BH) corrected p-values from two-sided Mann-Whitney U-tests comparing NES distributions between HCMI (red) and CCLE (blue) are annotated above the violin plots. These comparisons are shown only for the superior dataset within each cancer type (lung: p=1.2x10-57; liver: p=2.9x10^−42^; bile duct: p=3.8x10^−35^; bone: p=7.8x10^−29^; thyroid: p=3.6x10^−27^; pancreatic: p=8.3x10-3). Additionally, p-values displayed above the 'All models' (green) plots indicate statistical significance between the top-performing dataset and the combined group (HCMI + CCLE), calculated via two-sided Mann-Whitney U-tests with BH correction (lung: p=3.0x10^−6^; liver: p=3.8x10^−3^; bile duct: p=1.0x10^−2^; bone: p=1.4x10^−5^; thyroid: p=3.5x10^−11^; pancreatic: p=8.3x10^−3^; eye: p=8.3x10^−3^; sarcoma: p=4.9x10^−15^). The violin plots were sorted in the order of decreasing significance of having a greater NES in CCLE models than in HCMI.

Given this fidelity in the subset of HCMI models with matched tumours, we proceeded to evaluate the entire HCMI resource (*n* = 643) and compared results with the Cancer Cell Line Encyclopedia (CCLE)^1^ (*n* = 1,377). HCMI doubles the number of publicly available colorectal, pancreatic and oesophageal cancer models, and increases the representation of other cancers by a median of 25.4% (range of 1.2%–85%) (Fig. 4a, Extended Data Fig. 5a and Supplementary Table 4).

Notably, the HCMI resource includes 153 models of rare cancers, 42 of which represent 17 histologically distinct cancer subtypes that are not included in the CCLE, such as angioimmunoblastic T cell lymphoma, desmoid tumour, small intestine atypical carcinoid, peripheral T cell lymphoma-not otherwise specified, and clear-cell sarcoma. HCMI also includes models of pre-malignant lesions, including sessile serrated colon polyp, colon tubular–tubulovillous adenoma and intraductal papillary mucinous neoplasm of the pancreas (Fig. 4b, Extended Data Fig. 5b and Supplementary Table 4).

To assess whether the HCMI resource recapitulated tumour diversity at a cohort level, we compared genomic, epigenomic and transcriptional features with cohorts from TCGA^8^ and the therapeutically applicable research to generate effective treatments (TARGET) programme (https://www.cancer.gov/ccg/research/genome-sequencing/target). Driver alterations were largely conserved in COAD, PAAD, ESCA and breast cancer (BRCA) cohorts (Extended Data Fig. 5b). DNA methylation and transcriptional profiles showed strong alignment with histology-matched tumours (Fig. 4c,d and Extended Data Fig. 5d,e). Unsupervised clustering of the four largest HCMI model cohorts (COAD–READ, ESCA–stomach tubular adenocarcinoma (STAD), GBM and PAAD) effectively recapitulated TMP^32^ subtypes (Fig. 4e and Methods (‘Transcriptional relatedness measurements: MultiClass pair classification-based distance calculation’)). Using MOMA^33^, HCMI samples were classified into eight COAD, three STAD, five GBM and seven PAAD subtypes on the basis of master regulator activity (Fig. 4f and Supplementary Tables 5–7). Models and tumours broadly spanned the full spectrum of TCGA-defined subtypes, with over-representation for several substates (Fig. 4f, Methods (‘Transcriptional relatedness measurements: MOMA subtype comparison, OncoMatch analysis’) and Supplementary Tables 5–7).

Recent studies have shown that the OncoMatch algorithm^38,39^ can help identify translational-relevant models on the basis of conservation of master regulator activity^33^. Thus, for each TCGA sample, we tested whether HCMI, CCLE or the combined repositories (HCMI–CCLE) had at least one high-fidelity, histology-matched model (Fig. 4g and Extended Data Fig. 5f). HCMI outperformed CCLE in modelling 11 out of 19 cancer types (Benjamini–Hochberg-adjusted *P* < 0.05 by a two-sided Mann–Whitney *U*-test). By contrast, CCLE outperformed HCMI in six cancer types; however, four out of these six, were HCMI cohorts with a small number of models (*n* ≤ 10) (Extended Data Fig. 5f).

When HCMI and CCLE models were combined, the fraction of TCGA tumours for which a high-fidelity model was available increased, resulting in 77.3% (*n* = 6,902) of TCGA tumours having at least 1 high-fidelity model (normalized enrichment score (NES) ≥ 10). Thus, HCMI and CCLE are complementary resources for modelling human cancers.

## Single-cell analysis of tumour–model pairs

**Fig. 5:**
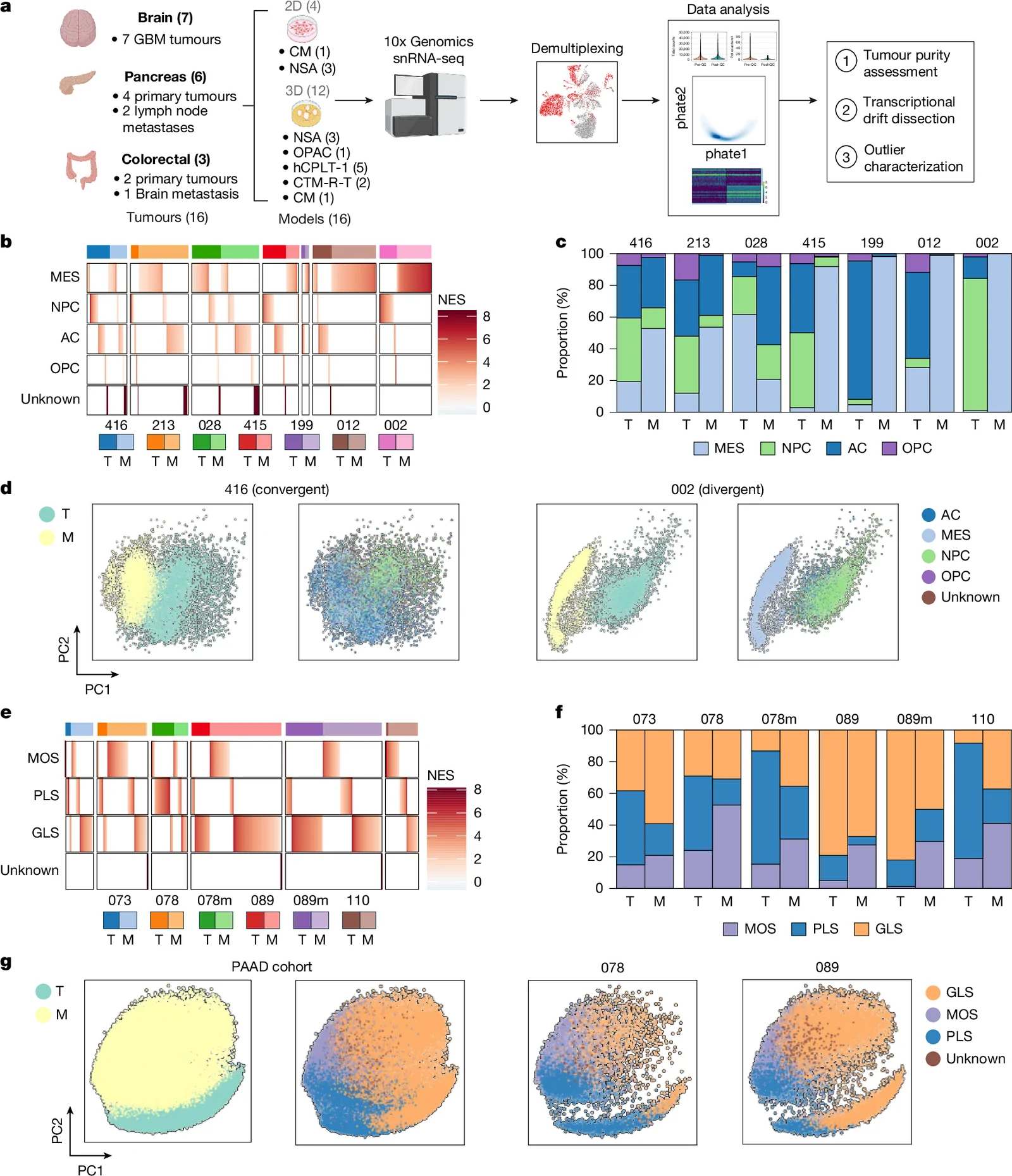
Assessment of HCMI model drift by single-nucleus RNA studies. **a**, Analysis workflow for single-nucleus RNA-Seq (snRNA-seq) data. **b**, Heatmap showing the enrichment analysis in malignant cells (cells: total *n* = 105,944, tumour 39,405; model *n* = 66,539) across seven GBM tumour–model pairs. Rows indicate mesenchymal (MES)-like, neural progenitor cell (NPC)-like, oligodendrocyte progenitor cell (OPC)-like and astrocyte cell (AC)-like cellular states^51^. Columns indicate samples, grouped by pair and sorted sequentially as tumours (T) and matched models (M). Sample key: 416, HCM-BROD-0416-C71; 213, HCM-BROD-0213-C71; 028, HCM-BROD-0028-C71; 415, HCM-BROD-0415-C71; 199, HCM-BROD-0199-C71; 012, HCM-BROD-0012-C71; and 002, HCM-BROD-0002-C71. **c**, Bar plots displaying malignant cellular states across seven GBM tumour–model pairs^51^, sorted sequentially as tumours and matched models. Cells labelled as ‘Unknown’ in **b** are not shown. **d**, Principal component analysis (PCA) of cell-type composition in conserved (416) and divergent (002) GBM tumour–model pairs. 416 (model, 3D; media, NSA) shows cell-state conservation; 002 (model, 2D; media, CM) shows drift towards a MES-like state. **e**, Heatmap showing enrichment analysis in malignant cells (total *n* = 56,526; tumours, *n* = 16,226, models, *n* = 40,300) across six PAAD tumour–model pairs. Rows indicate transcriptional states: morphogenic-like (MOS), primitive-like (PLS) and gastrointestinal-like (GLS)^58^. Columns indicate single nucleus samples and are grouped by pair and sorted sequentially as tumours or matched models. Sample key: 073, HCM-CSHL-0073-C25; 078 and 078m, HCM-CSHL-0078-C25, primary and metastatic, respectively; 089 and 089m, HCM-CSHL-0089-C25, primary and metastatic, respectively; 110, HCM-BROD-0110-C25. **f**, Bar plots displaying malignant cellular states across six PAAD tumour–model pairs^43^, sequentially sorted as tumour and matched model. Malignant cells comprise PLS, GLS and MOS subpopulations or cell types. **g**, PCA showing malignant subpopulations across the PAAD cohort (left) and for tumour–model pairs 078 and 089 (right). For the right panels, parental tumours and their matched models are jointly plotted, highlighting preservation of malignant subpopulations albeit at different frequencies. Schematic in **a** created in BioRender; Zanella, L. https://biorender.com/aqee5hf (2026).

We proposed that single-cell heterogeneity may account for at least some of the observed divergence between certain models and their parental tumours. We selected a representative subset of 16 tumour–model pairs for single-nucleus transcriptomic analysis, including 7 GBM, 6 PAAD and 3 COAD pairs (Fig. 5a). GBM models included both 2D models (*n* = 4), of which one was propagated in conditioned medium and three in NSA medium, and 3D models (*n *= 3), which were cultured in NSA medium. Selected PAAD models included 3D organoids established in OPAC (*n* = 1) and hCPLT-1 media (*n* = 5) (Supplementary Table 8). In total, 217,749 single-nucleus RNA sequencing (snRNA-seq) profiles were generated and analysed, including 117,257 from models and 100,492 from parental tumours.

**Extended Data Fig. 6:**
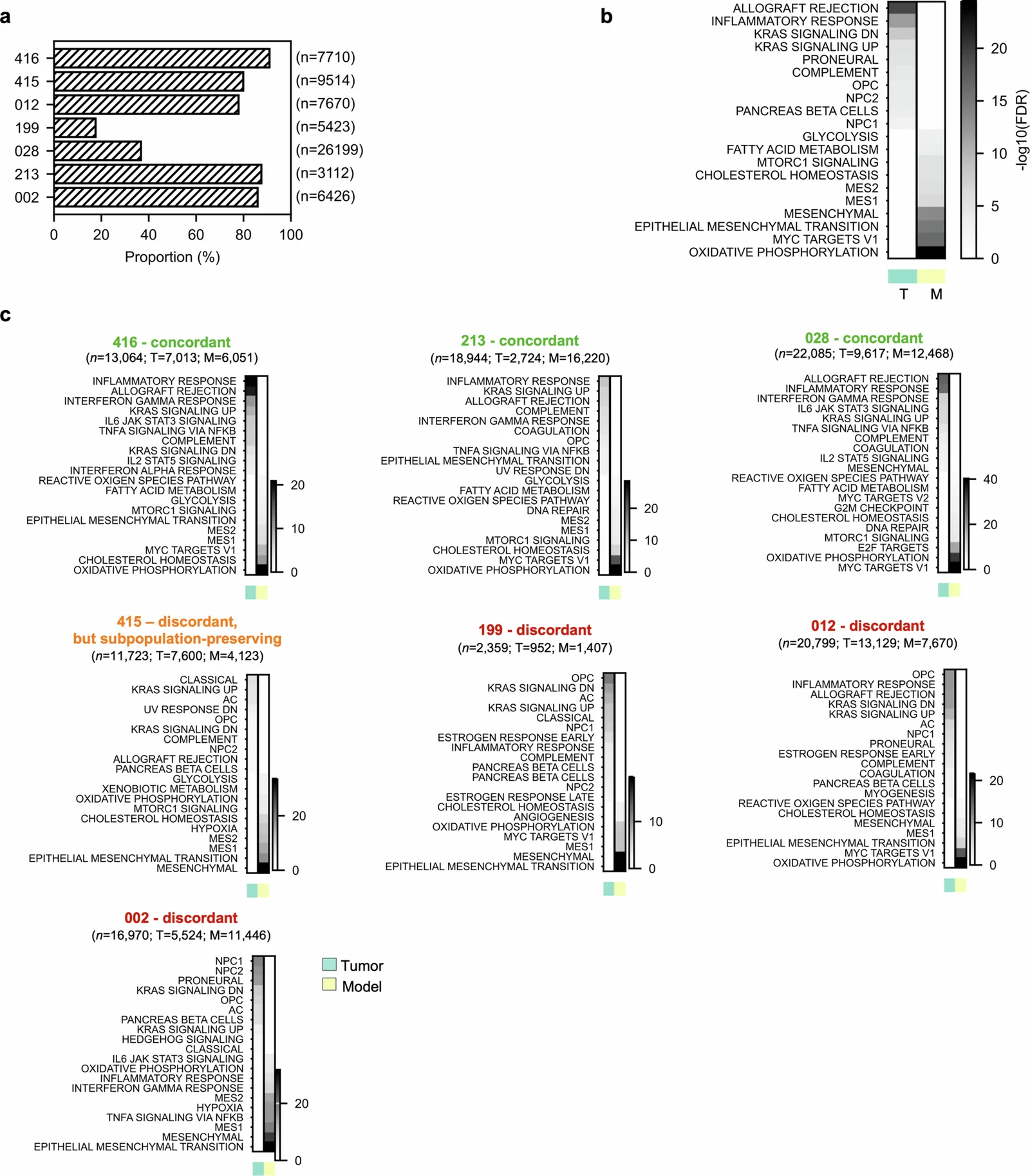
Single-nucleus characterization of tumor and models from the GBM cohort. (**a**) Proportion of malignant cells in GBM tumors, profiled using snRNA-seq. (**b**) Heatmap highlighting the top differentially regulated pathways (-log10(FDR)) in malignant cells (total cells: *n* = 105,944; tumors: 39,405; models: 66,539) across all GBM tumors (left) versus models (right). Pathways are derived from the MSigDB Hallmark gene set and glioma-intrinsic expression subtypes as defined by Wang *et al*.^170^ and Neftel *et al*.^40^. (**c**) Heatmaps highlighting the top differentially regulated pathways (-log10(FDR)) in malignant cells from individual tumor/model pairs. Pathways are derived from the MSigDB Hallmark gene set and glioma-intrinsic expression subtypes as defined by Wang *et al*.^170^ and Neftel *et al*.^40^.

For GBM tumour–model pairs, we annotated single nuclei using four cell states, as previously reported^40^. Transformation to the mesenchymal state is often associated with an immunosuppressive microenvironment and therapy resistance^41,42^. Cell states of three parental tumours were effectively retained in their cognate models, whereas the remaining four pairs showed different levels of transcriptional divergence (Fig. 5b,c). Specifically, three models (HCM-BROD-0012-C71, HCM-BROD-0002-C71 and HCM-BROD-0199-C71) presented almost a complete shift to a mesenchymal state, whereas the remaining (HCM-BROD-0415-C71) presented at least partial recapitulation of heterogeneity. Most parental tumours showed only minimal stromal infiltration, consistent with the purity estimates, which reduces the possibility of TME-dependent differences in transcriptional state (Extended Data Fig. 6a). In support of the above-described results from analyses at the bulk expression level (Fig. 3g), models cultured in NSA medium (Fig. 5d) retained more heterogeneity of the parental tumour. By contrast, those cultured in conditioned medium (Fig. 5d) showed an almost complete shift towards a mesenchymal state. Consistent with the models generated, pathway analysis of malignant cells showed analogous changes in proliferative, metabolic and inflammatory pathways (Extended Data Fig. 6c and Supplementary Table 9). By contrast, the main differences in the divergent model were related to the shift from a progenitor to a proneural state (Extended Data Fig. 6c and Supplementary Table 9).

**Extended Data Fig. 7:**
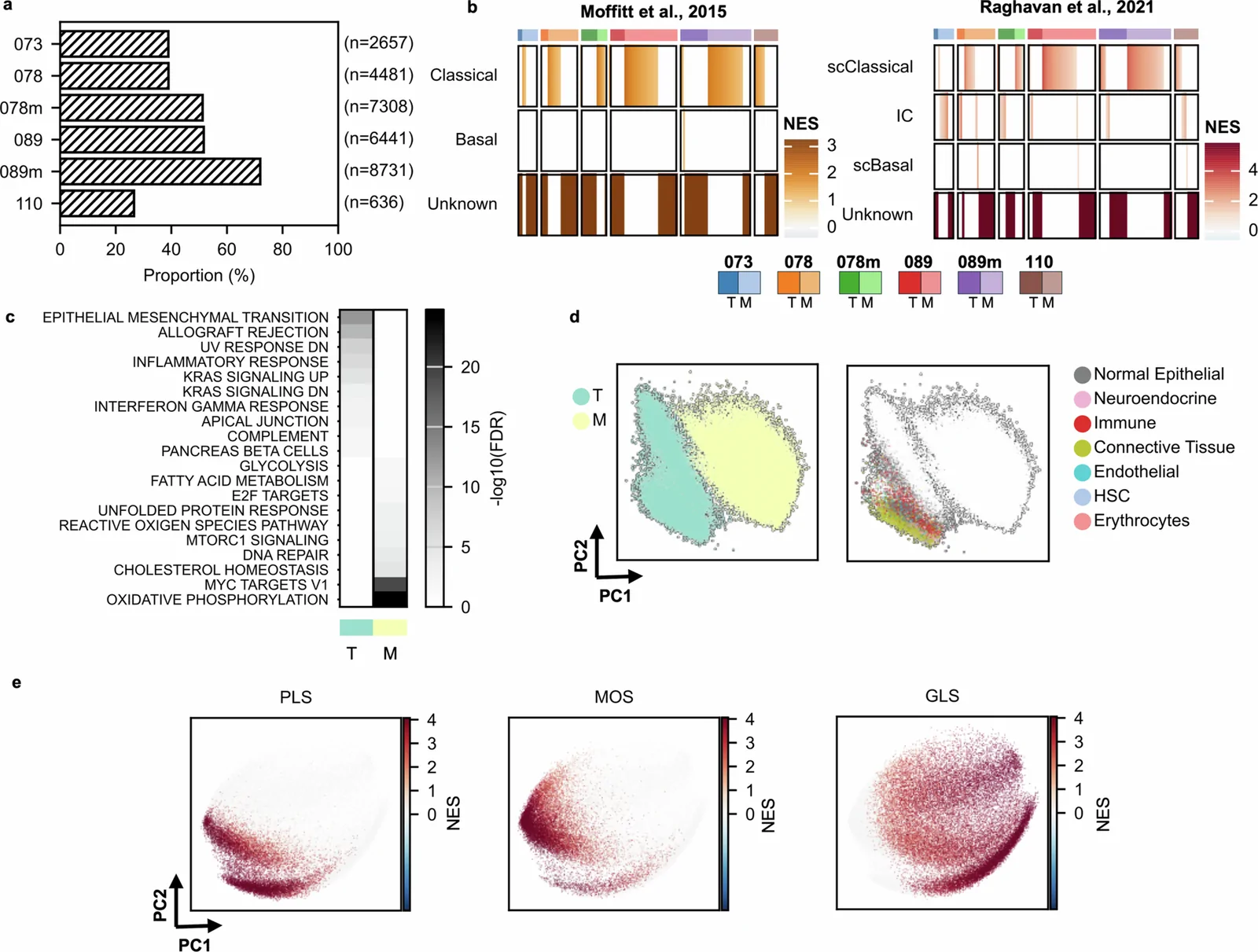
Single-nucleus characterization of tumor and models from the PAAD cohort. (**a**) Proportion of malignant cells in PAAD tumors profiled using snRNA-seq. (**b**) Heatmap showing enrichment analysis in malignant cells (total: *n* = 56,526; tumors: 16,226; models: 40,300) across six PAAD tumor/model pairs profiled at single-nucleus resolution. Rows indicate bulk and single-cell subtypes defined in studies by Moffitt *et al*.^163^ and Raghavan *et al*.^34^. The “Unknown” designation reflects NES values that were not significant. Columns represent single nuclei grouped by tumor/model pair and sorted sequentially as tumors and matched models. NES: Normalized Enrichment Score. Sample key: 073: HCM-CSHL-0073-C25; 078 and 078m: HCM-CSHL-0078-C25, primary and metastatic; 089 and 089m: HCM-CSHL-0089-C25, primary and metastatic; 110: HCM-BROD-0110-C25. (**c**) Heatmap highlighting the top differentially regulated pathways (-log10(FDR)) in malignant cells (*n* = 56,526; tumors: 16,226; models: 40,300) across all PAAD tumors (left) versus models (right). Pathways are derived from the MSigDB Hallmark gene sets. (**d**) PCA visualization of the tumor microenvironment in the PAAD snRNA-seq cohort. Non-malignant cell populations (e.g., stromal and immune cells) within tumor samples are highlighted, while malignant cells in tumors and models are left blank. (**e**) NES for the PLS, MOS and GLS transcriptional subtypes in malignant nuclei (*n* = 56,526; tumors: 16,226; models: 40,300) across the PAAD snRNA-seq cohort. Only malignant nuclei assigned to their corresponding subtype are displayed.

**Extended Data Fig. 8:**
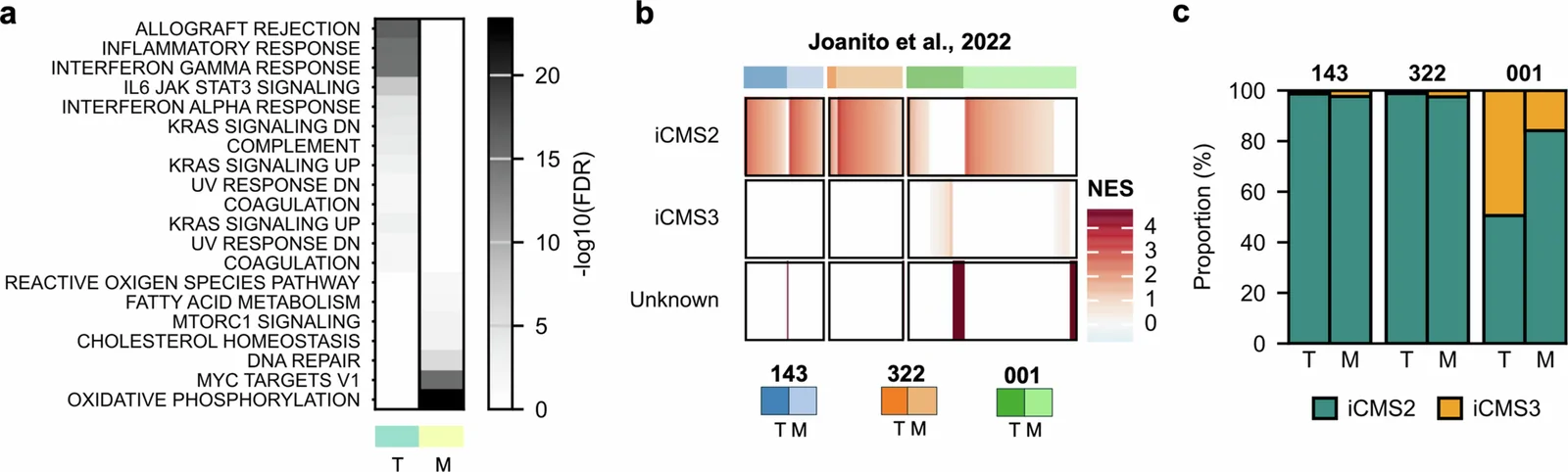
Single-nucleus characterization of matched COAD tumor/model pairs. (**a**) Heatmap highlighting the top differentially regulated pathways (-log10(FDR)) in malignant cells (total cells *n* = 12,468; tumor cells: 4,184; model cells: 8,284) across all COAD tumors (left) versus models (right). Tested pathways comprise the MSigDB Hallmark gene sets. (**b**) Heatmap showing the enrichment of differentially expressed genes in snRNA-seq profiles representing malignant cells from three COAD tumor/model pairs. Rows represent iCMS2 and iCMS3 cellular states^166^; columns represent individual snRNA-seq profiles, grouped by tumor/model pair and sorted sequentially by statistical significance. NES is the Normalized Enrichment Score. Sample keys are as follows: Sample 143 (HCM-BROD-0143-C20), sample 322 (HCM-CSHL-0322-C20), sample 001 (HCM-BROD-0001-C18). (**c**) Barplots displaying the fraction of malignant cells classified as iCMS2 and iCMS3 for each tumor/model pair^166^. Samples labeled as “Unknown” in panel (**b**) are not shown. T = tumor; M = model.

In PAAD, stromal subpopulations present in tumours were lost during model generation (Extended Data Fig. 7a,d). Classification using recently proposed subtypes^43^ effectively stratified single cells from HCMI tumour–model pairs (Fig. 5e,f). Morphogenic-like and primitive-like populations showed modest shifts in abundance, whereas qualitative parental tumour heterogeneity was largely preserved across models, except for HCM-BROD-0110-C25, which produced too few nuclei for comparison (*n* = 169) (Fig. 5f,g). Changes in pathway activity were consistent with culture conditions, including proliferation and metabolism (Extended Data Fig. 7c). Pathway analysis for the three profiled tumour–model pairs from the COAD cohort was highly consistent with findings from other cohorts (Extended Data Fig. 8). Taken together, these data suggest that HCMI models retain cell-state heterogeneity at the single-cell level in many instances.

## Translational utility of HCMI models

**Extended Data Fig. 9:**
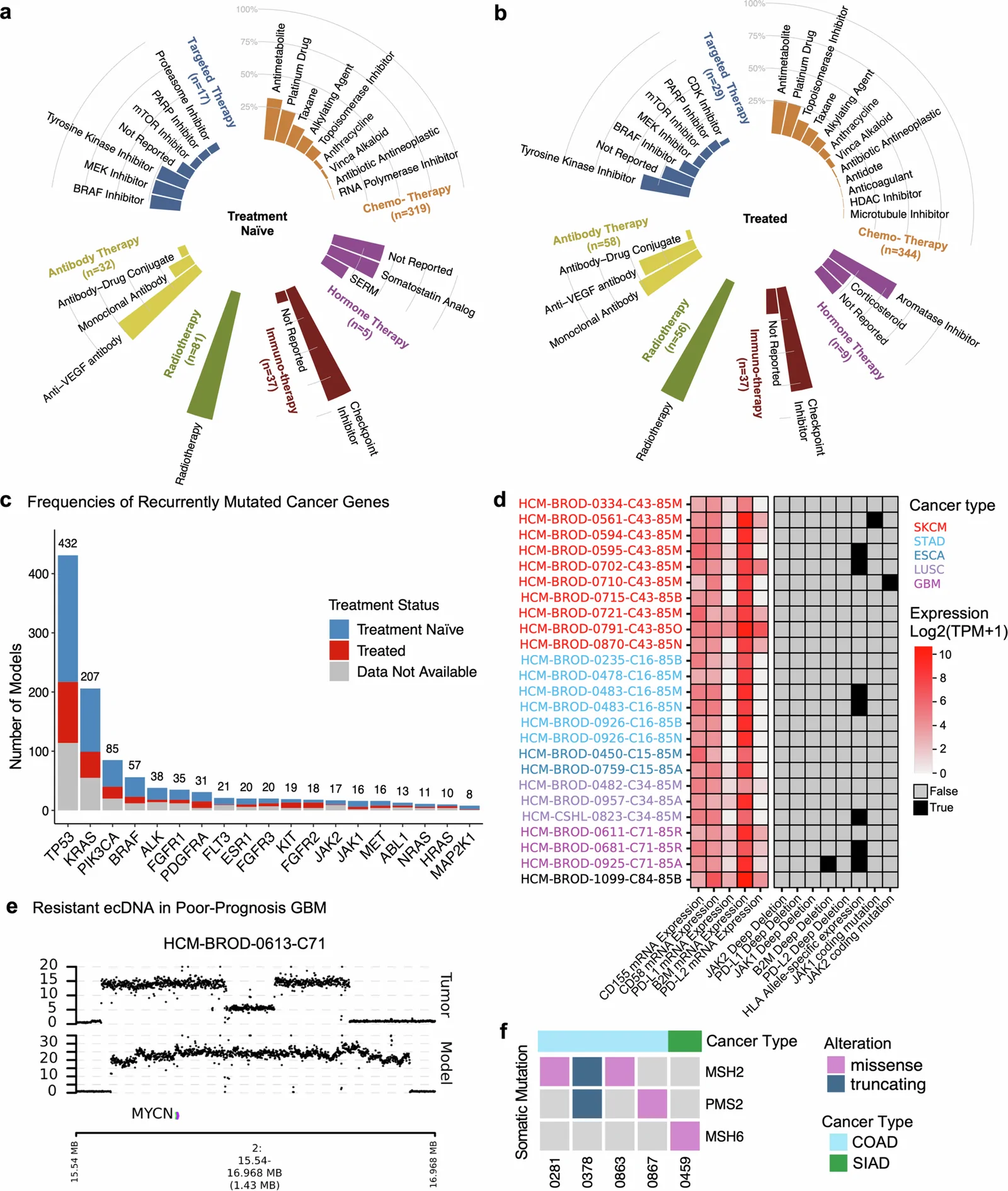
Characterization of HCMI treatment classes, resistance mechanisms, and germline predispositions. (**a,b**) The therapeutic classes within the HCMI collection. Each therapeutic class, represented by a distinct color, indicates the frequency of models associated with specific mechanisms of action; note that a single model may be represented across multiple treatment mechanisms. (**c**) Frequencies of resistance driver mutations across treated and treatment-naïve models. (**d**) Heatmap depicting the expression and mutation status of alteration in key regulators of immune response in models derived from patients previously treated with immunotherapy. (**e**) Putative reconstruction of a MYCN-amplifying ecDNA detected in a poor-prognosis GBM model/paired tumor (HCM-BROD-0613-C71). (**f**) An oncoprint that summarizes model somatic variants in hereditary Lynch syndrome cases.

We next integrated deidentified patient clinical data with molecular data to identify possible translational opportunities for the use of HCMI models to study therapeutic resistance. The HCMI collection includes models that are derived from patients with 491 treatment events after model generation (naive models) (Extended Data Fig. 9a) and 533 treatment events before model generation (post-treatment models) (Extended Data Fig. 9b). Overall, they represent 6 therapeutic classes that encompass 33 distinct mechanisms of action (Extended Data Fig. 9a,b).

**Fig. 6:**
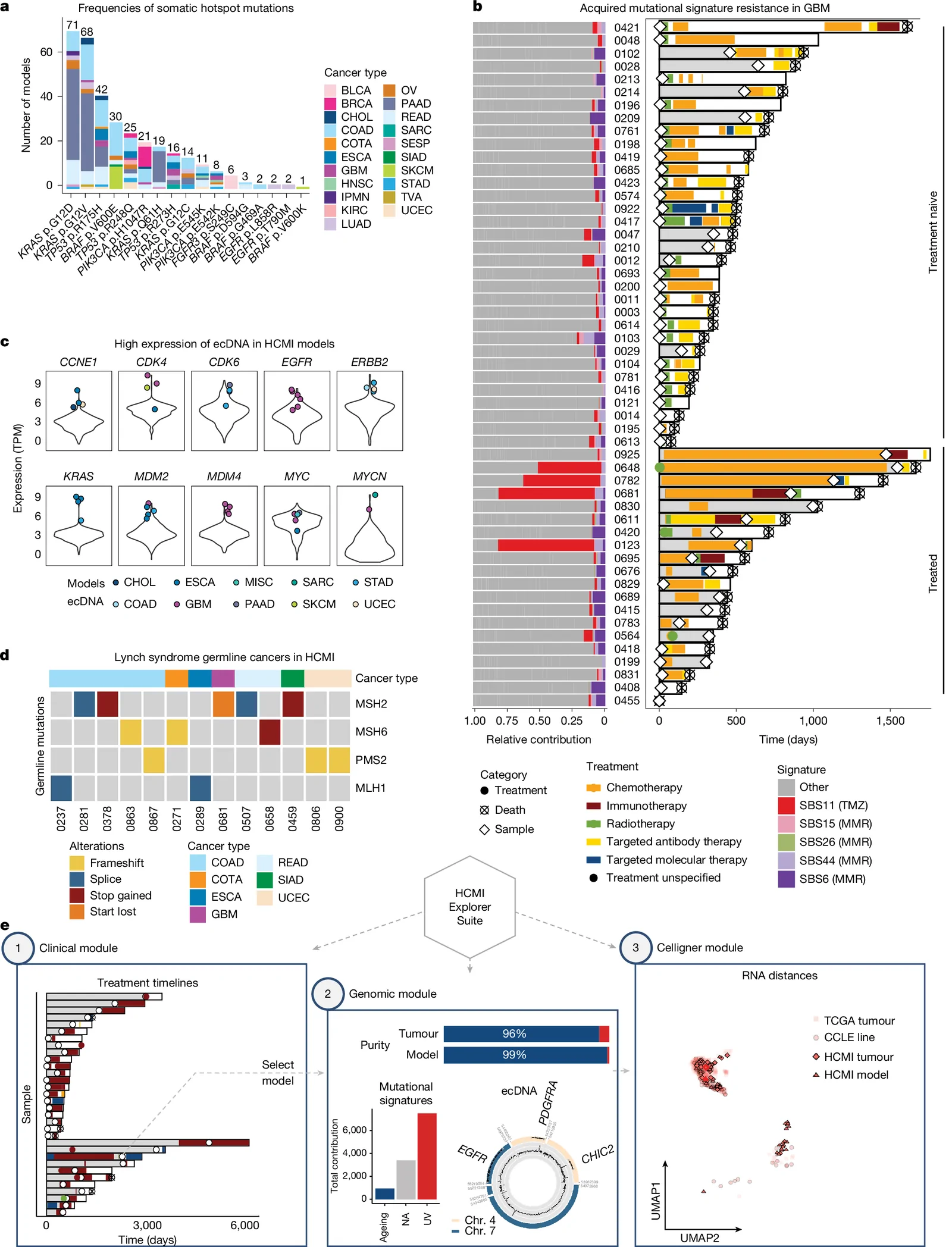
Translational utility of the HCMI resource. **a**, Frequencies of HCMI model resistance hotspot mutations across cancer types. **b**, Swimmer plot for the GBM cohort, highlighting the relative contributions of each sample to the MMR–temozolomide (TMZ) mutational signature. **c**, Violin plots depicting the high expression of recurrent ecDNA-amplified oncogenes in cancer models. Each panel shows a specific oncogene (for example, *MYCN*, *KRAS* or *EGFR*); coloured points represent the model cancer type. Each violin indicates the distribution of gene expression across all models. Sample–gene combinations with log_2_[transcripts per million (TPM)] ≤ 0 are excluded from the plot. **d**, Oncoprint plot summarizing the pathogenic germline variants in potential Lynch syndrome HCMI cancers. **e**, Overview of the HCMI Explorer Suite Shiny application. The Clinical module (1) displays interactive swimmer plots, revealing treatment timelines for the selected cancer type (or types). The Genomic module (2) visualizes the selected purity (%), mutational signatures and the detected ecDNA of the model. The Celligner module (3) shows the RNA distance UMAP plot that compares the selected HCMI model and its parent tumour to external TCGA and CCLE datasets.

We next identified models in our collection that had acquired known driver and hotspot resistance mutations^44–46^ (Fig. 6a and Extended Data Fig. 9c). In both naive and treated models, we found RAS family mutations (*KRAS*, *NRAS* and *HRAS*) in 228 models, recurrent alterations in *PIK3CA* (*n* = 85) and *FGFR* mutations (*n* = 54), among others, including 432 *TP53* mutations (Extended Data Fig. 9c). This analysis identified a total of 234 models with variants reported in the COSMIC resistance reference set^47^ (Fig. 6a).

In total, 82 HCMI models were from patients who received immunotherapy and 25 models were established from tumours that had previously been exposed to an immunotherapeutic. With respect to biomarkers relevant to immunotherapy resistance, we observed an expected range of expression of *PDL1* and *PDL2* and *HLA* allele-specific expression loss. Notably, one melanoma model, HCM-BROD-0710, had a *JAK2*.p1013F mutation (Extended Data Fig. 9d), a rare immune-evasion alteration^48^. These findings highlight the utility of HCMI models and data in dissecting molecular mechanisms of cell-intrinsic responses to immunotherapy.

Previous studies have shown that intrinsic and acquired resistance to the chemotherapy temozolomide is associated with mismatch repair (MMR) deficiency^49–51^. Examination of the GBM models revealed the presence of MMR mutational signatures linked to intrinsic resistance (SBS6, SBS15, SBS26 and SBS44) as well as acquired MMR deficiency associated with temozolomide resistance (SBS11) (Fig. 6b). A review of treatment histories showed that HCM-BROD-0648-C71, HCM-BROD-0782-C71 and HCM-BROD-1123-C71 exhibited a high number of treatment cycles of temozolomide exposure (Fig. 6b) and pathogenic mutations in MMR genes (for example, *MSH6*). The patient from which HCM-BROD-0681-C71 was generated had a germline MMR variant (in *MSH2*), combined with the SBS11 signature, which was also been associated with long-term temozolomide exposure (820 days) (Fig. 6b). Notably, although the tumour from which HCM-BROD-0925-C71 was generated was subjected to long-term temozolomide exposure (1,484 days), the model lacked the temozolomide-associated SBS11 signature. Further review of the integrated clinical data revealed that this patient’s tumour had an unmethylated *MGMT* promoter (*MGMT* proficient), which confers upstream resistance to temozolomide. After exploring ecDNA events with clinical relevance, we observed that the GBM model HCM-BROD-0613-C71 exhibited a *MYCN* ecDNA amplification and high *MYCN* expression (Fig. 6c and Extended Data Fig. 9e). These findings highlight the ability to use the linked clinical and molecular HCMI data to study resistance.

After functional validation, models with the SBS11 signature exhibited significantly attenuated temozolomide sensitivity compared with non-SBS11 counterparts (Wilcoxon *P* < 0.05), independent of *MGMT* promoter methylation status (Supplementary Fig. 7a–c). These results confirm that mutational signatures of acquired resistance translate into functional resistance^51,52^. The *MYCN*-amplified model HCM-BROD-0613-C71 also displayed temozolomide resistance consistent with an unmethylated *MGMT* promoter (Supplementary Fig. 7a).

Finally, we leveraged germline data to interrogate models related potentially to cancer predisposition syndromes. We identified 13 patients with germline mutations consistent with Lynch syndrome variants, including 5 models with loss-of-function pathogenic variants in *MSH2*, *MSH6*, *MLH1* or *PMS2* (Fig. 6d and Extended Data Fig. 9f). We conducted survival analyses for colorectal cancers among cases associated with Lynch syndrome and noted that these cases had a favourable prognosis (Supplementary Fig. 8). Thus, HCMI models are likely to be valuable for the functional study of germline-driven cancer predisposition disorders.

To facilitate exploration of the clinical and molecular features and to support translational research, we developed the HCMI Explorer Suite (https://appshare.cancer.gov/HCMI_Explorer_Suite/), an interactive Shiny tool for integrating and visualizing HCMI data (Fig. 6e and Supplementary Fig. 9). The tool provides access to patient treatment histories, model development timelines, genomic profiles and Celligner transcriptional mapping (Fig. 6e), which enables translationally focused users to select optimal models.

## Discussion

Here we presented the generation and comprehensive molecular characterization of 665 human cancer models and 581 tumours (413 that produced models and 168 from failed derivation attempts), together with detailed clinical characterization of the patients who donated tumour specimens. Notably, such models maintained high concordance with the primary tumour with respect to DNA alterations (97.8%) and RNA expression (92%), despite being cultured for a year or longer ex vivo. This fact and the scale of the resource provide new opportunities for the scientific community to investigate the therapeutic consequences of a wide range of genetic alterations and transcriptional states. For example, the two companion papers^26,27^ demonstrate that the HCMI models are indeed suitable for high-throughput characterization of genetic dependencies using genome-wide CRISPR–Cas9-based screens and systematic assessments of drug sensitivity.

Cell lines and organoid cultures have important limitations, several of which are documented herein. Moreover, a growing body of literature^34,53–55^ indicates that components of tissue culture media are key drivers of cell-state plasticity in cancer models. For example, our demonstration that NSA medium maintains the transcriptional states of GBM tumours, whereas other media do not, are reminiscent of recent reports that describe a similar influence of growth medium on cell states of pancreatic cancer models^34^. Such cell-state plasticity can be reversible, which suggests that models that have transcriptionally shifted can be converted into a state that is more faithful to the tumour by changing ex vivo growth conditions before experimentation.

The HCMI models are well suited to the systematic, scalable discovery of new targets and mechanisms of cancer. However, the absence of stromal and immune cells in the HCMI models limits their applicability in the study of immune responses; other model formats will be needed to fill this gap. Despite this limitation, in a subset of 25 models derived from patients previously treated with immunotherapy, we examined features previously associated with resistance phenotypes. Although these models lack an immune microenvironment, they may serve as a resource for studying tumour-intrinsic mechanisms of resistance and for identifying alternative therapeutic strategies.

Although most models were highly concordant with their parental tumours, approximately 4–8% of models seemed to be less concordant. For 16 tumour–model pairs, we performed snRNA-seq to clarify mechanisms of discordance at the single-cell level. These data supported the existence of three distinct mechanisms: (1) purification, in which loss of stromal and immune infiltrates present in the parent tumour are typically absent in cancer models; (2) selection, in which there is selective evolutionary outgrowth of a minor cancer subclone from the primary tumour; and (3) epigenomic plasticity, in which a transcriptional shift alters the relative representation of malignant cell states in models compared with the tumour of origin (in some cases, this was due to the culture medium). Despite the epigenomic plasticity of the cell state of some models, most models retained some representation of each of the cell states found in the parental tumour. This result suggests that single-cell analysis of HCMI models could gauge the influence of the most defined cellular states in response to drugs and to other perturbations.

Recent reports have highlighted the under-representation of publicly available cancer models of rare cancers, rare genotypes and cancers from ethnic and ancestry groups other than those of European origin^56,57^. We took specific steps to address those challenges. These efforts were partially successful; the HCMI includes 153 rare cancer models and 38 models from patients of recent African descent.

However, we acknowledge that this diversity is insufficient and that further efforts are needed internationally to develop cancer models that avoid historical biases to ensure that research benefits all patients with cancer.

With the inclusion of the CCLE cell-line resource, over 2,000 publicly available cancer models now exist in the public domain (https://cancermodels.org/). However, in a post-TCGA and International Cancer Genome Consortium (ICGC)^4^ era, we recognize the scale of diversity of tumour subtypes, genetic alterations and phenotypic states that dictate the prognosis and treatment response of patients with cancer. Thus, we consider that the HCMI effort is only the beginning. That is, an order of magnitude expansion in publicly available cancer models may be needed to fully capture the heterogeneity of human cancers and to power the future of drug discovery for all patients.

## Methods

### Biospecimen processing and quality control

Before extracting nucleic acids, pathology quality control (QC) was performed on all frozen and FFPE tumours and normal specimens. From frozen tissue, a 30 mg or smaller piece was prepared, or an equivalent amount of scrolls were cut from a FFPE block, with sections stained with haematoxylin and eosin taken from the top and bottom of each specimen. The stained slides were scanned and their pathology reviewed to confirm that the tumour was consistent with the reported histology, and to assess the per cent tumour nuclei, per cent necrosis and other pathological features. The tumour nucleus and necrosis percentages from the top and bottom slides were averaged, and tumour specimens with an average of ≥50% tumour nuclei and ≤20% necrosis were submitted for nucleic acid extraction. Normal tissues were rejected if they had any detectable tumour cells.

DNA was extracted from normal blood and saliva specimens. DNA and RNA were co-extracted from tumours, solid normal tissues and cancer models. Before extraction, cancer models were first washed with ice-cold PBS to remove residual Matrigel. Frozen tissues and cancer models were homogenized using a Qiagen TissueLyser, and RNA and DNA were extracted using a modification of the DNA/RNA AllPrep kit (Qiagen). The homogenate was applied to a Qiagen DNA column, and the flow-through was processed using a mirVana miRNA Isolation kit (Ambion). FFPE specimens were deparaffinized and cells were lysed. The pellet underwent DNA extraction using an AllPrep FFPE kit (Qiagen), whereas the supernatant underwent RNA extraction using a Highpure miRNA kit (Roche). Blood specimens were extracted using a QiaAmp DNA Blood Midi kit (Qiagen), and saliva was extracted using a Gentra Puregene Buccal Cell kit (Qiagen).

DNA was quantified by PicoGreen assay and RNA was quantified by measuring the absorbance at 260 nm with a UV spectrophotometer. DNA quality was assessed by 1% agarose gel electrophoresis to confirm high-molecular-weight fragments. RNA was analysed using a RNA6000 Nano assay (Agilent) on an Agilent Bioanalyzer, which returns an RNA integrity number (RIN) for RNA from frozen tissues, or a DV200 for RNA extracted from FFPE specimens. All extracted DNA was subjected to a custom Sequenom single-nucleotide polymorphism (SNP) panel or an AmpFISTR Identifiler (Applied Biosystems) to verify that all specimens representing a case were derived from the same patient.

### Genome sequencing

#### Data processing

The Genomics Data Commons (GDC) DNA-seq alignment pipeline^59^, was used to map WGS reads to a customized version of GRCh38. In brief, reads were aligned using BWA-MEM (v.0.7.15)^60^, followed by sorting, merging and duplicate-marking using Picard Tools (v2.26.10). Base quality scores were recalibrated using GATK (v.3.7.0)^58^ and BQSR. Sample contamination levels were estimated using GATK ContEst, and samples with contamination levels exceeding 4% were excluded from further analyses.

#### SNV and indel calling

*The New York Genome Center pipeline.* The New York Genome Center (NYGC; v.6) somatic SNV–indel-calling pipeline^61^ was run for each tumour–normal and model–normal pair, starting from the GDC-aligned BAM files. In brief, SNVs, multi-nucleotide variants (MNVs) and indels were called using MuTect2 (GATK v.4.0.5.1)^62^, Strelka2 (v.2.9.3)^63^ and Lancet (v.1.0.7)^64^. Indels were also called using SvABA (v.0.2.12)^65^. Candidate indels predicted by Manta (v.1.4.0)^66^ were used as input to Strelka2, as per developer recommendations. Variants were merged across callers and annotated using Ensembl (v.93)^67^, COSMIC (v.86)^47^, 1000Genomes (Phase3)^68^, ClinVar (201706)^69^, PolyPhen (v.2.2.2)^70^, SIFT (v.5.2.2)^71^, FATHMM (v.2.1)^72^, gnomAD (r.2.0.1)^73^ and dbSNP (v.150)^74^, using Variant Effect Predictor (v.93.2)^75^. From the final callset of SNVs and indels, we filtered those that met the following criteria: occurred in two or more individuals in a panel of normal samples^61^; those that had a minor allele frequency (MAF) of at least 1% in 1000Genomes or in gnomAD; had a tumour VAF less than 0.0001; had a normal VAF greater than 0.2; had depth less than 2 in either the tumour or the normal sample; or a VAF in the normal sample greater than in the tumour sample. The aforementioned panel of normal samples was constructed from 242 unrelated individuals, of which 148 were sequenced using HiSeqX in the Illumina Polaris project^76^, 73 were sequenced using HiSeqX at the NYGC, 11 were sequenced using NovaSeq at the NYGC, and 10 were sequenced on both HiSeqX and NovaSeq platforms at NYGC.

*Broad pipeline (whole-exome sequencing)*.Whole-exome sequencing (WES) BAM files were processed as described in the section ‘Data processing’. Variant calling was performed as previously described^77^. SNVs were called using MuTect and small indels were called using Strelka. Tumour-in-normal contamination estimation was performed using deTiN, and cross-participant contamination was estimated using ContEst. WES variant calling was performed as previously described^77^. A similar pipeline was used for processing variant calls from WGS.

*WashU pipeline*. The somatic variant pipeline TinDaisy2 (v.2.6.2; https://github.com/ding-lab/TinDaisy) was built around the common workflow language and rooted in the methods of an earlier GenomeVIP pipeline architecture^78^. For both WGS and WES data, we used TinDaisy2 for calling both SNV and indel variants. In brief, aligned BAMs for tumour and normal samples were processed using VarScan (v.2.3.8)^79^, Strelka2 (v.2.9.10)^63^, Pindel^80^ and MuTect (v.mutect-1.1.7)^81^. Filtering was performed to retain calls with length < 100, normal VAF ≤ 0.02, tumour VAF ≥ 0.05, tumour read depth > 14 and normal read depth > 8. Only variants identified by at least two or more callers were retained. Following annotation using VEP 99 (ref. ^82^), calls with a population allele frequency of <0.005 were retained, and those that are in dbSnP but not in COSMIC^47^ or ClinVar^83^ were excluded. Adjacent SNP variants were merged into double nucleotide polymorphisms and higher-order variants, and the resulting VCF output was normalized with bcftools (v.1.10.2)^84^.

*Consensus method*. The workflow MergeParticipantVcfs was run for all participants in the cohort. First, single-pair VCFs and MAFs from the NYGC (v.6) somatic pipeline, the Broad somatic pipeline and the WashU somatic pipeline were prepared for merging with the MergeVcf workflow. Multi-allelic calls were split, MNVs were labelled and then split into SNVs, centre names were prepended to all annotations and indels were left-aligned and normalized. Prepared VCFs were merged using BCFTools^84^. During the merge–normalization of variants, the merging of SNPs into indels was not allowed. We calculated allele depth, read depth and allele frequency from BAM pileups using a custom method as described previously desribed^61^. MNVs were re-established. If a pipeline reported only a subset of the SNVs that constitute an MNV, then the SNVs were also reported as a possible variant.

The merged, pair-level VCFs were merged again in the same fashion as described above using the MergeParticipantVcfs workflow. Next, we ran the MergeParticipantVcfs workflow, MuTect2 (GATK v.4.0.5.1)^62^ on the multisample VCF, in forcecalling mode. A MuTect2 filter with read orientation metrics was run on the force-called results. The filtered MuTect2 calls were used to annotate the merged centre VCF. The final genotype was taken from the MuTect2 forcecalls and all other MuTect2 annotations were included with the prefix “Mutect2Multi_.”

The non-normal aliquots that the variant was called in, and the list of callers supporting the variant, were annotated in the VCF. Variants were also annotated using Ensembl variant effect predictor (v.97)^67^ as well as the databases COSMIC (v.98)^47^, 1000Genomes (Phase3)^68^, ClinVar (09012020)^69^, Polyphen2 (v.2.2.2)^70^, SIFT (v.5.2.2)^71^, FATHMM (v.2.1)^72^, gnomAD (Genomes (v.3.1.2) and Exomes (v.2.1.1))^73^ and dbSNP (v.150)^74^.

The annotated calls were used as input for the MakePairHighConfidenceVcfs workflow and filtered into final HighConfidence VCFs. Calls were removed if MuTect2 failed the call. Calls were added for a pair if they had read support in all non-normal WGS BAMs for that participant (even if the variant was not formally called for this pair).

#### Copy number calling

*Ascat method*. Purity and ploidy estimates were generated for each tumour–normal and model–normal pair using AscatNGS (v.4.2.1)^85^ using default parameters.

*ABSOLUTE method*. Copy number segmentation was performed using fragcounter and ReCapSeg, and subsequently with AllelicCapSeg. AllelicCapSeg output files were used as inputs for ABSOLUTE, with WES (when available) or WGS mutation calls used to infer allelic integer copy number profiles. All ABSOLUTE calls were manually curated to identify the ABSOLUTE with integer copy number states best aligning with the observed copy number values. In samples with no CNAs, the optimal ABSOLUTE solution was inferred from SNV multiplicity.

*ReMixT method*. We applied ReMixT^86^ to predict allele-specific and clone-specific copy numbers from WGS samples according to previously described methods^87^.

*PURPLE method*. For each tumour–normal or model–normal sample pair, we generated a B-allele frequency of heterozygous SNP sites using AMBER (v.3.5)^88^, and we determined read depth ratios using COBALT (v.1.11)^88^. We integrated this information, together with somatic SNVs and structural variants (SVs) to estimate the purity, ploidy and copy number profile of the tumour using PURPLE (v.2.54)^88^. Tumour and model samples that did not have a matched normal sample were processed in a similar manner, but with several version changes: AMBER (v.3.9), COBALT (v.1.13) and PURPLE (v.3.4). AMBER, COBALT and PURPLE were developed by the Hartwig Medical Foundation and are freely available on GitHub (https://github.com/hartwigmedical/hmftools).

*HATCHet method*. For each tumour–normal or model–normal sample pair, we ran HATCHet-2 (ref. ^89^) and estimated purity and ploidy. We used default parameters, but with the following exceptions. The minimum and maximum number of states and state transition probability in the hidden Markov model were set to 20, 40 and 10^−12^, respectively, whereas ‘diploidbaf’ and ‘maxneutralshift’ parameters were both set to 0.06. The search space for the number of clones was between two and four. A Gurobi commercial optimizer was used as the preferred engine for solving integer linear programming.

*Consensus method*. For each sample, the ABSOLUTE solution for which values were nearest to the consensus purity and ploidy values was selected as the consensus copy number solution. In cases when the optimal ABSOLUTE purity and ploidy solution and the consensus purity and ploidy solutions were divergent, the consensus solution was still selected. ABSOLUTE forcecalling was performed using each selected purity and ploidy solution with the output being the segmented allelic copy number state at each genomic locus.

#### SV calling

*NYGC pipeline*. The NYGC (v.6) somatic SV-calling pipeline^61^ was run for each tumour–normal and model–normal pair, starting from the GDC-aligned BAM files. In brief, SV breakpoints were called using SvABA (v.0.2.12)^65^, Manta (v.1.4.0)^66^ and Lumpy (v.0.2.13)^90^. We excluded SVs below 500 bp, merged the rest across callers using bedtools (v.2.26.0)^91^, pair-to-pair with the slop parameter set to 300 bp, and requiring the same strand orientation and at least 50% reciprocal overlap. SVs were annotated using 1000Genomes (Phase3)^68^, DGV^92^, gnomAD-SV^93^ and the SV panel of normals (built from the same individuals as the NYGC SNV–indel pipeline) with bedtools pair-to-pair, using the same parameters as for the merge across callers. We removed from the final callset any SVs that overlapped variants in 1000Genomes, DGV, gnomAD-SV or the panel of normals.

*Broad pipeline*. SVs were called using Manta^66^, SvABA^65^ and dRanger^94^. Breakpointer^94^ was used to refine breakpoint locations of called variants. The same SV had to have been called across at least two of Manta, SvABA and dRanger (using a clustering window of 350 bp to match the SV to the same event) to show up in the final callset. Certain filters were applied across various steps of the pipeline based on span, mapping quality and reads of support for SV events.

*WashU pipeline*. We used Manta (v.1.6.0)^66^ for calling SVs on matched tumour–normal WGS data. We then filtered results to retain variants that met the following criteria: (1) the sample site depth was less than 3× the median chromosome depth near one or both variant breakends; (2) the somatic score was greater than 30; and (3) for a small variant (<1,000 bases) in the normal sample, the fraction of reads with MAPQ0 around the breakend did not exceed 0.4. We then converted VCF files to BEDPE format with svtools (v.0.5.1)^95^ (https://github.com/hall-lab/svtools).

*MSKCC pipeline*. We identified SVs using deStruct (v.0.4.18)^96^ and LUMPY (v.0.2.12)^90^, retaining only the breakpoints called by both methods. We applied filtering based on the following criteria: inter-breakpoint distances of ≤30 bp; deletions smaller than 1,000 bp; breakpoints with fewer than 5 supporting reads in the tumour sample; or any read support in the matched normal sample.

*EMBL-EBI pipeline*. For tumour–normal and model–normal sample pairs, somatic SVs were called using GRIDSS2 (v.2.12.0)^97^, annotated with RepeatMasker (v.4.1.2)^98^, and kraken2 (v.2.1.2)^99^ and filtered with GRIPSS (v.1.9)^100^. The final somatic SV set was further refined and annotated with the copy number profile estimated using PURPLE (v.2.54)^88^. SVs and copy number profiles were visualized using the ReConPlot R package (v.1.0)^101^. Tumour and model samples without a matched normal sample were processed in a similar manner but with several version changes: GRIPSS (v.2.0.1) and PURPLE (v.3.4).

*Consensus method*. Consensus SVs from the NYGC Broad, WashU, MSKCC and EMBL-EBI were identified as previously described^102^ using bedtools pair2pair with minor modifications. The original consensus algorithm used single-caller VCFs as inputs, whereas the modified pipeline uses multicaller consensus inputs from each centre. Moreover, the original pipeline used a slop value of 400, whereas the modified pipeline uses a slop value of 50. SVs called by pipelines from two or more analysis centres were accepted into the final SV consensus.

#### Purity and ploidy

*ESTIMATE*. Tumour purity was computed according to the ESTIMATE algorithm^103^, which generates a composite ESTIMATE score that represents the quantification of predefined gene signatures of non-tumour components (stromal and immune) in gene expression data. Tumour purity was then inferred using the following formula: tumour purity = cos (0.6049872018 + 0.0001467884 × ESTIMATE score).

*Consensus purity and ploidy*. We computed consensus tumour and model purity and ploidy from the purity and ploidy values from five DNA-based copy number callers (Ascat, ABSOLUTE, ReMixT, PURPLE and Hatchet) and purity values from a sixth RNA-based caller (ESTIMATE). Consensus purity was computed using an iterative process wherein the mean purity across all callers was first computed. If the individual purity values across all six purity callers fell within ±0.2 of the mean purity, then the mean purity was accepted as the consensus purity. If one or more callers fell outside the window, then the furthest caller from the mean was removed and the mean ploidy was recomputed. This process was repeated until all remaining callers converged on a consensus purity value or when fewer than three callers remained. In the latter case, the purity value was accepted as the value from ABSOLUTE or, in cases when there was no ABSOLUTE value, from PURPLE.

Ploidy values for each individual caller were first binned into ploidy classes: haploid (ploidy < 1.5), diploid (1.5 ≤ ploidy < 2.5), triploid (2.5 ≤ ploidy < 3.5) or tetraploid (≥3.5). If values from three or more callers fell in the same ploidy class, then the median ploidy from all callers in that ploidy class was taken as the consensus ploidy. If there was no agreement between three or more callers, then the ploidy value from ABSOLUTE was accepted as the consensus ploidy value or, in cases when there was no ABSOLUTE value, the value from PURPLE was accepted as the consensus ploidy value. All consensus purity and ploidy values went through subsequent manual curation and the ABSOLUTE solution was selected in cases when the consensus purity and ploidy values poorly reflected the underlying copy number states.

#### Analysis methods

*Tumour–model SNV concordance*. SNVs included in concordance analysis were filtered for variants with VAF > 0.15 or variants for which all non-normal aliquots contained read support.

*Tumour–model LOH concordance*. The LOH status for all tumour–model pairs was determined by examining whether the rounded minor copy number of each genomic bin was equal to zero. LOH status was then compared between the tumour and its corresponding model, assigning a value of 1 for concordance and 0 for discordance. The mean LOH concordance was subsequently calculated across all equal-sized bins, which provided a single LOH concordance value for each tumour–model pair.

*WGD inference*. We determined WGD status by assessing genome-wide CNAs. First, we calculated the fraction of the genome that was affected by copy number gains, weighting each segment by its genomic length. We evaluated the extent of duplication based on thresholds for copy number amplification across the genome. Samples in which a majority of the genome exhibited copy number gains beyond a defined threshold were classified as having undergone a single WGD event, whereas those surpassing a higher threshold were assigned multiple WGD events.

*Mutation signature*. SNV mutational signatures were computed using SignatureAnalyzer (GPU version)^104,105^ with the COSMIC single-base substitution signature reference comprising 96 mutational signatures. During mutational signature factorization, a set of 51 non-redundant mutational signatures were selected. For each tumour and matched model, we generated per-sample mutational signature profiles represented as exposure vectors across these 51 signatures. Concordance between tumours and their matched models (*n* = 440 pairs) was quantified in terms of the cosine similarity between their respective signature profiles. As a negative control, we generated random tumour–model pairings of equal number, both in the same tumour type and across different tumour types, and calculated cosine similarities for the randomized pairs.

*Driver oncogene selection and classification*. To assess the differences in the prevalence of driver mutations between tumour and model samples, we first curated a set of driver genes based on previous TCGA studies^106^ (Supplementary Table 10). To determine the prevalence of SNVs, small indels and copy number variations (CNVs), we integrated the results from both SNV–indel and copy number analyses. Gene annotations for SNVs and indels were obtained through the discovery pipeline. Only mutations with a predicted impact of moderate or high, as defined in the Ensembl calculated gene consequences table, were included in downstream analyses. An additional mutation category included upstream promoter missense mutations in the *TERT* gene. Gene-level CNVs were annotated by calculating the mean copy number across the genomic span of each gene. Only genes with high-level amplifications or homozygous deletions were included in the downstream analysis. A gene was classified as highly amplified if it met the following criteria: (1) sample ploidy ≥ 1.5; (2) either the gene copy number was ≥3 and ≥2 times the ploidy (adjusted by –0.5 if the sample was a model); or (3) the gene copy number was ≥7. Conversely, a gene was classified as having a homozygous deletion if its copy number was below 0.3.

For the final visualization in Fig. 2, mutations were classified as either gain-of-function (GOF) or loss-of-function (LOF). For each gene, only mutations that were found in both tumour and matched model samples (that is, shared in a tumour-model pair) were considered. Only mutations shared between tumour and matched model samples (that is, present in both) were considered for classification. If no shared mutations were identified for a given gene, it was labelled as unclassified. Mutation types were grouped as follows: LOF included homozygous deletions, truncating variants (frameshift indels, nonsense and splice-site mutations); GOF included missense mutations, high-level amplifications, *TERT* promoter mutations and in-frame insertions or deletions. A gene was labelled LOF if more than 15% of its shared mutations were LOF events, otherwise, it was labelled as GOF.

*Power calculation*. We calculated a measure of the power to detect model SNVs in a given tumour by considering the probability that model SNVs would be detected in the tumour, assuming that there were clonal and at one allelic copy. The expected VAF for a single-allelic clonal tumour mutation at a genomic position with tumour copy number *T* is given by

$$\mathrm{VAF}=\frac{\rho }{\rho T+(1-\rho )N}$$

Here *N* is the copy number at the given position of the contaminating cells, which was assumed to be two for autosomes. Then, assuming a binomial distribution of read counts and one read necessary to detect, the average power to detect a clonal SNV was calculated by

$$\mathrm{Power}\,\mathrm{to}\,\mathrm{detect}=\frac{1}{N}{\sum }_{i}^{N}1-{\mathrm{Binom}}_{\mathrm{CDF}}({C}_{i},0,{\mathrm{VAF}}_{i})$$

where Binomial_CDF_ is the binomial cumulative density function. Here *C*_*i*_ is the read coverage in the tumour at the position of the *i*th model SNV. This value is calculated by summing over all *N* SNV positions identified as having at least one alternative read in the model and any number of reads in the tumour. Only autosomal SNVs were considered for the power calculation.

*ecDNA*. Raw coverage was calculated from the GDC-aligned BAM files using fragCounter (v.1.0; https://github.com/mskilab-org/fragCounter). These values were corrected using dryClean (v.1.0), a robust PCA-based method that separates the foreground from the background signal, which reduces noise and artefacts. It was run using a panel of 390 normal samples, which we built by selecting random normal samples across different datasets: ICGC DCC ESAD-UK^107^, TCGA^108^, the MSKCC–WCM–NYGC HRD project^109^, The Cancer Alliance at NYGC^110^, the Hartwig Medical Foundation (https://www.hartwigmedicalfoundation.nl/en/data/), ICGC PanCancer Analysis of Whole Genomes^7^, CCLE^2^ and other publications^111–116^. These corrected values were used as inputs for CBS^117^ to calculate the tumour–normal coverage ratio and to segment the genome into regions of similar copy number, thereby identifying potential amplifications and deletions. This information, together with the consensus purity and ploidy values and the consensus SVs, were used to construct junction-balanced genome graphs that had high-fidelity copy number profiles using JaBbA (v.1.1)^110,118^.

GDC-aligned BAM files and JaBbA-derived copy number profiles were then used as input to the AmpliconSuite-pipeline (v.0.5.2)^119^. This pipeline is a wrapper for the AmpliconArchitect (v.1.3.r5)^120^ and downstream AmpliconClassifier (v.0.5.3)^119^ tools. As per developer recommendations, samples derived from the same patient (for example, paired tumour–models) were run as a group using GroupedAnalysisAmpSuite.py, using default parameters.

We then compared the resulting amplicon calls using the comparison tool feature_similarity.py, with default parameters. ecDNAs were considered concordant between tumour and model if the overlapping amplicons were classified the same way and had a Jaccard genomic interval similarity of ≧0.75. A Jaccard genomic interval similarity was calculated as the total length of the intersection of the amplicon footprints, divided by length of the union. We retained amplicon calls if the indicated filter was ‘none’. ecDNA calls, filtered by AmpliconArchitect, were ‘rescued’ back into the callset if they had a passing concordant ecDNA in the associated tumour or model.

Putative cyclic ecDNA reconstructions were generated using the AmpliconSuite-pipeline module Candidate AMplicon Path EnumeratoR. In brief, the tool searches each amplicon graph for the longest cyclic and non-cyclic paths, choosing paths that best explain the observed copy numbers, and filtering based on how well the best reconstruction fits the data. Reconstructions that passed were plotted using CycleViz (https://github.com/AmpliconSuite/CycleViz).

*Clonal phylogenies*. Force-called SNVs were input into pyclone and phyclone to generate clonal phylogenies. We excluded indels from the analysis. We annotated each SNV with the major and minor allele copy number of the encompassing segment from consensus copy number calling. SNV copy number and supporting read counts were input to pyclone-vi (v.0.1.6)^121^, which we ran with a beta-binomial observation model, 10 restarts and a maximum of 40 clusters. We removed clusters comprising less than 1% of all SNVs and clusters that were approximately 0.5 cancer cell fraction across all samples (cancer cell fraction range of 0.3–0.7). The resulting pyclone clusters were input to phyclone (v.0.5.1)^122^, which we ran with a beta-binomial observation model, 16 chains, 100,000 iterations and outlier probability set to 0.1.

*Genetic ancestry estimation*. Ancestry proportion was determined using ADMIXTURE (v.1.3.0)^123,124^, which used a maximum likelihood-based method to estimate the proportion of reference-population ancestries in a sample. To do this, we genotyped reference markers that we generated from 1,964 unrelated 1000Genomes project samples directly on the whole-genome samples using GATK pileup (v.3.4.0). We excluded individuals from the populations MXL (Mexican ancestry from Los Angeles, United States), ACB (African Caribbean in Barbados) and ASW (African ancestry in the Southwest United States) from the reference owing to their being putatively admixed. We further filtered the reference by using only SNP markers with a minimum MAF of 0.01 overall and 0.05 in at least one 1000Genomes continental population. Variants were also pruned on the basis of linkage disequilibrium using PLINK (v.1.9) with a window size of 500 kb, a step size of 250 kb and an r2 threshold of 0.2. The analysis resulted in a proportional breakdown of each sample into five continental populations (AFR, AMR, EAS, EUR and SAS) and 23 populations. We then categorized patients by the continental population of highest proportion.

### DNA methylation and epigenetic fidelity

#### DNA methylation data

DNA methylation was evaluated using the Illumina HumanMethylationEPIC (EPICv1) array (Illumina). We downloaded raw IDAT files produced by the Illumina iScan system from the GDC data portal (https://portal.gdc.cancer.gov). We calculated DNA methylation levels (*β* values) from the IDAT files using the openSesame pipeline with the default arguments implemented in the R package SeSAMe (v.1.18.4)^125^.

#### Normal tissue methylation and probe selection

We used normal tissue DNA methylation data from external resources to investigate cancer-associated DNA methylation profiles. We had previously identified 146,385 CpGs that were unmethylated in eight normal tissue types (breast, adrenal gland, liver, lung, ovary, skin, blood and brain) on the EPICv1 array^126^. We processed additional ENCODE normal tissue DNA methylation data from 23 normal tissue samples from the gastrointestinal tract, including oesophagus (muscularis mucosa *n* = 4, squamous epithelium *n* = 4), stomach (*n* = 3), colon (transverse *n* = 4, sigmoid *n* = 4) and pancreas (*n* = 4). We downloaded the EPICv1 IDAT files from the ENCODE data portal^127^ and generated *β* values using the openSesame pipeline, as described above. We identified 159,361 CpGs that had a mean *β *value of <0.2 in any of the four gastrointestinal tissue types. Collectively, we selected 144,571 CpGs unmethylated in normal tissues from 12 tissue types to investigate cancer-associated DNA hypermethylation profiles (Supplementary Table 11).

#### TCGA–TARGET DNA methylation data

We analysed TCGA Pan-Cancer Atlas (PanCanAtlas) DNA methylation data profiled using the Infinium HumanMethylation450 (HM450) array. The raw IDAT files were obtained and processed using the R package SeSAMe. IDAT files from TARGET’s neuroblastoma and Wilms tumour projects^128,129^ were downloaded from the GDC data portal and processed using the R package SeSAMe. Our analysis included 130 Wilms tumours and 91 neuroblastomas originating in the adrenal glands.

#### Joint HCMI and TCGA–TARGET methylation data

We merged the HCMI and TCGA–TARGET DNA methylation data profiled using EPICv1 and HM450 arrays, respectively, to generate a dataset with the probes shared between the two platforms^125^. We excluded samples that had a CpG probe success rate of less than 90%. We also excluded probes with ‘NA’-masked data points that were present in more than 10% of the samples and probes on the X and Y chromosomes. To investigate the cancer-associated DNA hypermethylation profiles, we analysed the probe set that lacked tissue-specific DNA methylation (selected as described above) and then extracted 53,204 CpG sites that acquired methylation (*β* value of >0.3) in at least two samples in any HCMI cancer type (Supplementary Table 11).

#### UMAP of DNA methylation data

We performed dimension reduction using NMF on the combined HCMI and TCGA–TARGET DNA methylation data matrix described above. *β* Values of 0 were replaced with 1.0 × 10^−12^, and missing values were replaced with zeros. The resulting matrix was subjected to NMF, masking zeros, as implemented in the RcppML R package (v.0.5.6)^130^. We assessed the optimal rank for an NMF model by performing matrix decomposition across ranks ranging from 2 to 200, each with three random initializations, using the crossValidate function in the RcppML R package. We selected an NMF model of rank 170, as this model showed the minimum mean squared error of reconstruction consistently across the three runs. UMAP visualization of the rank-170 NMF model was generated using the umap function with the cosine distance metric implemented in the R package umap (v.0.2.10.0) (Supplementary Table 11). We produced the UMAP in Fig. 4c using the subset of the rank-170 NMF matrix, which included the cancer types represented in both the HCMI and the TCGA–TARGET projects.

#### Heatmap of DNA methylation profiles

For the four cancer cohorts on which we focused, and from the HCMI–TCGA merged DNA methylation data described above, we identified the top 10% of the most variably methylated CpGs separately in each cancer type (Fig. 4e). We selected the 8,614 CpGs that represented the union of the 4 variably methylated CpG sets. To minimize the influence of variable tumour purity levels on clustering results, we dichotomized the data, using a *β* value of ≥0.3 to define positive DNA methylation and <0.3 to define a lack of methylation. The sample distance matrix was computed using the Jaccard index, and then unsupervised hierarchical clustering was performed for each TMP cancer subtype. We generated the heatmap using the ComplexHeatmap R package (v.2.20.0)^131^.

#### Tumour–model hypermethylation similarity scores

We assessed DNA methylation-based similarity between a model and its parent tumour based on cancer-associated CpG hypermethylation profiles. We identified the 144,571 CpGs unmethylated in normal tissues as described above. For each HCMI cancer type, we further selected CpGs hypermethylated (*β* value of >0.3) in at least two samples. Then we compared the Pearson’s correlation coefficient (*r*) as a similarity score for the following samples: (1) models and their matched tumours; (2) models and unmatched tumours from the same cancer type; and (3) models and unmatched tumours from different cancer types. A *P *value of paired model–tumour relatedness was then determined by calculating the probability of observing an unpaired model–tumour distance equal to or smaller than the same model’s distance to all unrelated tumours that were not from the same tissue type. After FDR adjustment, model–tumour pairs with adjusted *P *≥ 0.1 were considered to be models that were non-concordant with their original tumours.

### Transcriptional fidelity and relatedness

#### RNA analyte processing and sequencing

*Quality assurance and QC of RNA analytes*. RNA from fresh-frozen models was sent for characterization, whereas for most tumours, samples of FFPE-derived RNA were sent. Fresh-frozen RNA analytes were assayed for RNA integrity, concentration and fragment size. Samples for total RNA-seq were quantified on a TapeStation system (Agilent) and RIN scores were calculated. Model-derived RNAs with RINs > 8.0 were considered high quality. For FFPE samples, we used DV200 and fragment size to evaluate sample quality. Although we targeted input concentrations greater than 100 ng µl^–1^, some FFPE samples were lower.

*Total RNA-seq library construction*. To construct total RNA-seq libraries, we used Illumina Stranded Total RNA Prep with RiboZero Gold, and we barcoded samples with individual tags, following the manufacturer’s instructions (Illumina). We prepared libraries, which we then pooled using an automated liquid-handling system to minimize variance. Typically, these were pools of 38–92 samples, depending on the available capacity on a sequencer. At every step we performed QC. We used a TapeStation system to quantify library concentrations, fragment size and distribution. As needed, pool balance and library quality were assessed using miSeq Nano single-end 50-bp sequencing.

*Total RNA-seq*. We prepared indexed libraries and ran them on an Illumina NovaSeq 6000, using paired-end 100 bp reads and generating a minimum of 150 million reads per sample library, with a target of greater than 90% mapped reads. In all but a few cases, all data were from the same sequencing run. For the few samples that needed additional read depth, we provided this with a secondary sequencing run. We demultiplexed raw Illumina sequence data and converted these to fastq files while quantifying adapter and low-quality sequences. Samples were assessed for information quality by mapping reads to the human hg38 genome reference, estimating the total number of reads that mapped, the fraction of RNA reads that mapped to coding regions, the amount of rRNA in a sample, the number of genes expressed and the relative expression of housekeeping genes. The samples that passed this quality assurance and QC step were then clustered with other expression data from similar and distinct tumour types to confirm expected expression patterns. We SNP-typed atypical samples to confirm the source analyte. FASTQ files of all reads were then uploaded to the GDC repository and distributed to the analysis teams.

*MicroRNA (miRNA)-seq library construction*. miRNA-seq library construction used a v4 NEXTflex Small RNA-seq kit (PerkinElmer), then samples were barcoded with individual tags following the manufacturer’s instructions. We prepared libraries on a Sciclone liquid-handling workstation. We performed QC at every step and quantified the libraries using a TapeStation system and an Agilent Bioanalyzer using a Small RNA Analysis kit. Pooled libraries were then size-selected according to specifications from a NEXTflex kit. Post-sequencing quality assurance and QC evaluated the abundance and diversity of miRNA. Data that passed were provided to the GDC.

#### Transcriptional relatedness measurements

*MOMA subtype identification*. To compare tumour subtypes in HCMI to those previously identified in TCGA, using the network-based MOMA algorithm^33^, we used the OncoMatch algorithm^38^ (see below). Specifically, we assessed the similarity of each HCMI sample to those in the subtypes identified by MOMA in TCGA. As these methods compare samples on the basis of the conservation of their master regulator proteins, which are highly enriched in mechanistic determinants of tumour cell state^37,132–134^, this approach effectively complements the Celligner and DNA-methylation analyses. Specifically, this analysis helps refine subtype classification by mitigating potential confounding effects in gene expression profiles—such as those related to tissue histology, unrelated to tumour biology—as well as effectively mitigating technical batch effects^33^. Master regulators, although rarely mutated, have crucial roles in cancer progression, as they orchestrate transcriptional networks that are disrupted by upstream genomic alterations, which makes them critical therapeutic targets^38,39^, including in clinical trials^135,136^. As such, they are more conserved in each tumour subtype than the corresponding transcriptional profiles^33,132^. In the following sections, we discuss the various algorithms used in this analysis.

*Original MOMA analysis*. In brief, MOMA is based on the assumption that transcriptional cell states are implemented and homeostatically maintained by small, autoregulated modules of master regulator proteins, comprising transcription factors (TFs) and co-factors (co-TFs). We assessed the activities of all TFs and co-TFs using the VIPER algorithm^137^, which is based on the expression of their transcriptional targets, which we inferred using the ARACNe algorithm^138^. Tumour subtypes (*n* = 112) were then identified by clustering samples based on TF–co-TF activity, using the ‘partitioning around medoids’ algorithm^139^. Below, we describe VIPER and ARACNe.

*MOMA subtype comparison*. To compare HCMI samples to those in the 112 MOMA subtypes in TCGA, we first used the metaVIPER algorithm^140^ to compute the activity of all TFs and co-TFs. MetaVIPER—a multinetwork version of the original VIPER algorithm^137^ that integrates the protein activities assessed by each network—enabled the use of networks from multiple TCGA cohorts that were matched to the histology of HCMI samples (Extended Data Table 1). Enrichment of the top 50 most differentially activated genes in a HCMI sample (that is, 25 most active and 25 most inactive) in TFs–co-TFs that were differentially expressed in each TCGA MOMA subtype were used to assess their similarity (with the OncoMatch algorithm). Indeed, we have shown that across all TCGA cohorts, >80% of the functional mutations in each sample are in pathways upstream of the top 50 master regulators. Moreover, we have shown that changing the number of master regulators between 20 and 200 does not significantly affect the OncoMatch statistics^33^.

*MetaVIPER analysis*. For the analyses in this paper, we used the most recent version of the metaVIPER algorithm^140^. The main difference is that we replaced the original method for performing gene set analysis (aREA) by nonparametric analytical-rank-based enrichment analysis (NaRnEA)^141^, which improved the assessment of significance for differentially active proteins. For implementing NaRnEA, we used the matrix_narnea() function in the PISCES R package^142^. For each sample, TF–co-TF transcriptional targets (that is, regulatory networks) were inferred by ARACNe analysis of one or more lineage-matched TCGA cohorts. In brief, metaVIPER estimates the differential activity of each regulatory protein by integrating the NES statistics produced by VIPER analysis using each of the selected networks. VIPER assesses the activity of a protein by assessing the NES of its activated and repressed targets in genes that were differentially expressed in a signature of interest.

*Differential expression signature generation*. To remove batch effects between TCGA and HCMI samples, TPM-normalized gene expression profiles were first processed using a variational autoencoder (VAE) model called scGEN^143^. For subtype stratification, for which the goal is to assess differentially active proteins in samples in the same cohort, differential expression signatures for VIPER analysis were optimally computed by comparing each sample to the centroid of the entire cohort. We accomplished this by subtracting the median expression across all samples and then dividing by the median absolute deviation (MAD). To prevent division by zero, MAD values <0.01 were set to 0.01. For both HCMI and TCGA, we downloaded the TPM-normalized gene expression for protein-coding transcripts from the GDC portal^144^. The cancer-specific networks used in the analysis were inferred by ARACNe as described below.

*Regulatory network inference*. Regulatory networks for each HCMI cohort were generated by analysing the gene expression profiles in their lineage-matched TCGA cohorts using ARACNe3 (ref. ^141^). ARACNe3 is the latest incarnation of the ARACNe algorithm^138^. The algorithm identifies regulatory protein–target interactions on the basis of the greatest conservation of the transferred information, as assessed by computing the mutual information on the direct path and on every indirect path traversing an intermediary TF–co-TF protein, based on the data-processing inequality^145^.

To generate cancer-type-specific networks, we applied ARACNe3 to TPM-normalized expression profiles across 32 TCGA cohorts (ACC, BLCA, BRCA, CESC, CHOL, COAD, DLBC, ESCA, GBM, HNSC, KICH, KIRC, KIRP, LGG, LIHC, LUAD, LUSC, MESO, OV, PAAD, PCPG, PRAD, READ, SARC, SKCM, STAD, TGCT, THCA, THYM, UCEC, UCS and UVM). Each ARACNe3 network was inferred by subsampling the gene expression profiles until ≥50 targets were identified for each TF–co-TF in the consensus network. The analysis included 1,645 TFs and 1,556 co-TFs, retrieved from ref. ^146^ and ref. ^147^, respectively. Networks were pruned to the 100 most significant targets for each regulatory protein, based on consensus mutual information statistics. For TCGA–ESCA, we inferred networks for adenocarcinoma and squamous cell carcinoma, separately.

*OncoMatch analysis*. OncoMatch^38^ was used to assess the similarity between HCMI and TCGA samples based on a weighted enrichment analysis of their differential protein activities. First, we computed the differential protein activities for each of *n* = 112 MOMA subtypes, as assessed by analysis of 20 cohorts with sufficient size for the analysis, including BLCA, BRCA, COAD, GBM, HNSC, KIRC, LAML, LGG, LIHC, LUAD, LUSC, OV, PAAD, PRAD, READ, SARC, SKCM, STAD, THCA and UCEC. Specifically, for each subtype, we generated an average protein activity (consensus protein activity signature) by integrating its VIPER-inferred NES values across each sample in the subtype, using Stouffer’s *z* score method. Then, we generated a *P *value to assess the similarity of each HCMI sample to each MOMA subtype in its lineage-matched cohorts. This was accomplished by assessing the following criteria: (1) the enrichment of the top 50 most differentially active protein (top and bottom 25) in the HCMI sample in a protein differentially active in the consensus protein activity signature of each MOMA subtype using NaRnEA; (2) the enrichment of the top 50 most differentially active protein (top and bottom 25) in the consensus protein activity signature of each MOMA subtype in a protein differentially active in the HCMI sample; and (3) by integrating the two using Stouffer’s method.

HCMI samples may show high similarity to more than one subtype. As such, a final assignment was performed as follows. As the tumour and the model samples were derived from the same tumour mass, we assumed that their subtype assignment should be conserved. Thus, among all MOMA subtypes producing a significant match to a model and its parental tumour *P* < 0.05, one-tailed, Benjamini–Hochberg-adjusted, we selected the subtype with their best integrated Stouffer score. If no agreement was identified (that is, no MOMA subtype with significant OncoMatch score for both the model and its parental tumour), then the two were independently assigned to their best scoring MOMA subtype. For simplicity, we mapped HCMI colorectal, oesophageal–gastric and GBM samples only to COAD, STAD and GBM MOMA subtypes in TCGA, respectively, thus excluding subtypes from READ, ESCA and LGG. A null model for the OncoMatch analysis was generated by generating a probability density of OncoMatch NES scores matching each HCMI sample to all non-lineage-related TCGA subtypes. A one-tail *P *value was assessed, as the only relevant result would be for the HCMI OncoMatch score to be larger than the OncoMatch from the null hypothesis.

*Tumour–model similarity analysis*. The transcriptional state similarity between models and their parental tumours was also assessed based on OncoMatch statistics. Differential expression signatures for VIPER analysis were computed by further normalizing and scaling the TPM-normalized gene expression data by subtracting the median expression across all same type samples (that is, tumours or models) in the same HCMI cohort and dividing by the MAD. Again, to prevent division by zero, MAD values <0.01 were set to 0.01. Differential gene expression was assessed separately for tumours and models to avoid sample type related batch effects. For small cohorts (*n* ≤ 10 samples), we used the median and the MAD of lineage-matched samples that they belonged to. Specifically, the following cohorts were normalized together: (1) intrahepatic, extrahepatic cholangiocarcinoma and hepatocellular carcinoma; (2) all sarcoma types, including bone cancer and desmoid tumours; and (3) tubulovillous adenoma, rare gastrointestinal cancers and colorectal cancer. Similar to the above analysis, we inferred protein activity using metaVIPER with the NaRnEA enrichment analysis algorithm and cancer-type-specific networks. For instance, some HCMI cohorts (for example, COAD) could be matched to multiple lineage networks inferred from TCGA cohorts (that is, COAD and READ). Thus, we assessed protein activities for colorectal, lung, bile duct–liver, brain and gastroesophageal samples in HCMI using COAD–READ, LUAD–LUSC, LIHC–CHOL, GBM–LGG and ESCA–STAD networks, respectively. For rare cohorts that lacked sufficient samples to perform ARACNe analyses, (that is, Wilms tumour, small intestine cancer, gallbladder cancer and unknown carcinoma), we leveraged the ability of metaVIPER to automatically integrate across multiple networks. Specifically, we selected the three networks that produced the greatest differential activity for the top 50 proteins. The rationale is that incorrect networks can only decrease but not increase activity (that is, the more unrelated the network, the smaller the differential activity). To avoid diluting the results based on subpar networks, networks that produced significantly lower differential activity for each protein were excluded from the analysis. We also normalized samples in rare cohorts together with the samples identified as having the best matching networks.

To generate a conservative, nonparametric null-model, we computed the probability density function (PDF) of the NES generated by matching each model with all the non-lineage-matched tumours in HCMI. To improve the PDF estimate, which was highly non-Gaussian, we bootstrapped null-model generation 1,000 times using 60% of the non-lineage-matched samples and used the resulting PDF to convert matched tumour–model pair values to *z* scores. As both positive and negative NES were integrated, we used Stouffer’s method to generate integrated *z* scores. Tumour–model pairs with FDR > 0.1 were considered poor matches.

*Celligner*. To align our HCMI collection to publicly available collections of tumours (TCGA and TARGET) and models (CCLE), we used Celligner^31^, a computational framework to integrate and compare multiple gene expression datasets. Initially, we attempted to expand the tumour and model datasets by directly merging the transcriptional profile of the HCMI collection. However, this approach resulted in poor alignment of the data (Supplementary Fig. 10a). To address this, we expanded on the established Celligner approach by aligning the HCMI dataset to the existing reference datasets in a two-step process. First, we replicated the alignment of TCGA and TARGET tumours onto the CCLE cell lines to create a reference tumour–model map, as described in the original Celligner publication^31^ . Subsequently, we introduced the HCMI dataset into this integrated space.

In brief, using contrastive PCA, we identified gene expression signatures elevated in the TCGA–TARGET tumour collection compared with the CCLE models, consistent with the results reported in the original Celligner analysis (Supplementary Fig. 10b). These signatures were enriched for pathways related to immune and non-malignant features, including stromal cell enrichment (Supplementary Fig. 10b). To mitigate the influence of these non-malignant features, we removed the contrastive principal components (cPCs) associated with these signatures before proceeding to the next steps. We then applied mutual nearest neighbours (MNN) batch correction as part of the Celligner algorithm to align the datasets.

Using this aligned gene expression space as a reference, we integrated the HCMI models and tumours. Specifically, we removed the cPCs increased in the HCMI dataset, which were similarly enriched for immune-related gene signatures (Supplementary Fig. 10c), correlated to DNA-based tumour impurity assessment (Supplementary Fig. 10d) and then we applied MNN batch correction. For this integration, we removed the first three cPCs, which maximized the number of MNN pairs (Supplementary Fig. 10e) and aligned well with the expected variability in the dataset through gene-set enrichment analysis of gene expression profiles. We used a *k*_1_ value of 20 and *k*_2_ value of 50 for MNN, as recommended by the Celligner developers, to reflect differences in dataset size and composition. The results suggested that the HCMI tumour collection exhibited similar levels of variability and contamination as the reference TCGA–TARGET dataset.

After integrating all datasets, we performed PCA on the combined data and calculated pairwise Euclidean distances between all tumours and models in the 70-PC space. These distances formed the basis for all Celligner distance-based analyses that we described in this study. To visualize the integrated data, we created a 2D UMAP embedding based on the 70 PCs for visualization (Supplementary Table 11).

To measure transcriptional relatedness between matched HCMI model–tumour pairs, we used the Celligner distances, which represent the Euclidean distance between a model and its paired tumour in 70-PC space. A *P* value of paired model–tumour relatedness was then determined by calculating the probability that an unpaired model–tumour distance equal to or smaller than the same model’s distance to all unrelated tumours that were not from the same tissue type. After FDR adjustment, model–tumour pairs with adjusted *P *values equal to or greater than 0.1 were considered to be models that were non-concordant with their original tumours. Relatedness of tumour lineage is as indicated in Supplementary Table 12.

*Expression data input for Celligner*. TARGET samples (*n* = 784) and TCGA expression data (*n* = 9,806) were obtained from the Xena browser (https://xenabrowser.net). Cell line gene expression data for 1,377 samples were taken from the DepMap Public 19Q4 file. HCMI dataset expression data were downloaded from the GDC portal, and TPM unstranded data were used for only protein-coding genes. Gene expression data were then log_2_ transformed after adding a pseudocount of 1. Finally, we subset gene expression data to the 18,550 protein-coding genes that were present in all datasets for all Celligner analyses.

*Euclidean and latent TF (OHSU methods) preprocessing*. The unique genetic and molecular distribution call for each cancer cohort (for example, pancreatic cancer) and specimen type (that is, tumour or derived model) for these groupings suggested that each be run independently through the following pipeline. We filtered RNA gene expression data for biologically relevant features (the combined set of feature-selected genes of top methods from TCGA trained algorithms)^32^. For instances where it was needed for analysis, we aggregated multiple TCGA cohorts to more closely match the specific cancer types included in HCMI cohorts (that is, the HCMI lung cancer cohort included TCGA LUAD and LUSC cohorts; the HCMI STAD–ESCC cohort included TCGA GEA and ESCC cohorts; the HCMI kidney cohort included KIRP, KIRC and KICH TCGA cohorts). Any remaining cohorts with low sample size (*n* < 8) were statistically underpowered and excluded from downstream analysis.

*Euclidean distance calculation*. Euclidean-based distances were measured in a group (for example, in pancreatic tumours). First, we computed the mean pairwise gene Euclidean distances. For example, we generated a list of pairwise gene distances by calculating all gene distances between pairs of samples. We reported the sample pair distance as the mean of this list. We then repeated the process for all sample pairs, reporting sample pairs for both tumour–tumour pairs for intracohort similarity and tumour–model matched pairs for derived model similarity. We report the *z* score of these distances and identified outliers as (>3 *z* score).

Latent TF distance calculation. Latent TF distances were generated using neural networks. Specifically, a variational autoencoder model (NetVae) was trained with gene expression of normal tissues from all TCGA cohorts and genotype–tissue expression (GTEx). These datasets were aligned using quantile ranking. On a gene-wise basis, we calculated the summed difference between a HCMI sample (tumour or model) and normal tissue. These deviation scores were then correlated with mutations and encoded into latent space. The same methods described in the section ‘Euclidean distance calculation’ were applied to these latent values to generate the latent TF distances. Both intracohort and intercohort distances were calculated.

*Multiclass pair classification-based distance calculation*. We observed that relative gene expression was effective in removing batch effects across different datasets. When we plotted TCGA data using the relative expression of the most variable 500 genes on UMAP space and mapped HCMI samples to the plot, we found that data were clustered on the basis of tissue type. This was in contrast to continuous gene expression data, which clustered data in cohorts. Given this, we used a multiclass pair classification-based approach to compute the distances between matched tumour–model pairs. The multiclass pair classification algorithm^148^ uses the relative expression between gene pairs as input and is an extension to multiple classes of the *k*-top-scoring pairs algorithm^149^ that was developed for binary classification. We regressed out the purity effects in both TMP and HCMI data and found that removing purity effects did not make subtypes indistinguishable. We applied the best model selected over cross validations in TMP data to HCMI models and tumours and computed the pairwise Euclidean distances between the assignment probabilities of each HCMI sample to TMP cohort subtypes. We labelled a matched tumour–model distance as an outlier if it was above Q3 + 1.5 × IQR of the tumour–model distances in the TMP cohort.

*Canonical parallel direction*. We transformed and normalized the combined gene expression matrix using the variance-stabilizing transform from DESeq2 (ref. ^150^) then split the matrix into separate tumour and model matrices. We calculated the canonical parallel direction (CPD) by taking the difference between tumour–model pairs and then performing PCA on the resulting difference matrix with the R package irlba (https://github.com/bwlewis/irlba). We took the CPD as the first loading vector. We derived distances by projecting individual expression matrices onto the CPD to obtain a score, and we took the difference in scores between model–tumour pairs as the CPD distance. We labelled samples as outliers when their distance was greater than two standard deviations from the mean.

#### HCMI and CCLE model coverage comparison

A critical rationale for model generation is to provide proxies for in vitro or in vivo studies of tumour biology. As a result, it is critical to assess what fraction of the tumours in a large-scale repository (for example, TCGA) are associated with effective proxies in large model repositories such as CCLE and HCMI. For this purpose, we assessed the fraction of TCGA tumours that had at least one high-fidelity matched model in CCLE and HCMI. Specifically, we identified high-fidelity models as those producing an OncoMatch score of NES ≥ 10, as previously discussed^38,39^.

For this purpose, we generated violin plots representing the probability density of the highest OncoMatch NES scores against each model in HCMI, CCLE and the joint repository of HCMI and CCLE models. As in the other analyses, batch-effect-corrected, TPM-normalized datasets were mitigated using the scGEN algorithm, without considering cancer type and tumour–model covariates. Then, TPM-normalized CCLE gene expression profiles were obtained from DepMap 21Q3 (refs. ^9,151^). Gene expression profiles were then centred by subtracting the median expression across all samples and dividing by the MAD. As discussed in previous sections, we used MetaVIPER to assess the activity of TF and co-TF proteins, using regulatory networks generated by ARACNe analysis of the TCGA cohort from which each tumour sample was selected.

### Tumour molecular pathology subtype predictions

We predicted the subtype of each sample using TMP models^32^. These models were trained on molecular data (gene expression, CNV, DNA methylation, miRNA expression and somatic mutations) from TCGA primary tumours. We included a library of models that used either one data type or multiple data types. We applied models that used only gene expression, or DNA methylation, to HCMI samples because of their top performance with TCGA data. We quantile-ranked gene expression data before application. For each sample, we report a single subtype by considering all models and their model confidence values (for example, we report the consensus subtype of the five independent models that were run for ovarian samples).

### snRNA-seq analysis

#### snRNA-seq sample preparation and analysis

Frozen samples (*n* = 34, from 17 tumour–model pairs) were obtained from the BPC Nationwide Children’s Hospital, DFCI and ATCC. Nucleus isolation was performed using a Chromium Nuclei Isolation kit with RNase inhibitor (10x Genomics, 1000494). Samples were homogenized using a pestle in lysis buffer, passed through a column and centrifuged in debris-removal buffer to eliminate residual tissue and debris. The isolated nuclei were then washed, resuspended and loaded onto a 10x Chromium platform for gel bead-in-emulsion (GEM) generation and barcoding. Following GEM reverse transcription, samples underwent post-GEM RT cleanup and cDNA amplification. After quality control and quantification of cDNA, libraries were prepared using the Chromium Single Cell 3′ Gene Expression Library Construction protocol and sequenced on a NovaSeq platform.

We aligned snRNA-seq short-read data from 17 tumour–model pairs (34 samples) multiplexed across 16 runs, generated by the Columbia University Single Cell Analysis Core, including 7 GBM, 6 PAAD and 4 COAD pairs, to the prebuilt 10x Genomics human reference genome GRCh38-2024-A, using 10x Genomics Cell Ranger software (v.8.0.1)^152^. Illumina base call files were converted to FASTQ files with the command cellranger mkfastq. Expression data were processed with cellranger count on the pre-built human reference, which encompassed 38,606 features. Cell Ranger performsd default filtering for QC, and generates filtered feature–barcode matrix files (filtered_feature_bc_matrix.h5; barcodes.tsv, genes.tsv, and matrix.mts) containing unique molecular identifier (UMI) counts for genes for each run.

#### snRNA-seq demultiplexing

To recover sample-specific profiles for samples processed in the same 10x Chromium flow cell, we demultiplexed the Cell Ranger output^152^ using Demuxlet^153^. As Demuxlet requires a list of sample-specific SNPs as an input, we called germline variants for each human sample from the matched normal WGS data. To limit the number of false-positive doublets, which are common in snRNA-seq demultiplexing owing to background RNA and potential large duplications or deletions shared by tumours in the same cohort, we used the gnomADv4 exome data to subset only the most likely germline variants. Specifically, we retained only biallelic SNPs that met the following criteria: (1) population MAF ≥ 0.1%; (2) identified in the gnomADv4 WES cohort^154^; and (3) flagged as PASS in gnomAD. We ran Demuxlet with the following parameters: --field GT and --group-list barcodes.txt, where ‘barcodes.txt’ was the cell barcode file generated by Cell Ranger. As previously recommended^155^ for demultiplexing snRNA-seq samples, we assigned cells to a sample and used these cells in downstream analyses only if the BEST column of the Demuxlet-generated <Cell Ranger run >.best file started with ‘SNG-’ or the BEST column started with ‘DBL-’ and the PRB.DBL column contained a value of ≤0.99. We classified droplet barcodes with ‘DBL-’ in the BEST column and >0.99 in the PRB.DBL column as doublets, whereas those labelled with AMB were unassigned; droplets labelled as doublet or unassigned were excluded from downstream analyses.

#### snRNA-seq gene expression analysis

UMI matrices for each demultiplexed sample were processed using Scanpy (v.1.9.3)^156^. For QC, we only retained cells with <25% mitochondrial RNA content and UMI counts in the range (800; 50,000). To recover more cells in tumour samples with low cell counts (that is, cases HCM-BROD-0110-C25 and HCM-CSHL-0073-C25), we increased the mitochondrial RNA content threshold to 35% and removed the lower bound on UMI counts. To recover more cells from a run that included three COAD tumours, we removed the threshold on mitochondrial RNA content while maintaining an upper UMI count limit of 100,000. Owing to the low number of cells in the tumour from case HCM-CSHL-0247-C18, we excluded this tumour–model pair from downstream analyses. As a result, we analysed 16 tumour–model pairs: 7 for GBM, 6 for PAAD and 3 for COAD. For statistics on UMI counts before and after QC for individual samples, refer to Supplementary Table 8. UMI counts were normalized, log transformed and scaled, following the standard Scanpy preprocessing workflow. PCA was performed with the ‘arpack’ solver (‘scanpy.tl.pca’). The nearest-neighbour distance matrix was computed with ‘scanpy.pp.neighbors’, setting ‘n_neighbors’ = 15 (default). UMAP embeddings were computed using ‘scanpy.tl.umap’, and PCA and UMAP visualizations were generated with the ‘scanpy.pl.pca’ and ‘scanpy.pl.umap’ functions. For analyses involving dimensionality reduction and visualization of tumour–model pairs or involving multiple cases, the same single-sample workflow was applied by re-scaling the multi-sample concatenated data. Individual samples were clustered using the resolution-optimized Leiden clustering algorithm via the acdc-py wrapper for the Scanpy’s Leiden implementation (https://pypi.org/project/acdc-py/). We determined the optimal number of clusters by varying resolution values from 0.01 to 1.01 in 0.01 increments, selecting the solution that produced the highest average Silhouette score.

#### Copy number and putative malignant cells detection

We inferred CNAs from expression counts at the single-nucleus level using the inferCNV package^157^. We clustered nuclei according to their unsupervised clustering labels, based on gene expression. A reference set of 2,193 nuclei (1,840 oligodendrocytes and 352 microglia) were sampled from four GBM tumours (HCM-BROD-0199-C71, HCM-BROD-0415-C71, HCM-BROD-0012-C71 and HCM-BROD-0002-C71). We used these reference nuclei as controls to infer CNAs in GBM samples. We used a reference set of 3,592 nuclei (2,112 fibroblasts and 1,480 stellate cells) sampled from 6 PAAD tumours (HCM-CSHL-0078-C25 primary and metastatic, HCM-CSHL-0089-C25 primary and metastatic, HCM-CSHL-0073-C25 and HCM-BROD-0110-C25) to infer CNAs in each PAAD sample. We labelled cell types in the references (oligodendrocytes, microglia, fibroblasts and stellate cells) via over-representation analysis (ORA) using the Python decoupler package with canonical human markers (‘brain’ and ‘immune system’ for GBMs; ‘pancreas’, ‘immune system’ and ‘connective tissue’ for PAAD) from the PanglaoDB database^158,159^. We sampled a reference set of 435 nuclei (annotated as macrophages by SingleR) sampled from HCM-BROD-0001-C18, and we used the only COAD sample that had enough non-epithelial cells to infer CNAs in each COAD sample. The following parameters were used in each inferCNV run: cutoff=0.1, window_length = 101, HMM=TRUE, mode=’i6’, analysis_mode = ‘subclusters’, denoise=TRUE. To classify putative malignant cells, we compared the inferred CNAs in each snRNA-seq sample to the CNAs detected by WGS in matched bulk samples. Cells were labelled as malignant if they belonged to a cluster with those CNAs.

#### Cell-type calling for GBM samples

snRNA-seq profiles from GBM samples first underwent coarse-grain cell-type assignment via ORA, using the decoupler Python package with canonical human markers for the brain from the PanglaoDB database^158,159^. To address the low signal-to-noise ratio inherent in snRNA-seq data, we performed soft gene imputation by summing the UMI counts of the ten nearest neighbours of each nucleus before running ORA. We labelled nuclei not labelled as ‘oligodendrocytes’ or ‘microglia’ and belonging to clusters of putative malignant cells from inferCNV analysis as ‘malignant’ and assigned these cells to specific cellular states, as described below.

#### Malignant state assignment for GBM samples

For each GBM nucleus labelled as malignant, a gene expression signature was computed as follows: ${z}_{i}^{(k)}=\frac{{x}_{i}^{(k)}-{\overline{x}}_{\mathrm{ref}}}{{\sigma }_{\mathrm{ref}}}$, where ${x}_{i}^{(k)}$ is the *d*-dimensional vector of ln(*x* + 1)-transformed gene expression for nucleus *i* in snRNA-seq sample *k* (with *d* equal to the number of genes), and ${\overline{x}}_{\mathrm{ref}}$ and ${\sigma }_{\mathrm{ref}}$ are the *d*-dimensional vectors representing the mean and standard deviation of ln(*x* + 1)-transformed gene expression across all malignant GBM snRNA-seq profiles, respectively. We performed gene set enrichment analysis of each gene expression signature across the six subtypes—NPC1-like, NPC2-like, MES1-like, MES2-like AC-like and OPC-like—as previously identified^40^, using the NaRnEA algorithm^141^. For this purpose, we used the Python package ‘pyVIPER’^160^. Each gene set comprised 39–50 genes that represented distinct cellular states. We then assigned each nucleus to the subtype that produced the maximum NaRnEA NES. Nuclei with the smallest *P* value (>0.15) were assigned no cellular state and were labelled as unknown. For simplicity, we combined the NPC1-like and NPC2-like subtypes into a single NPC-like subtype; similarly, we merged the MES1-like and MES-2 like subtypes into a single MES-like subtype.

#### Cell-type calling for PAAD and COAD samples

For each PAAD and COAD snRNA-seq profile, an initial coarse-grain cell-type assignment was performed using the Python implementation of SingleR using the Blueprint-ENCODE reference^161,162^. SingleR computes the correlation between each individual nucleus and every sample in the reference. It then labels each nucleus with the cell type with the highest average correlation. To deal with the low signal-to-noise ratio characteristic of snRNA-seq data, before to running SingleR, we applied soft gene imputation using a metacell approach. That is, by adding the UMI counts of the ten nearest neighbours of each nucleus using scanpy.pp.neighbors (Euclidean distance). Nuclei with an ‘epithelial cell’ annotation, which were also classified as putative malignant cells on the basis of inferCNV analysis, were labelled as malignant and assigned to specific transcriptional states, as detailed below. In the COAD cohort, we also retained a small subset of putative malignant cells annotated as ‘neurons’ by SingleR, as these were likely to be malignant cells with neuroendocrine features.

#### Malignant state assignment for PAAD samples

For nuclei derived from PAAD samples, we used a subtype classification strategy similar to that for GBM. We tested three classification strategies proposed in the literature, including those proposed by ref. ^163^, ref. ^34^ ref. ^43^; the first was from bulk profiles and the other were two from single-cell profiles. For the first two, we used the same methodology described for the GBM samples. For the latter, we performed the comparison at the protein activity level. Specifically, protein activity in malignant cells was assessed using metaVIPER^140^ by integrating six PAAD gene regulatory networks, which we generated independently from distinct PAAD cohorts, including single-cell profiles (scNET)^43^, laser microdissected samples (CUMC-net)^164^, the TCGA PAAD cohort (TCGA-net)^165^, the ICGC PAAD cohort (ICGC-net)^166^, the UNC cohort (UNC-net)^163^ and single-cell profiles from HCMI samples (HCMI-net). These networks captured regulatory interactions for TFs, co-TFs, signalling proteins and surface markers. For the first five networks, detailed information for the generation has been previously provided^43^. The HCMI-net was generated from snRNA-seq profiles from malignant nuclei in the HCMI PAAD cohort using ARACNe3 (ref. ^141^), with metacell generation constructed using the ‘pyviper.pp.repr_metacells’ function as input (the metacell approach adaptively aims at a target median depth of 10,000 UMIs per metacell whenever feasible). Although this target was not always met, a minimum depth of 8,432 UMIs was reached, which ensured robust representation of the gene expression profiles. To avoid bias associated with different regulon size, all regulons were pruned to the 100 most significant targets based on mutual information analysis. We then classified each single cell on the basis of three main PAAD transcriptional lineages identified as previously described^43^, including GLS, MOS and PLS. Classification was based on the significance of the NaRnEA-based NES representing the enrichment of the top 100 most differentially active proteins in each single nucleus (50 most activated and 50 most inactivated proteins) differentially active in the GLS, MOS and PLS signatures. Each nucleus was assigned to the cell state with the highest NaRnEA-inferred NES. Nuclei in which the highest-scoring subtype had a *P *> 0.15 were assigned to no specific transcriptional state and were labelled as ‘unknown’.

#### Malignant state assignment for COAD samples

For each nucleus in the COAD cohort, we computed a gene expression signature using the same methodology as applied to GBM and PAAD samples. Enrichment of each signature for two previously reported intrinsic subtypes, iCMS2 and iCMS3 (ref. ^167^), was calculated using NaRnEA based on the 50 most overexpressed and 50 most underexpressed genes in each signature. Each nucleus was assigned to the cellular state corresponding to the highest NES, whereas nuclei with a *P*  >0.15 value were labelled as unknown. The vast majority of cells in HCM-CSHL-0143-C20 and HCM-CSHL-0322-C20 were classified as iCMS2, the predominant epithelial state, which is enriched in tumours with strong WNT and MYC signalling activation^167,168^. We identified a subset of iCMS3 cells, typically in tumours with substantial immune activation and metabolic dysregulation^167,168^, in the HCM-BROD-0001-C18 tumour–model pair (about 50% in the tumour, around 20% in the model; Extended Data Fig. 8b,c). Although the proportions of iCMS2 and iCMS3 cells differed between the tumour and model, the analysis demonstrated that the model effectively recapitulated both cell states that were observed in the parental tumour.

#### Tumour–model differential gene expression

Differentially expressed genes between tumour and matched model samples were called using the Scanpy function ‘scanpy.tl.rank_genes_groups’ after ln(*x *+ 1) transformation. Statistics were assessed using the Wilcoxon rank-sum test followed by Benjamini–Hochberg correction for multiple-hypothesis testing. We used the resulting signatures to assess pathway enrichment with the ‘pyviper.tl.path_enr’ function in pyVIPER^160^. The following gene sets were used: MSigDB Hallmark gene sets for PAAD and COAD^169^; MSigDB Hallmark gene sets^169^; single-cell GBM expression meta-modules^40^; and bulk-based transcriptional signatures^170^ for GBM cases.

### Medium-switching assay

To assess transcriptional plasticity driven by culture media, HCM-BROD-0416-C71 GBM cells, originally maintained in NSA medium, were transitioned to formulated-conditioned medium. After initial recovery and expansion in NSA medium, cells were plated on laminin-coated culture vessels and, following 24 h of adhesion, switched to formulated-conditioned medium for either 72 h or 2 weeks. Cells were then fixed and stained for the indicated markers and imaged using the Operetta CLS High-Content Analysis system (Revvity). Full experimental details are provided in the Supplementary Methods.

### In vitro drug-sensitivity testing

Patient-derived GBM cells were maintained in NSA medium supplemented with epidermal growth factor and fibroblast growth factor under standard culture conditions^28,51^. For drug-sensitivity assays, cells were seeded at a density of 2,000 cells per well in ultra-low-attachment 96-well plates. Twenty-four hours after seeding, temozolomide was dispensed using a D300e digital dispenser in an 8-point titration series (0.03–300 µM). Cell viability was assessed after 5 days using a CellTiter-Glo luminescent assay (Promega).

### The HCMI Explorer Suite

To facilitate the exploration of HCMI translational and clinical utility, we developed the HCMI Explorer Suite, a web-based application built using R Shiny (v.1.8.1.1)^171^. The application was developed in the R programming language (v.4.3.2) and leverages key Shiny packages such as shiny, shinydashboard and shinythemes to provide a dynamic and interactive graphical user interface. The core modules of the HCMI Explorer Suite enable users to explore treatment timelines, model-specific genomic data and transcriptional similarities between cancer models, paired tumours and reference datasets (TCGA and CCLE). All plots were generated using ggplot2 (v3.3.5), with interactive features integrated using plotly (v4.9.3). The Clinical Module uses the swimplot package^172^ to visualize patient treatment timelines and to track model treatment exposures. The application is deployed via Shiny Server, with all backend processing and analyses performed server-side. The source code for the app is available at GitHub (https://github.com/human-cancer-model-initiative/HCMI-Explorer-Suite), and the hosted application can be accessed online (https://appshare.cancer.gov/HCMI_Explorer_Suite/).

### Ethics statement

All human tissue samples and associated clinical data used in this study were collected as part of the HCMI under protocols approved by the Institutional Review Boards (IRBs) of the respective Cancer Model Development Centres (CMDCs) and participating clinical sites. All procedures were conducted in accordance with relevant ethical guidelines and regulations. Written informed consent was obtained from all participants before sample collection, in accordance with HCMI programme requirements and institutional policies. Each participating centre fulfilled all regulatory requirements, including IRB-approved protocols, informed consent procedures and data-sharing agreements (Supplementary Note 2).

### Reporting summary

Further information on research design is available in the Nature Portfolio Reporting Summary linked to this article.

## Online content

Any methods, additional references, Nature Portfolio reporting summaries, source data, extended data, supplementary information, acknowledgements, peer review information; details of author contributions and competing interests; and statements of data and code availability are available at https://doi.org/10.1038/s41586-026-10806-y.

## Supplementary information

Supplementary Information This file contains Supplementary Notes 1–3, Supplementary Figs. 1–13, a guide to Supplementary Tables 1–15 (tables provided separately), Supplementary Materials and Methods and Supplementary References.

Reporting Summary

Supplementary Tables Supplementary Tables 1–15 (see Supplementary Information for descriptions).

Peer Review File

## Figures and Tables

**Extended Data Table 1:**
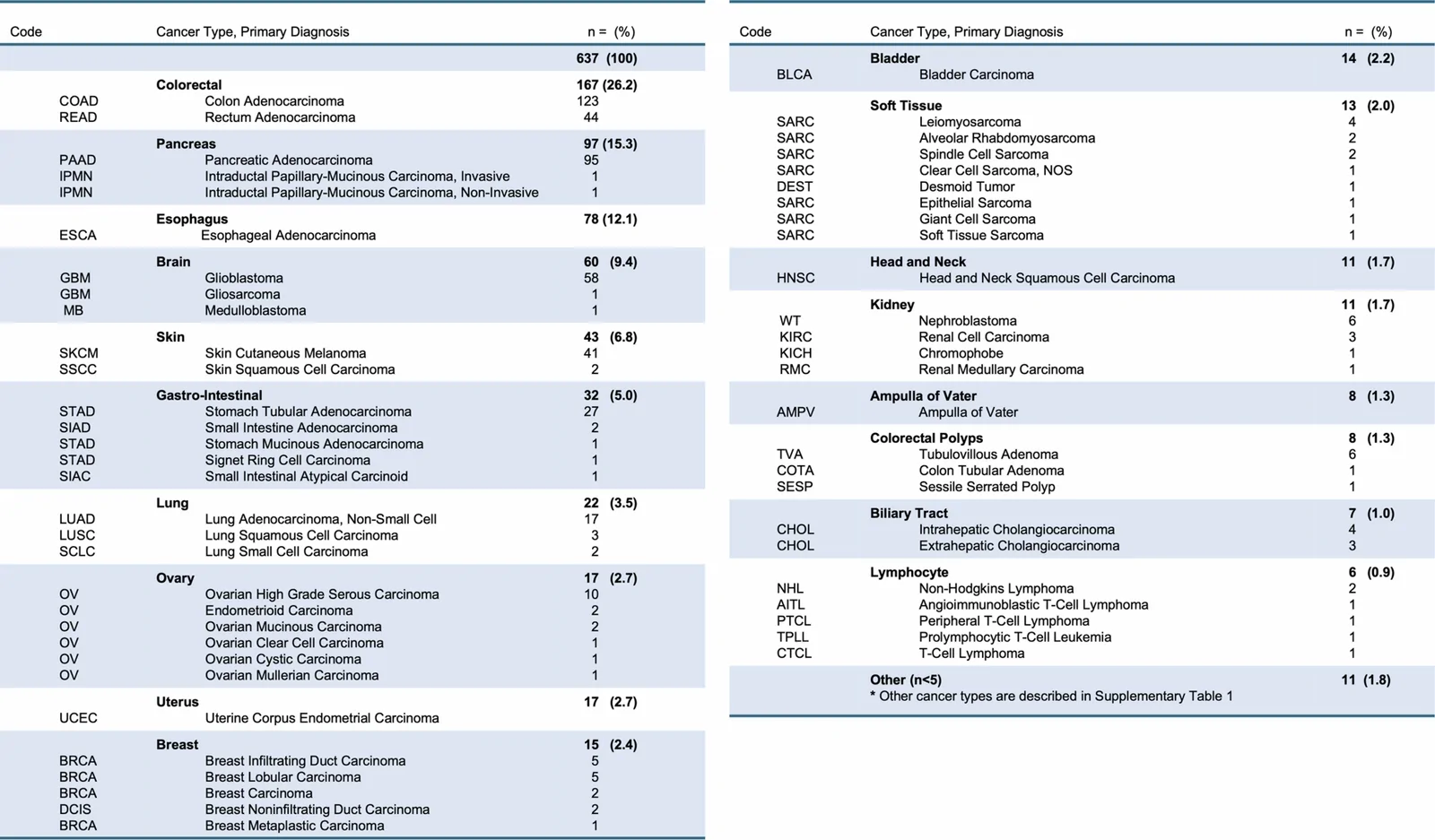
HCMI cancer types and primary diagnoses

**Extended Data Table 2:**
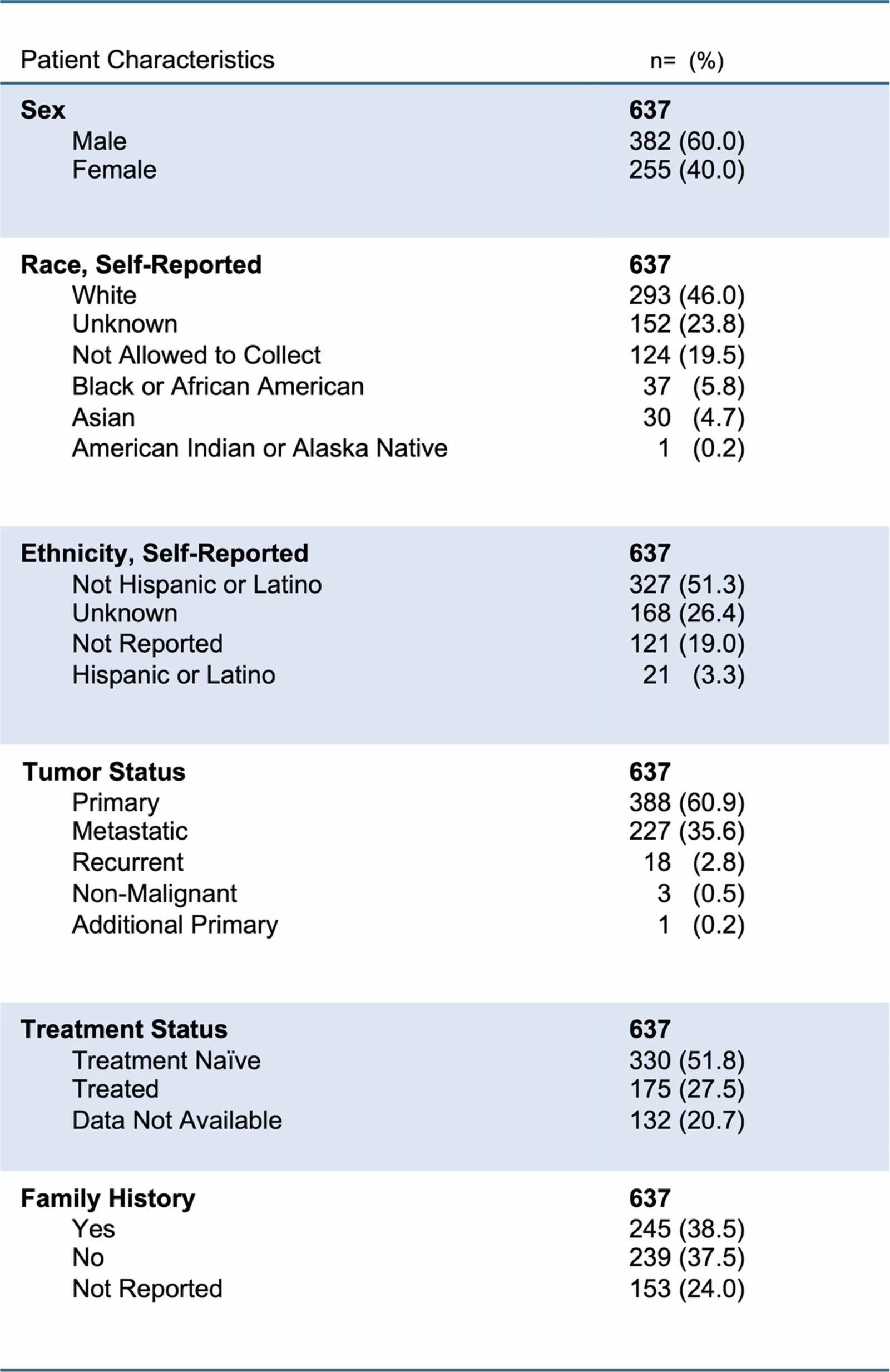
HCMI patient demographics and characteristics

## Data Availability

To support data access, we maintain a publicly accessible instruction portal (HCMI page at https://www.cancer.gov/ccg/research/functional-genomics/hcmi/using-hcmi), which serves as the central entry point for the resource. Molecular and genomic characterization data from this study are available through the HCMI–CMDC project on the NCI GDC web page (https://portal.gdc.cancer.gov/projects/HCMI-CMDC) and its accompanying GDC publication page (https://gdc.cancer.gov/about-data/publications/HCMI-CMDC-2026). Controlled-access raw sequencing data are available through dbGaP under study accession phs001486. Processed molecular datasets (mutation calls, copy number profiles and clinical annotations) are available through cBioPortal (https://www.cbioportal.org/study/summary?id=pancan_hcmi_2025). Interactive model-level exploration is available through the HCMI searchable catalogue (https://hcmi-searchable-catalog.nci.nih.gov).

## Code availability

All computational workflows, analysis pipelines and scripts used in this study are consolidated in a dedicated GitHub repository (https://github.com/NCICCGPO/HCMI-Flagship-Manuscript).
