# Supplementary material for: A compendium of next-generation patient-derived models for diverse cancers
Source: Nature. 2026 Aug 5;657(8132):775–88. doi: 10.1038/s41586-026-10806-y (PMC13581597; doi:10.1038/s41586-026-10806-y)
Supplement: Supplementary file 1 [file 41586_2026_10806_MOESM1_ESM.pdf]

nature portfolio

https://doi.org/10.1038/s41586-026-10806-y

Supplementary information

A compendium of next-generation patient-derived models for diverse cancers

In the format provided by the authors and unedited

Supplementary Information

A Compendium of Next-Generation Patient-Derived Models for Diverse Cancers

---

Supplementary Notes

Supplementary Note 1:

The Human Cancer Models Initiative (HCMI) is a large-scale international effort focused on expanding the diversity of available preclinical cancer models. The initiative has generated 665 models, including organoids, neurospheres, and cell lines, with comprehensive genomic, transcriptomic, and epigenomic characterization. As a complementary resource, the Sanger Institute, working collaboratively with hospitals across the United Kingdom derived 256 tumor organoids with a deep focus on 5 cancer types (colorectal, esophageal, ovarian, pancreatic and stomach) of which 133 were contributed to the HCMI (these 133 are amongst the 665 reported herein). The Broad Institute Dependency Map project aggregated 314 organoid models donated by collaborators inclusive of 156 HCMI models (these 156 are amongst the 665 reported herein) (**Supplementary Fig. 13**).

The Dependency Map project at the Broad Institute conducted CRISPR dependency screens on 149 models, 77 of which overlap with HCMI and 11 with Sanger. Additionally, the Sanger Institute CRISPR screened 98 models, 89 of which are part of the HCMI collection, to map cancer vulnerabilities across next-generation models. These findings are reported in the companion manuscripts by Neiswender *et al*. (Broad) and Herranz-Orset *et al*. (Sanger).

Overall, the three manuscripts describe the power of patient-derived models and the use of these models to map vulnerabilities and discover therapeutic targets.

Supplementary Note 2:

The HCMI Programmatic Workflow

Section 1: Administrative prerequisites to enrollment & tissue collection

During the initial phase of HCMI, for commencement of subject enrollment, each Cancer Model Development Center (CMDC) completed all required human subject and regulatory compliance documents for the HCMI.

These included:

- An IRB-approved Informed Consent that addressed all HCMI-specific requirements (outlined in the template at https://www.cancer.gov/ccg/research/functional-genomics/hcmi/using-hcmi#informed-consent-template-for-hcmi)
- A signed Institutional Certification Letter, allowing general research use of shared data
- Licensing agreements with intellectual property (IP) methodology owner(s), as needed
- Material Transfer Agreements (MTAs) and Data Use Agreements (DUAs), as required, to permit receipt and distribution of materials and data.

Section 2: Model Generation

At protocol initiation, with subject enrollment and the start of model development, the CMDCs deployed an internal ID linkage database that mapped between ID1, ID2 and ID3.

Step 1: An ID1 was assigned to consented donors by a CMDC, working in conjunction with the CMDC's clinical site partners. The ID1 was not shared outside the CMDC.
Step 2: Donor-matched normal and tumor material were obtained. A portion of the tumor was used for model generation. Sample aliquots for normal and remaining tumors were frozen and saved for shipment for molecular characterization.
Step 3: At the initiation of model generation, an ID2 was assigned by the CMDC. As model development progressed, the CMDC requested an ID3 from the Clinical Data Center (CDC).
Step 4: After assigning the ID3, the CDC registered the subject ID in dbGaP.
Step 5: The CMDC collected and submitted clinical enrollment data to the CDC. The CDC reviewed the clinical data submitted by the CMDC, and, if additional information was needed, requested it from the CMDC.
Step 6: The CMDC expanded the model until it obtained at least twelve vials of each model, with at least 1×10^6 cells per vial. The vials were frozen at the same time point so that the cells would have the same characteristics for all downstream applications.

Section 3: Initial Model QC

The initial quality control (QC) for models included validation performed by the CMDC. This initial QC involved confirming that the model was a cancer model.

Step 1: The CMDC confirmed via sequencing or other methods (either while the model was still growing, or after sufficient model material had been frozen), that the model consisted predominantly of cancer cells, and submitted QC confirmation data (e.g. MAFs from QC sequencing, images of slides, or other evidence that the model had been verified as cancer) to the Data Coordinating Center (DCC).

Section 4. Molecular Characterization of tumor/model/normal samples

Step 1: The CMDC and BPC (Biospecimen Processing Core) coordinated the shipment of tumor and normal material (including two vials of the normal), to the BPC. The CMDC also completed the required

documentation for the shipment of model vials to the distributor, ATCC (including sharing protocols), and coordinated with the distributor to ship ten vials of each model to the distributor.

Step 2: The BPC performed DNA and RNA extraction: DNA was isolated from tumor, normal, and model; RNA was isolated from tumor and model. The BPC performed genotyping to confirm that case samples matched and molecular QC of the DNA and RNA to verify the quantity and quality were adequate for sequencing by Genome Characterization Centers (GCCs). The BPC shipped DNA and RNA to the GCCs for characterization. The BPC also submitted biospecimen JSON files to the GDC.

Step 3: The GCCs performed sequencing of DNA and RNA received from BPC. The resulting primary data, BAM files (from whole exome sequencing [WXS] and whole genome sequencing [WGS]), and FASTQ files (from total RNA sequencing [RNA-seq]), were uploaded to the GDC.

Step 4: The GDC harmonized, analyzed, and integrated the sequencing and clinical data; and deposited the data files in a controlled-access location for review by the HCMI Analysis Working Group (AWG).

Step 4.1. The CMDC submitted follow-up clinical data (e.g. outcome data, treatment response) at least three months after donor enrollment.

Step 4.2. The CDC reviewed the follow-up clinical data and contacted the CMDC if additional information was needed. When QC was passed, CDC deposited the final clinical case information to the GDC. CDC informed the LBR, the CMDC and the BPC when the link between ID2 and ID3 was severed at the CDC.

Step 5: NCI and DCC preliminarily evaluated tumor and model mutation profiles, passing the data to the AWG. The AWG produced the analyses in this manuscript.

Supplementary Note 3:

When considering the WGS concordance between tumor and model samples, a few samples had low concordance at the SNV level. Two PDAC cases in particular stood out:

HCM-CSHL-1264 **(Supplementary Fig. 11)** and HCM-CSHL-0371 **(Supplementary Fig. 12)**

In both cases, the SNV-level concordance was less than 3%, the tumor purity was higher than 40% and the "potential driver mutations" (TP53, KRAS, etc) were different (identified both in WGS, RNA-Seq and WES). Intriguingly in both cases, the model sample harbored a KRAS G12 mutation (KRAS G12D at 48% VAF for HCM-CSHL-1264 and KRAS G12V at 90% VAF for HCM-CSHL-0371), but this mutation was absent in the tumor samples. Instead, another KRAS G12 mutation was present but undetected by the variant calling algorithms in the WGS pipeline (in both cases KRAS G12R at 10% and 12% VAF. The KRAS G12R mutation occurred at a different nucleotide position than the KRAS G12D or KRAS G12V mutations observed in the models).

Although clonal competition was initially thought to be the cause of this discordance, the two models were found to be established from tissue fragments geographically distant from those later submitted for sequencing for comparative analyses.

HCM-CSHL-1264 was labeled as a preinvasive neoplasm termed an Intraductal Papillary Mucinous Neoplasm (IPMN). The sampled tissue specimen presented spatially separated IPMNs classified as either low- or high-grade based on dysplasia of the lining epithelium, with the invasive component located nearby. The model was established from a tissue fragment containing a high-grade IPMN located near the invasive component. The tissue fragment subjected to sequencing was sampled from an adjacent tissue portion that contained a distinct invasive component. High-coverage targeted sequencing of the tissue fragment revealed that the invasive carcinoma harbored a KRAS G12R mutation, while the high-grade IPMN contained a G12D mutation. A nearby low-grade IPMN instead showed both G12V and G12D mutations, albeit at very low allele frequencies. The results support the concept that IPMNs present in a multi-focal manner as distinct clones, and therefore the invasive carcinoma may not reflect the genetic alterations present in the nearby IPMN.

HCM-CSHL-0371 was not labelled as IPMN, but similarly, the model has been established from a tissue fragment geographically distant from that sampled for sequencing analysis. Given the known polyclonal composition of preinvasive PDAC, these two cases reinforce the need to directly sequence a portion of the tumor material that is being used for model production.

Supplementary Figures

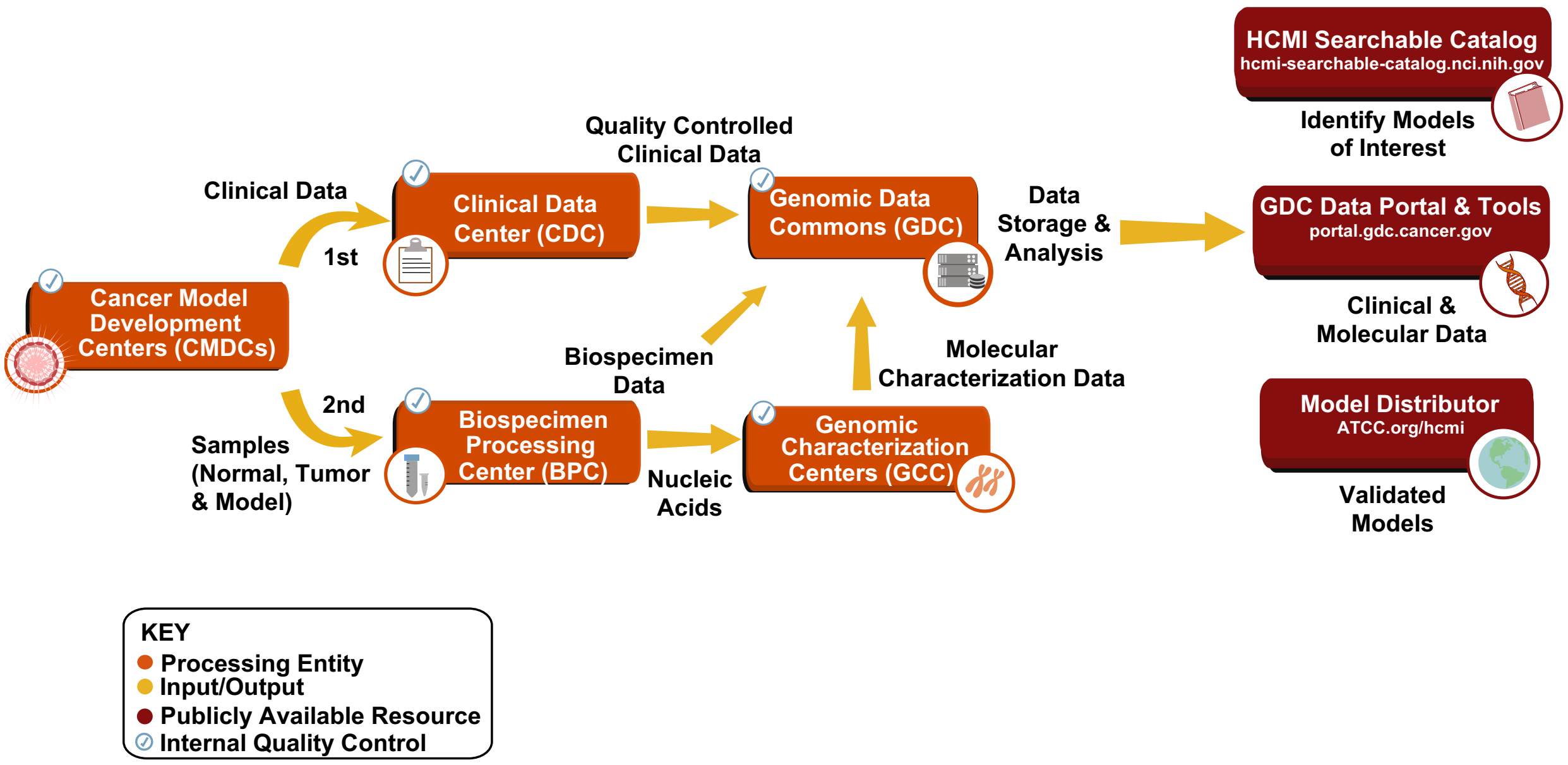

Supplementary Figure 1: **NCI HCMI cancer model development**

The models are first generated by the CMDCs. Clinical data associated with the model's case are submitted to the Clinical Data Center (CDC). The CDC quality-controlled clinical data are sent to the GDC. Case-matched normal tissue, parent tumor, and derived model are sent to the Biospecimen Processing Center (BPC) for isolation of nucleic acids. Nucleic acids passing QC are sent to the Genome Characterization Centers (GCCs) for 150x WXS, 15x WGS, and 120 million read RNA-seq, and raw sequencing data is submitted to the GDC for harmonization. DNA methylation arrays are completed on a subset of models and tumors. CMDC-validated models and culture protocols are available through the NCI-designated distributor, ATCC. The case-associated genomic, clinical, and biospecimen data are available at NCI's GDC. Figure originally published by the National Cancer Institute.

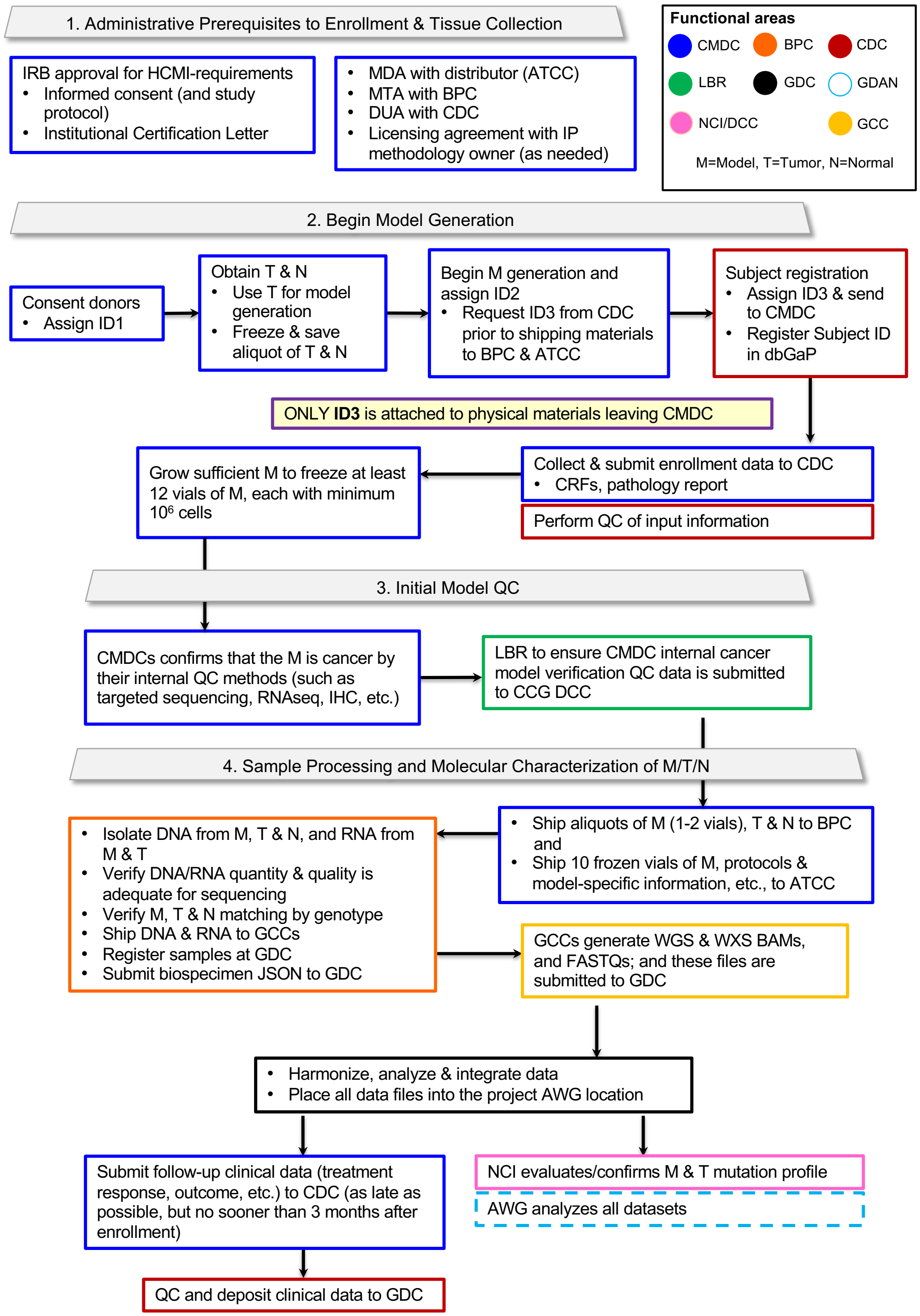

Supplementary Figure 2: Programmatic overview of cancer model pipeline
This schematic outlines the administrative, clinical, and molecular processes involved in model development within the HCMI. The workflow includes prerequisites for subject enrollment and tissue collection, model generation and quality control, and molecular characterization of tumor, model, and

normal samples. The figure highlights key steps, including informed consent approval, sample processing, ID assignment, sequencing, data deposition to the GDC, and final clinical follow-up as fully described in **Supplementary Note 2.**

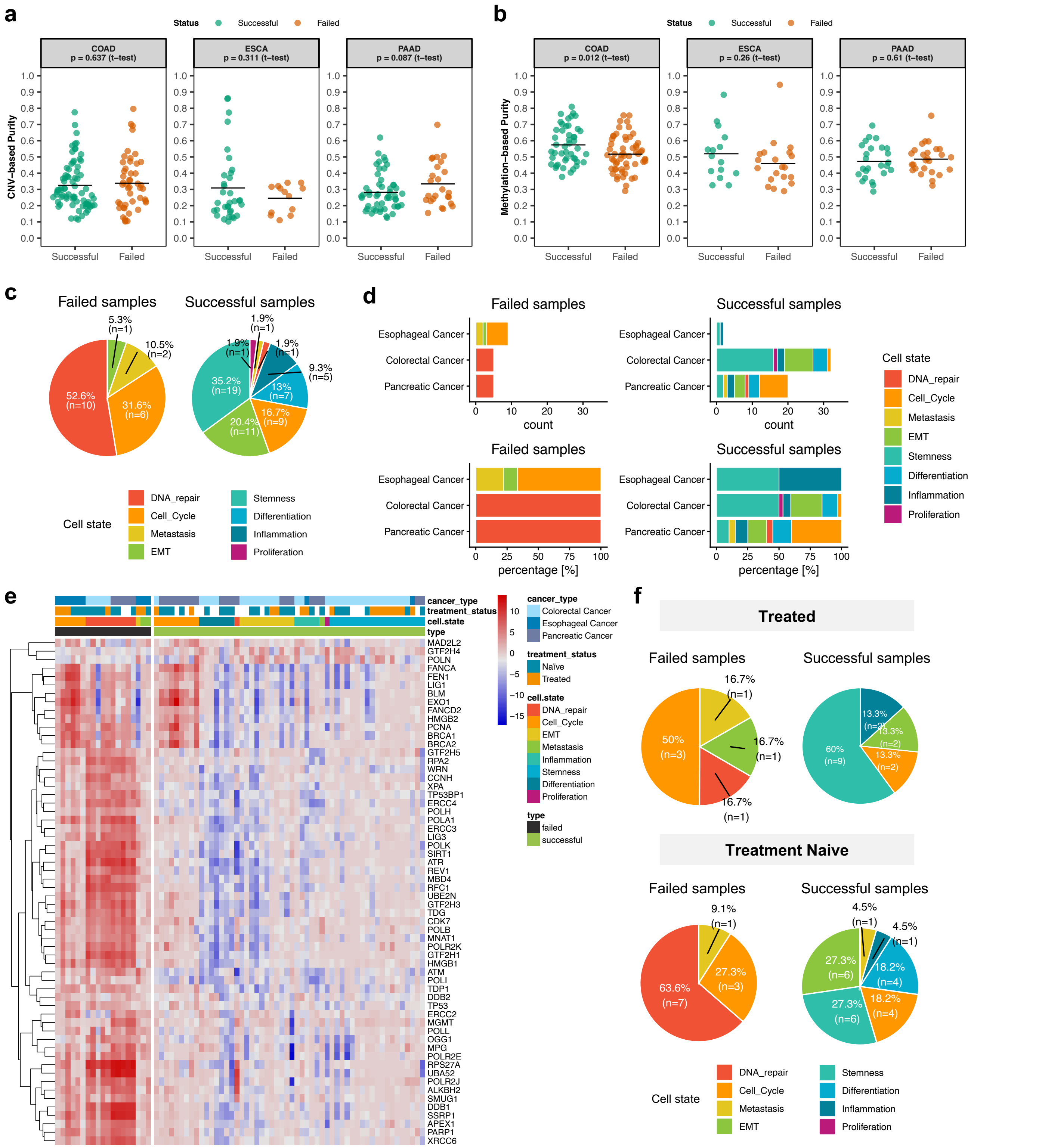

Supplementary Figure 3: Determinants of model establishment success. Comparison of tumor purity between successful and failed model derivations across colorectal (COAD), esophageal (ESCA), and pancreatic (PAAD) cancers. **(a)** CNV-based purity estimates and **(b)** Methylation-based purity. *P* values were calculated using two-sided *t*-tests. **(c)** Cancer cell states (CancerSEA[1]) identified by GSEA

among failed and successful tumors for colorectal, pancreatic, and esophageal cancers. The pie charts display the proportion (%) of each cancer cell state among failed (left) and successful tumors (right), respectively. **(d)** bar plots indicate the number of failed (left) and successful tumors (right) for each cancer type, with colors representing different cancer cell states. The bottom bar plots (left; failed tumors, right: successful tumors) show the percentage distribution of cell states for each cancer type. **(e)** Heatmap displaying master regulator activities of genes (rows) annotated for DNA repair across failed and successful tumor samples (columns). Positive and negative gene activities were colored red and blue, respectively. **(f)** Pie charts showing cancer cell states in failed and successful samples for treated (top) and treatment-naïve cases (bottom), respectively.

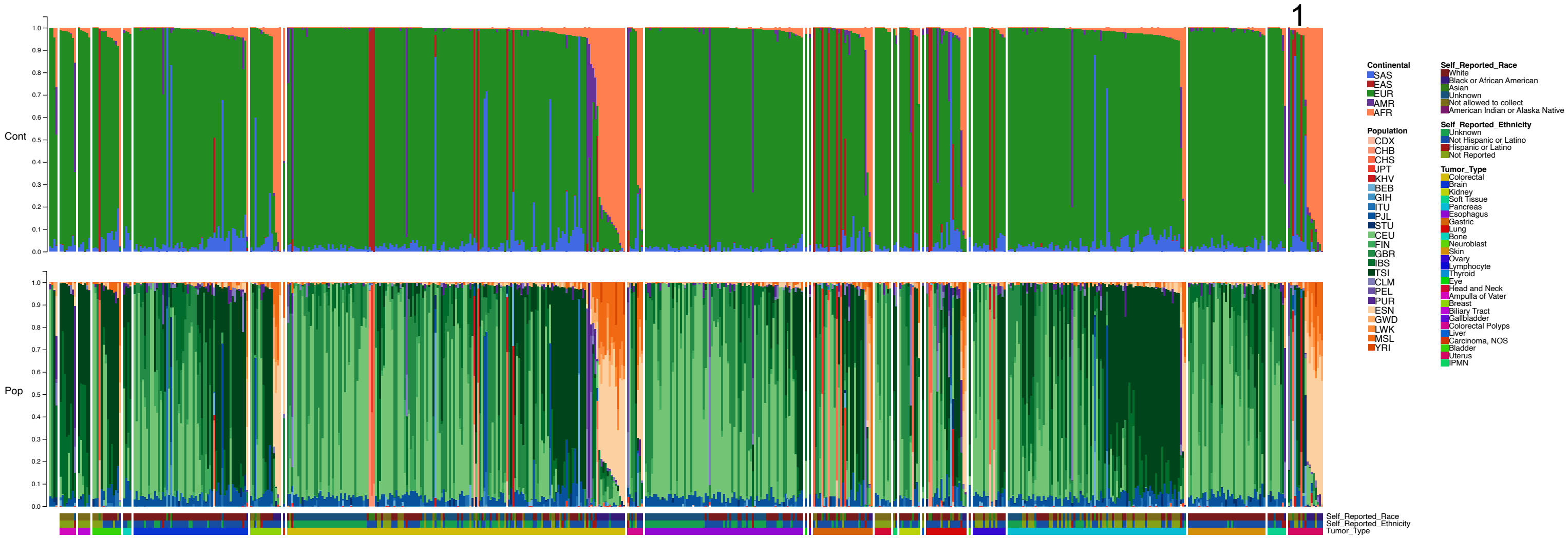

Supplementary Figure 4: Estimated genetic ancestry distribution in the HCMI cohort. Genetic ancestry was estimated from genotypes of the ancestry-informed markers obtained from our WGS alignments, in which we have continental-level ancestry estimations (upper) and population-level ancestry estimations (middle track) for each individual in our cohort. Patient self-reported race and ethnicity and associated tumor type are also shown for each patient (lower). Samples are grouped by tumor type and ordered by increasing AFR ancestry. Continental-level ancestry categories: AFR = African, EUR = European, EAS = East Asian, SAS = South Asian, AMR = Admixed American. Population-level abbreviations correspond to 1000 Genomes Project reference populations: CHB = Han Chinese in Beijing, CHS = Southern Han Chinese, JPT = Japanese in Tokyo, KHV = Kinh in Vietnam, BEB = Bengali in Bangladesh, GIH = Gujarati Indian in Houston, ITU = Indian Telugu in the UK, PJL = Punjabi in Lahore, STU = Sri Lankan Tamil in the UK, CEU = Utah Residents with Northern and Western European ancestry, FIN = Finnish in Finland, GBR = British in England and Scotland, IBS = Iberian populations in Spain, TSI = Toscani in Italy, CLM = Colombian in Medellín, PEL = Peruvian in Lima, PUR = Puerto Rican in Puerto Rico, ESN = Esan in Nigeria, GWD = Gambian in Western Division, LWK = Luhya in Kenya, MSL = Mende in Sierra Leone, YRI = Yoruba in Ibadan.

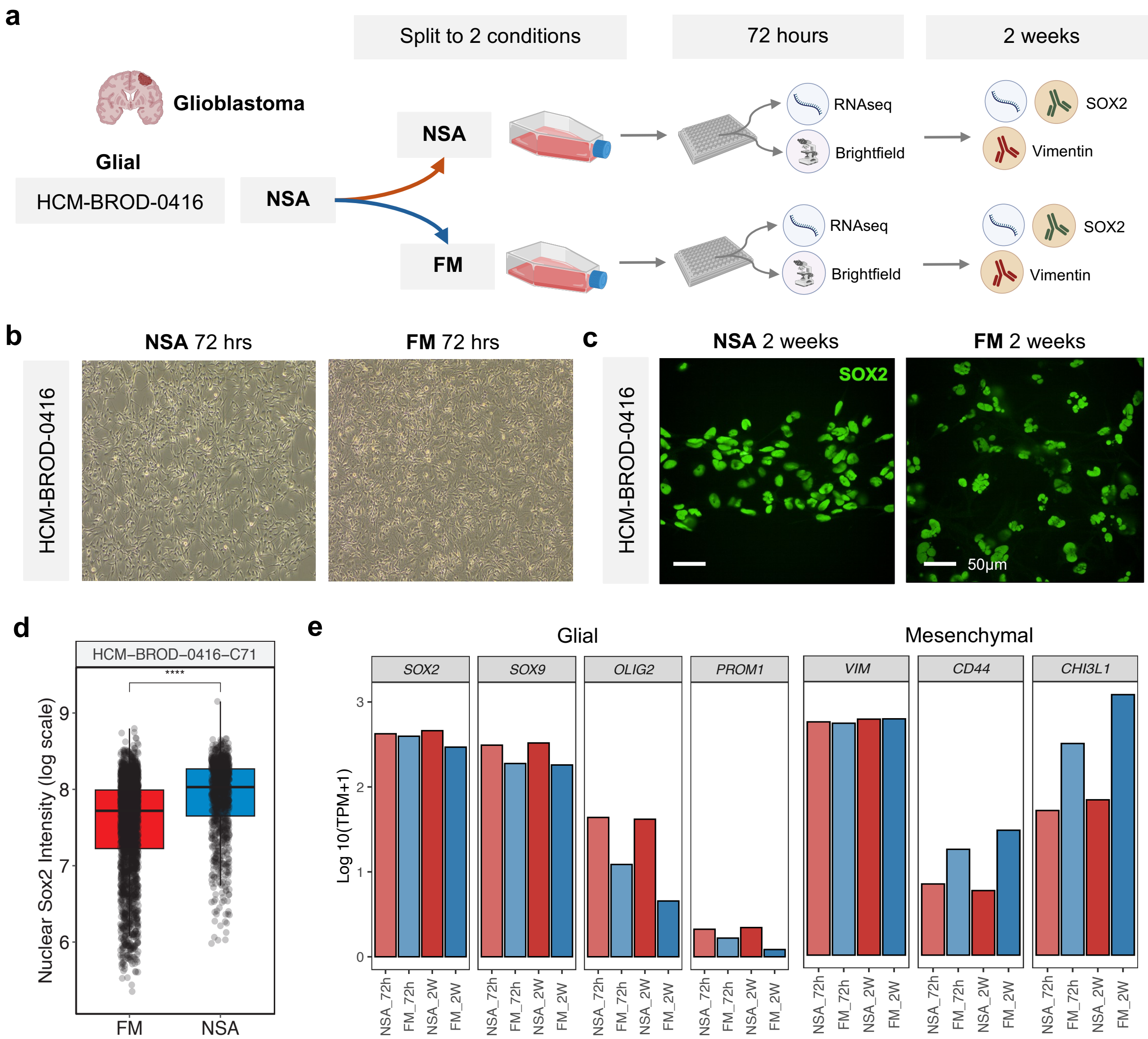

Supplementary Figure 5: Modulation of media-driven transcriptional states in glioblastoma. (a) Schematic overview of the experimental design. The glioblastoma model HCM-BROD-0416, originally maintained under serum-free NSA conditions, was split and re-cultured in NSA or FM. RNAseq and bright field imaging were performed 72 hours later. **(b)** Representative brightfield images of HCM-BROD-0416 after 72 hours and two weeks in NSA or FM. **(c)** Immunofluorescence staining of SOX2 after two weeks of culture in NSA or following transition to FM. **(d)** Quantification of nuclear SOX2 intensity across >10,000 cells per condition. **(e)** RNA-seq expression of glial (SOX2, SOX9, OLIG2, PROM1) and mesenchymal (VIM, CD44, CHI3L1) lineage markers across media conditions and time points.

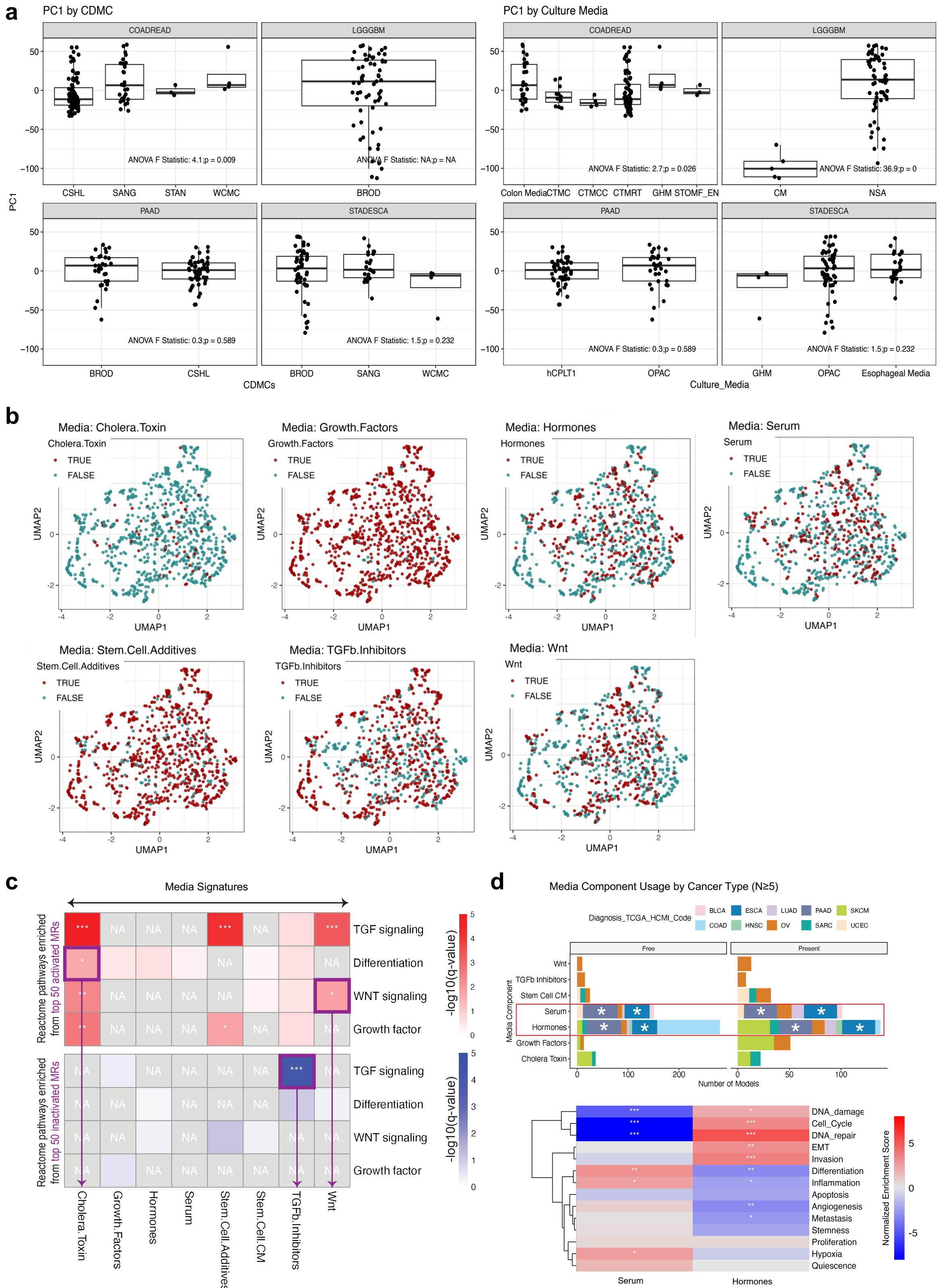

Supplementary Figure 6: Systematic effects of culture medium components on transcriptional states across HCMI models. (a) Distribution of PC1 scores by CMDC (left) or Culture medium (right).

Boxplots of the first principal component of the top 2,000 most variably expressed genes displayed for four tumor types. ANOVA F statistic and p value are displayed. **(b)** UMAP plot color-annotated with individual media components: Serum, WNT, cholera toxin, growth factors, TGF-beta inhibitors, stem cell additives, and hormones. **(c)** GSEA of cancer cell states for each media condition-specific signature. The heatmap displays normalized enrichment scores (NES), with red indicating positive NES and blue indicating negative NES. Columns represent media condition-specific signatures, and rows represent cancer cell states obtained from CancerSEA[1]. **(d)** Comparative analysis of media components–specific transcriptional programs in pancreatic (PAAD) and esophageal (ESCA) cancer models. GSEA plots display pathways enriched in models cultured with versus without gastrin or serum.

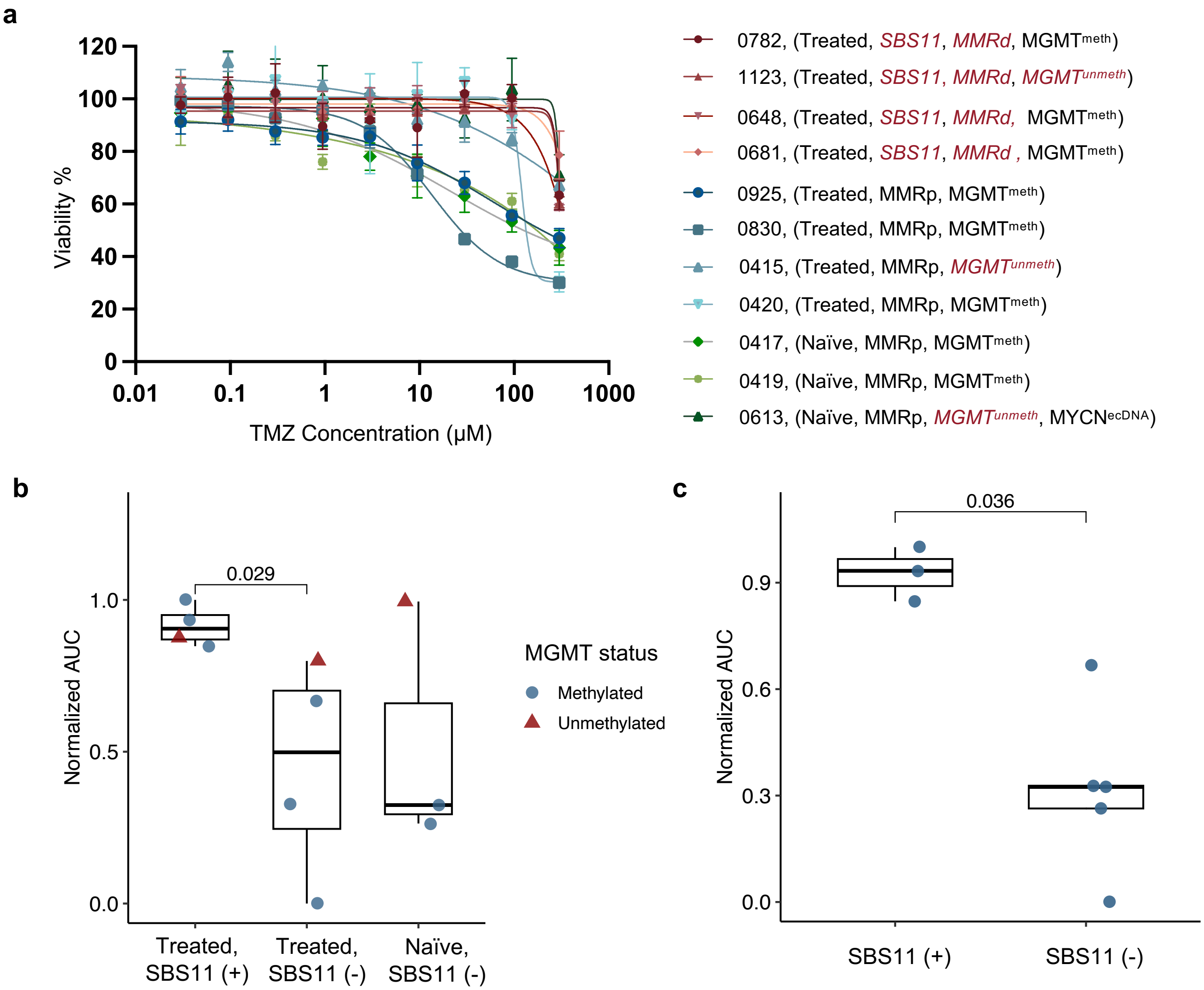

Supplementary Figure 7: Functional validation of temozolomide (TMZ) resistance and associated genomic predictors in glioblastoma models. (a) Dose–response curves for representative GBM models treated with TMZ (0.03–300 µM). **(b)** Distribution of normalized area-under-the-curve (AUC) values comparing treatment-naïve and treated low SBS11 signature (–), and high SBS11 signature (+) models. Colors indicate MGMT promoter methylation status. **(c)** Boxplot summarizing normalized AUC values across MGMT methylated models only stratified by SBS11 mutational signature magnitude: high (+), low (-).

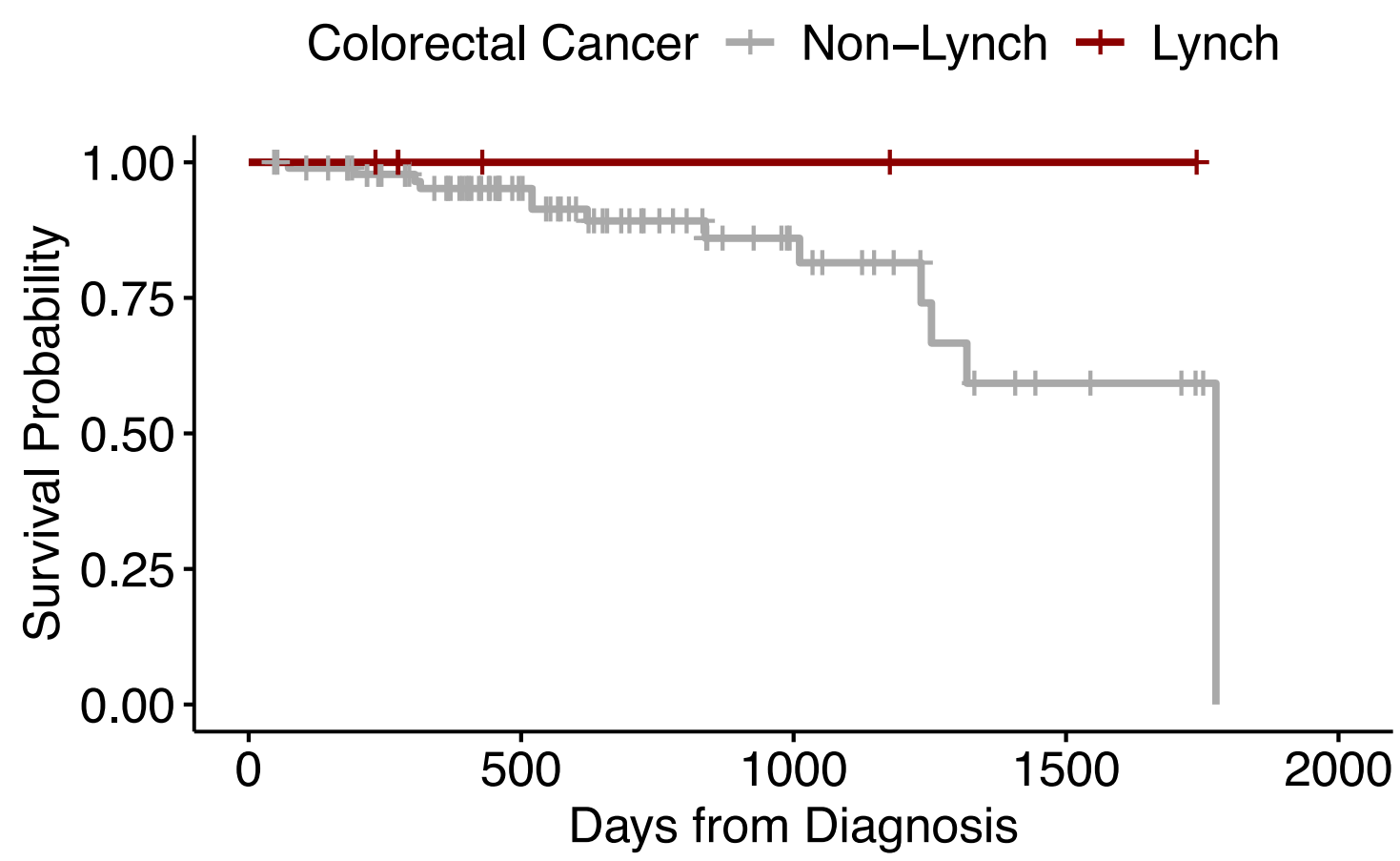

Supplementary Figure 8: Colorectal Lynch Syndrome Kaplan-Meier curves. Comparing survival probabilities of Lynch syndrome-associated colorectal cancers (COAD and READ) to non-Lynch colorectal cancers, with a maximum follow-up of 2000 days in both arms.

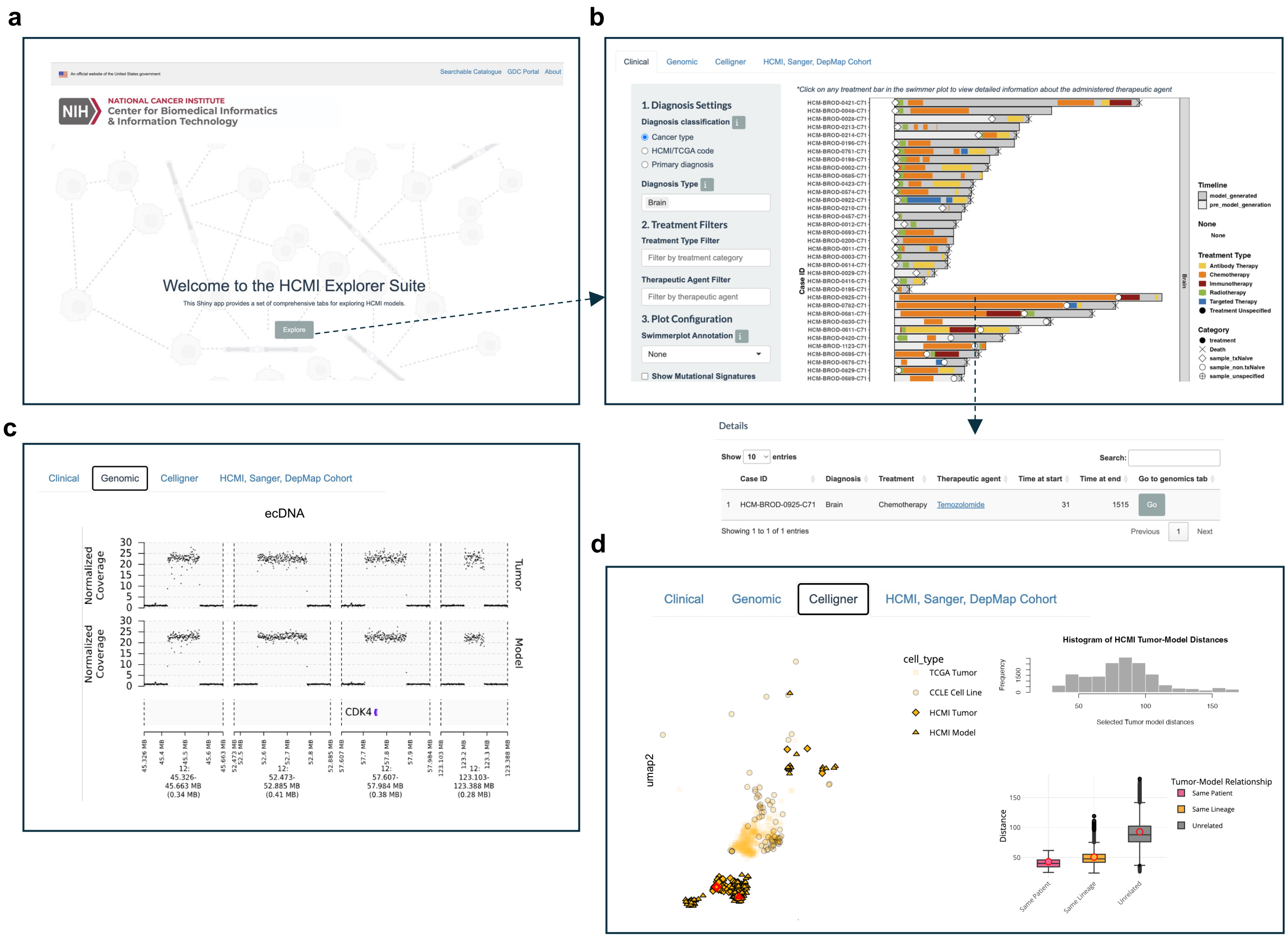

Supplementary Figure 9: Overview of the HCMI Explorer Suite. (a) Overview of the HCMI Explorer Suite home page**, (b)** Clinical module: Swimmer plot visualization showing key treatment and sample collection events pre- and post-model generation, categorized by different therapy types, including chemotherapy, radiotherapy, targeted molecular therapy, and immunotherapy. **(c)** Genomic module: visualization of model/tumor ecDNA amplicons. **(d)** Celligner module: Visualizing UMAP clustering of model and tumor to reference datasets (CCLE/TCGA/TARGET).

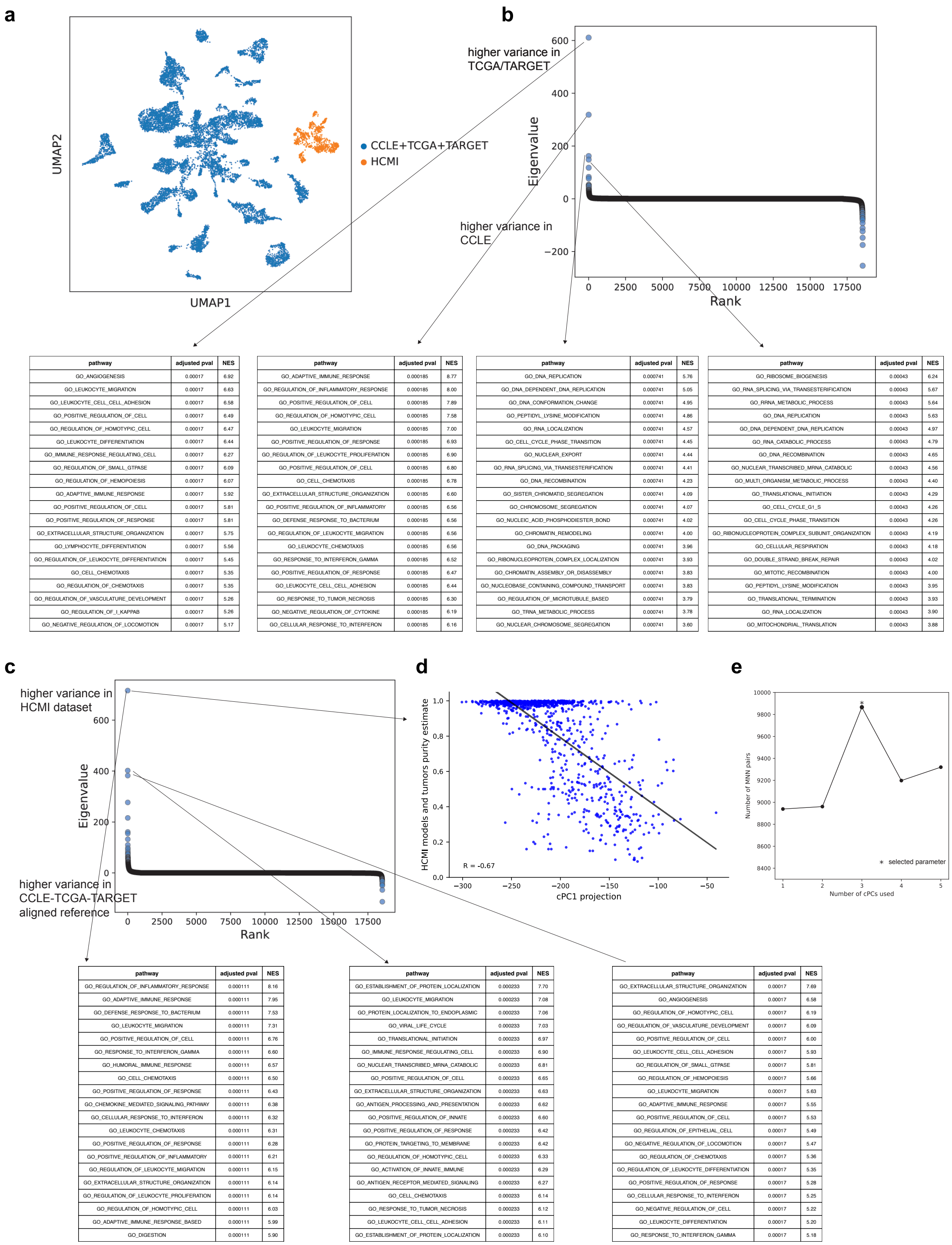

Supplementary Figure 10: Overview of the celligner contrastive Principle Component Analysis (cPCA). (a) 2D UMAP projection of merged, uncorrected expression data from CCLE, TCGA, TARGET and HCMI before Celligner. **(b)** cPC eigenvalues of CCLE-TCGA/TARGET alignment ordered by rank. Arrows show the top twenty pathways from gene set enrichment analysis (GSEA) of the indicated cPCs.

(c) cPC eigenvalues of HCMI dataset to CCLE-TCGA-TARGET reference alignment ordered by rank. Arrows show the top twenty pathways from gene set enrichment analysis (GSEA) of the indicated cPCs. P-values are based on a gene-permutation test and adjusted using the Benjamini-Hochberg procedure. **(d)** Pearson correlation between the projection of HCMI tumors and models onto cPC1 and their estimated purity. **(e)** The number of mutual nearest neighbors based on the different number of cPCs tested. Regressing out the top three cPCs with higher variance in the HCMI data increased the number of mutual nearest neighbors.

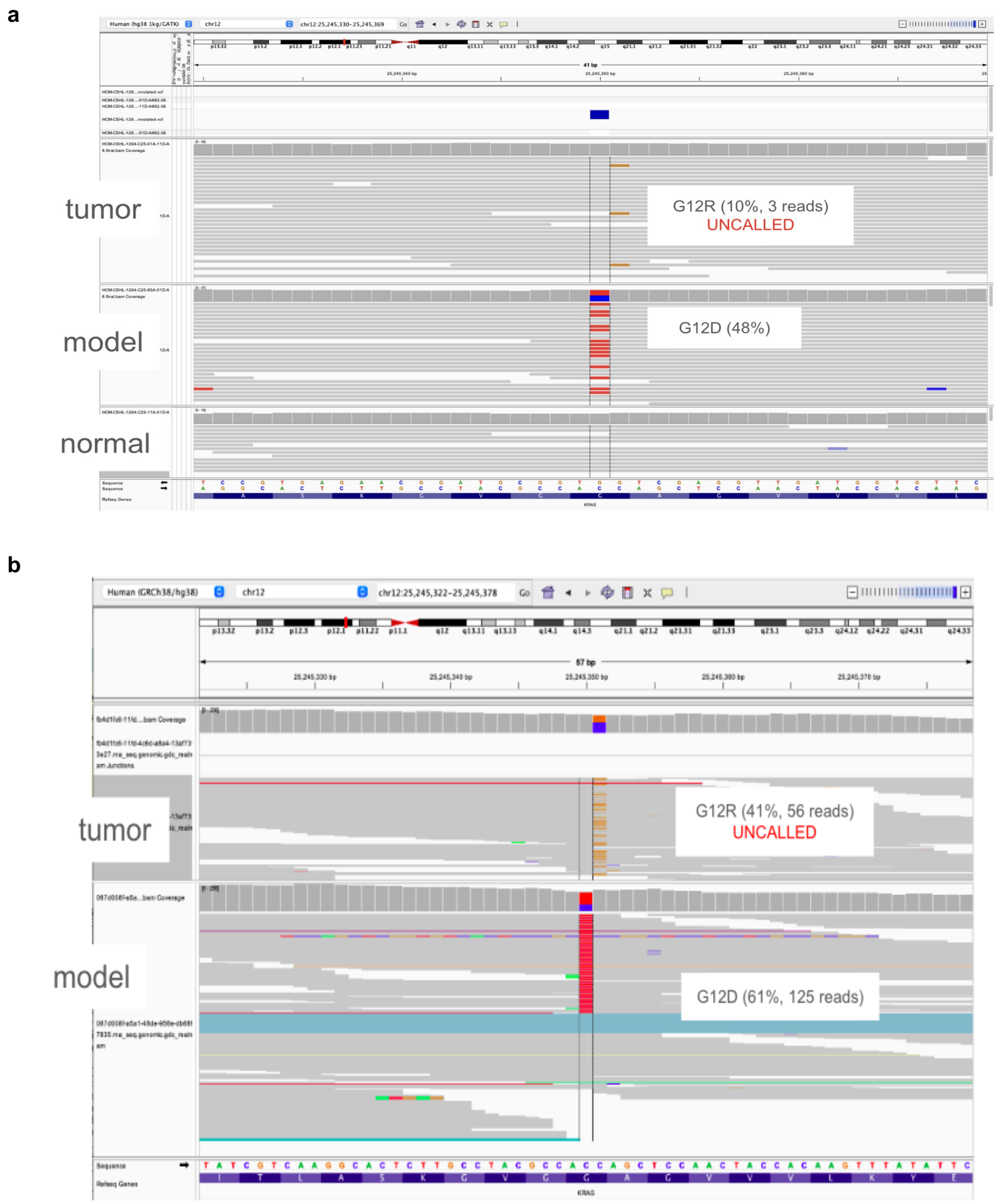

Supplementary Figure 11: IGV screenshot of HCM-CSHL-1264 around the KRAS G12 location; **(a)** WGS, **(b)** RNA-Seq.

a

b

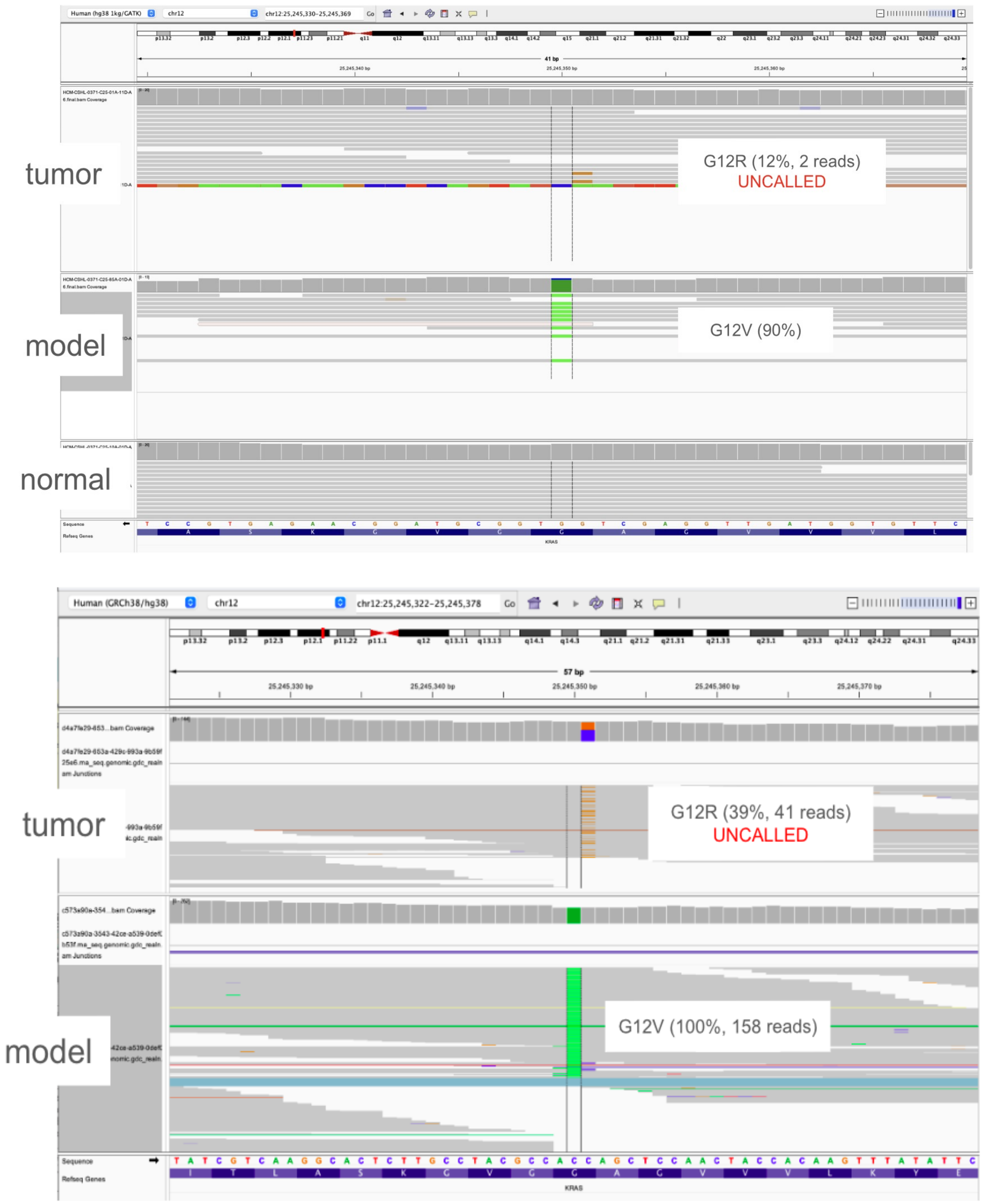

Supplementary Figure 12: IGV screenshot of HCM-CSHL-0371 around the KRAS G12 location; **(a)** WGS, **(b)** RNA-Seq.

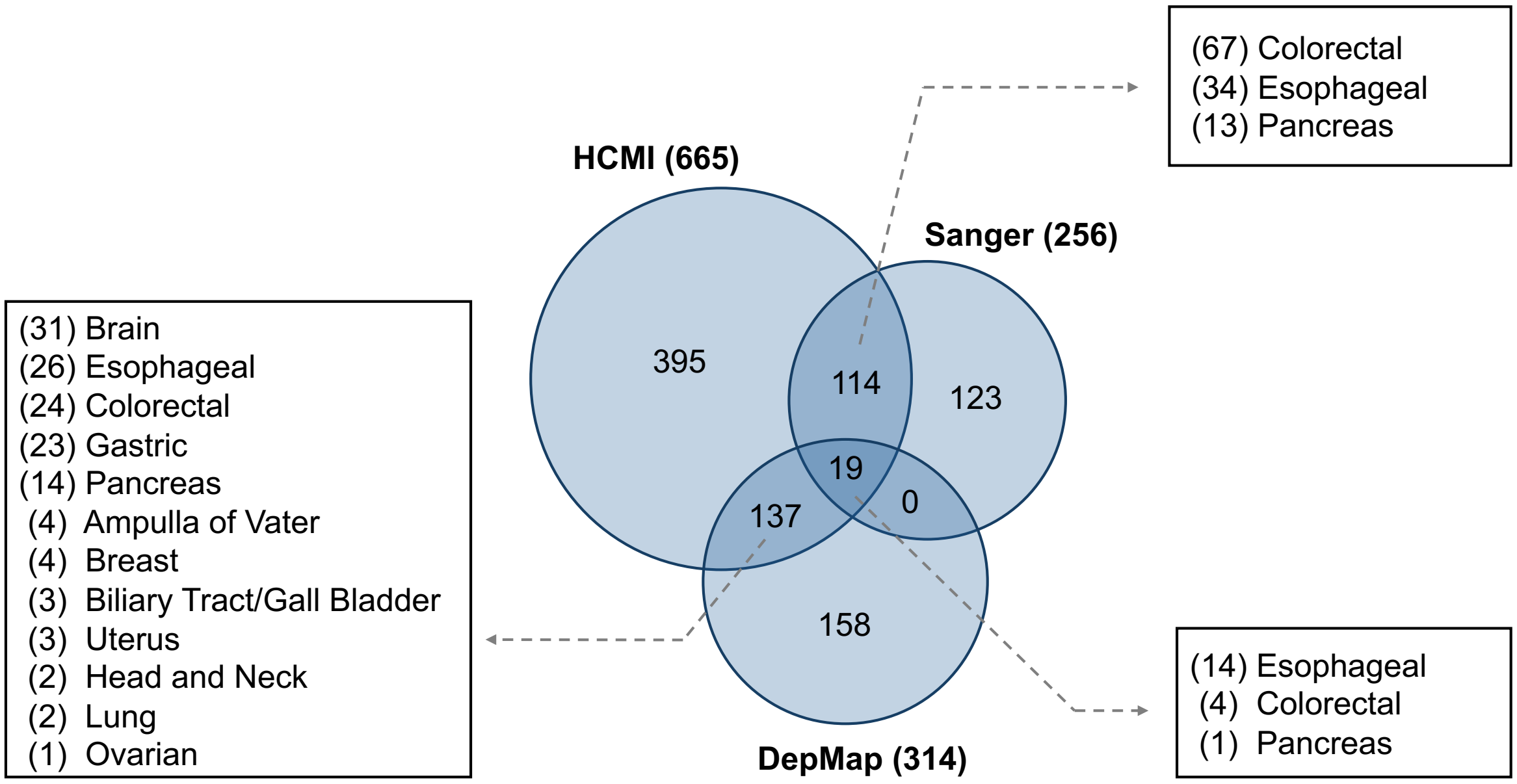

Supplementary Figure 13: Venn diagram illustrating the intersection of models across the three efforts (HCMI, Sanger and DepMap).

Supplementary Table Legends

Supplementary Table 1: Comprehensive metadata and characterization of HCMI models
This table provides detailed metadata and characterization of HCMI models. The table includes model establishment details, growth media compositions, patient demographics, sequencing metadata, clinical timelines, and ancestry prediction calls.

Supplementary Table 2: Mutational signature matrix of the HCMI collection
This table presents the mutational signature matrix of the HCMI collection, based on single-base substitution (SBS) signatures derived from COSMIC.

Supplementary Table 3: Transcriptional and epigenetic fidelity of HCMI models
This table provides the model-tumor transcriptional and epigenetic similarity scores as evaluated by seven independent methods. The table includes a brief description of each method, the score generated by this method for each model-tumor pair evaluated, and TMP and MOMA subtype membership. Moreover, differential gene expression analysis for GBM models grown in NS-A and CM conditions, plus gene set enrichment analysis, are included in this table.

Supplementary Table 4: HCMI-CCLE cell lines and 42 unique HCMI Models
This table provides the Case IDs, Model IDs and cancer types for HCMI Models and the DepMap IDs for the CCLE Cell Lines used for analysis in Fig. 4a. The 42 unique HCMI models shown in Fig. 4b are also listed along with their Case IDs, Model IDs and cancer subtype annotations.

Supplementary Table 5: TCGA-MOMA subtypes
The table provides MOMA subtypes of TCGA samples for COAD (sheet: TCGA-MOMA_Colorectal), STAD (sheet: TCGA-MOMA_Gastroesophageal), GBM (sheet: TCGA-MOMA_Glioblastoma), and PAAD (sheet: TCGA-MOMA_Pancreatic) cohorts, which were analyzed for the MR activity in Fig. 4f. The first column in each sheet denotes TCGA sample IDs, the second column indicates sample type, and the third column represents MOMA subtypes identified for individual samples. For TCGA MOMA subtypes, we utilized clustering annotations of TCGA MOMA subtypes (COAD: 8 subtypes, STAD: 3 subtypes, GBM: 5 subtypes, and PAAD: 7 subtypes).

Supplementary Table 6: HCMI-MOMA subtypes
The table provides MOMA subtypes of HCMI samples for COAD/READ (sheet: HCMI-MOMA_Colorectal), STAD/ESCA (sheet: HCMI-MOMA_Gastroesophageal), GBM/LGG (sheet: HCMI-MOMA_Glioblastoma), and PAAD (sheet: HCMI-MOMA_Pancreatic) cohorts, which were analyzed for the MR activity in Fig. 4f. The first column in each sheet denotes HCMI sample IDs, the second column indicates sample type, and the third column represents MOMA subtypes identified for individual samples. HCMI MOMA subtypes were identified using OncoMatch analysis with TCGA MOMA signatures, as described in the methods.

Supplementary Table 7: Top 50 MRs for MOMA subtypes
The table provides the top 50 most activated MRs for each MOMA subtype analyzed using TCGA and HCMI samples for Fig. 4f.

Supplementary Table 8: Overview of samples profiled using single-nucleus RNA sequencing
Overview of samples profiled using snRNA-seq, including case identifiers, short case identifier (used in Figures), whether the sample is a tumor (T) or model (M), type of sample, original tissue site, notes on the disease, cancer cohort, model type, associated culture media, Multiplex ID, identifier for the snRNA-seq sample (comprising multiplex ID and Case ID) and median UMI/nucleus before and after quality-control and filtering.

Supplementary Table 9: Differentially expressed genes for GBM Tumor-Model pairs profiled at snRNA-seq
This table provides the top 30 differentially expressed genes (DEG) for each of the seven GBM tumor model pairs, identified using the Wilcoxon Rank Sum test. Each sheet corresponds to a specific GBM tumor-model pair, listing genes ranked by the score computed with 'scanpy.pp.rank_gene_groups'. Top and bottom 15 genes are upregulated in the tumor and model, respectively.

Supplementary Table 10: Curated genes for identifying copy number variation driver events
This table presents expert-curated driver genes associated with common copy number variations, categorized by cancer type. Genes are specifically classified based on amplification or homozygous deletion events.

Supplementary Table 11: DNA methylation and RNA UMAP coordinates
This table contains the coordinates for each sample in the DNA methylation and RNA UMAP plots shown in Fig. 4c, Fig. 4d, Extended Fig. 5c, and Extended Fig. 5d. It also provides the probe IDs for the cancer-associated CpGs that were used to generate the DNA methylation UMAP.

Supplementary Table 12: Model-tumor celligner distances and lineage assignment
This table lists the Celligner-based distances between every model-tumor pair and the lineage category to which each sample belongs.

Supplementary Table 13: SNV/indel/CNV events from driver genes
This table summarizes all driver events, including SNVs, indels, and CNVs, from the selected driver genes mentioned above. Only tumor-model pairs with available data for both simple somatic alterations and copy number variations were included. For the event column, hlamp: high-level amplification, homdel: homozygous deletion, missense: missense somatic SNV, truncating: truncating somatic SNV/indels, insertion: non-truncating somatic insertion, deletion: non-truncating somatic deletion, promoter: TERT promoter mutation. The variant column is a unique identifier for the driver mutation of the gene for a sample.

Supplementary Table 14: ABSOLUTE input data source
Indicates whether whole-exome sequencing (WES) or whole-genome sequencing (WGS) was used for ABSOLUTE analysis for each tumor–model pair.

Supplementary Table 15: HCMI Models Media formulations Matrix
Matrix detailing components of each culture medium used for model derivation, including basal media, supplements, growth factors, and small-molecule additives.

Supplementary Materials and Methods

Establishment of Cancer Model Development Centers (CMDCs)

To address the need for better tools to study cancer, NCI's Center for Cancer Genomics (CCG), in collaboration with international institutions, has established the Human Cancer Models Initiative (HCMI) consortium, with a goal to make available to the scientific community large numbers of "next generation" patient tissue-derived *in vitro* cancer models. International consortium members include the Wellcome Sanger Institute (Hinxton, United Kingdom), Cancer Research UK (Oxford, United Kingdom), and HUB Organoids (Netherlands).

Sponsored by NCI's Center for Cancer Genomics, to contribute towards the goals of the HCMI, beginning in 2016, a number of Cancer Model Development Centers (CMDCs) were established through the management of Frederick National Laboratory for Cancer Research (FNLCR). The four funded CMDCs are:

1. The Broad Institute CMDC (BROD CMDC) (Cambridge, MA)
2. Cold Spring Harbor Laboratory (CSHL CMDC) (Cold Spring Harbor, NY). The CSHL CMDC also had the following sites contributing towards the HCMI goals.
   - Northwell Health (New Hyde Park, New York, USA)
   - University of Verona ARC-Net Research Centre (Verona, Italy)
   - Hubrecht Institute (Utrecht, Netherlands)
3. Weill Medical College of Cornell University (WCMC CMDC) (New York, NY)
4. Stanford University (STAN CMDC) (Palo Alto, CA)

Each NCI-supported CMDC completed HCMI project requirements per the HCMI Programmatic Workflow (**Supplementary Fig. 1**). Tasks included: enrolling donors, collecting tissues, establishing and validating cancer models, and submission of biospecimens and associated clinical and biospecimen data, in accordance with the HCMI requirements and guidelines.

Human subjects protocol and other regulatory requirements

During the initial phase of the project, each CMDC, in collaboration with other organizations participating in the HCMI, completed the regulatory requirements for this project. These requirements included: Institutional Review Board (IRB)-approved Protocol and Informed Consent documents; Institutional Certification Letter; Material Transfer Agreements (MTAs) as needed (such as with the Biospecimen Processing Center (BPC), Nationwide Children's Hospital, Columbus, OH); Data Use Agreement (DUA) with the Clinical Data Center (CDC) (Information Management Services, Inc., Calverton, MD); and Material Deposit Agreement (MDA) with HCMI distributor (American Type Culture Collection [ATCC], Manassas, Virginia, USA).

Patient recruiting, consenting, and enrolling donors for the HCMI consortium were conducted by the CMDCs, in compliance with human subject protocol requirements, as mandated by the IRB. Biospecimens were only collected from the donors when their signed informed consent and IRB protocols met the HCMI requirements per the guidelines provided in HCMI consent template (https://www.cancer.gov/ccg/research/functional-genomics/hcmi/using-hcmi#informed-consent-template-for-hcmi). To support acquisition of specimens from underrepresented cancer patients for the Development of Models for the Human Cancer Models Initiative (HCMI), biospecimens, along with associated clinical data were obtained and shipped to the CMDCs by the NCI supported Tissue Source Sites (TSS), which were focused on procuring specimens from underrepresented cancer patients.

Sample labeling

Prior to initiating model generation activities, each CMDC and/or the associated Tissue Source Sites (TSSs) created and managed a database with the local participant identifier (ID1). The ID1 was not shared outside of the CMDC. Each ID1 was linked to a non-identifying random participant code (ID2); ID2s were also assigned by the CMDC. Each NCI-designated CDC was responsible for creating a tertiary, de-identified random code (ID3) that was unique to each subject. Each ID3 was the final (and only) ID attached and used in all public repositories of HCMI (including the cancer models, associated clinical and molecular data, SOPs, etc.). Below is an example of an ID3:

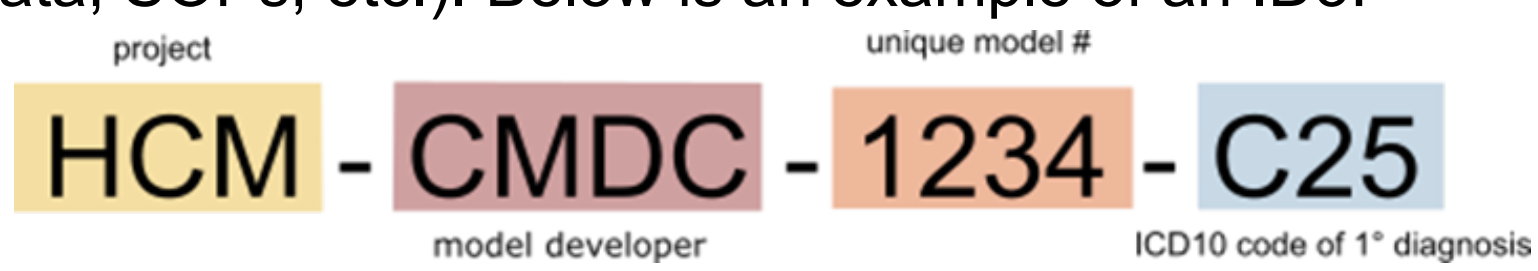

Clinical data

Cancer-specific Case Report Forms (CRFs; https://www.cancer.gov/ccg/research/functional-genomics/hcmi/using-hcmi/case-report-forms) were developed through HCMI Clinical Data Working Groups, which included oncologists, pathologists, geneticists, and bioinformatics experts.

Clinical data that were collected and submitted satisfied the following requirements:

- A minimum set of required demographic and clinical data variables.
- Treatment information and initial response.
- Follow-up clinical data, including disease state and vital status.

Key features of the clinical data include:

- All variables were encoded as Common Data Elements (CDEs) and were registered with the NCI’s Cancer Data Standards Registry (caDSR).
- Clinical and research events were recorded to integer-day intervals from the donor’s index date, which included date of diagnosis, date of sample procurement, or date of initial visit.

- Collection of treatment data includes specific treatment intervals or a definitive assertion of non-treatment, enabling the classification of models as being exposed to treatment or treatment-naive tumor tissue.

Each CMDC, in conjunction with a clinical site, submitted participant data (e.g. clinical presentation, treatment, outcome, pathology, etc.) for all cases to the HCMI CDC, using either a web-based CRF or by direct upload through an API (Application Programming Interface). Once in the CDC system, the clinical data was converted to a standardized JSON format, validated, verified as de-identified, and translated to conform to the NCI Genomic Data Commons (GDC) data dictionary and data model prior to upload to the GDC. In addition, the raw clinical data are available at the GDC HCMI Program page as supplemental files.

The raw, forms-based clinical data were transformed into two tabular files (and a README file) used for the analyses in this paper and made available with **Supplementary Table 1**

Biospecimen collection and establishment of in vitro cultures:

Each CMDC implemented processes for tissue acquisition and processing, which included close collaborations with clinicians for, and engaging with, different clinics and hospitals for procurement of specimens from cancer patients. For each case, cells were expanded to sufficient biomass to be able to freeze at least 12 vials of 10^6 cells per vial (at each time point / passage number to ensure that cells submitted to the BPC and to ATCC (per the HCMI Programmatic Workflow) for the time point had the same characteristics for all downstream characterizations).

Biospecimen Processing Center (BPC)

For each case, two vials of the cancer model (comprising a minimum of 10^6 cells), ~50 mg from the original tumor material, and source of germline DNA (preferably blood, or buccal cells, or an alternative source of high quality (>2kb) germline DNA) were batch shipped to the BPC.

The BPC isolated DNA and RNA performed quantification and quality control of the nucleic acids and confirmed that materials were a genotypic case-match between the primary tumor, model cell pellet, and normal tissue. The BPC then shipped these nucleic acids to the Genomic Characterization Centers (GCCs) for molecular characterization.

HCMI distributor (ATCC)

CMDC- provided vials of HCMI models were received by ATCC and stored in vapor phase liquid nitrogen. CMDC provided protocols and media formulations were reviewed and adapted for bioproduction and published in our quality management system. Prior to bioproduction, each model was subject to quality control testing following standard, validated protocols which included short tandem repeat (STR) profiling via the PowerPlex® 18D kit (Promega), cross contamination testing via

cytochrome oxidase C1 (CO1) barcoding PCR assay, sterility testing for aerobic and anaerobic microbes using the BacT/ALERT 3D Microbial Identification System (bioMérieux), human pathogenic virus testing (HIV, HepB, HPV, EBV, and CMV) via PCR assay, and detection of mycoplasma (ATCC catalog No. 30-1012K). Models were thawed, expanded, and cryopreserved according to model-specific protocols and media formulations recommended by the depositor. During expansion, cultures were monitored for growth and morphology via brightfield microscopy. Cell counts and viability determination was performed using an automated cell counter. Cryopreservation was performed in a controlled rate freezer with typically 2-4x10^6 viable cells per vial in ~1.0 mL cryopreservation media. After cryopreservation, vials were stored in vapor phase liquid nitrogen. For each lot produced, random vials were selected and tested for STR profile, CO1 barcoding, sterility, mycoplasma and pathogenic viruses as described above. At least two vials from each production lot were tested for recoverable cell count (>1.0x10^6) and viability (>50%) and the ability to grow for at least 2 passages in culture. Master cell bank lots were produced for the further generation of distribution lots to maintain low passage numbers.

Of the models ATCC has received, ATCC has manufactured 316 models (Human Cancer Models Initiative (HCMI) | ATCC). Additional unexpanded models can be found on the ATCC website (HCMI Unmanufactured Models | ATCC). All ATCC purchases require an executed ATCC materials transfer agreement (MTA) and certain models that use the HUB organoid technology also require an executed addendum to the ATCC material transfer agreement for organoid products. These documents can be found on the product pages for the models on the ATCC website and further information can be found on the ATCC website under Permits and Restrictions | ATCC.

Molecular characterization:

The samples were shipped from the Biospecimen Processing Center (BPC) to two HCMI Genome Characterization Centers (GCCs).

The Broad Institute (Boston, MA) GCC received DNA samples, and performed:

- >150X whole exome sequencing (WES) of the model, normal, and tumor material.
- 15X & 30X whole genome sequencing (WGS), using NovaSeq PCR-Plus, of the model, normal, and tumor material; and
- Methylation arrays, >831k sites per sample, using the Illumina Infinium Methylation EPIC BeadChip arrays, of the model and tumor material.

The University of North Carolina (UNC; Chapel Hill, NC) GCC received RNA samples, and performed transcriptome sequencing (RNAseq and miRNAseq):

- Total RNAseq, 150M reads, using the Illumina HiSeq 4000, for the model and tumor material; and
- miRNAseq, using Illumina NextSeq 2000 and NovaSeq 6000, for available model and tumor material.

The resulting primary data, BAM files (from WXS and WGS) and FASTQ files (from RNA sequencing), were uploaded to the Genomic Data Commons (GDC).

CMDC model development

BROD CMDC

Sample collection and transport

Fresh tissue samples were collected from patients with informed consent under appropriate institutional IRB approved protocols. Patient whole blood, adjacent normal tissue and tumor tissue were obtained within 3-24 hours of collection at the clinical site. To transport to the laboratory, solid tissues were placed in 15mL conical tubes submerged in Dulbecco's Modified Eagle's Media (DMEM) containing 10% FBS (Sigma, F8317) and penicillin/streptomycin. The samples were placed inside Nanocool boxes (FedEx) during shipment to preserve samples at 2-8°C during transport. Upon receipt, the samples were processed immediately or stored at 4°C then processed within 24 hours. Cell lines were then established using disease specific protocols as described below.

Tissue dissociation and cryopreservation

Tumor tissue samples were transferred from conical tubes to a sterile petri dish using a sterile pipette or tweezers. Any residual media was aspirated, and 5 mL of phosphate-buffered saline (PBS) was immediately added to the tissue. A second petri dish was prepared by adding 10 mL of PBS and set aside for subsequent processing. The resected tissue was briefly rinsed with 1 mL of PBS and immediately transferred using sterile forceps to the second petri dish containing PBS. The tissue was gently swirled in PBS, and the PBS was then aspirated. A small piece is collected from the solid tissue and frozen inside a cryovial on dry ice. To further cleanse the remaining tissue, 5 mL of wash media (DMEM + 10% fetal FBS + penicillin/streptomycin) was added to the petri dish. The tissue was then minced into 1-3 mm^3 fragments. For tissues that appeared sufficiently soft and dissociable via mechanical means, enzymatic digestion was not performed. Otherwise, the 5 mL of wash media containing minced tissue is transferred to a 15mL conical tube, to which 1 mL of collagenase (StemCell, cat. #07912) and 2 mL of dispase (StemCell Technologies, cat. #07913) were added to a final volume of 8 mL. After mixing by inversion three times, the tube was placed on a rotator and incubated at 37°C with rotation for a maximum of two hours. Visual inspections to confirm full digestion occurred every 30 minutes. Once digestion is complete, 200 uL of DNase I (StemCell Technologies, cat. #07900) is added to the sample and the tube is returned to the rotator/incubator for an additional 5 minutes. The sample is then centrifuged at 250 g for 5 minutes and the supernatant is discarded. If the sample contains a visible layer of red blood cells, 2 mL of ACK Lysis Buffer (Gibco, cat. #A10492-01) is used to resuspend the cell pellet then the sample is incubated at room temperature for 5 minutes. The lysis buffer is then diluted with 10 mL wash media and centrifuged at 250g for 5 minutes then the supernatant is discarded. The sample is then resuspended in 3-5 mL of PBS (depending on sample size) and divided for cryopreservation using 1mL of cell freezing media (Thermo Fisher Scientific, cat. #12648010) per cryovial. A portion of the cell suspension in PBS is also collected by centrifugation on a benchtop centrifuge for 2 minutes. The PBS supernatant is discarded, and the pellet is frozen on dry ice.

Media-matrix (Hybrid) model generation

Before seeding cells, a matrix of media recipes is first established based on disease cohort. Briefly, 4-8 individual media conditions are mixed in 1:1 ratio creating a 16 to 64-media matrix. All recipes are available at (https://cellfactory.broadinstitute.org/#/sops). The day of seeding, 200 uL of each media is aliquoted into a 96-well plate. Thawed cells are first spun at 250 g for 5 minutes then resuspended in PBS and 50 uL of cell suspension is added to each well. Cells are then incubated at 37°C at 5% CO2. Approximately 24 hours after seeding, the media is replaced with fresh media. Visual inspection under microscope and automated imaging using the Incucyte (Sartorius) are used to track confluency. When a well is approximately 80% confluent, the cells are passaged to 48-well plates, then 24-well, then 6-well plates. A portion of confluent cultures at this stage are then taken for genomic verification, and the remainder of cells is cryopreserved. Verified models are then thawed and expanded in tissue culture flasks up to 15 passages before being deposited into ATCC. During the expansion process, additional genomic pellets are collected for verification. Cell and media aliquots were collected for mycoplasma testing (ATCC).

Neurosphere generation

Tumor samples were enzymatically and mechanically dissociated and then cultured in Neurosphere cultures conditions. Primary cultures were cultured in Neurocult NS-A Stem Cell media (Stemcell Technologies) supplemented with epidermal growth factor and fibroblast growth factor at 20 ng/mL as well as 0.2% heparin. Models were propagated in both ultra-low attachment flasks as well as laminin coated flasks. Cells were passaged by incubating with Accutase (StemCell Technologies) at 37C for 5 minutes and cultures were resuspended and seeded at a concentration of 3.0e5 cells/ml.

Organoid model generation

Tissues were finely minced into 1–2 mm^3 pieces and enzymatically digested at 37C for 15-30 minutes using complete organoid medium, 1mg/ml collagenase XI, 10uM Y27632, and 10ug/ml DNase. Dissociated cells were used to initiate patient-derived organoids as previously described[2,3]. In brief, cells were plated with Matrigel (Corning) and complete growth medium: advanced DMEM/F12, HEPES 10mM, Glutamax 1X, A83-01 500nM, hEGF 50ng/mL, mNoggin 100ng/mL, hFGF10 100ng/mL, hGastrin I 0.01μM, N-acetylcysteine 1.25mM, Nicotinamide 10mM, B27 supplement 1X final, R-spondin1 conditioned media 10% final, Wnt3A conditioned media 50% final.

2D Adherent model generation

Tissue samples were enzymatically and mechanically dissociated and then cultures were initiated with a minimum of 4 and a maximum of 8 media recipes in combinations (16 to 64 total conditions). Cells were passaged at 80% confluence using TrypLE (Thermofisher) and reseeded at a concentration of 2 to 5 x 10^4 cells per cm^2. Cells were passaged at minimum 5 times and then genomically verified using a custom sequencing panel to confirm which media condition more closely recapitulated the original tumor sample. Models that maintained original tumor profiles were further expanded.

WCMC CMDC

Patient-derived organoids from the Englander Institute for Precision Medicine (EIPM) were generated as previously described[4,5]. Tissue samples were collected with informed consent at Weill Cornell Medicine (WCM). Tissues were washed, mechanically dissected, and enzymatically digested with collagenase IV (Life Technologies). After digestion, the suspension was centrifuged, washed, and resuspended in organoid-specific media (details available online). The cell pellet was plated in a 1:2 matrix:media ratio for organoid formation, with media changes twice a week. For passaging, organoids were dissociated with Accutase (STEMCELL Technologies) or TrypLE Express (Gibco) (5-7 minutes, depending on the model) and cryopreserved in freezing medium (Corning). Cultures were routinely screened for mycoplasma. When available, organoids were validated against parent tumors through histopathology by the WCM Center for Translational Pathology and reviewed by board-certified pathologists. Whole exome or targeted sequencing was performed by the WCM Clinical Genomics Laboratory and analyzed by the EIPM Computational Team to assess mutational and copy number concordance with the parent tumors.

CSHL CMDC

Establishment and culture of organoids from human colorectal tumor tissues

The method used to establish colorectal tumor organoids from human tissue was performed as previously described in Basic Protocol 1 by Pleguezuelos-Manzano et al.[6]. In parallel, 50 mg of tumor tissue was excised and stored in liquid nitrogen for additional DNA analysis. The established organoids were cultured and passaged as described in Basic Protocol 2 of this paper[6]. 1.0 x 10^6 cells were cryopreserved per vial as described in Basic Protocol 3[6].

Establishment and culture of organoids from human breast tumor tissues

The method used to establish breast tumor organoids from human tissue was performed as previously described by Sachs et al.[7]. Breast tumor tissue was only incubated for 45 minutes in digestion medium. The established organoids were cultured and passaged as previously described[7]. Except, for the breast tumor organoid culture medium 2% Rspo3-Fc Fusion Protein Conditioned Medium was used (ImmunoPrecise Antibodies, R001), 1% Noggin-Fc Fusion Protein Conditioned Medium (ImmunoPrecise Antibodies, N002) and 0.8 ng/ml EGF (Peprotech, AF-100-15-1mg). 1.0 x 10^6 cells were cryopreserved per vial as previously described[6].

Establishment and culture of organoids from human head and neck tumor tissues

The method used to establish, culture and passage head and neck tumor organoids from human tissue was performed as previously described[8]. 1.0 x 10^6 cells were cryopreserved per vial as previously described[6].

Establishment and culture of organoids from human ovarian tumor tissues

The method used to establish ovarian tumor organoids from human tissue was performed as previously described by Kopper et al.[8]. The established organoids were cultured only in ovarian cancer (OC)

medium (also known as M+Est medium) or ovarian surface epithelium (OSE) medium (also known as C420% medium). Instead of Wnt conditioned medium, 0.5 nM WNT Surrogate-Fc Fusion Protein (ImmunoPrecise Antibodies, N001) was used. Passaging of organoids was only done by mechanical shearing. 1.0 x 10^6 cells were cryopreserved per vial as previously described[6].

Establishment and culture of organoids from human bladder tumor tissues

The method used to establish bladder tumor organoids from human tissue was performed as previously described by Mullenders et al.[9]. In parallel, 50 mg of tumor tissue was excised and stored in liquid nitrogen for additional DNA analysis. The established organoids were cultured and passaged as described previously[9]. 1.0 x 10^6 cells were cryopreserved per vial as previously described[6].

Breast cancer organoids

Breast cancer organoids were derived and cultured using a published method[7]. Fresh patient tissue was washed in base media (ADF+++) and weighed; at least 30 mg was allocated and snap-frozen for tumor tissue characterization. Base media recipe (ADF+++) included Advanced DMEM-F12 (Invitrogen, #12634–034) supplemented with 10 mmol/L Hepes (Invitrogen, #15630–056), 1× Glutamax (Invitrogen, #12634–034) and 100 U/mL Pen-Strep (Invitrogen, #15140–122). The rest of the tissue was cut, scalpel-minced into small pieces, and treated with Collagenase IV dissolved in ADF+++ for up to 90 minutes at 37°C with continuous agitation. We washed the cell suspension at 300g for five minutes and treated it with red blood cell lysis buffer (Sigma, #11814389001) for five minutes at room temperature if the pellet appeared red in color. The cell suspension was washed with ADF+++ two times, and plated in 50ul domes of Matrigel on tissue culture plates that were pre-warmed at 37°C. Matrigel (Corning, #356231) was lot-tested for concentrations of 8-10mg/ml before use[6,10]. The domes were allowed to set at 37°C for 15 minutes before adding complete breast cancer organoid media. Organoids were incubated at 37°C to form and grow, with media replacement performed once a week. The organoids were passaged with TrypLE (Thermo Fisher, #12605028) every 15-30 days. For passing organoids were broken down into small clusters of cells and replated in Matrigel domes[6].

Endometrial cancer organoids

Fresh tumor and normal endometrial tissue samples are collected from patients undergoing hysterectomy for endometrial cancer (EC), with informed consent and Institutional Review Board (IRB) approval (IRB study #18-0897). Fresh tissues were washed twice with PBS and minced into ~1 mm^3 tissue fragments in a petri dish using sterile shears. Tissue fragments are transferred to a 15 mL Falcon tube containing RPMI-1640 medium (Sigma-Aldrich #R73-88) with 1 mg/mL Collagenase IV (Sigma-Aldrich #C9407) and 10 μM Y-27632 dihydrochloride (Tocris #1254). Samples were incubated at 37 °C for 1–1.5 hours, with gentle mixing every 15 minutes to promote tissue dissociation. Digestion was monitored under light microscopy to assess the release of cell clusters. The supernatant containing dissociated cells was carefully transferred to a fresh tube, leaving any remaining tissue behind, and centrifuged at 300×g for five minutes at room temperature to pellet the cells. The supernatant wes discarded, and the cell pellet was resuspended in 1 mL of TrypLE™ Express Enzyme (Thermo Fisher Scientific #12604103) containing 10 μM Y-27632 to further dissociate tissue clumps. The mixture was incubated at 37 °C for up to 15 minutes, with gentle pipetting every five minutes, until small cell clumps

were achieved without complete dissociation into single cells. Digestion was halted with Advanced DMEM/F12 (Thermo Fisher Scientific #12634028), and the mixture was centrifuged at 300×g for five minutes. If red blood cells were present, the pellet was resuspended in 1–2 mL of ACK Lysis Buffer (Sigma-Aldrich #A1049201) for two minutes before another centrifugation step. The final pellet was resuspended in a 70:30 mixture of Matrigel® matrix (Corning #356234) and organoid media on ice, and 50 µL domes were plated onto a prewarmed six-well plate. The plate was placed in a CO_2 incubator at 37 °C for 10 minutes to allow Matrigel solidification, followed by the addition of 3 mL of organoid media per well to support organoid growth. Organoid growth was monitored daily to assess viability and prepare samples for downstream analyses. Protocol was adapted from[11,12].

Pancreatic, colorectal and lung cancer organoids

Tumor tissue was processed as previously published by Tiriac *et al.*[2] to generate PDOs. Tumor tissue from resections/biopsies were washed, minced over ice, and incubated in digestion media (1 mg/mL Collagenase XI, 10 µg/mL DNAse I, 10.5 µM Y-27632 in Human complete Medium) at 37°C with mild agitation for up to 1 hour followed by mechanical shaking. Digested tissue was incubated in ACK (Ammonium-Chloride-Potassium) Lysing Buffer to lyse red blood if needed. Following wash steps, cells were plated with Matrigel and grown in Human Complete Medium. While establishing PDOs, culture media was routinely changed, and passing occurred as needed to expand PDO cultures. During passaging, PDOs were harvested from Matrigel using cell recovery solution and broken down to cell clusters/single cells with 5 to 10 min incubation in TrypLE at 37℃ with mild agitation, followed by mechanical breaking, washing and plating. Successful PDO generation was demonstrated by continued growth, expansion and cryopreservation of the PDO cultures[2].

ARC-Net

To establish organoid cultures, we collected fresh tissue specimens from surgical resections. We verified neoplastic cell content histologically via mirror-image cryostat sectioning. Specimens were then preserved on ice in Human Splitting Medium and enzymatically digested with Collagenase II, Dispase I, and TrypLE before embedding in Matrigel as reported previously[13]. Cultures were maintained in Human Complete Medium supplemented with various growth factors and pathway modulators, with medium changes every 3-4 days. Due to a low proliferative index, organoids were passed approximately every 7-10 days[14].

SANG model development

Organoid derivation

Organoid derivation from tumour samples involved initial PBS washes and mechanical mincing. Samples were then either cryopreserved or digested in a buffer at 37°C for 1–2 hours. Post-digestion, we filtered the cell suspension through a 100-µm strainer, centrifuged it at 800g, and repeatedly washed it to eliminate debris. We embedded isolated cells in ECM-media droplets (80:20 ratio, 6.4–9.6 mg/mL protein concentration of Cultrex BME Type 2 (BME-2) Select - 3532-001-02), plated cells on pre-warmed six-well plates, and incubated cells at 37°C to allow matrix polymerization. Finally, we

supplemented wells with media[15,16], containing antibiotics and 1 µL/mL ROCK inhibitor (Y-27632) before incubating.

Whole-Genome Sequencing and alignment
DNA extracted from snap-frozen original tumour tissues, snap-frozen organoid cell pellets or blood samples were prepared for whole genome sequencing (WGS). Whole genome paired-end sequencing reads (150bp) were generated using an Illumina HiSeq X Ten platform, with an average depth of 30x. Reads were aligned using Burrows-Wheeler Alignment (BWA-MEM)[17]. Before downstream analysis, we filtered out PCR duplicates, unmapped and non-uniquely mapped reads.

STAN CMDC

Sample procurement
Patients provided written and informed consent to donate tissue and blood samples for this study, covered by Stanford IRB approved protocol #28908. Excess clinical tissue samples were collected from clinical pathology and immediately transferred to HypoThermosol ® FRS Preservation medium (StemCell Technologies, #07936) containing 100 ug/mL Primocin (InvivoGen) and stored at 4℃. Blood was collected in a BD Vacutainer K2 EDTA tube.

Sample processing
Fresh tumor tissue was transferred to the lab on ice and processed within 1-2 hours of procurement. Tissue was sectioned into equal fragments and was randomly distributed for FFPE generation, sequencing and organoid generation. Approximately 10% of tissue (not exceeding 50 mg tissue) was fixed in 10% formalin for 18-24 hrs and was paraffin-embedded for histological characterization by Stanford's Pathology Core. Another 10% of tissue (not exceeding 50 mg tissue) was flash frozen for future exome sequencing while remaining tissue (80%) was utilized for organoid generation.

Model generation & expansion
Tissue fragments were further minced and resuspended in Type I collagen (Trevigen) for ALI organoid culture as previously described[18]. Approximately 5% of the minced tissue was flash frozen for sequencing. After one to two passages in ALI culture, organoids were transferred to submerged BME for continued growth and expansion, using submerged organoid culture methods as previously described[17]. Briefly, organoids were released from the collagen bed and digested to small cellular chunks using TrypLE (Gibco) and resuspended in Cultrex RGF BME (Bio-Techne).

DNA extracted from PBMCs: Isolation and storage
Whole blood was processed within 2-8 hours after collection. Peripheral blood mononuclear cells (PBMCs) were isolated using Ficoll-Paque density gradient centrifugation; red blood cells were lysed using RBC lysis buffer (Millipore-Sigma) according to manufacturer's protocols. DNA extracted using Qiagen DNeasy kits.

Model characterization & QC

Derived organoids were fixed and histologically assessed against parental tissue by Stanford's Pathology team. When appropriate, IHC and IF staining was utilized to confirm the presence of tissue-identifying markers or oncogenic biomarkers[18]. To assess potential outgrowth of non-malignant organoids within tumor cultures, we longitudinally sampled organoids for histological evaluation and targeted sequencing, throughout the organoid expansion phase. We utilized the Stanford Actionable Mutation Panel (STAMP) provided by Stanford's Molecular Genetic Pathology Laboratory to confirm that driver mutations were preserved in generated organoid models, comparing these models to the patient's primary tumor. The STAMP panel covers over 130 genes using targeted exome sequencing. Organoid cultures were routinely monitored for potential microbial contamination using three methods: weekly visual inspection of cultures, monthly testing of mycoplasma using the MycoFluor Mycoplasma Detection Kit (ThermoFisher, #M7006), and monthly testing of bacteria, fungi, and yeast using the Cell Culture Contamination Detection Kit (ThermoFisher, #C7028).

Transcriptional State Analysis of Failed versus Successful Tumors

We assessed transcriptional state differences between failed and successful tumors to generate models at the master regulator level. To do this, VIPER inference was applied to differential gene expression data combining both failed and successful tumor samples. In this analysis, differential gene expression signatures were obtained by normalizing and scaling the TPM-normalized expression data by subtracting the median expression across all samples (failed + successful tumors) and dividing by MAD within each HCMI cancer cohort. Protein activity profiles were then assessed for the three largest cohorts (colorectal, pancreatic, and esophageal) in HCMI using the COAD/READ, PAAD, and ESCA/STAD networks, respectively.

Next, we investigated the enrichment of cancer cell states within the protein activity signature of individual samples using the following annotation terms from CancerSEA[1]: Angiogenesis, Apoptosis, Cell_Cycle, Differentiation, DNA_damage, DNA_repair, EMT, Hypoxia, Inflammation, Invasion, Metastasis, Proliferation, Quiescence, and Stemness. The enrichment analysis was conducted using the matrix_narnea() function in the PISCES R package, with a minimum target size of 10. Samples without significant enrichment (NES < 2) for any cancer cell state were classified as "unknown." Furthermore, if a sample showed enrichment with NES > 2 but the second-best cell state also had a high NES (i.e., $NES_{best}/NES_{2ndBest} < 1.5$), the sample was classified as "ambiguous." Finally, we compared the distributions of enriched cancer cell states between failed and successful samples, excluding those classified as unknown or ambiguous (**Supplementary Fig. 3c, 3d, and 3f**).

Media Switching Experimental Assay

HCM-BROD-0416-C71 cells were grown in NSA medium consisting of Neurocult NS-A Basal Medium with 10% Neurocult NS-A Proliferation Supplement (StemCell Technologies, 05751), 20 ng/mL EGF (Thermo Fisher Scientific, PHG0311L), 20 ng/mL bFGF (Miltenyi Biotec, 130-093-564), and 2 µg/mL

Heparin (StemCell Technologies, 07980). During initial thawing, cells were cultured in NSA medium supplemented with 1X antibiotic–antimycotic (Thermo Fisher Scientific, 15240112). All flasks and plates were coated with 0.01% laminin (Bio-Techne, 3400-010-02) in PBS. Cells were maintained at 37°C in 5% CO_2 and passaged at ~80% confluence at a 1:3 split ratio using TrypLE Express (Thermo Fisher Scientific, 12604039) for 5 minutes. All centrifugations were performed at 250 × g for 5 minutes.

After an initial recovery and expansion period in NSA medium, cells were evenly split into laminin-coated flasks. After 24 hours to allow for adhesion, the medium was replaced with FM medium consisting of 50% DMEM F-12 with NaPyr (Thermo Fisher Scientific, 11330032), 50% DMEM with NaPyr (Thermo Fisher Scientific, 11995065), 7.5% fetal bovine serum (Thermo Fisher Scientific, A5669801), 10 ng/mL EGF (Thermo Fisher Scientific, PHG0311L), 5 µg/mL insulin (Thermo Fisher Scientific, 12585-014), 0.4 µg/mL hydrocortisone (Sigma, H0888-1G), 8.6 ng/mL cholera toxin (Sigma, C8052), and 10 µM Y-27632 (Enzo, ALX-270-333-M025). Cells were conditioned in FM medium for 72 hours or two weeks, as indicated in the figures.

Following conditioning, cells were detached with TrypLE Express and 1,000 cells per well were seeded in 50 µL volumes into laminin-coated 384-well black, clear-bottom plates (Revvity, 6057302). After 24 hours in FM medium, cells were fixed, and immunofluorescence staining was performed as described below.

Immunofluorescence Staining

All staining steps were conducted at room temperature. Formaldehyde (14%) was added directly to the 50 µL medium to achieve a final concentration of ~4%. After 20 minutes of fixation, cells were washed three times with 80 µL PBS using a Biotek EL406 plate washer (Agilent). Cells were permeabilized and blocked for 30 minutes in 50 µL of 0.1% Triton X-100 (Sigma, 93443) and 0.5% BSA (Sigma, 126615) in PBS, followed by three PBS washes.

Cells were incubated for 30 minutes with 50 µL of Rat anti-SOX2 monoclonal antibody (Thermo Fisher Scientific, 740013T) at 4 µg/mL or Mouse anti-Vimentin monoclonal antibody (BioLegend, 677802) at 2 µg/mL in 0.5% BSA/PBS. After three PBS washes, cells were incubated for 30 minutes with 2 µg/mL Goat anti-Rat Alexa Fluor 488-conjugated antibody (Thermo Fisher Scientific, A-11006) or Goat anti-Mouse Alexa Fluor 647-conjugated antibody (Thermo Fisher Scientific, A32728TR), together with Hoechst 33342 (1:20,000; BD, 561908). Cells were washed three times with PBS, and 60 µL PBS was added per well for imaging.

Imaging and Quantification

Cells were imaged using the Operetta CLS High-Content Analysis System (Revvity, Waltham, MA, USA) with a 40X water-immersion objective in confocal mode. Fourteen fields of view per well were captured using the following settings: Hoechst 33342 (100 ms exposure, 30% power, 2.0 µm height), Alexa Fluor 488 (300 ms exposure, 30% power, 2.0 µm height), and Alexa Fluor 647 (300 ms exposure, 100% power, 2.0 µm height).

SOX2 nuclear intensity was quantified using Harmony Analysis v5.2 (Revvity). Nuclei were segmented using the Hoechst channel with the "Find Nuclei" module (Method C). The Alexa Fluor 488 intensity per nucleus was measured using the "Calculate Intensity Properties" module, and the mean pixel intensity within each nucleus was reported as the SOX2 intensity.

Media Component–Specific Protein Activity Signatures

To explore transcriptional signatures influenced by individual media components, we categorized samples into binary classes based on the presence or absence of each component: serum, wnt, cholera toxin, interleukin, growth factors, TGFβ Inhibitors, stem cell additives, and hormones. In this analysis, we used protein activity profiles from model samples normalized within each HCMI cancer cohort as described in Methods. Samples sequenced at the Sanger Institute were excluded due to batch effects. Each media component-specific signature was derived from the effect sizes of associations between protein activities and the binary variables representing the eight media components, using the following GLM model: $protein_k$ ~ Serum + Wnt + Cholera_Toxin + Interleukin + Growth_Factors + TGF_Inhibitors + Stem_Cell_Additives + Hormones. In this model, each coefficient represents the association effect between a given media component and protein activity, accounting for the presence of other media components as covariates.

Master regulators corresponding to the top 50 and bottom 50 beta coefficients were applied to Reactome pathway enrichment analysis using the enrichPathway() function from the ReactomePA package[19], with minimum and maximum gene set sizes set to 5 and 50, respectively. Statistical significance (p-values) for pathways related to key media-targeted processes, namely TGF signaling, Differentiation, WNT signaling, and Growth factor pathways, were integrated using Fisher's method (sumlog() in the metap R package[20]) (**Supplementary Fig. 6c**).

To compare media effects across models, we focused on serum and hormone components, as both were relatively balanced between media-present and media-free groups in pancreatic and esophageal cancer models. Serum- and hormone-specific signatures were obtained from the beta coefficients of the following GLM model from both pancreatic and esophageal samples: $protein_k$ ~ Serum + Hormone. Finally, enrichment of cancer cell states within the beta coefficient-based signatures was assessed using the matrix_narnea() function from the PISCES package and gene sets representing 14 cancer cell states from CancerSEA[1] (**Supplementary Fig. 6d**).

References

1. Yuan, H. *et al.* CancerSEA: a cancer single-cell state atlas. *Nucleic Acids Res* 47, D900–D908 (2019).
2. Tiriac, H. *et al.* Organoid Profiling Identifies Common Responders to Chemotherapy in Pancreatic Cancer. *Cancer Discov* 8, 1112–1129 (2018).
3. Boj, S. F. *et al.* Organoid Models of Human and Mouse Ductal Pancreatic Cancer. *Cell* 160, 324–338 (2015).
4. Pauli, C. *et al.* Personalized In Vitro and In Vivo Cancer Models to Guide Precision Medicine. *Cancer Discov* 7, 462–477 (2017).
5. Madorsky Rowdo, F. P. *et al.* Patient-derived tumor organoids with p53 mutations, and not wild-type p53, are sensitive to synergistic combination PARP inhibitor treatment. *Cancer Lett* 584, 216608 (2024).
6. Pleguezuelos-Manzano, C. *et al.* Establishment and Culture of Human Intestinal Organoids Derived from Adult Stem Cells. *Curr Protoc Immunol* 130, (2020).
7. Sachs, N. *et al.* A Living Biobank of Breast Cancer Organoids Captures Disease Heterogeneity. *Cell* 172, 373-386.e10 (2018).
8. Kopper, O. *et al.* An organoid platform for ovarian cancer captures intra- and interpatient heterogeneity. *Nat Med* 25, 838–849 (2019).
9. Mullenders, J. *et al.* Mouse and human urothelial cancer organoids: A tool for bladder cancer research. *Proc Natl Acad Sci U S A* 116, 4567–4574 (2019).
10. Bhatia, S. *et al.* Patient-Derived Triple-Negative Breast Cancer Organoids Provide Robust Model Systems That Recapitulate Tumor Intrinsic Characteristics. *Cancer Res* 82, 1174–1192 (2022).
11. Katcher, A. *et al.* Establishing patient-derived organoids from human endometrial cancer and normal endometrium. *Front Endocrinol (Lausanne)* 14, (2023).
12. Barbi, barbi@cshl. edu *et al.* Generation and Maintenance of Patient-Derived Endometrial Cancer Organoids. *Bio Protoc* 14, (2024).
13. D’Agosto, S., Lupo, F. & Corbo, V. Generation of Pancreatic Organoid-Derived Isografts. *STAR Protoc* 1, 100047 (2020).
14. D’Agosto, S. *et al.* Long-term organoid culture of a small intestinal neuroendocrine tumor. *Front Endocrinol (Lausanne)* 14, (2023).
15. van de Wetering, M. *et al.* Prospective derivation of a living organoid biobank of colorectal cancer patients. *Cell* 161, 933–45 (2015).
16. Li, X. *et al.* Organoid cultures recapitulate esophageal adenocarcinoma heterogeneity providing a model for clonality studies and precision therapeutics. *Nat Commun* 9, 2983 (2018).
17. Li, H. & Durbin, R. Fast and accurate long-read alignment with Burrows–Wheeler transform. *Bioinformatics* 26, 589–595 (2010).
18. Neal, J. T. *et al.* Organoid Modeling of the Tumor Immune Microenvironment. *Cell* 175, 1972-1988.e16 (2018).
19. Yu, G. & He, Q.-Y. ReactomePA: an R/Bioconductor package for reactome pathway analysis and visualization. *Mol Biosyst* 12, 477–9 (2016).

20. Dewey, M. metap: Meta-Analysis of Significance Values. *CRAN: Contributed Packages* Preprint at https://doi.org/10.32614/CRAN.package.metap (2014).
