# Supplementary material for: A compendium of next-generation patient-derived models for diverse cancers
Source: Nature. 2026 Aug 5;657(8132):775–88. doi: 10.1038/s41586-026-10806-y (PMC13581597; doi:10.1038/s41586-026-10806-y)
Supplement: Supplementary file 2 [file 41586_2026_10806_MOESM2_ESM.pdf]

nature portfolio

Corresponding author(s): Keith L. Ligon
Louis M. Staudt
Jesse S. Boehm

Last updated by author(s): 04/10/2026

Reporting Summary

Nature Portfolio wishes to improve the reproducibility of the work that we publish. This form provides structure for consistency and transparency in reporting. For further information on Nature Portfolio policies, see our Editorial Policies and the Editorial Policy Checklist.

Statistics

For all statistical analyses, confirm that the following items are present in the figure legend, table legend, main text, or Methods section.

n/a	Confirmed	
☐	☒	The exact sample size (*n*) for each experimental group/condition, given as a discrete number and unit of measurement
☐	☒	A statement on whether measurements were taken from distinct samples or whether the same sample was measured repeatedly
☐	☒	The statistical test(s) used AND whether they are one- or two-sided<br>*Only common tests should be described solely by name; describe more complex techniques in the Methods section.*
☐	☒	A description of all covariates tested
☐	☒	A description of any assumptions or corrections, such as tests of normality and adjustment for multiple comparisons
☐	☒	A full description of the statistical parameters including central tendency (e.g. means) or other basic estimates (e.g. regression coefficient) AND variation (e.g. standard deviation) or associated estimates of uncertainty (e.g. confidence intervals)
☐	☒	For null hypothesis testing, the test statistic (e.g. *F*, *t*, *r*) with confidence intervals, effect sizes, degrees of freedom and *P* value noted<br>*Give P values as exact values whenever suitable.*
☒	☐	For Bayesian analysis, information on the choice of priors and Markov chain Monte Carlo settings
☐	☒	For hierarchical and complex designs, identification of the appropriate level for tests and full reporting of outcomes
☐	☒	Estimates of effect sizes (e.g. Cohen's *d*, Pearson's *r*), indicating how they were calculated

Our web collection on statistics for biologists contains articles on many of the points above.

Software and code

Policy information about availability of computer code

Data collection	Data collection and metadata curation for the Human Cancer Models Initiative (HCMI) followed a standardized framework integrating clinical, biospecimen, and multi-omics data. Clinical and patient metadata were collected using Case Report Forms (CRFs) and submitted via a web-based system/API to the National Cancer Institute Genomic Data Commons, where data were validated and harmonized into structured JSON formats for downstream integration.

Data analysis	The analysis of the HCMI dataset incorporated a combination of commercial, open-source, and custom software. Whole-exome sequencing (WES) and whole-genome sequencing (WGS) reads were aligned using BWA-MEM (v0.7.15), with quality control performed using Picard Tools (v2.26.10) and base recalibration using GATK (v3.7.0). Somatic variant calling was performed using MuTect (v1.1.7), Strelka (v2.9.10), VarScan (v2.3.8), and deTiN (v1.0). Structural variants were identified using Manta (v1.6.0) and SvABA (v1.1.0), and copy number alterations were inferred using Hatchet (v1.0). Variant calls were merged and processed using BCFTools (v1.10.2).<br>RNA sequencing data were aligned using STAR (v2.7.3a), quantified using Salmon (v1.3.0), and normalized using DESeq2 (v1.30.1) with variance-stabilizing transformation in R (v4.1.0). DNA methylation data generated using the Illumina HumanMethylationEPIC array were processed using SeSAMe (v1.18.4) in R.<br>Downstream computational analyses included pathway and regulatory network inference using Gene Set Enrichment Analysis (GSEA v4.1.0) with MSigDB Hallmark gene sets, pyVIPER (v1.0) for transcription factor activity inference, and NaRnEA (v1.0) for regulatory network enrichment analysis. Transcriptomic harmonization across datasets was performed .using Celligner (v1.0; https://github.com/broadinstitute/celligner). Custom data analysis and visualization were conducted as part of this study, including the development of HCMI Explorer Suite, implemented as an R Shiny application (https://github.com/human-cancer-model-initiative/HCMI-Explorer-Suite/). All custom code used in this study is publicly available at: https://github.com/NCICCGPO/HCMI-Flagship-Manuscript

For manuscripts utilizing custom algorithms or software that are central to the research but not yet described in published literature, software must be made available to editors and reviewers. We strongly encourage code deposition in a community repository (e.g. GitHub). See the Nature Portfolio guidelines for submitting code & software for further information.

Data

Policy information about availability of data

All manuscripts must include a data availability statement. This statement should provide the following information, where applicable:

- Accession codes, unique identifiers, or web links for publicly available datasets
- A description of any restrictions on data availability
- For clinical datasets or third party data, please ensure that the statement adheres to our policy

To support data access, we maintain a publicly accessible instruction portal (HCMI Page at https://www.cancer.gov/ccg/research/functional-genomics/hcmi/using-hcmi), which serves as the central entry point for the resource. Molecular and genomic characterization data from this study are available through the HCMI-CMDC project on the NCI Genomic Data Commons (GDC) web page (https://portal.gdc.cancer.gov/projects/HCMI-CMDC) and its accompanying GDC publication page (https://gdc.cancer.gov/about-data/publications/HCMI-CMDC-2026). Controlled-access raw sequencing data are available through dbGaP under study accession phs001486. Processed molecular datasets (mutation calls, copy-number profiles, clinical annotations) are available through cBioPortal (https://www.cbioportal.org/study/summary?id=pancan_hcmi_2025). Interactive model-level exploration is available through the HCMI Searchable Catalog (https://hcmi-searchable-catalog.nci.nih.gov). All genomic analyses were performed using the human reference genome GRCh38 (Genome Reference Consortium Human Build 38).

Research involving human participants, their data, or biological material

Policy information about studies with human participants or human data. See also policy information about sex, gender (identity/presentation), and sexual orientation and race, ethnicity and racism.

Reporting on sex and gender	Findings in the study report on the self reported biological sex of the patients and their derived models. Consent was obtained for patients involved in the study and reported overall for 665 individuals.
Reporting on race, ethnicity, or other socially relevant groupings	The self-reported race and ethnicity data in the HCMI manuscript was included to ensure a comprehensive understanding of the diversity represented in the patient-derived models. The study collected self-reported race and ethnicity data from patients, which was then compared with genetic ancestry estimates obtained from whole-genome sequencing (WGS) alignments. Genetic ancestry was estimated using the ADMIXTURE (v1.3.0) tool. This approach categorized patients into five continental-level ancestry groups (AFR, AMR, EAS, EUR, SAS), further refined into 23 sub-populations. The race and ethnicity categories were included in the study to assess tumor-model representation and diversity. This categorization was primarily used for demographic reporting and was not incorporated as a direct variable in downstream molecular or functional analyses.
Population characteristics	Covariate-relevant population characteristics collected for participants in the HCMI study included age at diagnosis, sex, genetic ancestry, primary tumor diagnosis, disease stage (when available), and treatment history (including treatment-naïve versus post-treatment status and therapeutic exposures). These variables were systematically recorded using standardized Case Report Forms and curated as part of the clinical metadata linked to each patient-derived model.<br>The study cohort comprised both adult and pediatric patients, with the majority of models derived from adult donors (~93.5%) and a smaller proportion from pediatric and adolescent patients (~6.5%). Genetic ancestry was inferred from germline sequencing data and included predominantly European ancestry, with representation from African, East Asian, South Asian, and admixed populations. Clinical follow-up data, including survival and treatment response, were available for a subset of participants. No restrictions were applied based on age, sex, or disease stage during enrollment.
Recruitment	The participant recruitment for the HCMI study followed a structured approach to ensure compliance with ethical and regulatory standards. Donors were enrolled through the NCI's network of Tissue Source Sites (TSSs).
Ethics oversight	Patient recruiting, consenting, and enrolling donors for the HCMI consortium were conducted by the CMDCs, in compliance with human subject protocol requirements, as mandated by the IRB. Biospecimens were only collected from the donors when their signed informed consent and IRB protocols met the HCMI requirements per the guidelines provided in HCMI consent template (https://www.cancer.gov/ccg/research/functional-genomics/hcmi/using-hcmi#informed-consent-template-for-hcmi).

Note that full information on the approval of the study protocol must also be provided in the manuscript.

Field-specific reporting

Please select the one below that is the best fit for your research. If you are not sure, read the appropriate sections before making your selection.

☒ Life sciences ☐ Behavioural & social sciences ☐ Ecological, evolutionary & environmental sciences

For a reference copy of the document with all sections, see nature.com/documents/nr-reporting-summary-flat.pdf

Life sciences study design

All studies must disclose on these points even when the disclosure is negative.

Sample size

The sample size rationale for the HCMI study was driven by the goal of generating a diverse, well-characterized resource of patient-derived models to expand publicly available cancer models across multiple tumor types. Between 2016 and 2021, a total of 2,780 patients from the United States, United Kingdom, Italy, and the Netherlands consented to participate in the study, donating tumor material, matched germline tissue, and de-identified clinical data for model generation. After processing and quality control (QC), 665 models were successfully derived from 637 unique patients spanning 25 tumor types. This sample size was not predetermined by statistical calculations, but rather determined based on feasibility, model success rates, and the goal of improving cancer model diversity in preclinical research.

Data exclusions

A total of 22 models (5%) were excluded from downstream analyses due to low tumor purity. These models failed to meet the quality control threshold for epigenetic and transcriptomic fidelity assessments, as low tumor purity can lead to misleading genomic and transcriptomic signatures. Consequently, these samples were not included in the final dataset of 643 prioritized HCMI models used for further analyses.

Replication

Replication of experimental observations in this study was achieved through cohort-level analyses and independent validation across multiple data modalities. Genomic, transcriptomic, and epigenetic analyses were performed across large cohorts of independently derived patient–model pairs (e.g., n = 421 tumor–model pairs for genomic analyses and n = 297 pairs for transcriptomic analyses), enabling reproducibility through population-scale consistency. Key findings were validated across multiple independent computational methods (e.g., Celligner, OncoMatch, Euclidean distance, latent transcription factor distance, and classification-based approaches), ensuring robustness across analytical frameworks.

For experimental assays, measurements were performed on independently derived biological samples (distinct patient-derived models) and across independent sequencing experiments (e.g., WGS, WES, RNA-seq, and DNA methylation profiling) generated at multiple centers. For single-nucleus RNA sequencing analyses, independent tumor–model pairs (n = 16) were profiled to confirm reproducibility of observed cellular states. Technical replicates were not systematically performed, as analyses were conducted on independently derived biological samples across a large cohort.

Randomization

Randomization was not applicable to this study as patient-derived models were generated based on tissue availability and quality control metrics, rather than through an experimental grouping approach.

Blinding

Blinding was not performed during data analysis, as this study did not involve experimental group allocation or investigator-driven assignment of samples. All analyses were conducted on de-identified datasets (using ID3 identifiers) generated through standardized pipelines across multiple centers.

Reporting for specific materials, systems and methods

We require information from authors about some types of materials, experimental systems and methods used in many studies. Here, indicate whether each material, system or method listed is relevant to your study. If you are not sure if a list item applies to your research, read the appropriate section before selecting a response.

Materials & experimental systems

n/a	Involved in the study
☒	☐ Antibodies
☐	☒ Eukaryotic cell lines
☒	☐ Palaeontology and archaeology
☒	☐ Animals and other organisms
☐	☒ Clinical data
☒	☐ Dual use research of concern
☒	☐ Plants

Methods

n/a	Involved in the study
☒	☐ ChIP-seq
☒	☐ Flow cytometry
☒	☐ MRI-based neuroimaging

Eukaryotic cell lines

Policy information about cell lines and Sex and Gender in Research

Cell line source(s)

The HCMI models were generated from patient-derived tumor samples collected under institutional review board (IRB)-approved protocols. Tissue samples were obtained from the NCI's Cancer Model Development Centers (CMDCs) and processed following standardized methodologies.

Authentication	All models underwent short tandem repeat (STR) profiling using the PowerPlex® 18D kit (Promega) to confirm the tumor origin and ensure sample identity. Cross-contamination was assessed using cytochrome oxidase C1 (CO1) barcoding PCR assays.
Mycoplasma contamination	Each cell line was screened for mycoplasma contamination using the ATCC Mycoplasma Detection Kit (Catalog No. 30-1012K) before expansion and cryopreservation.
Commonly misidentified lines (See ICLAC register)	No commonly misidentified cell lines (as listed in the ICLAC Register) were used in this study. All models were patient-derived and authenticated prior to inclusion in downstream analyses.

Clinical data

Policy information about clinical studies

All manuscripts should comply with the ICMJE guidelines for publication of clinical research and a completed CONSORT checklist must be included with all submissions.

Clinical trial registration	This study is not a clinical trial and was not registered under a clinical trial database. Instead, it is an observational study focused on patient-derived cancer model generation and molecular characterization.
Study protocol	Institutional Review Board (IRB) approvals were obtained at each participating Cancer Model Development Center (CMDC) before patient enrollment and sample collection. Patient recruitment, consent, and data collection followed the ethical guidelines outlined in the HCMI consent template (https://www.cancer.gov/ccg/research/functional-genomics/hcmi/using-hcmi#informed-consent-template-for-hcmi).
Data collection	Clinical data collection for the HCMI study followed a structured approach to ensure standardization, reproducibility, and compliance with ethical guidelines. Patient data were collected using cancer-specific Case Report Forms (CRFs) developed by the HCMI Clinical Data Working Groups, which included oncologists, pathologists, and bioinformatics experts. These forms captured key patient demographics, tumor characteristics, treatment history, and clinical outcomes. All clinical variables were encoded as Common Data Elements (CDEs) and registered in the NCI Cancer Data Standards Registry (caDSR) to ensure consistency.
Outcomes	Clinical outcomes in the HCMI study were systematically collected to support translational research. Key outcome variables included the last day of follow-up, vital status, and overall survival (OS) for deceased patients, ensuring comprehensive survival analysis. Additionally, treatment histories and timelines were recorded.

Plants

Seed stocks	-
Novel plant genotypes	-
Authentication	-
