# Supplementary material for: A compendium of next-generation patient-derived models for diverse cancers
Source: Nature. 2026 Aug 5;657(8132):775–88. doi: 10.1038/s41586-026-10806-y (PMC13581597; doi:10.1038/s41586-026-10806-y)
Supplement: Supplementary file 3 [file 41586_2026_10806_MOESM4_ESM.pdf]

nature portfolio

Peer Review File

A Compendium of Next-Generation Patient-Derived Models for Diverse Cancers

Corresponding Author: Dr Jesse Boehm

Any redactions in this file are there to maintain patient confidentiality, the confidentiality of unpublished data, or to remove third-party material.

This file contains all reviewer reports in order by version, followed by all author rebuttals in order by version.

Version 0:

Reviewer comments:

Referee #1

(Remarks to the Author)
The authors of this manuscript report the successful establishment of 665 next-generation in vitro tumor cell cultures. Following the development of large-scale cancer genomics databases such as TCGA and DepMap, this study provides a valuable resource of diverse tumor organoid and new 2D culture lines. Through collaborative efforts, the authors conducted genomic profiling, gene expression analyses, and single-nucleus RNA sequencing (of a small subset) on the cultured cell/organoid lines and their matched tumor samples. The manuscript does not shed much light on new biology, yet establishes a potentially invaluable resource for the cancer research community. To achieve its potential, the authors have to strongly improve data and code availability, method reporting, and provide detailed user instructions to navigate this highly complex dataset.

Below are specific comments:

To enhance the user experience and the impact of this resource, the authors should provide a detailed instruction manual that references for each tumor type / each established model all the available datasets, the used analysis code, corresponding SOPs, regulatory information & restrictions (MTA, controlled access data procedures). Currently, these data points seem to spread across many different portals. Many parts were not yet deposited / accessible or generally retrievable within a reasonable effort during this review.

Data availability: most of the provided links do not work or reference only a subset of the cases that are reported in the manuscript (eg https://portal.gdc.cancer.gov/projects/HCMI-CMDC). This has to be corrected before submitting a revised version of the manuscript; currently, data is not available for proper review.

Code availability: The current manuscript only seems to point to the source code of some used functions but not (any) analysis code. The computational method section is convoluted (different centers use different pipelines) and not reproducible, the authors have to provide the actually used code to enable usability and reproducibility.

The used concordance/divergence thresholds (figs 2 and 3) should be better retrievable in the method section. The authors should better justify the selection of the used thresholds and demonstrate that the downstream interpretation is not overly sensitive to the chosen thresholds. Along the same lines:

The manuscript text frequently references “Methods”, this is a 20+ page part in the manuscript, so the corresponding exact location can not easily be identified. The authors should improve the method of reporting and use sections and subsections to point the reader to exact locations.

The QC process involved imaging H&E-stained sections of frozen or FFPE blocks. Are these images available? Morphological and histological features are crucial for assessing the similarity between tumors and cultured cells/organoids.

Since different centers contributed to sample collection and in vitro model establishment, can the authors summarize how

variations in protocols and culture conditions may have influenced success rates?

SNV calling was performed using four different methods (some on WGS, others on WES) and then compared. How comparable are the results? It would be more appropriate to compare datasets that underwent the same processing and computational pipelines.

Similar to the last point, was copy number calling and structural variant (SV) analysis performed using uniform pipelines across all samples?

Figure 2b presents an informative tumor/model pairing analysis but lacks negative controls. How much concordance is observed when random pairing is performed within the same tissue type? How much across tumor types?

Figure 2d indicates that some tumor types are polyploid (some models exceeding >4n). Do these cultured cells maintain a 3-4n state during division, or is there an active conversion from 2n to higher ploidy states?

In Figure 2e, it would be useful to highlight the most prevalent mutations (missense/nonsense) in the cohort and cultured models. Additionally, are these models typically driven by a single driver mutation, or do they exhibit polyclonality with multiple driver mutations?

The manuscript does not provide details on how samples were processed and prepared for snRNA-seq. These methodological aspects should be described.

The result section focuses on HCM-BROD-0613-C71 in the context of Figure 6. Does this culture model indeed remain resistant to TMZ therapy in vitro?

Minor points:

https://github.com/beroukhim-lab/hcmi_copy_number_consensus/: empty readme file

Page 7 lines 15-17: where is this result reported?

Page 7 line 40: in the text, it mentioned that fig 2g compares models after a year of passaging with the originating tumor. This was not clear to me from the figure and its corresponding legend. The passage status of the models in the figure 2 and 3 comparisons should be clarified.

Page 13 line 6: this adds little value, should either be specified here, in the supplemental text or removed.

Page 14 lines 27-28: it is unclear to me why the short OS suggests that MYCN amplification is a causative driver. This should be rephrased or supported by functional evidence.

Page 16 line 1-2: the observance of resistance signatures does not suffice to indicate that these models (that lack immune cells) can be utilized to study immunotherapy. This should be rephrased or supported by drug testing data.

(Remarks on code availability)

Code availability: The current manuscript only seems to point to the source code of some used functions but not (any) analysis code. The computational method section is convoluted (different centers use different pipelines) and not reproducible, the authors have to provide the actually used code to enable usability and reproducibility.

Referee #2

(Remarks to the Author)
I co-reviewed this manuscript with one of the reviewers who provided the listed reports.

(Remarks on code availability)

Referee #3

(Remarks to the Author)
A report from the Human Cancer Models Initiative presents a large collection of patient-derived tumor models, each provided with rich clinical and multi-omic information. Deep molecular analysis of the models shows that patient-derived models largely conserve the molecular characteristics of the original tumors, thereby providing high-fidelity and accessible tools for preclinical cancer research. The molecular concordance of models and the parental tumors is consistent with prior studies on patient-derived organoids, and the current study, together with accompanying papers, expands such knowledge to the multi-cancer level. Nonetheless, the high degree of the molecular conservation between patient-derived models and tumors has been repeatedly reported, and the readers may want to see some new and exciting insights beyond a series of descriptive observations. Below are my specific comments.

Major
1. Variable media formulations and culture formats are used depending on the facility. While the genomic profile is not expected to be affected by such variability, transcriptome and perhaps epigenetic status may profoundly be affected by the culture condition or institute location, as is evident from the GBM data. Besides GBM models are there any molecular bias due to the culture batch?

2. Related to the comment above, the authors show that GBM models cultured in different media (NSA vs CM) show distinct levels of neuronal-to-mesenchymal transition. Which component is actually preventing or driving this transition? It looks as if CM is not at all a suitable medium for GBM culture.

3. The section -Translational utility of HCMI models- only infers therapy resistance or sensitivity based on genomics, and whether the models actually have resistance to the pertinent drugs is unclear. The GBM part is especially correlative, and the authors should show whether the models with MMR or TMZ resistance signature are really resistance to TMZ compared to those without using a panel of GBM models.
Also, the paragraph describing GBM with MYCN ecDNA amplification is n = 1 data, and it is difficult to tell whether the MYCN ecDNA amplification contributed to the poor prognosis as well as clinical aggressiveness. The patient only lived 78 days after sampling, but we cannot judge whether this is short or not, as the tumor may have already been lethally large at the time of sampling. To formally show the aggressiveness of the tumor model, the authors should at least xenotransplant the MYCN ecDNA-amplified GBM model and see whether it's highly invasive and propagative or not.

Minor
1. Please show the success/failure rate for each tumor type. Although organoid derivation was done at multiple facilities using differing strategies, the take rate is an important piece of information.

2. Were the patients and tumor types selected base on some criteria, or simply randomly recruited? I just wondered, as some major tumor types, such as esophageal squamous cell carcinoma, lung squamous cell carcinoma, hepatocellular carcinoma and prostate cancer are not represented in the cohort.

3. Fig 6d and Extended Data Fig. 9f show that some tumor models were derived from patients with Lynch syndrome. How was the MSI status of these tumors? Also, why does the number of cases differ between the germline data and somatic data? Weren't second hit mutations in MMR genes identified in the remaining cases?

4. Fig. 4b and Extended Data Table 1 shows that there are 2 models of small intestinal adenocarcinoma, but n = 3 in the main text (page 10 line 15). Please correct.

5. The number of TCGA samples in Extended Data Fig. 5b is very small in many cancer types compared to the original TCGA reports. For example, TCGA sequenced more than 200 LUADs, but only 2 are used here. Why are there so few cases?

(Remarks on code availability)

Referee #4

(Remarks to the Author)
Major points
● Incredible collaborative effort – the creation of 665 new models is staggering and should be commended! Particularly 153 models of rare cancers and 71 non-european populations - wow!
● The efforts to make these "distributable" are wonderful
● The description of the genetic ancestry analysis on lines 40-42 of p5 is a bit clunky and makes it sound incorrectly that each model comes from a single population. Please rephrase. Figure 1c gives a similar impression, since each model appears to be categorized to a single ancestry, whereas each model includes various contributions of each ancestry. It would also be helpful if the authors could provide a little more context as to thresholds used to determine ancestry based on genetic analysis.
● The finding of culture media influencing mesenchymal state is important and significant but was however based on an n=3 for the CM condition. In other tumor types were there any culture-related differences? If authors shift the existing models into CM media, would this shift cells to a mesenchymal state?
o It would also be good to know if these mesenchymal states are reversible, for example can they regain the epithelial state when media is shifted?
● All data availability links should be verified activated at publication time, most of the links do not yet work. For example, I was not able to evaluate this tool because it has not been shared with me: HCMI Explorer Suite (https://appshare.cancer.gov/connect/#/apps/6c6f4b6c-8e55-4410-a49f-dbac2eebeba5) The link in the methods here did not work: https://github.com/D-Harouni/HCMI_Explorer_Suite)
● The title says "organoid models" but the HCMI also includes a proportion of 2D models. Consider removing organoid from the title or adding "cell line" to the title? Also, were the discordant models enriched for organoids or cell lines? In other words, was there any difference in model fidelity based on the 2D vs 3D culture?
● Are organoid models superior to the 2D models? This is the group that can answer this important question. If so why? If not, why not?

Other specific points

● There seem to be a lot of "N/A" values in Extended Data 1, HCMI model sequencing and align. Some added context for why there are so many missing values would be helpful.
● The legend for Supp Figure 3 should define the population abbreviations used in the labels in the figure. It also appears that the samples are ordered by increasing AFR ancestry, rather than decreasing as stated in the legend, if I understand the figure correctly.
● Were germline polymorphisms used to ensure that the model and tumor were derived from the same individual? This could have been clarified in the text and used to further support that the more divergent models were in fact still from the same individual.
● Figure 5g – it is not clear to me whether the data shown in the right two panels (078 and 089) is from the model-only, tumor-only, or both
● Line 15-17 on p13 appears incorrect: "Overall the HCMI collection includes models that were derived from patients with 491 treatment events before model generation (naive models) (Extended Data Fig. 9a) and 533 treatment events post model generation (post-treatment models)." If treatment events occurred prior to model generation, then these models should be post-treatment, not naïve.
● For SNV/indel calling- please clarify how variants were "merged" from the three callers. Were they required to be called by 1,2,or 3 of the callers?
● A summary of the tumor stage of the tumor used to derive each model would have been helpful. I did not see this commented on in the text – are most of these tumors early stage primaries? Some seem to be metastatic and post-progression, so a more clear summary of this in Fig 1 would be helpful. Does this differ from CCLE in a complementary way?
● In figure 2 the authors do a good job of comparing concordance between tumor and model. Particularly they show that majorly across models there is a significant concordance in mutations and signatures. However a couple of things would be helpful to the readers:
o Are the VAF of mutations (SNV/INDELs/CNS/SV etc) in models the same as tumors, particularly for driver/tumor supressors alterations.
o For drivers/tumor suppressors mutations that are only seen either in models or in tumors have a different VAF. Meaning if a very low VAF of drivers seen in either tumor or model affecting their detection ?
o Also are there any clinical patterns that correlate with discordance? Aka what is driving discordance? Is it genetically defined or more technical issues that could cause the discordance? Note: the reviewer has taken into account the suggestions that low tumor purity and/or FFPE artifacts could be reasons
o Extended Data Fig 2f needs to be cited in the main text (p6 line 31-32)
● It would be helpful overall to include FFPE corrections where possible for FFPE-derived data. For example was a FFPE panel of normals used where possible reduce FFPE artifacts? Correction of copy number calls in a similar way can be performed.
● The authors should expand upon how they determine mutational signature concordance to give more clarity. Are they only assigning concordance on the basis of presence or absence of mutational signature or on the basis of fraction of mutations with each signature? Or are they generating a per-sample based signature and then assessing cosine similarity in comparing tumor/model pairs?

(Remarks on code availability)
The link does not work: https://github.com/D-Harouni/HCMI_Explorer_Suite

Referee #5

(Remarks to the Author)
I co-reviewed this manuscript with one of the reviewers who provided the listed reports.

(Remarks on code availability)

Version 2:

Reviewer comments:

Referee #1

(Remarks to the Author)
We appreciate the authors' detailed responses and the subsequent improvements to the manuscript. The enhanced documentation significantly strengthens the work's reusability within the research community, and we are pleased with the clarifications provided regarding the cancer biology aspects of the study. That said, we have a few remaining points that require further attention:

-Code: the linked repository (https://github.com/NCICCGPO/HCMI-Flagship-Manuscript) is not available and cannot be peer reviewed.

-The mentioned HCMI explorer is inaccessible

(Remarks on code availability)
it is not accessible and should still be reviewed

Referee #2

(Remarks to the Author)
I co-reviewed this manuscript with one of the reviewers who provided the listed reports.

(Remarks on code availability)

Referee #3

(Remarks to the Author)
The authors have addressed all raised concerns, and I have no further comments.

(Remarks on code availability)

Referee #4

(Remarks to the Author)
We thank the authors for their thoughtful responses and new data and clarifications added. They have addressed all of our comments.

(Remarks on code availability)

Referee #5

(Remarks to the Author)
I co-reviewed this manuscript with one of the reviewers who provided the listed reports.

(Remarks on code availability)

Open Access This Peer Review File is licensed under a Creative Commons Attribution 4.0 International License, which permits use, sharing, adaptation, distribution and reproduction in any medium or format, as long as you give appropriate credit to the original author(s) and the source, provide a link to the Creative Commons license, and indicate if changes were

made.
In cases where reviewers are anonymous, credit should be given to 'Anonymous Referee' and the source.
The images or other third party material in this Peer Review File are included in the article's Creative Commons license, unless indicated otherwise in a credit line to the material. If material is not included in the article's Creative Commons license and your intended use is not permitted by statutory regulation or exceeds the permitted use, you will need to obtain permission directly from the copyright holder.
To view a copy of this license, visit https://creativecommons.org/licenses/by/4.0/

26 November 2025

Response to Shared Comments of the Referees
Manuscript numbers: 2025-01-02429, 2025-01-02750, 2025-02-03572

Dear referees,

Thank you for your prior consideration of the three manuscripts from the HCMI network, the Sanger Institute, and the Broad Institute, each reporting large-scale efforts to derive next-generation (NextGen) cancer models and/or to generate multi-omics and CRISPR functional data for public dissemination. We appreciate your insightful feedback, which raised several highly addressable common themes across the three manuscripts. Below, we provide our joint high-level responses and the coordinated updates implemented across the manuscripts to address these shared comments. Full point-by-point responses are provided in the accompanying revised versions of each manuscript. Based on these comprehensive revisions, we respectfully request your reconsideration of our revised manuscripts, which we feel are now highly responsive to your comments.

● Referees #3 and #4/5 commented on the relative merits of 2D and 3D culture systems, questioning whether one is generally superior. Based on our observations, both have value and are complementary, with advantages depending on the intended application. As demonstrated in the HCMI and Broad studies, integrating both CCLE (2D) and HCMI (3D) models substantially improved coverage of TCGA tumors with high-fidelity matches, underscoring the combined value of 2D and 3D resources in representing tumor diversity. This includes tumor types such as esophageal adenocarcinoma, prostate and GBM, which are absent or poorly represented in current 2D collections. The availability of a matched tumor sample for genomic comparison, germline DNA for accurate calling of somatic variation, and linked patient clinical data are additional advantages of next-generation models compared to most 2D collections.

In addition, while 2D systems generally allow rapid and cost-efficient profiling, certain tumor features are uniquely represented in 3D systems, including preserving lineage-specific tumor transcriptional programs (Broad manuscript) and clonal heterogeneity (Sanger manuscript). Importantly, both the HCMI and Broad manuscripts show that the primary driver of transcriptional divergence among the GBM models is the composition of the culture medium, rather than growth dimensionality. Additionally, we provide an in-depth analysis and data of the dependencies differences between 2D and 3D models including validation with a secondary screen (Broad manuscript). In the revised Broad and Sanger manuscripts, we have more explicitly emphasized these insights and strengthened the comparative analysis of 2D and 3D systems to better guide model selection for different use cases.

● Referees #1/2 and #3 requested clarification on the success rates of model derivation in the HCMI and Sanger studies. In the Sanger manuscript, we further contextualized these success rates by detailing common reasons for failure, such as generation of ‘short-term cultures’, and how specific protocol variations, including media formulations and tissue handling approaches, affected outgrowth. In parallel, the HCMI manuscript expanded the analysis to include 168 failed tumor growth attempts, enabling exploration of intrinsic biological features associated with

successful versus unsuccessful model derivation. Together, these complementary analyses deepen the understanding of both technical and biological determinants of model derivation success across diverse tumor types.

● Referees #1/2 and #3 expressed concerns about the limited single-gene knockout validation of genome-wide CRISPR screen findings in the Sanger and Broad studies. We agree that validation is essential to assess the biological and translational relevance of these findings. These targeted validations have been incorporated into the revised manuscripts. However, the central aim of these manuscripts is to present broadly accessible model and data resources that enable users to discover cancer dependencies at scale using next-generation models. To remain within this scope, we focused validation efforts on a limited subset of the most compelling and biologically relevant findings as proof-of-concept demonstrations, while clearly positioning broader systematic validation as beyond the remit of these resource manuscripts and more appropriate for future mechanistic follow-up studies.

● All referees requested deeper investigation and clearer explanation of the transcriptional changes observed in GBM models under different culture conditions, as reported in both the HCMI and Broad submissions. The available evidence from both studies strongly supports the role of culture medium composition as a key driver of neural-to-mesenchymal transcriptional transitions, rather than inherent model instability. Importantly, within the HCMI study, we have now included a culture medium modulation experiment to directly assess the effect on these transcriptional programs. These results have been incorporated into the revised HCMI manuscript and used to further support the mechanistic interpretation raised by the reviewers.

In addition, to enhance data accessibility and provide a unified overview of the models and resources included across the three studies, we have leveraged the HCMI Explorer Suite, an interactive Shiny-based exploratory platform developed by the HCMI team. While the platform does not enable direct browsing of all raw datasets, it will provide an integrative view indicating whether a given model appears in one or more of the three efforts, along with annotations of data modalities, availability, and links to the respective portals. Furthermore, we have compiled summary tables in the supplementary materials that harmonize and annotate shared models across the three studies. In addition, we included all the HCMI RNAseq expression data in an app hosted in the DepMap Broad portal that allows matching models to tumor or PDX data. Upon publication, the portals of all three resources will be fully accessible, ensuring open access to raw and processed data. Responses to the remaining detailed comments are provided in the individual letters from each study group.

We believe that the proposed revisions will address referees' concerns and strengthen the collective message of our work, underscoring the significant resource value of these three coordinated studies. Thank you in advance for considering our revised manuscripts.

On behalf of the authors from the Sanger Institute, HCMI network and Broad Institute.

KOCH INSTITUTE
for Integrative Cancer Research at MIT
Massachusetts Institute of Technology ● ki.mit.edu
77 Massachusetts Avenue ● 76-453 ● Cambridge, MA 02139 ● boehm@mit.edu

Jesse S. Boehm, PhD
Principal Investigator
Massachusetts Institute of Technology

Response to Referees

Manuscript Revised Title: A Compendium of Next-Generation Patient-Derived Models for Diverse Cancer Types

Manuscript number: 2025-01-02429

We thank the referees for their thoughtful and constructive feedback. We greatly appreciated the opportunity to revise our manuscript *"A Compendium of Organoid Models for Diverse Cancer Types"* to address comments related to clarity, scientific rigor, translational relevance, and methodological transparency. Below, we summarize the major revisions and additions made to the manuscript, followed by a detailed point-by-point response.

In this revised version, we have incorporated new functional and computational analyses that directly strengthen the translational and biological utility of the resource.

An important focus of our revision was to **improve the accessibility of the data, the code, and the description of our methodological framework**. We highlight the publicly accessible instruction portal (HCMI Page), which now serves as the primary access point for the resource, providing an overview of the HCMI program, data portals and model access pages. All model-level data are now available through the GDC Data Portal, ensuring fully open access to the underlying genomic and clinical datasets. All processed and raw datasets have been organized and validated for public release through a curated GDC Publication Page, which will provide permanent accession links to every referenced dataset and model upon completion of the NCI data release process (representative snapshots of the publication page development interface are provided in the rebuttal appendix). In parallel, we established a centralized HCMI GitHub repository that integrates all analysis pipelines, figure-generation scripts, and software resources described in the manuscript. The repository is currently private and will be made public at the time of publication. Furthermore, a total of 857 digitized H&E whole-slide images, including 198 from unsuccessful derivations (see below), have been curated and made publicly accessible to support digital pathology research and model validation. In terms of the **enhancements to the methodological framework**, the Methods section has been reorganized into clearly defined subsections aligned with each analytical module, including genome, transcriptome, and model-fidelity analyses.

Another major addition to the revised manuscript, is that we have supplemented the already extensive genomic and molecular data in the original version of the manuscript with an additional resource - **what we believe is the largest "dataset of model development failure" released publicly.** With this **expanded dataset we were able to perform new genomic analysis of 168 tumors that did not yield successful models** in addition to the 665 successfully established

models, enabling an evaluation of factors governing model establishment across cancer types. These analyses indicate that model success depends on tumor-intrinsic biology, with successful tumors favoring differentiated, stem-like programs over proliferative and DNA-repair states. The molecular data overview summarizing the new data has been incorporated into the revised extended data, and a new supplementary figure (**Supplementary Fig. 3**) has been added to detail the comparative results.

In response to the referees' interest in the **modulation of media-associated transcriptional states**, we have performed new experiments using glioblastoma (GBM) as a model lineage. In our prior version of the manuscript, transcriptional profiling suggested that serum-containing media strongly drive a mesenchymal-like transition, whereas serum-free neural stem–adapted (NSA) formulations preserve glial and progenitor-like features. To test that proposition and to evaluate the plasticity of those states, we performed a media-switching experiment in which both immunofluorescence and RNA sequencing demonstrated that serum-containing media drive a mesenchymal shift. Those results underscored the modulatory effect of culture conditions on lineage fidelity. These findings have been incorporated into the revised manuscript (**Fig. 3** and **Supplementary Fig. 5**).

In response to the referees' request for **functional validation of temozolomide (TMZ) resistance**, we also performed viability assays across a representative panel of GBM models, including treatment-naïve and pretreated lines with or without SBS11 signatures. Our new results (**Supplementary Fig. 7**) provide direct functional evidence supporting genomic predictions of resistance.

Finally, we have removed the word "organoid" from the title of the manuscript in response to a referee's comment that 16% of the included models were not organoids. The new title is "***A Compendium of Next-Generation Patient-Derived Models for Diverse Cancer Types.***"

Collectively, these revisions provide new experimental validation, expand biological insight, and ensure complete methodological transparency. We believe that the revised manuscript now fully addresses the referees' concerns and significantly strengthens the scientific and translational impact of the HCMI resource.

Please find below our point-by-point responses

Referees #1 and #2

"The authors of this manuscript report the successful establishment of 665 next-generation in vitro tumor cell cultures. Following the development of large-scale cancer genomics databases such as TCGA and DepMap, this study provides a valuable resource of diverse tumor organoid and new 2D culture lines."

We thank the reviewers for this recognition of the value of the resource.

"The manuscript does not shed much light on new biology yet establishes a potentially invaluable resource for the cancer research community. To achieve its potential, the authors have to strongly improve data and code availability, method reporting, and provide detailed user instructions to navigate this highly complex datasets."

We agree that additional biological insights and strengthening data and code availability would be helpful. Please see more details below on how we have addressed this suggestion.

Major comments:

1- *To enhance the user experience and the impact of this resource, the authors should provide a detailed instruction manual that references for each tumor type / each established model all the available datasets, the used analysis code, corresponding SOPs, regulatory information & restrictions (MTA, controlled access data procedures). Currently, these data points seem to spread across many different portals. Many parts were not yet deposited / accessible or generally retrievable within a reasonable effort during this review.*

We agree that usability and accessibility are critical to maximizing the impact of the HCMI resource. Toward that end, we have made a comprehensive series of improvements:

1. We call attention to the publicly accessible instruction portal (HCMI page), which now serves as the central entry point for the resource. This portal provides a comprehensive overview of HCMI and the Cancer Model Development Centers (CMDC) workflow. Further, it provides direct navigation to all linked data portals, and instructions about accessing the models, including protocols for model expansion hosted by ATCC.
2. We developed a curated Genomic Data Commons (GDC) Publication Page, designed to provide a centralized and structured framework summarizing data availability of raw and processed data, sequencing modalities, and access tiers across the full cohort. Although this publication page has been created and approved by the NCI, it remains temporarily unavailable pending NCI release (representative snapshots of the publication page development interface are provided in the rebuttal appendix). In the meantime, all data are now fully accessible through the HCMI-CMDC project on the GDC portal.
3. We have updated **Supplementary Table 1** to include a systematically mapped HCMI model ID of each patient-derived model to its metadata, including tumor type, tissue of origin, and availability of omics datasets (WXS, WGS, RNA-seq, methylation).
4. For computational reproducibility, all analysis pipelines and scripts have been consolidated into a unified GitHub repository (HCMI GitHub page), providing a single access point for the analyses described in this study. The repository is currently maintained under restricted access for program coordination and will be made public upon manuscript acceptance.
5. We have now compiled all relevant standard operating procedures (SOPs) for tissue processing, model derivation and quality control in the Supplementary Materials and Methods. In addition, the HCMI page provides direct navigation to the ATCC HCMI models portal, where each model entry includes its corresponding experimental protocols.

6. We now provide additional clarity on the ATCC distribution system for HCMI models (**Supplementary Materials and Methods: HCMI distributor (ATCC)**) At the time of manuscript revision, all models are available for academic use through ATCC under non-commercial MTAs, with industry access governed by approval of commercial MTAs. To highlight model availability more clearly, we distinguish between two categories:
    a. Distribution-ready models: At the time of writing this manuscript, ATCC has expanded and released 316 HCMI models for distribution.
    b. HCMI "Unmanufactured Models" collection: The remainder of the HCMI models are also accessible through the HCMI Unmanufactured Models nomination system, which enables researchers to view associated omics and clinical metadata and nominate models for prioritized expansion and rapid distribution. This system prioritizes the HCMI models with the greatest community interest and ensures readiness as customers request particular models.

We believe these additions significantly improve data discoverability, integration, and transparency for end users.

2- *Data availability: most of the provided links do not work or reference only a subset of the cases that are reported in the manuscript (eg https://portal.gdc.cancer.gov/projects/HCMI-CMDC). This has to be corrected before submitting a revised version of the manuscript; currently, data is not available for proper review.*

To improve accessibility and streamline data retrieval by the community, we have included in the revised submission a curated publication page on the Genomic Data Commons (GDC) that consolidates data across sequencing modalities. It includes validated, functional links for all referenced datasets (representative snapshots of the publication page development interface are provided in the rebuttal appendix). The GDC portal and cBioPortal now host the complete successful model dataset described in the manuscript (665 models from 637 patients), ensuring that the full cohort is accessible.

The HCMI Searchable Catalog currently lists 588 models, aligned with NCI's phased release schedule, and will be updated to include the full set of 665 models upon completion of the ongoing NCI integration process. The HCMI Explorer Suite, which provides interactive visualization of clinical timelines is currently maintained under restricted review access and will be made public concurrently with publication. Representative screenshots and interface examples are included in **Supplementary Fig. 9**.

We have updated the Data Availability section [Article page 55, line 1-13] to clearly specify all access portals and their intended uses, ensuring that readers can readily navigate raw, processed, and interactive datasets as follows:

"To support data access, we maintain a publicly accessible instruction portal (HCMI Page at https://www.cancer.gov/ccg/research/functional-genomics/hcmi/using-hcmi), which serves as the central entry point for the resource. Molecular and genomic characterization data from this study are available through the HCMI-CMDC project on the NCI Genomic Data Commons (GDC) web

page (https://portal.gdc.cancer.gov/projects/HCMI-CMDC). Processed molecular datasets (mutation calls, copy-number profiles, clinical annotations) are available through cBioPortal (https://www.cbioportal.org/study/summary?id=pancan_hcmi_2025). Interactive model-level exploration is available through the HCMI Searchable Catalog (https://hcmi-searchable-catalog.nci.nih.gov) and the HCMI Explorer Suite (https://appshare.cancer.gov/content/b8c2812f-6ca5-492d-a9f4-b81107aed8d6/), which offer interactive visualization of clinical timelines and genomic annotations. HCMI patient-derived models are distributed through the ATCC HCMI collection (https://www.atcc.org/hcmi).”

***3-** Code availability: The current manuscript only seems to point to the source code of some used functions but not (any) analysis code. The computational method section is convoluted (different centers use different pipelines) and not reproducible, the authors have to provide the actually used code to enable usability and reproducibility.*

To ensure transparency and reproducibility of our analyses, we have organized all computational pipelines and analysis code under a unified github repository available at https://github.com/NCICCGPO/HCMI-Flagship-Manuscript. This repository contains the complete set of scripts used to generate the analyses and figures reported in the manuscript, covering all sequencing modalities and harmonized across contributing centers. The repository is currently private but will be made fully public at the time of publication.

We have also updated the Code Availability section, the revised text now reads [Article page 55, line 15-17]:

“All computational workflows, analysis pipelines and scripts used in this study are consolidated in a dedicated GitHub repository: https://github.com/NCICCGPO/HCMI-Flagship-Manuscript”

***4-** The used concordance/divergence thresholds (figs 2 and 3) should be better retrievable in the method section. The authors should better justify the selection of the used thresholds and demonstrate that the downstream interpretation is not overly sensitive to the chosen thresholds*

We thank the referee for emphasizing the need to clarify how concordance and divergence thresholds were selected. The thresholds used in the concordance and divergence analyses are now defined in the revised Methods section and referenced in the figure legends. Thresholds for LOH concordance (≥ 0.8), copy-number event detection, and transcriptomic similarity were determined empirically through benchmarking against unrelated tumor–model pairs and validation in comparable datasets.

For distance-based metrics (e.g., Celligner-derived transcriptomic concordance), thresholds were established using null distributions derived from pairwise distances between unrelated tumor-model pairs, with cutoffs set at FDR < 0.1 to balance sensitivity and specificity. Similar negative control strategies were used across other pipelines to calibrate thresholds specific to each modality.

To address the referee's concern regarding robustness, we performed a sensitivity analysis by varying thresholds across modalities and quantifying the downstream impact:

- For **LOH concordance** (or shared LOH fraction), setting different LOH shared fraction cutoffs for concordance shows that a cutoff of 0.8 is reasonable considering that differing cutoffs would not change the conclusion drastically unless set to an extreme value like 0.95 (Supp. Table LOH_cutoff_changes).

Shared LOH fraction cutoff	Concordant pair fraction
0.65	0.99742268
0.70	0.981958763
0.75	0.963917526
0.80	0.930412371
0.85	0.889175258
0.90	0.817010309
0.95	0.639175258

Table: Impact of varying LOH concordance thresholds on tumor–model concordance classification. *The table reports the proportion of tumor–model pairs classified as LOH-concordant under different shared-LOH fraction cutoffs (0.6, 0.7, 0.8, 0.9, and 0.95). Although extremely stringent thresholds (≥ 0.95) reduce the number of concordant pairs, all reasonable cutoffs (0.7–0.9) yield consistent patterns, supporting the robustness of the selected LOH concordance threshold (≥ 0.8).*

- For **Celligner-based transcriptomic concordance**, we further evaluated the impact of varying the (FDR) thresholds used to define outlier tumor–model pairs. Adjusting the FDR cutoff from 0.1 to 0.05 or 0.001 resulted in only minor changes in the proportion of outlier models—from approximately 24 % to 27 % and 37 %, respectively—confirming that the majority of models remain transcriptionally concordant with their parental tumors across a broad range of thresholds.

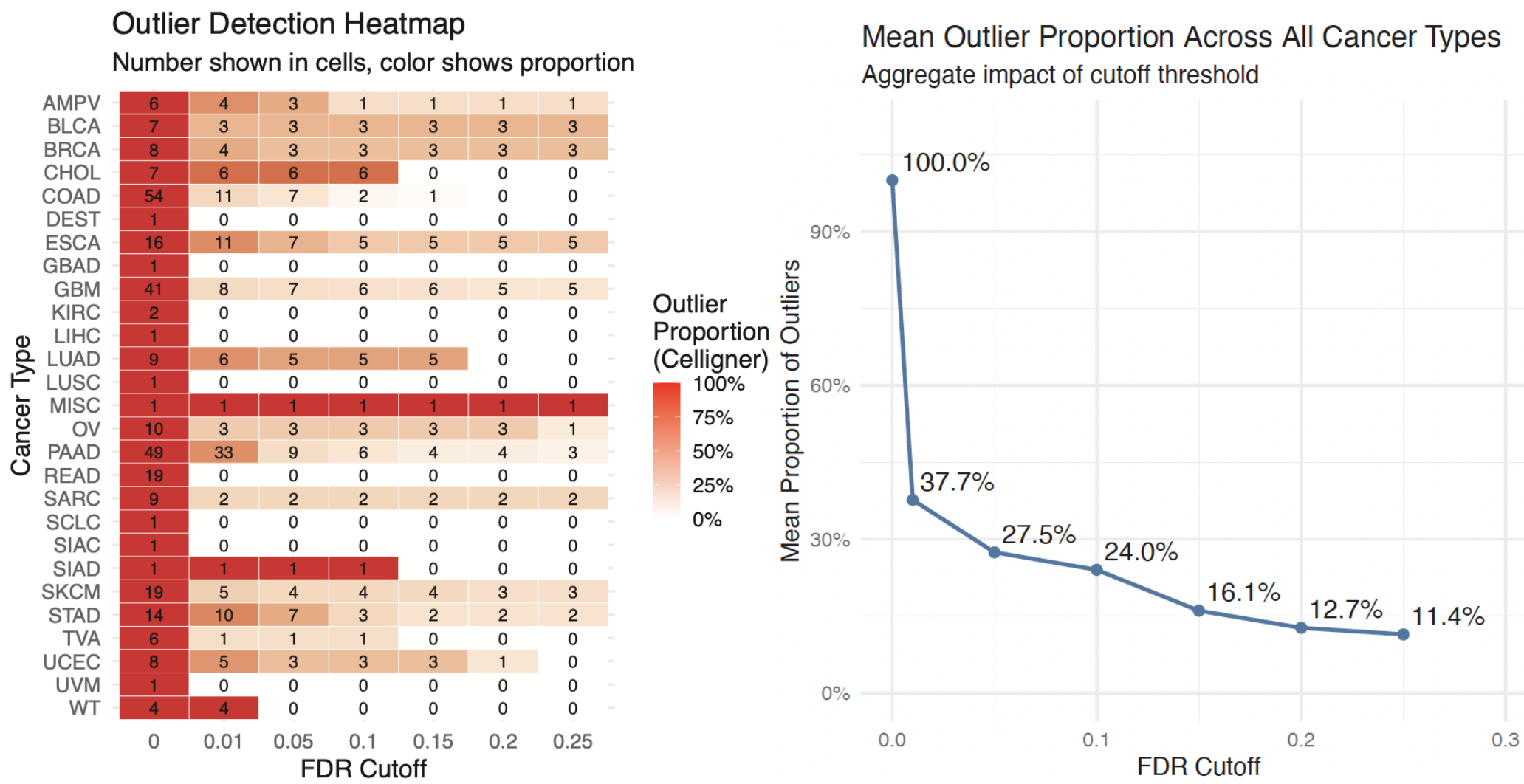

***Figure: Sensitivity analysis of distance-based transcriptomic concordance thresholds. (a)** Heatmap showing the number and proportion of tumor–model pairs classified as outliers across 20 tumor types over a range of Celligner FDR thresholds (0–0.25). Each cell displays both the number of outlier pairs (text) and their relative proportion (color). **(b)** Mean proportion of outlier tumor–model pairs across all cancer types as a function of the Celligner FDR cutoff. Although more stringent thresholds (e.g., FDR = 0.001) increase the outlier fraction, overall proportions remain low across tested cutoffs.*

Importantly, the media-associated transcriptional patterns observed in GBM (FM vs NSA) remained consistent under all tested cutoffs, supporting the overall robustness of the Celligner-derived conclusions.

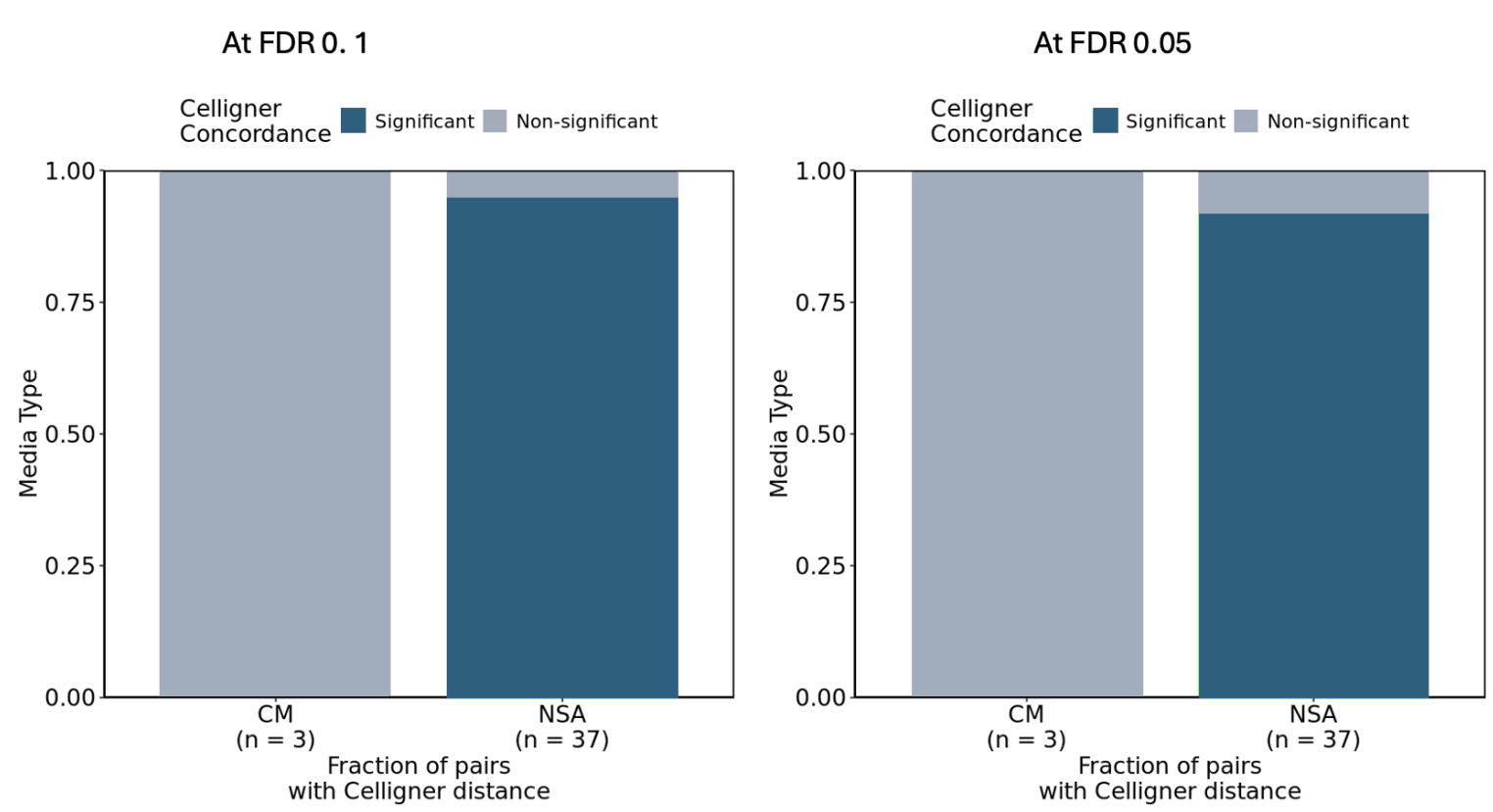

***Figure: Robustness of media-associated transcriptional patterns in GBM across concordance thresholds.** Concordance of media-associated transcriptional differences in GBM (FM vs. NSA) evaluated under two representative Celligner FDR thresholds (0.1 and 0.05). Barplots show the fraction of tumor–model pairs classified as significant versus non-significant for CM- and NSA-cultured models at each cutoff.*

Together, these analyses demonstrate that our downstream interpretations are not sensitive to the exact threshold choices, and that all selected parameters are grounded in empirical benchmarking and reproducibility considerations.

5- *The manuscript text frequently references "Methods", this is a 20+ page part in the manuscript, so the corresponding exact location can not easily be identified. The authors should improve the method of reporting and use sections and subsections to point the reader to exact locations.*

We appreciate this comment and fully agree that methodological clarity and navigability are essential to the utility of the manuscript. In the revised version, we have restructured the Methods section into clearly labeled subsections corresponding to major data types and analytical modules (e.g., "Genome Sequencing: Purity and Ploidy Estimation"). Each subsection title directly reflects the analysis performed, enabling readers to quickly identify the relevant methods. References to the Methods throughout the main text have been systematically updated to cite these specific section and subsection headers, ensuring that all methodological details can be located without ambiguity.

6- *The QC process involved imaging H&E-stained sections of frozen or FFPE blocks. Are these images available? Morphological and histological features are crucial for assessing the similarity between tumors and cultured cells/organoids.*

We agree that histopathological review is a critical component of tumor–model characterization. All available hematoxylin and eosin (H&E) whole-slide images (WSIs) generated in the context of this study have now been curated and are available through the GDC portal. We have incorporated model-level WSI availability into Supplementary Table 1 (HCMI Model Sequencing and Data Availability tab), enabling streamlined access to this information alongside other modality-level annotations.

A total of 857 digitized H&E WSIs are available, corresponding to 386 tumor samples and 89 adjacent normal tissues. These WSIs are now integrated into the model-level metadata. Additionally, 198 WSIs from 99 tumors that failed model generation are also available.

7- *Since different centers contributed to sample collection and in vitro model establishment, can the authors summarize how variations in protocols and culture conditions may have influenced success rates?*

To better understand factors underlying model derivation outcomes, we incorporated **a new genomic dataset of 168 failed derivation attempts** alongside the 665 successfully established models reported in this study (**Supplementary Table 1**). These failed attempts represent tumor tissues collected and processed across all five Centers for Cancer Model Development (CMDCs) using standardized, cancer-type–specific derivation protocols, harmonized media formulations, and consistent quality-control checkpoints. As protocols were identical within each center and diagnosis group, variation in model success is unlikely to reflect inter-center or technical inconsistencies but rather reflects tumor-intrinsic biology.

We consolidated CMDC documentation into four standardized categories of derivation failure:

- Primary fails – no tumor cell outgrowth observed after initial seeding.
- Slow proliferators – cultures with growth but unable to expand sustainably.
- Short-term cultures – initially expanding cultures that underwent sudden growth arrest or senescence.
- Outgrowth of normal cells – cultures overtaken by non-tumor stromal or epithelial cells.

These failure categories were tumor-type specific: for example, breast cancer cultures frequently failed due to fibroblast contamination and necrotic tissue; pancreatic tumors showed high stromal content and low tumor purity consistent with their desmoplastic microenvironment; and colorectal cancers were prone to senescence and differentiation-related arrest.

We have expanded the dataset and visualization to include the 168 failed model derivation attempts alongside the 665 successfully established models. These data are now integrated into **Extended Data Fig. 1**, which provides a comprehensive overview of successful and failed derivations and summarizes the genomic and molecular data availability for both groups.

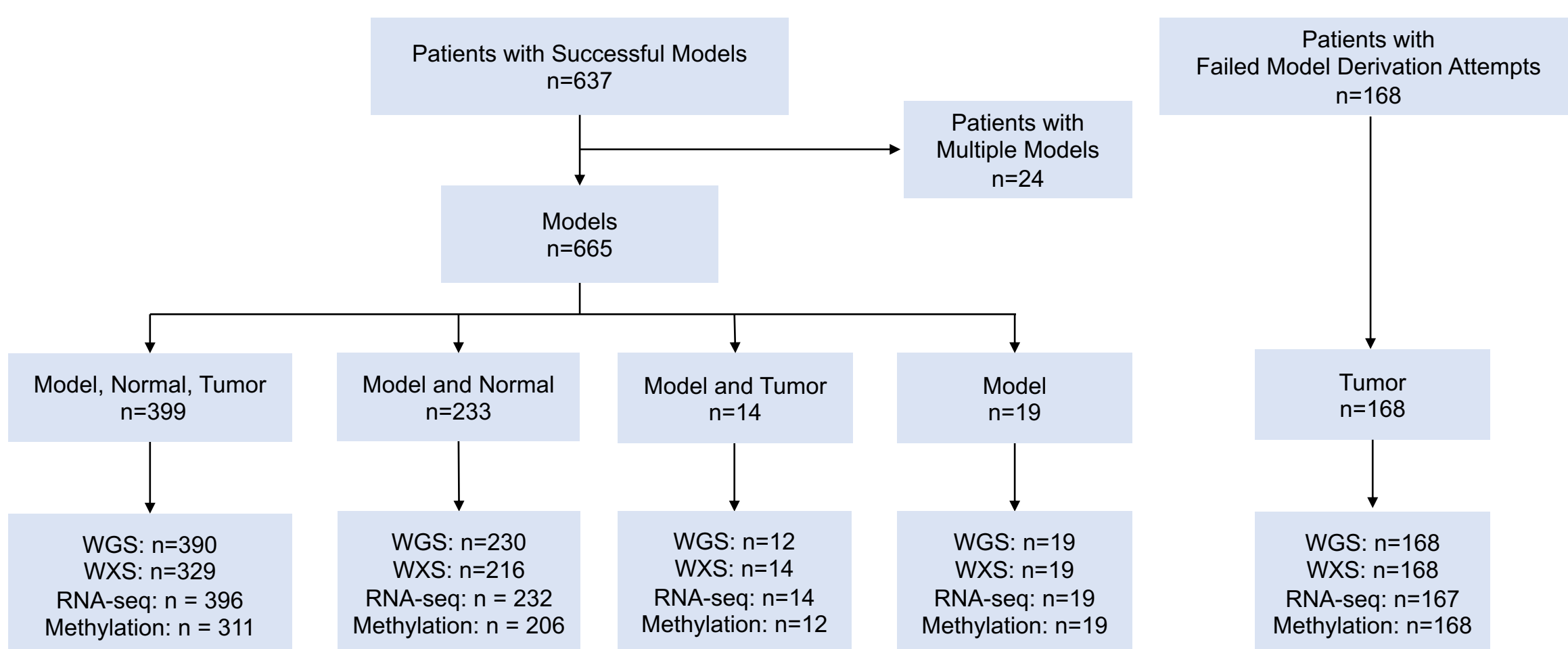

Extended Data Fig. 1: Consort diagram of HCMI model frequencies and molecular characterization. *The diagram summarizes sequencing data generated for the HCMI cohort (n = 665 models). Counts are shown for models with available tumor, normal, and model samples, including complete trios (n = 399), pairs of model and normal (n = 233), pairs of model and tumor (n = 14), and models without matched tumor or normal samples (n = 19). For each group, the number of samples profiled by WGS, WXS, RNA-seq, and DNA methylation is indicated. In addition, 168 failed derivation attempts are included, with sequencing data available for tumor tissues (WGS, WXS, RNA-seq, and methylation).*

To further investigate biological factors associated with model derivation outcomes, we compared molecular features between the 665 successfully established models and the 168 failed derivation attempts, focusing on the three largest tumor types in the cohort (colorectal, pancreatic, and esophageal adenocarcinomas). Although tumor purity did not show correlation with model success (**Supplementary Fig. 3 a,b**), gene set enrichment analysis (GSEA) of cancer cell state

signatures (CancerSEA; Yuan et al., 2019) revealed distinct enrichment patterns between failed and successful cases. We noticed that failed derivations were significantly enriched for DNA-repair and cell-cycle–associated states, whereas successful models exhibited lower representation of these programs, suggesting that aggressive proliferative or repair phenotypes within tumors may not translate into long-term culture adaptability (**Supplementary Fig. 3 c-f**)

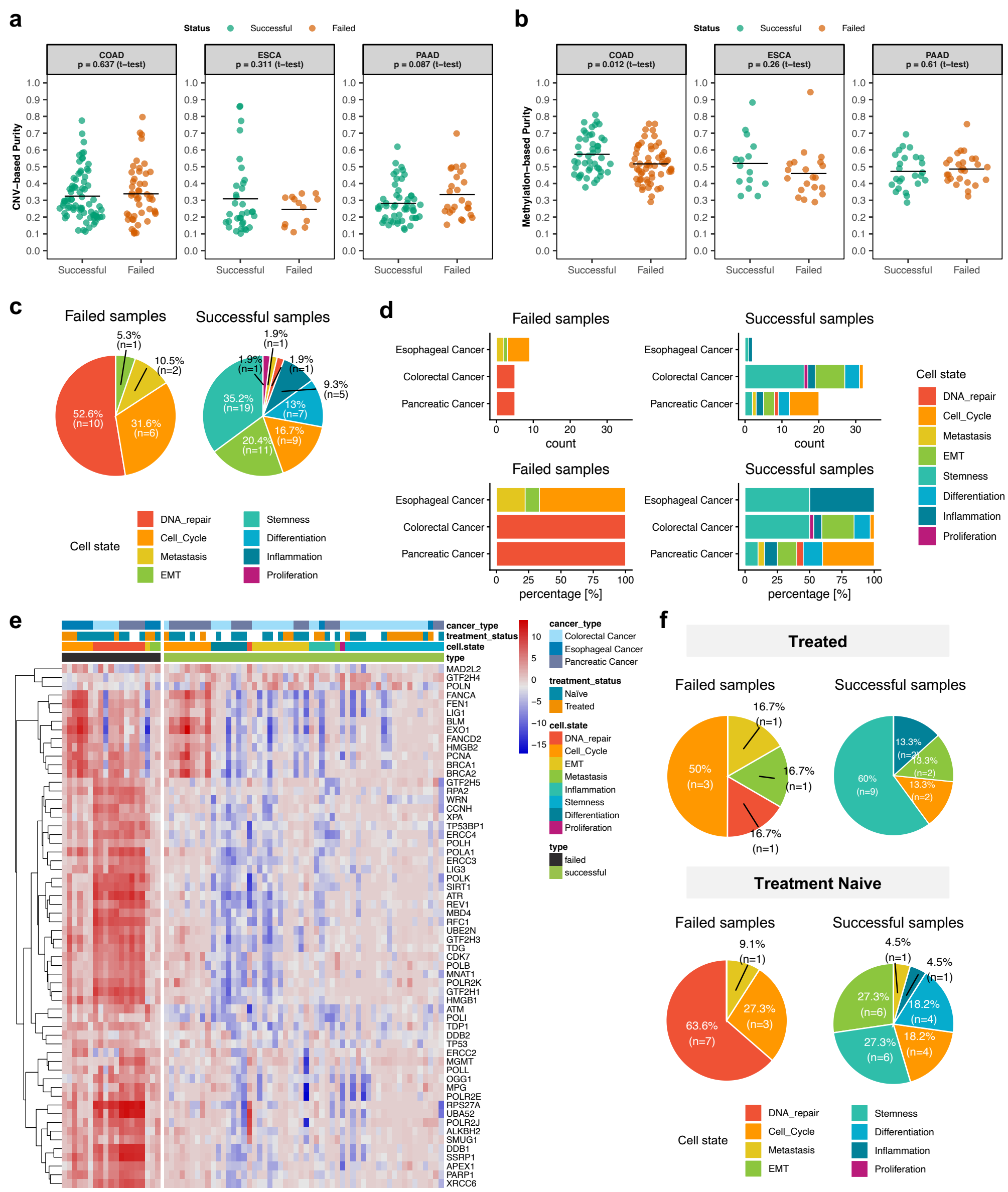

Supplementary Figure 3: Determinants of model establishment success. Comparison of tumor purity between successful and failed model derivations across colorectal (COAD), esophageal (ESCA), and pancreatic (PAAD) cancers. ***(a)*** *CNV-based purity estimates and* ***(b)*** *Methylation-based purity. P values were calculated using two-sided t-tests.* ***(c)*** *Cancer cell states (CancerSEA; Yuan et al., 2019) identified by GSEA among failed and successful tumors for colorectal, pancreatic, and esophageal cancers. The piecharts display the proportion (%) of each cancer cell state among failed (left) and successful tumors (right), respectively.* ***(d)*** *bar plots indicate the number of failed (left) and successful tumors (right) for each cancer type, with colors representing different cancer cell states. The bottom bar*

plots (left: failed tumors, right: successful tumors) show the percentage distribution of cell states for each cancer type. ***(e)*** *Heatmap displaying master regulator activities of genes (rows) annotated for DNA repair across failed and successful tumor samples (columns). Positive and negative gene activities were colored red and blue, respectively.* ***(f)*** *Pie charts showing cancer cell states in failed and successful samples for treated (top) and treatment-naïve cases (bottom), respectively.*

Together, these findings demonstrate that tumor-intrinsic biology, rather than protocol variation, is the major determinant of model derivation success. This dataset provides an important initial resource for the community to explore reasons that certain tumors fail to yield successful cellular models and provides rationale for other initiatives to characterize failures with the same rigor as successes. These insights have been incorporated into the revised manuscript [Article page 5, line 38 – page 6, line 3] and are summarized in **Extended Data Fig. 1** and **Supplementary Fig. 3**.

8- *SNV calling was performed using four different methods (some on WGS, others on WES) and then compared. How comparable are the results? It would be more appropriate to compare datasets that underwent the same processing and computational pipelines.*

We thank the referee for this important point. While some SNV calling was augmented with WES data for certain samples, comparisons based solely on WGS data are indeed feasible and were carefully evaluated. We found that the concordance of driver mutations between WGS+WES and WGS-only analyses did not differ substantially. To directly address this concern, we show below results generated using only WGS samples—processed through a consistent pipeline without WES augmentation. These analyses confirm that the key conclusions remain unchanged.

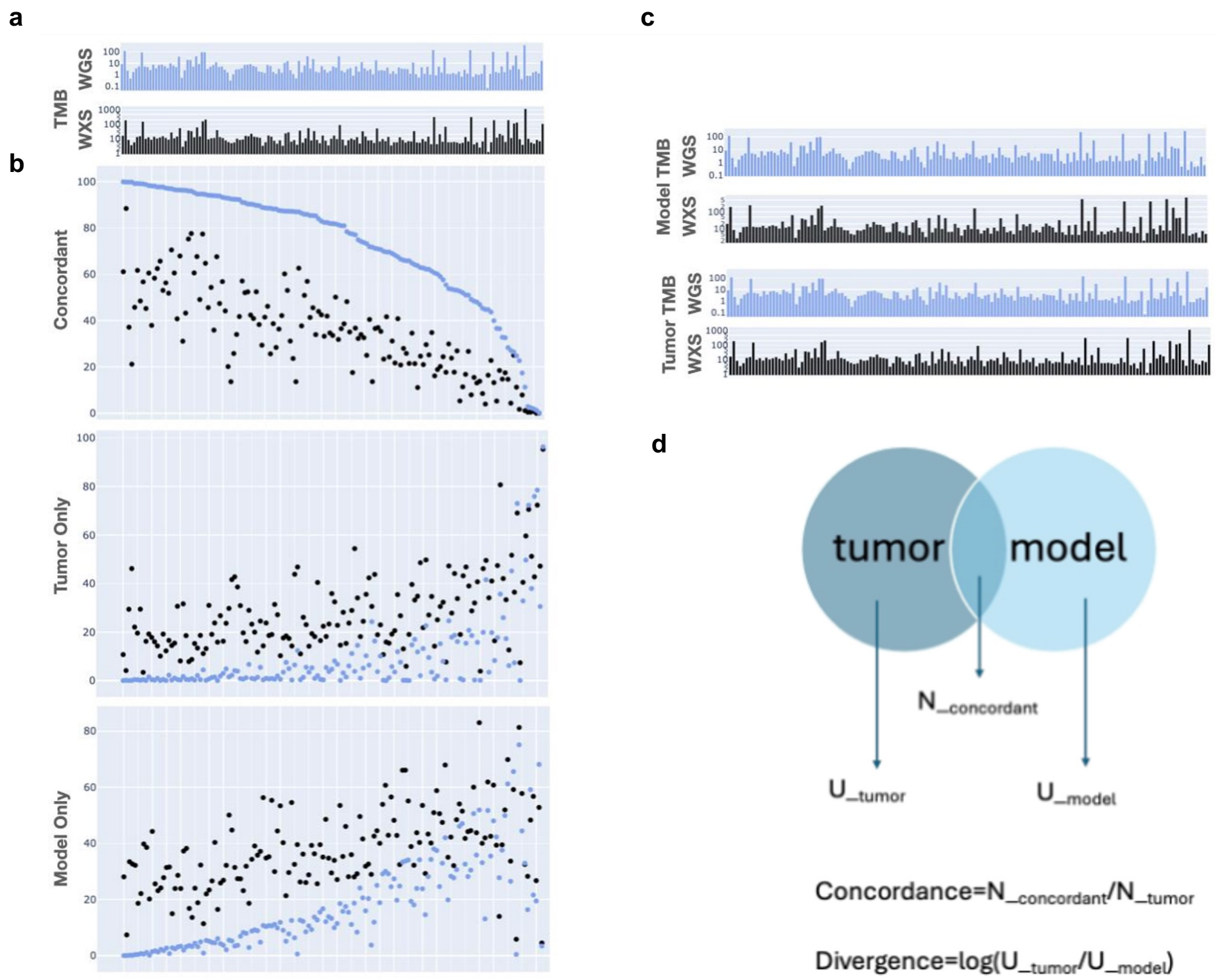

Figure: Comparison of WGS-only and WGS+WES SNV calls. (a) *Tumor mutation burden (TMB) in tumor samples for WGS pairs (blue) and matched WES pairs (black), filtered down to the MultiCallerCalled set.* ***(b)*** *Percent concordance of subsets of calls relative to tumor calls.* ***(c)*** *Comparison of TMB in the tumor samples and matched model samples.* ***(d)*** *Concordance and divergence metrics. Concordance was defined as the fraction of tumor calls observed in the matched model, and divergence as log(U_tumor/U_model), where U represents unique calls.*

9- *Similar to the last point, was copy number calling and structural variant (SV) analysis performed using uniform pipelines across all samples?*

Both copy number (CN) and structural variant (SV) analyses were performed using standardized, consensus pipelines across all samples. For CN analysis, consensus purity and ploidy estimates were used to guide ABSOLUTE solution selection. When available, WES-based mutation calls were used as input; WGS-based calls were substituted in cases in which WES data were not available. For a subset of samples, not all contributing centers returned CN or SV calls; in these cases, consensus calls were derived from the available outputs. Similarly, SV analysis was performed using uniform processing pipelines, with consensus SV calls generated across centers and adjustments made for incomplete data where necessary.

To ensure transparency in genomic input sources for purity and ploidy estimation, we have added **Supplementary Table 14**, which specifies whether WES or WGS data was used for ABSOLUTE analysis for each model.

10- *Figure 2b presents an informative tumor/model pairing analysis but lacks negative controls. How much concordance is observed when random pairing is performed within the same tissue type? How much across tumor types?*

We agree that including negative controls would strengthen the interpretation of the pairing analysis in **Fig. 2b**. To address this, we implemented randomized pairing strategies to serve as a negative control: (i) tumors randomly paired with models across the entire cohort, (ii) tumors randomly paired to models from the same tissue type (intra-tumor-type control), and (iii) tumors randomly paired from different tissue types (inter-tumor-type control). We then computed cosine similarity distributions for each scenario. As shown in the updated **Fig. 2b**, tumor–model pairs exhibit substantially higher signature concordance compared to all randomized controls, including those restricted to the same tumor type. These results underscore the specificity and robustness of the observed tumor–model relationships. **Fig. 2b** in the manuscript has been revised accordingly.

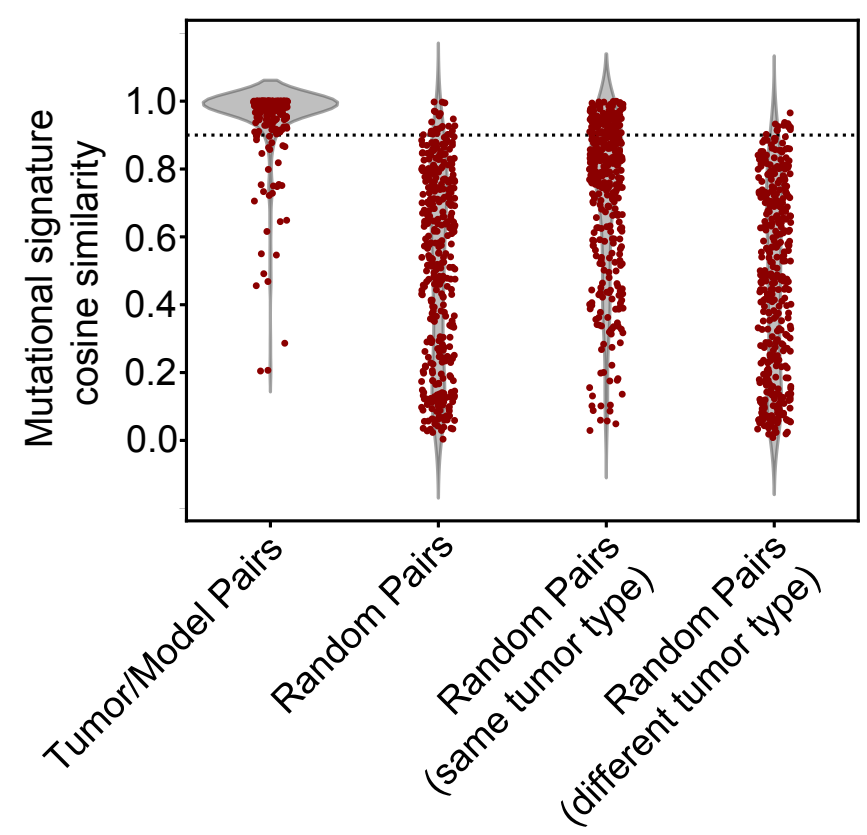

Figure 2: Tumor genomic alterations are recapitulated in cancer models. (b) *Trinucleotide NMF mutation signatures were computed for all tumor/model pairs, as described in Methods: Analysis Methods - Mutation Signature. Similarity in mutational signature profiles was inferred by computing the cosine similarity for each tumor/model pair. We computed a background distribution of cosine similarity scores for randomly shuffled pairs of tumors and models (random pairs). Tumor/model pairs with a cosine similarity score greater than 0.9 were considered concordant.*

11- *Figure 2d indicates that some tumor types are polyploid (some models exceeding >4n). Do these cultured cells maintain a 3-4n state during division, or is there an active conversion from 2n to higher ploidy states?*

The elevated ploidy states observed in cultured tumor models are most plausibly explained by clonal selection following a discrete whole-genome doubling (WGD) event, rather than by ongoing diploid-to-polyploid conversion within a population of WGD-prone cells. This interpretation is supported by the pan-cancer study by Gerstung et al. (Nature 2020, PMID: 32025013), which reconstructs the evolutionary trajectories of over 2,600 tumors and demonstrates that WGD

typically occurs as a single early event in tumorigenesis, rather than as multiple independent events across subclones.

Further supporting this view, single-cell whole-genome sequencing (scWGS) analysis by Funnell et al. (Nature, 2022, PMID: 36289342) showed that polyploidy generally arises from clonal expansion of a cell that has undergone WGD, rather than from multiple cells independently acquiring WGD. Their findings suggest that polyploid genomes tend to stabilize after the initial doubling, without evidence of further rounds of polyploidization.

In our study, we also observe that the copy number profiles of most tumor-model pairs remain similar, suggesting that polyploid clones present in the original tumor are maintained during culture, rather than resulting from ongoing chromosomal duplication. Taken together, these lines of evidence support the interpretation that polyploidy in our models results from clonal selection of WGD-positive clones rather than from continuous acquisition of additional WGD events.

12- *In Figure 2e, it would be useful to highlight the most prevalent mutations (missense/nonsense) in the cohort and cultured models. Additionally, are these models typically driven by a single driver mutation, or do they exhibit polyclonality with multiple driver mutations?*

We appreciate this insightful suggestion. To improve the interpretability of **Fig. 2e**, we have revised the figure to annotate genes based on the predominant functional consequence of their mutations—classified as either gain-of-function (GoF) or loss-of-function (LoF). This approach highlights key biological distinctions while improving visual clarity across gene-level alterations. For readers interested in more detailed variant-level information (e.g., missense vs. truncating mutations), we now direct them to our cBioPortal instance (to be made public upon publication), which includes an interactive OncoPrint and full variant classifications per model.

To further clarify our classification strategy, we have added a detailed paragraph to the Methods section: Genomic Analysis Methods - Driver Oncogene Selection and Classification, which details the approach used to assign GoF or LoF status based on shared mutations in tumor–model pairs. Briefly, only mutations present in both tumor and matched model samples were considered. If no shared mutations were identified for a given gene, it was labeled as Unclassified. Mutation types were grouped as follows: LoF included homozygous deletions and truncating variants (frameshift indels, nonsense, and splice-site mutations); GoF included missense mutations, high-level amplifications, TERT promoter mutations, and in-frame insertions/deletions. A gene was labeled LoF if more than 15% of its shared mutations were LoF events; otherwise, it was labeled GoF.

Regarding clonality, while many tumors and their derived models harbor multiple driver alterations, this does not necessarily imply polyclonality, as multiple drivers may coexist within a single clone. Specifically, only 2% of the samples contain only 1 SNV/indel driver mutation, and 69% samples contain <10 mutations. We included the full list of driver mutation calls in the Supplementary Data: **Supplementary Table 13.**

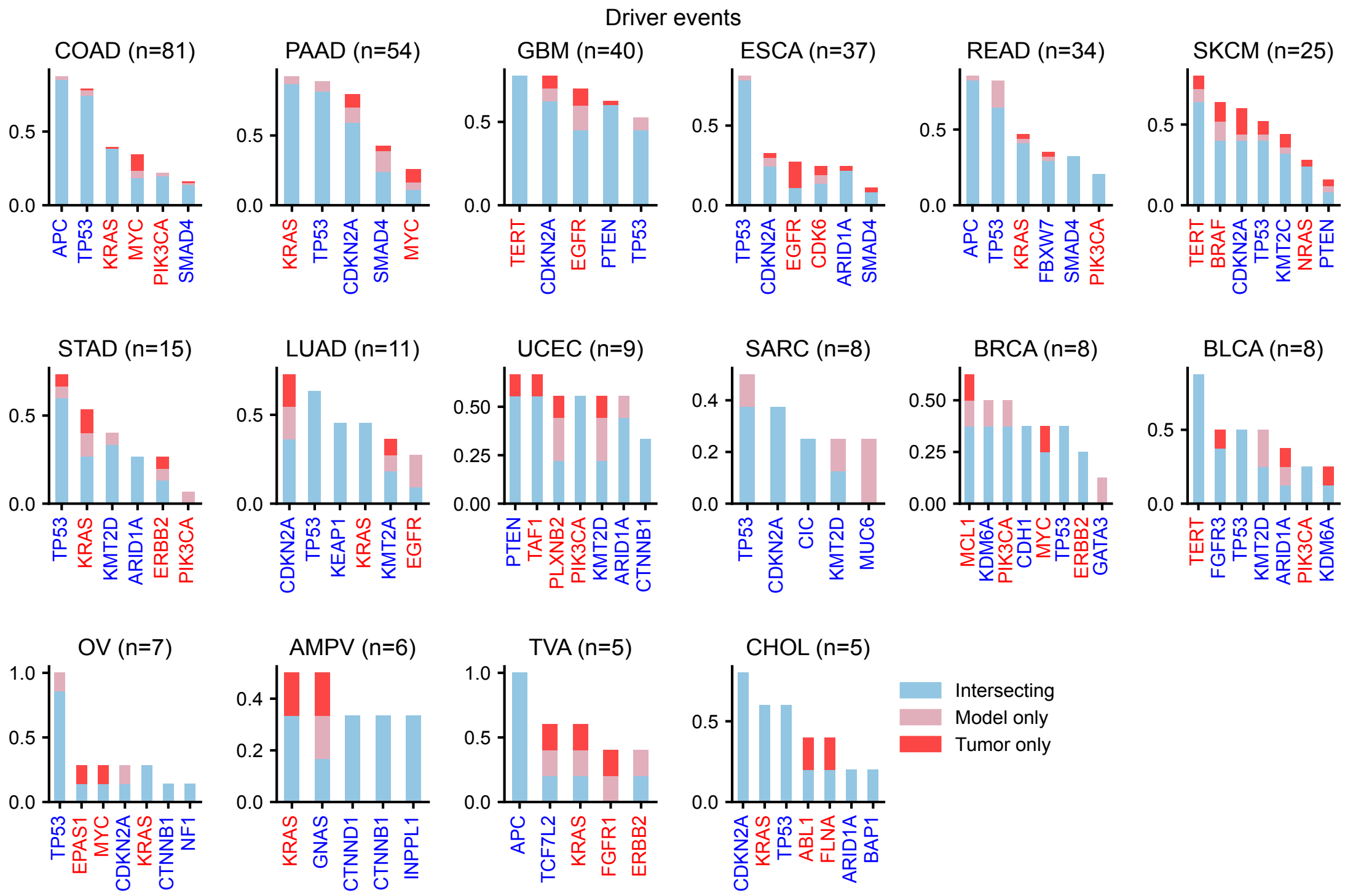

Figure 2: Tumor genomic alterations are recapitulated in cancer models. (e) *Conservation of driver events across tumor/model pairs. Genes were selected based on mutation frequency (either SNVs or copy number alterations (CNAs)) within each tumor type. In this analysis, TERT mutations were canonical TERT promoter mutations. Events were binned into those detected in both the tumor and model (light blue), in the model (light red), or in the tumor (dark red). Genes were classified as either gain-of-function (red text) or loss-of-function (blue text) as described in Methods: Analysis Methods - Driver Oncogene Selection and Classification.*

13- *The manuscript does not provide details on how samples were processed and prepared for snRNA-seq. These methodological aspects should be described.*

We appreciate the referee's comment and have now incorporated a detailed description of the single-nucleus RNA sequencing (snRNA-seq) protocol into the revised Methods section: Single-Nucleus RNA Sequencing (snRNA-seq) - Sample Preparation as indicated in [Article page 49, line 3-13] as follows:

"Frozen samples (n = 34, from 17 tumor–model pairs) were obtained from the BPC Nationwide Children's Hospital, DFCI and ATCC. Nuclei isolation was performed using the Chromium Nuclei Isolation Kit with RNase inhibitor (10X Genomics, Cat. no. 1000494). Samples were homogenized using a pestle in lysis buffer, passed through a column, and centrifuged in debris removal buffer to eliminate residual tissue and debris. The isolated nuclei were then washed, resuspended, and loaded onto the 10X Chromium platform for GEM (gel bead-in-emulsion) generation and barcoding. Following GEM reverse transcription, samples underwent post-GEM RT cleanup and cDNA amplification. After quality control and quantification of cDNA, libraries were prepared using

the Chromium Single Cell 3' Gene Expression Library Construction protocol and sequenced on the NovaSeq platform."

***14-** The result section focuses on HCM-BROD-0613-C71 in the context of Figure 6. Does this culture model indeed remain resistant to TMZ therapy in vitro?*

We thank the referee for this important comment. We agree that functional validation of drug response is essential to support interpretations of resistance phenotypes. Accordingly, in this revision, we performed in-vitro functional viability assays across a representative panel of glioblastoma (GBM) models encompassing both treatment-naïve and previously treated lines, including HCM-BROD-0613-C71. Each model was exposed to temozolomide (TMZ) across an eight-point concentration range (0.03–300 μM) for five days, and cell viability was quantified using the CellTiter-Glo assay.

Within this cohort, the MYCN-amplified model HCM-BROD-0613-C71 exhibited pronounced resistance to TMZ, consistent with its unmethylated *MGMT* promoter, and we noted that this patient had a short overall survival. While the mechanistic contribution of *MYCN* to TMZ resistance remains unclear and has not been systematically investigated in GBM, these findings experimentally confirm the inferred model resistance phenotype.

The new data and analyses have been incorporated into the revised manuscript (**Supplementary Fig. 7**), and details of the experimental setup are described in the updated Methods section under "In Vitro Drug Sensitivity Testing".

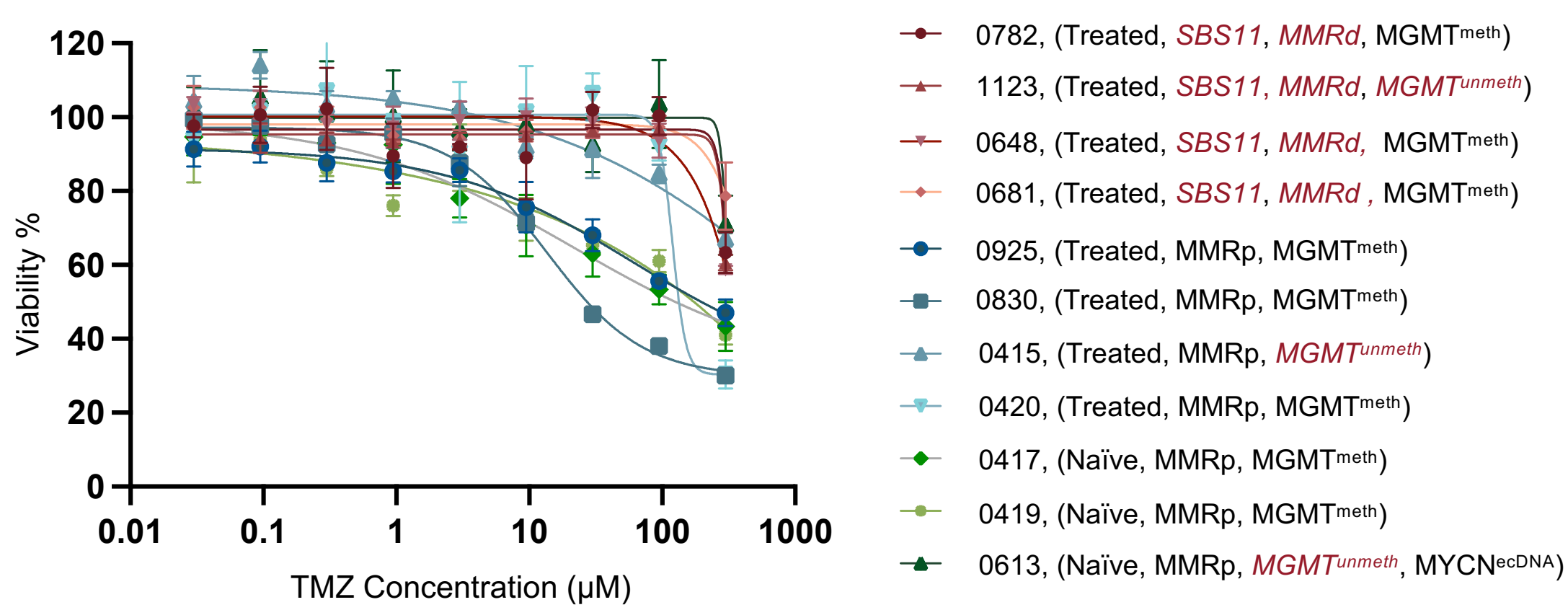

Supplementary Figure 7: Functional validation of temozolomide (TMZ) resistance and associated genomic predictors in glioblastoma models. (a) *Dose–response curves for representative GBM models treated with TMZ (0.03–300 μM).*

Minor comments:

1- *https://github.com/beroukhim-lab/hcmi_copy_number_consensus/: empty readme file*

We appreciate the referee's note. The referenced repository has been linked as a submodule within our centralized GitHub repository (https://github.com/NCICCGPO/HCMI-Flagship-

Manuscript), which consolidates all analysis workflows supporting the manuscript. A full README file and detailed usage documentation for the copy number consensus pipeline are now included within the submodule and will be made publicly available upon publication.

***2-** Page 7 lines 15-17: where is this result reported?*

We thank the referees for noting this. The results for mutation timing and whole-genome doubling (WGD) event comparison have now been explicitly included in the revised manuscript. As shown in **Extended Data Fig. 3c**, we compare the estimated timing of WGD events between models with concordant WGD in both tumor and model ("Both WGD") versus those with model-only WGD. The analysis demonstrates that WGD events observed exclusively in models occur at later relative mutational timepoints, supporting the interpretation that these represent subclonal expansions or selection events during culture adaptation rather than de novo genome duplications.

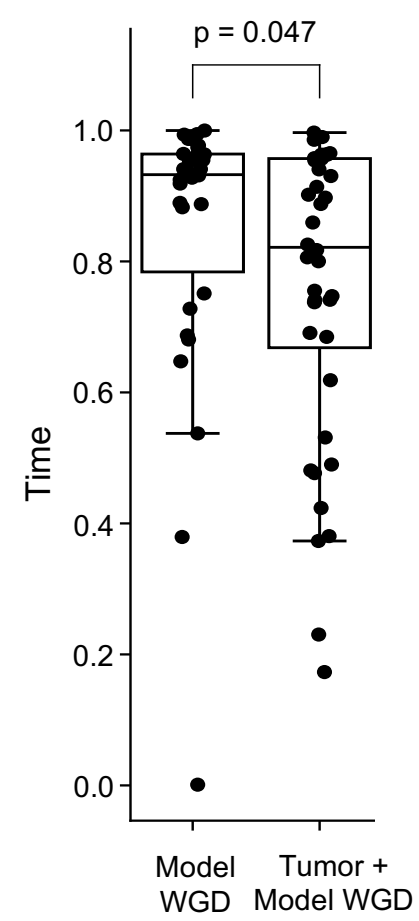

***Extended Data Fig. 3: Levels of concordance of mutational signatures and ecDNA between tumor/model pairs. (c)** Comparison of the estimated relative timing of WGD events between models exhibiting concordant WGD in both tumor and model versus those with WGD detected only in the model. WGD events exclusive to models occur at significantly later relative mutational timepoints (p = 0.047, Wilcoxon rank-sum test).*

***3-** Page 7 line 40: in the text, it mentioned that fig 2g compares models after a year of passaging with the originating tumor. This was not clear to me from the figure and its corresponding legend. The passage status of the models in the figure 2 and 3 comparisons should be clarified.*

We appreciate the referee's comment and agree that clarification of the passage timeline is important for accurate interpretation. All models were cultured to enable long term banking and quality control. In **Figures 2** and **3**, as with other Figures, the models shown in these comparisons were profiled after approximately 10–20 passages, which included expansion, freeze–thaw cycles, and internal quality control procedures.

To address this point, we have added a description of the QC steps in the Supplementary Methods under the section "Biospecimen Collection and Establishment of In Vitro Cultures" [Supplementary Information page 27, line 16].

4- *Page 13 line 6: this adds little value, should either be specified here, in the supplemental text or removed.*

We agree with the referee and have removed the sentence to eliminate redundancy in the revised manuscript.

5- *Page 14 lines 27-28: it is unclear to me why the short OS suggests that MYCN amplification is a causative driver. This should be rephrased or supported by functional evidence.*

We thank the referee for this thoughtful comment and have changed the wording. We agree that the previous phrasing implied causality that is not supported by evidence. While *MYCN* amplification—particularly in the form of extrachromosomal DNA (ecDNA)—has been associated with tumor aggressiveness (Lang *et al.*, *Nat. Genet.*, 2022; PMID: 36123406), its precise contribution to clinical outcome in glioblastoma remains uncertain.

In this revision [Article page 15, line 31-33], we have adjusted the statement to avoid overinterpretation and to provide additional clinical context as follows:

"IIn this participant's tumor and its derived model, MYCN ecDNA amplification coincided with an exceptionally short overall survival (78 days) and an unmethylated MGMT promoter; however, the extent to which MYCN ecDNA contributed causally to clinical outcome remains unclear."

To complement this observation, we performed in-vitro drug sensitivity assays of the HCM-BROD-0613-C71 model, which demonstrated resistance to temozolomide (TMZ), consistent with its unmethylated *MGMT* promoter. These results are now included in the revised manuscript (**Supplementary Fig. 7**).

6- *Page 16 line 1-2: the observance of resistance signatures does not suffice to indicate that these models (that lack immune cells) can be utilized to study immunotherapy. This should be rephrased or supported by drug testing data.*

We agree that in the absence of an immune microenvironment, these models are not suitable for direct functional interrogation of immunotherapy responses. In the revised manuscript, we have rephrased the statement to avoid overinterpretation and to clarify that the analysis is focused on models derived from patients previously exposed to immunotherapy, highlighting those that may inform hypotheses about tumor-intrinsic resistance mechanisms [Article page 17, line 13-16]:

"In a subset of 25 models derived from patients previously treated with immunotherapy, we examined features previously associated with resistance phenotypes. While these models lack an immune microenvironment, they may serve as a resource for studying tumor-intrinsic mechanisms of resistance and identifying alternative therapeutic strategies"

Referee #3

Major comments:

1- *Variable media formulations and culture formats are used depending on the facility. While the genomic profile is not expected to be affected by such variability, transcriptome and perhaps epigenetic status may profoundly be affected by the culture condition or institute location, as is evident from the GBM data. Besides GBM models, are there any data for molecular bias due to the culture batch?*

We agree that transcriptomic and epigenetic profiles are more susceptible to derivation conditions than genomic features, particularly in media-sensitive lineages such as glioblastoma. To assess potential media-associated effects beyond GBM, we performed a new transcriptional distance analysis across the three largest tumor types that were generated at 3 or more institutes and with 2 or more culture conditions – COADREAD, PAAD, and STADESCA. The data on this topic is now included as a new **Supplementary Fig. 6** in the manuscript.

We looked at the top 2,000 variably expressed genes within each tumor type and looked for association of the first principal component (PC) with CMDC or media type. We observed no significant difference in PAAD or STADESCA. While there was an observed association with both CMDC and culture media and the first PC in COADREAD; however, the F-statistics were modest compared to that observed in GBM (Media F-statistics: COADREAD 2.67 vs LGGGBM 36.90; CMDC F-statistic: COADREAD 4.1, LGGGBM was a single CMDC) (**Supplementary Fig. 6a**).

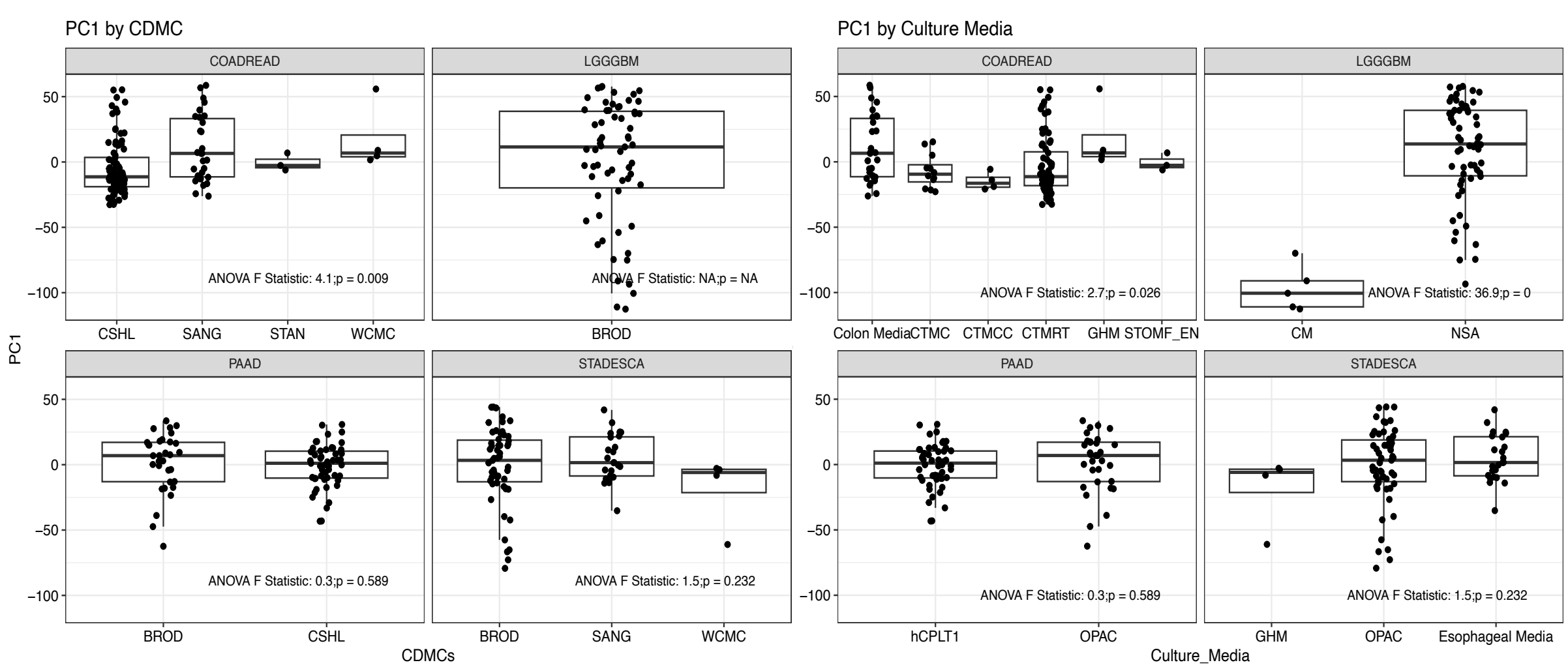

Supplementary Figure 6: Systematic effects of culture media components on transcriptional states across HCMI models. (a) *Distribution of PC1 scores by CMDC (left) or Culture media (right). Boxplots of the first principal component of the top 2,000 most variably expressed genes displayed for four tumor types. ANOVA F statistic and p value are displayed.*

To further address the reviewer request, we also included a new analysis to understand how media components influence key regulatory programs. We analyzed corresponding master regulator (MR) profiles and referenced these to a detailed summary of media formulation composition (now provided in **Supplementary Table 15**). We generated differential MR activity signatures comparing conditions with and without each of 7 HCMI media components: serum, WNT signaling, cholera toxin, TGF beta inhibitors, stem cell additives, hormones and other growth factors. This was performed using a generalized linear model (GLM), controlling for the presence of other media components as covariates. Additionally, to increase statistical power, we pooled models from all cancer types rather than analyzing them within individual cancer types.

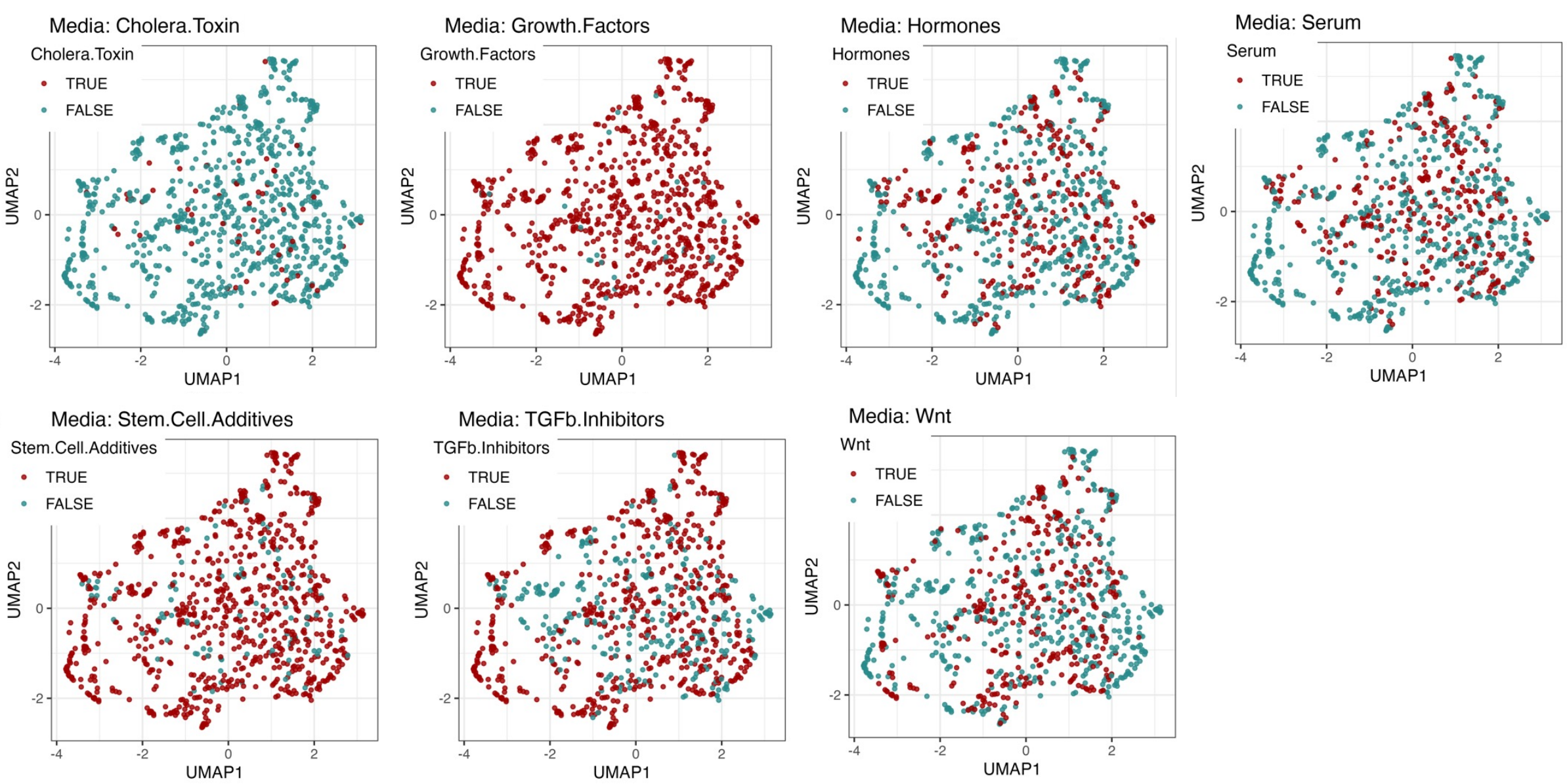

Supplementary Figure 6: Systematic effects of culture media components on transcriptional states across HCMI models. (b) *UMAP plot color-annotated with individual media components: Serum, WNT, cholera toxin, growth factors, TGF-beta inhibitors, stem cell additives, and hormones.*

Furthermore, reactome pathway enrichment analysis of these MR activity signatures confirms that pathways targeted by individual media components were perturbed. For example, the MRs for beta-catenin signaling and cell differentiation pathways MRs were enriched in models grown inWNT and cholera toxin-included media, respectively, while the MR for TGF-beta signaling pathway was enriched in models grown in media containing TGF-beta inhibitors.

GSEA of 14 cancer cell states (angiogenesis, apoptosis, cell cycle, differentiation, DNA damage, DNA repair, EMT, hypoxia, inflammation, invasion, metastasis, proliferation, and quiescence) in CanserSEA (Yuan et al., 2019) further demonstrated that model states varied across media conditions. The differentiation state was highly enriched in models cultured in media containing cholera toxin, while DNA repair states were positively enriched in models grown in media with stem cell additives. Similarly, proliferation and quiescence states were positively enriched in cultures including serum. These results suggest that different culture conditions can induce variation in model states, potentially through the differential activation of regulatory pathways. (**Supplementary Fig. 6c,d**)

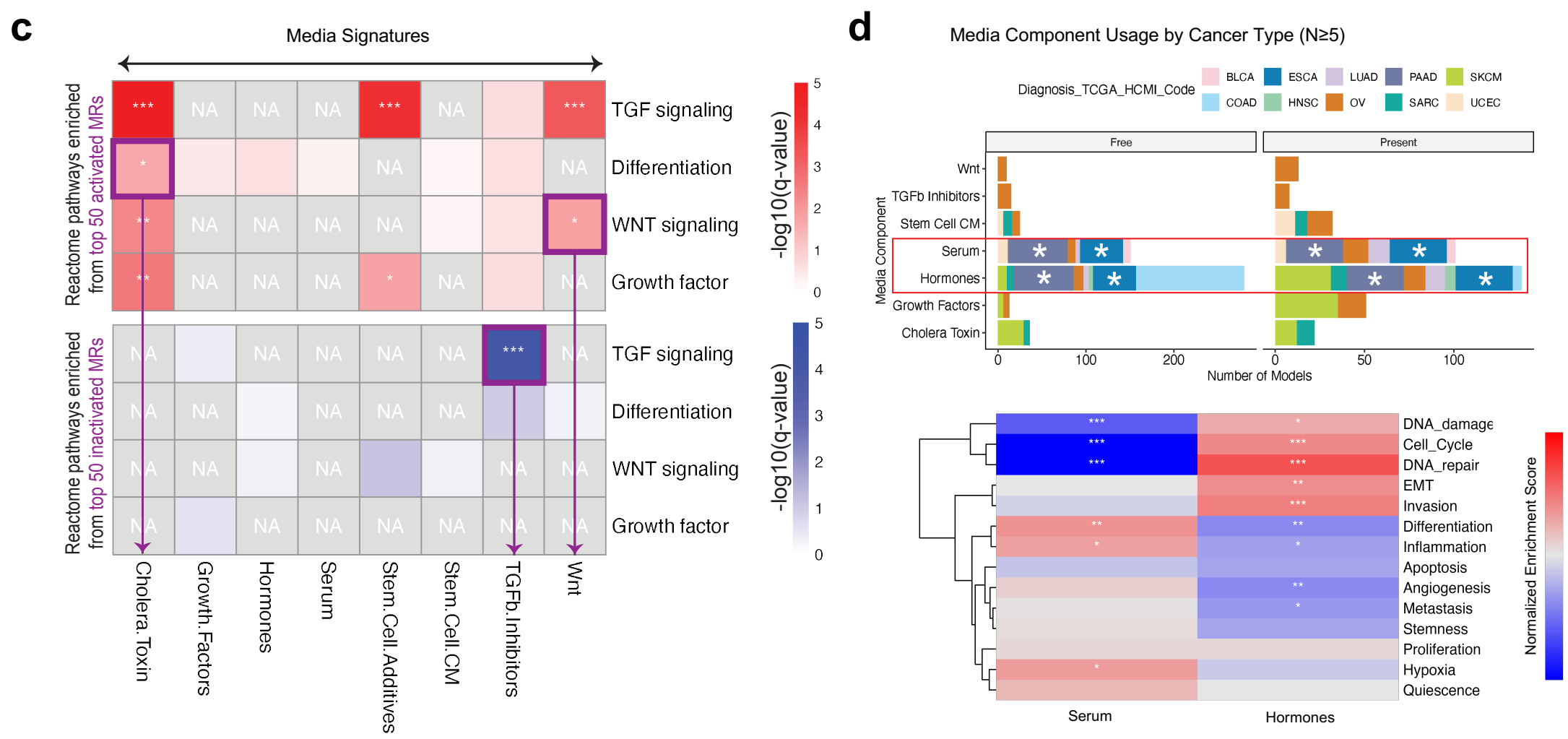

Supplementary Figure 6: Systematic effects of culture media components on transcriptional states across HCMI models. (c) *GSEA of cancer cell states for each media condition-specific signature. The heatmap displays normalized enrichment scores (NES), with red indicating positive NES and blue indicating negative NES. Columns represent media condition-specific signatures, and rows represent cancer cell states obtained from CancerSEA.* ***(d)*** *Comparative analysis of media components–specific transcriptional programs in pancreatic (PAAD) and esophageal (ESCA) cancer models. GSEA plots display pathways enriched in models cultured with versus without gastrin or serum*

2- *Related to the comment above, the authors show that GBM models cultured in different media (NSA vs CM) show distinct levels of neuronal-to-mesenchymal transition. Which component is actually preventing or driving this transition? It looks as if CM is not at all a suitable medium for GBM culture.*

We appreciate this important observation. The NSA medium used for GBM is a defined, serum-free formulation supplemented with EGF, bFGF, and heparin—components known to support neural stem-like states and maintain glial lineage fidelity. In contrast, the CM (conditioned medium) formulation contains fetal bovine serum and cholera toxin, which are both known independently to promote differentiation and introduce variability in cell states. Based on new experiments (**Figure 3 and Supplementary Fig. 5**) we define that serum exposure is a primary driver contributing to the neuroglial-to-mesenchymal shift observed in GBM models cultured in CM and, as the reviewer suggests, labs have made the conclusion that CM is not appropriate for GBM culture. While cholera toxin is reported to elevate intracellular cAMP and modulate differentiation pathways in glioma and other neural cancers (Li et al., *PNAS* 2007, PMID: 17679696; O'Keefe and Cuatrecasas, *PNAS* 1974, PMID: 4366771), its specific role in the context of our NextGen models remains less clear and requires further investigation.

To provide clarity, we have included a comprehensive media components summary of media formulations used across all derivation centers in the revised manuscript as **Supplementary Table 15**.

3- *The section - Translational utility of HCMI models - only infers therapy resistance or sensitivity based on genomics, and whether the models actually have resistance to the pertinent drugs is*

unclear. The GBM part is especially correlative, and the authors should show whether the models with MMR or TMZ resistance signature are really resistan[t] to TMZ compared to those without using a panel of GBM models. Also, the paragraph describing GBM with MYCN ecDNA amplification is n = 1 data, and it is difficult to tell whether the MYCN ecDNA amplification contributed to the poor prognosis as well as clinical aggressiveness. The patient only lived 78 days after sampling, but we cannot judge whether this is short or not, as the tumor may have already been lethally large at the time of sampling. To formally show the aggressiveness of the tumor model, the authors should at least xenotransplant the MYCN ecDNA-amplified GBM model and see whether it's highly invasive and propagative or not.

We appreciate the referee's emphasis on the importance of functional validation to support translational inferences, particularly concerning therapeutic resistance.

Regarding temozolomide (TMZ) resistance in glioblastoma (GBM) models, we acknowledge that our initial interpretations were primarily based on genomic associations. To strengthen these conclusions, we selected a representative subset of GBM models (n = 11) for in vitro TMZ sensitivity assays, including both treatment-naïve and previously treated lines with or without the SBS11 mutational signature. As expected, SBS11-positive models correspond to MMR-deficient tumors, while the remaining models were MMR-proficient.

In vitro functional viability assays were performed across a 0.03–300 µM TMZ range, and normalized area-under-the-curve (AUC) values were derived to quantify response. Among known determinants of TMZ resistance—including MGMT promoter methylation and MMR status—MGMT methylation was the only covariate variable within our tested cohort and was explicitly annotated in our analysis as a potential confounder.

To further substantiate these findings, we have added **Supplementary Fig. 7** to the revised manuscript, reporting the newly added functional data. In brief, the figure presents (a) real-time dose–response curves for the profiled models across the full TMZ concentration range; (b) the distribution of normalized AUC values comparing SBS11/treated versus non-SBS11 (naïve ∪ treated-without-SBS11) groups with explicit MGMT methylation annotation; and (c) two-sided Wilcoxon rank-sum tests, including an analysis restricted to the MGMT-methylated subset, which confirms that SBS11/treated models display significantly attenuated TMZ sensitivity even when MGMT status is controlled. Together, these data demonstrate that the genomic predictions of resistance translate into measurable in vitro resistance, while appropriately validating MGMT methylation as a key covariate in this panel.

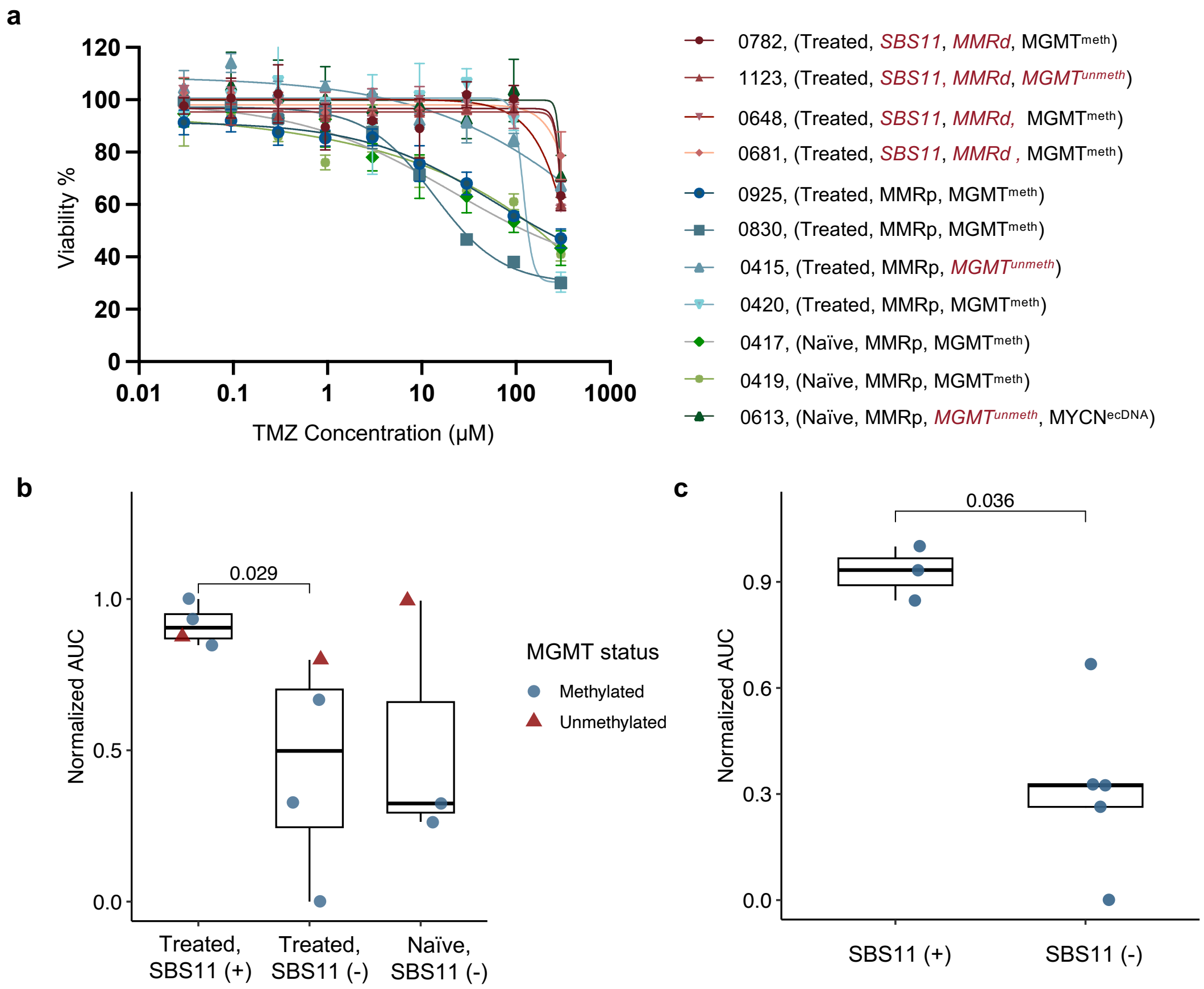

Supplementary Figure 7: Functional validation of temozolomide (TMZ) resistance and associated genomic predictors in glioblastoma models. (a) *Dose–response curves for representative GBM models treated with TMZ (0.03–300 µM).* ***(b)*** *Distribution of normalized area-under-the-curve (AUC) values comparing treatment-naïve and treated low SBS11 signature (–), and high SBS11 signature (+) models. Colors indicate MGMT promoter methylation status.* ***(c)*** *Boxplot summarizing normalized AUC values across MGMT methylated models only stratified by SBS11 mutational signature magnitude: high (+), low (-).*

Notably, one of the HCMI models, HCM-BROD-0782-C71, was included in a comprehensive study by Touat et al. (Nature, 2020, PMID: 32322066), which analyzed hypermutation mechanisms in gliomas. This study highlighted the association between MMR deficiency and hypermutation, providing context for potential TMZ resistance mechanisms. Furthermore, in a subsequent study by Stockslager et al. (Cell Reports, 2021, PMID: 34610309), this model underwent functional drug susceptibility testing using single-cell mass measurements. The findings demonstrated that HCM-BROD-0782-C71 exhibited resistance to TMZ, providing external functional support for the genomic–phenotypic concordance we observe.

Regarding the MYCN ecDNA–amplified GBM case (HCM-BROD-0613-C71), we acknowledge the limitation of *n = 1* and have revised the manuscript to avoid implying causality between MYCN amplification and clinical outcome. This model was included in our TMZ sensitivity screen and showed TMZ-resistance (**Supplementary Fig. 7**) and now have determined it to have an

unmethylated MGMT promoter, consistent with an additional established mechanism of resistance; accordingly, we refrain from attributing resistance or clinical aggressiveness to MYCN amplification per se. Accordingly, we have tempered the text to reflect this limitation and just highlight it as a model available for potential study of MYCN in gliomas. Article text has been revised to [page 15, line 31-33]:

"In this participant's tumor and its derived model, MYCN ecDNA amplification coincided with an exceptionally short overall survival (78 days) and an unmethylated MGMT promoter; however, the extent to which MYCN ecDNA contributed causally to clinical outcome remains unclear.

Additionally, we now include the results of the In vitro functional viability assays in the article [Page 16, line 38 – page 16, line 6] as follows:

"To functionally substantiate the genomic predictors of temozolomide (TMZ) resistance, we performed real-time viability assays across representative GBM models encompassing both treatment-naïve and previously treated lines with or without the SBS11 signature (Supplementary Fig. 7a). Models harboring SBS11 exhibited significantly attenuated TMZ sensitivity compared to non-SBS11 counterparts (Wilcoxon $p < 0.05$), independent of MGMT promoter methylation status (Supplementary Fig. 7b,c). These results confirm that mutational signatures of acquired resistance translate into measurable pharmacologic resistance, consistent with prior reports linking MMR deficiency to hypermutation and therapy escape (Touat et al., 2020; Stockslager et al., 2021). The MYCN-amplified model HCM-BROD-0613-C71 also displayed TMZ resistance consistent with an unmethylated MGMT promoter (Supplementary Fig. 7a), though causality between MYCN amplification and clinical aggressiveness remains unproven, though plausible."

Minor comments:

1- *Please show the success/failure rate for each tumor type. Although organoid derivation was done at multiple facilities using differing strategies, the take rate is an important piece of information.*

We thank the referee for emphasizing the importance of reporting model derivation success and failure rates. A precise take rate across all tumor types cannot be calculated, as not all unsuccessful derivation attempts were systematically sequenced, molecularly verified and recorded with clinical diagnosis by every Cancer Model Development Center (CMDC). However, to increase transparency, we have now incorporated in the revised manuscript a dataset of 168 sequenced tumor samples that failed model establishment, representing the subset for which molecular and diagnostic information was available.

These failed derivations span multiple epithelial lineages, with breast, colorectal, pancreatic, and esophageal cancers representing the most frequently attempted tumor types within this sequenced subset:

Cancer Type	Count (Failed Derivations)
Colorectal	57
Breast	54
Pancreas	25
Esophagus	19
Lung	7
Skin	3
Soft Tissue	2
Brain	1
**Total**	**168**

To better understand factors influencing model derivation outcomes, we consolidated CMDC documentation into four standardized categories of derivation failure:

1. *Primary fails* – no tumor cell outgrowth observed after initial seeding.
2. *Slow proliferators* – cultures exhibiting growth but unable to expand sustainably.
3. *Short-term cultures* – initial growth followed by senescence or growth arrest.
4. *Normal cell outgrowth* – overgrowth by non-tumor stromal or epithelial cells.

These categories were tumor-type–specific: for example, breast cancer cultures frequently failed due to fibroblast contamination and necrotic tissue; pancreatic tumors exhibited high stromal content and low tumor purity consistent with their desmoplastic microenvironment; and colorectal cancers often showed differentiation-related arrest.

To further explore biological determinants of model establishment, we compared molecular features between the 665 successfully derived models and the 168 failed derivations, focusing on the three largest tumor types (colorectal, pancreatic, and esophageal adenocarcinomas). Although tumor purity showed no significant association with model establishment success (**Supplementary Fig. 3 a,b**), gene set enrichment analysis (GSEA) of cancer cell-state signatures (CancerSEA; Yuan et al., 2019) revealed distinct enrichment patterns between failed and successful cases. Failed derivations were enriched for DNA-repair and cell-cycle–associated programs, whereas successful models exhibited greater representation of differentiation, stemness, and EMT-related pathways—suggesting that highly proliferative or repair-active tumors may be less adaptable to long-term culture (**Supplementary Fig. 3 c-f**).

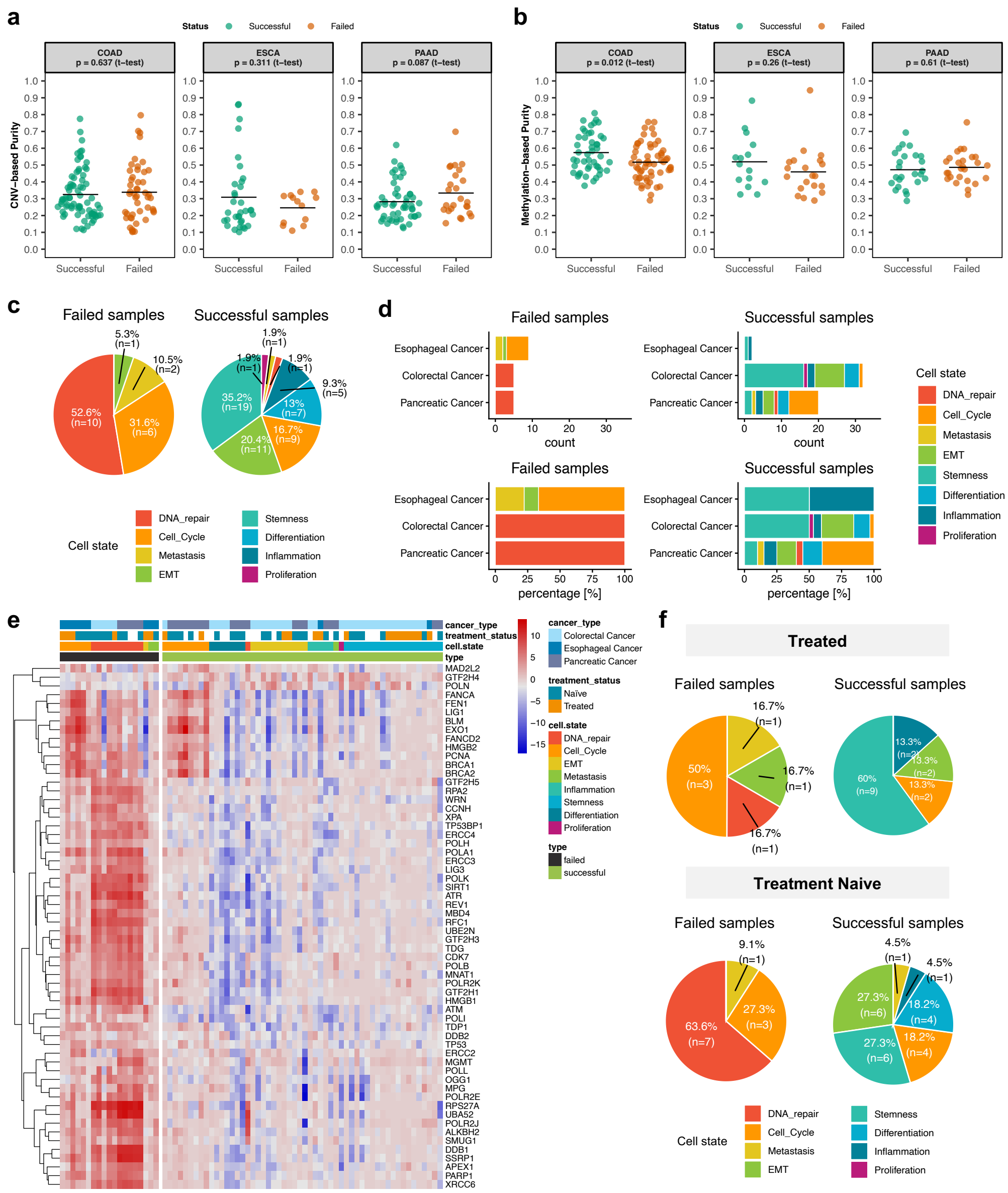

Supplementary Figure 3: Determinants of model establishment success. Comparison of tumor purity between successful and failed model derivations across colorectal (COAD), esophageal (ESCA), and pancreatic (PAAD) cancers. (a) *CNV-based purity estimates and* ***(b)*** *Methylation-based purity. P values were calculated using two-sided t-tests.* ***(c)*** *Cancer cell states (CancerSEA; Yuan et al., 2019) identified by GSEA among failed and successful tumors for colorectal, pancreatic, and esophageal cancers. The piecharts display the proportion (%) of each cancer cell state among failed (left) and successful tumors (right), respectively.* ***(d)*** *bar plots indicate the number of failed (left) and successful tumors (right) for each cancer type, with color annotations representing different cancer cell states. The bottom bar plots (left: failed tumors, right: successful tumors) show the percentage distribution of cell states for each cancer type.* ***(e)*** *Heatmap displaying master regulator activities of genes (rows) annotated for DNA repair across failed and successful tumor samples (columns). Positive and negative gene activities were colored red and blue, respectively.* ***(f)*** *Pie charts showing cancer cell states in failed and successful samples for treated (top) and treatment-naïve cases (bottom), respectively.*

We have added this information to the revised manuscript (**Extended Data Fig. 1** and **Supplementary Table 1**, **Supplementary Fig. 3**) and clarified in the results as follows [Article page 5, line 38 – page 6, line 3]:

"To further characterize variables contributing to model establishment outcomes, we analyzed 168 failed derivation attempts alongside the 665 successfully established models (Extended Data Fig. 1). Specifically, we compared molecular features of successful and failed derivations across the three largest tumor cohorts (COAD, ESCA, and PAAD). While tumor purity did not emerge as a significant covariate of success (Supplementary Fig. 3a,b), failed tumor samples presented increased representation of DNA-repair and cell cycle–associated states, whereas successful tumor samples displayed greater representation of differentiation, stemness, and EMT-related programs (Supplementary Fig. 3c-f)."

2- *Were the patients and tumor types selected base[d] on some criteria, or simply randomly recruited? I just wondered, as some major tumor types, such as esophageal squamous cell carcinoma, lung squamous cell carcinoma, hepatocellular carcinoma and prostate cancer are not represented in the cohort.*

Patients were recruited under shared inclusion criteria applied across all Cancer Model Development Centers (CMDCs) but were generally not restricted by clinical or demographic characteristics. Tumor type selection was not random; rather, HCMI and participating CMDCs intentionally prioritized cancer types with significant unmet clinical or research needs—such as rare, understudied, or treatment-resistant tumors—as well as those for which institutional expertise, access to well-annotated biospecimens, and interest in downstream functional studies were strongest. This approach ensured that the resulting models would be both clinically relevant and uniquely positioned to support mechanistic investigation, translational research, and development of new therapeutic strategies.

The absence of certain major tumor types—such as esophageal squamous cell carcinoma, lung squamous cell carcinoma, hepatocellular carcinoma, and prostate cancer—reflects the fact that each CMDC focused on tumor types supported by their specific expertise and specimen availability, rather than any lack of clinical relevance. On a more practical basis, it also relates to the nature of the funding mechanism which was established as a quantitative federal contract to produce a certain number of models in a specified time period, so feasibility of model generation was also a factor.

3- *Fig 6d and Extended Data Fig. 9f show that some tumor models were derived from patients with Lynch syndrome. How was the MSI status of these tumors? Also, why does the number of cases differ between the germline data and somatic data? Weren't second hit mutations in MMR genes identified in the remaining cases?*

We thank the referee for raising this important point and appreciate the opportunity to clarify. Germline analysis identifies Lynch carriers based on pathogenic variants present in the normal sample, whereas the somatic calls shown in **Extended Data Fig. 9f** represents second-hit events in mismatch repair (MMR) genes detected in tumors or derived models. By definition, germline

variants are not expected to appear in somatic mutation calls, and second hits were detected only in a subset of germline-positive cases, consistent with the known biological variability of Lynch syndrome.

To address the referee's question regarding MSI status, we applied MANTIS to tumor and model sequencing data where coverage permitted. Among the evaluable Lynch-associated cases, one model (HCM-CSHL-0378-C18) was classified as MSI-H, whereas five others were MSS (e.g., HCM-CSHL-0237-C18, HCM-SANG-0281-C18, HCM-SANG-0271-D12). For the remaining carriers, MSI status could not be determined due to insufficient coverage.

4- *Fig. 4b and Extended Data Table 1 shows that there are 2 models of small intestinal adenocarcinoma, but n = 3 in the main text (page 10 line 15). Please correct.*

We thank the referee for identifying this inconsistency. The discrepancy has been reviewed, and the main text has been corrected in the revised manuscript to reflect the accurate count of two small intestinal adenocarcinoma models.

5- *The number of TCGA samples in Extended Data Fig. 5b is very small in many cancer types compared to the original TCGA reports. For example, TCGA sequenced more than 200 LUADs, but only 2 are used here. Why are there so few cases?*

At the time of our initial analysis, only 1,467 copy number segment files were available through the GDC TCGA data portal. Consequently, the number of samples with sufficient data to infer driver mutations represented approximately 17% of all TCGA cases with copy number information, resulting in uneven representation across cancer types. As of January 30, 2025, the GATK4 CNV segment files were released as part of GDC Data Release 42.0, substantially expanding copy number data availability across TCGA. We have now incorporated these updated GATK4 CNV files, re-performed the validation, and increased cohort coverage, thereby improving the robustness and representativeness of our findings. The plot has been updated in **Extended Data Fig. 5**, as shown below.

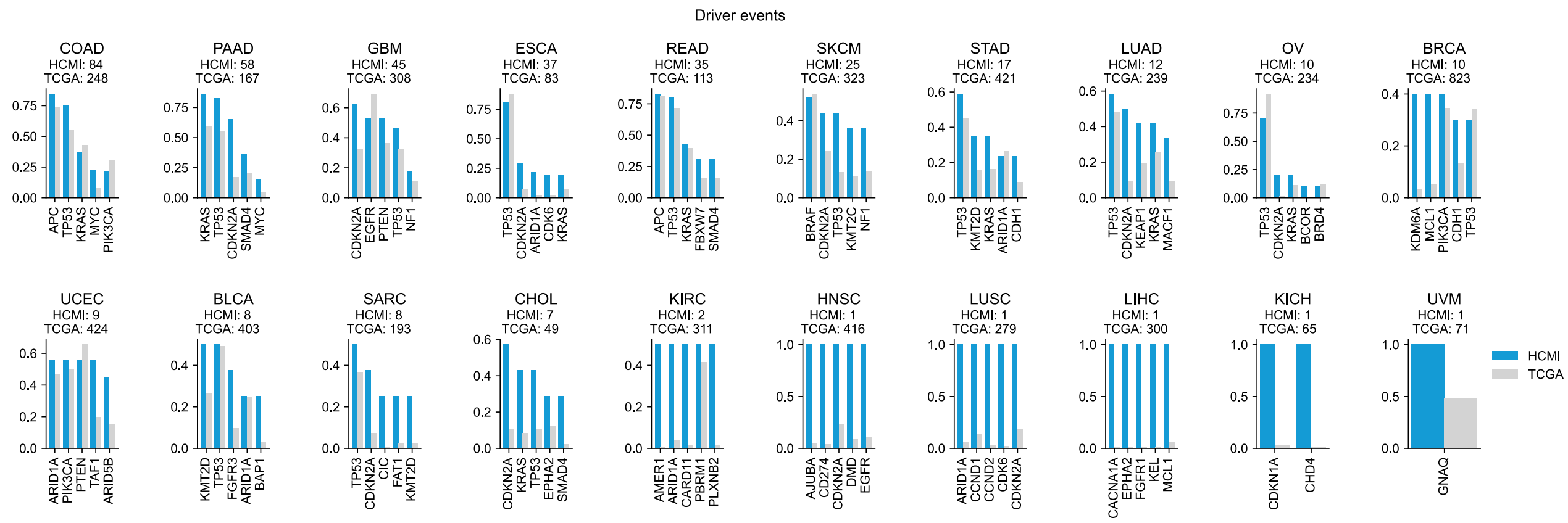

Extended Data Fig. 5: Comparison of HCMI, TCGA and CCLE. (c) *Comparison of tumor-type relevant driver events in patient tumor samples for HCMI (blue) and TCGA (grey) cohorts. ED Fig. 5b shows driver events, including pathogenic and likely-pathogenic SNVs, high-level amplifications, and homozygous deletions in each gene. For the HCMI cohort, patient tumor samples used to directly generate the matching model were used for the comparison. Genes associated with these driver events were curated from Bailey et al.124 and supplemented by expert opinion (Supplementary Table 10).*

Referees #4 and 5

"Incredible collaborative effort – the creation of 665 new models is staggering and should be commended! Particularly 153 models of rare cancers and 71 non-european populations - wow! The efforts to make these "distributable" are wonderful."

We thank the reviewers for these positive comments.

Major comments:

1- *The description of the genetic ancestry analysis on lines 40-42 of p5 is a bit clunky and makes it sound incorrectly that each model comes from a single population. Please rephrase. Figure 1c gives a similar impression, since each model appears to be categorized to a single ancestry, whereas each model includes various contributions of each ancestry. It would also be helpful if the authors could provide a little more context as to thresholds used to determine ancestry based on genetic analysis.*

We thank the referee for this important observation. In the revised manuscript, we have clarified that genetic ancestry assignment was based on the highest proportional contribution of inferred ancestry, rather than assuming exclusivity. This labeling was used to simplify visualization and align with summary frameworks commonly used in population-level studies. To ensure clarity, we revised the main text, figure legend (**Fig. 1c**), and added explicit cross-references to **Supplementary Fig. 3**, which provides the full proportional ancestry estimates for all models.

The Main text [Page 6, line 8-14] is now revised to:

"To characterize the ancestral makeup of the patients from whom HCMI models were derived, we estimated genetic ancestry using whole genome sequencing (WGS) data from the germline tissue and inferred proportional contributions across five continental groups (Fig. 1c) (see Methods: Genetic Ancestry Estimation). Across the cohort, the predominant ancestry groups were European (85%), African (6.5%), East Asian (3.7%), admixed American (2.5%), and South Asian (2.3%). Overall, 71 models are from primarily non-EUR ancestry donors. As expected, some participants showed complex admixture of multiple ancestries (Supplementary Fig. 4)."

And **Fig. 1c** legend is revised to:

"Predicted continental ancestry of HCMI models, based on genomic data. Each model is represented by its majority ancestry component (see Supplementary Fig. 4 for full ancestry proportion assignments)".

2- *The finding of culture media influencing mesenchymal state is important and significant but was however based on an n=3 for the CM condition. In other tumor types were there any culture-related differences? If authors shift the existing models into CM media, would this shift cells to a mesenchymal state? It would also be good to know if these mesenchymal states are reversible, for example can they regain the epithelial state when media is shifted?*

We appreciate the referee's recognition of the significance of media-driven mesenchymal transitions. In our GBM cohort, the serum-containing (FM/CM) arm comprised three models, which we acknowledge is a limited sample size. However, the broader transcriptional pattern is supported by the corresponding manuscript from the Broad Institute team in which historical serum-cultured cell lines with next-generation patient-derived neurosphere models grown under serum-free conditions (NSA) are compared. Both datasets consistently demonstrate that serum exposure of GBM-derived cell models promotes mesenchymal-like transcriptional states, whereas serum-free conditions preserve glial/stem-like programs.

To assess whether media-associated effects extend beyond GBM, we analyzed the top 2,000 most variably expressed genes within each tumor type and tested the association of the first principal component (PC1) with culture media across the three largest cohorts generated in multiple media conditions: COADREAD, PAAD, and STADESCA. As shown in **Supplementary Fig. 6**, no significant associations were detected in PAAD or STADESCA, whereas COADREAD displayed a modest media-related effect. In contrast, GBM showed a markedly stronger association (ANOVA F-statistic: GBM 36.9 vs. COADREAD 2.7). These results support the conclusion that among the tumor types analyzed, GBM models exhibit the strongest detectable transcriptional response to culture medium. These results do not exclude, however, the potential for strong media effects in other tumor types under more divergent culture conditions.

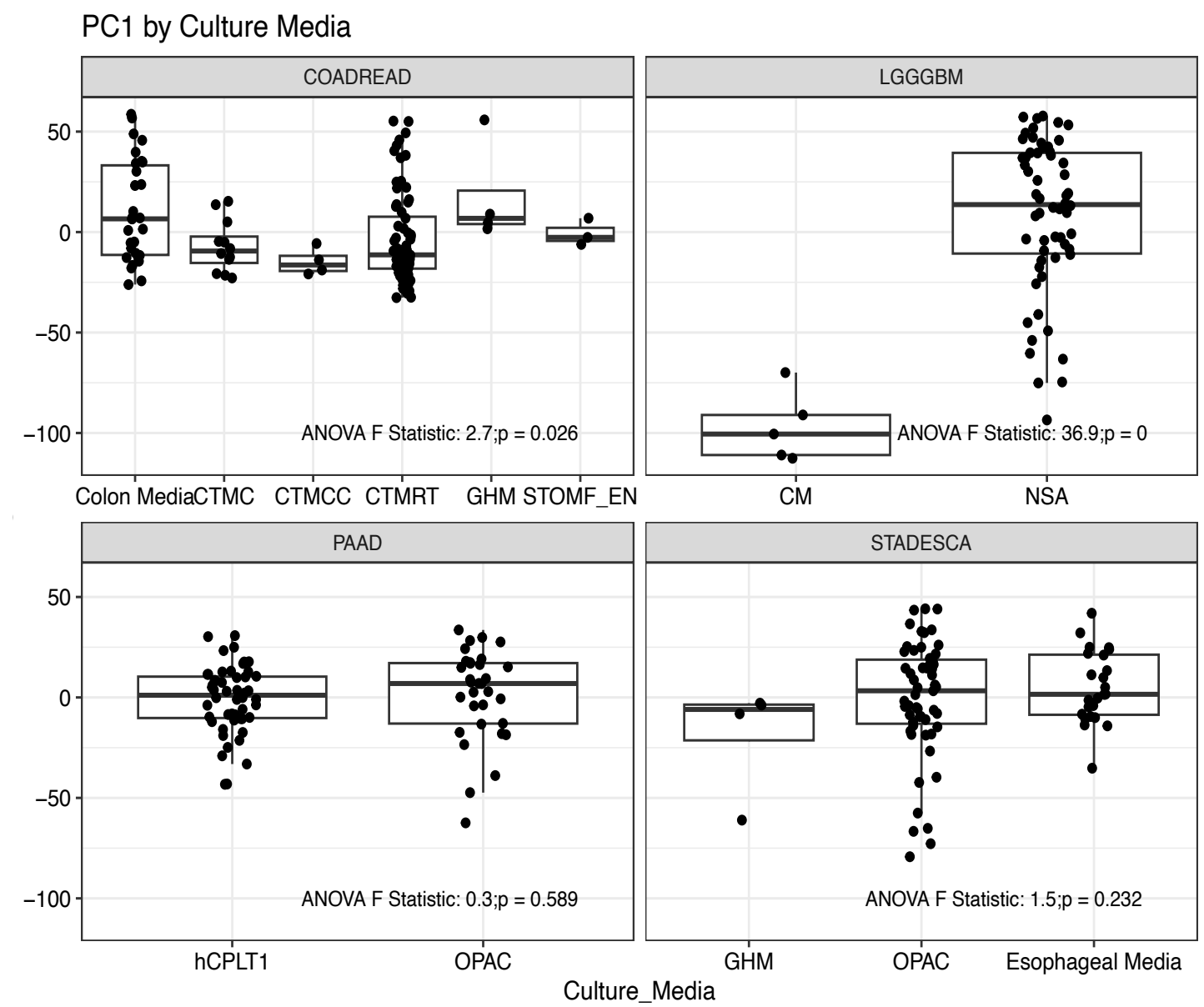

Supplementary Figure 6: Systematic effects of culture medium components on transcriptional states across HCMI models. (a) *Distribution of PC1 scores by Culture medium. Boxplots of the first principal component of the top 2,000 most variably expressed genes displayed for four tumor types. ANOVA F statistic and p value are displayed.*

To determine whether medium-induced states are reversible, we conducted medium-switching experiments on a GBM model with distinct baseline conditions—HCM-BROD-0416-C71 (NSA-derived, glial). After recovery in its native media, the model was cultured under the alternate condition (NSA to FM) for two weeks, followed by immunofluorescence imaging for SOX2 and Vimentin and RNA-seq profiling at 72 hours and two weeks (**Fig. 3h,i; Supplementary Fig. 5**).

NSA to FM transitions induced a mesenchymal-like morphological shift evidenced by reduction of SOX2 glial protein levels in the nucleus, together with transcriptional up-regulation of mesenchymal *CD44*, *CHI3L1* and suppression of glial *SOX2*, *SOX9*, *OLIG2*, *PROM1*. To explore the reciprocal transition, we attempted FM to NSA switching in an FM-derived mesenchymal model (HCM-BROD-0002-C71); however, cells did not remain viable beyond 72 hours under NSA conditions, preventing further molecular characterization. While this suggests a potential lack of plasticity in certain mesenchymal-biased models, additional testing across a broader set of models and extended conditioning timelines would be required to determine whether these states are reversible.

While the FM to NSA transition in the mesenchymal-derived model did not yield viable cultures for further molecular characterization, the NSA to FM transition in the glial-derived model (HCM-BROD-0416-C71) demonstrated clear modulation of lineage programs and was therefore incorporated into the revised manuscript [Article page 10, line 19-29] as follows:

"To assess the modulation of media-induced transcriptional states, we used the glioblastoma model HCM-BROD-0416-C71, originally derived under serum-free NSA conditions. The model

was maintained in its native NSA medium and switched to serum-containing (FM medium) conditions (Supplementary Fig. 5a). Immunofluorescence imaging revealed a morphological reorganization toward a mesenchymal-like phenotype in FM medium, and glial-like morphology in NSA medium (Fig. 3h). This morphological shift was associated with reduced nuclear SOX2 intensity in FM conditions (Supplementary Fig. 5b-d). Transcriptomic profiling at 72 hours and two weeks demonstrated a directional shift in lineage programs, characterized by induction of mesenchymal markers and suppression of glial genes under FM conditions (Fig. 3i, Supplementary Fig. 5e). Together, these findings indicate that serum-driven mesenchymal programs in GBM exhibit plasticity under different experimental conditions."

And the corresponding data are shown in **Fig. 3h,i** and **Supplementary Fig. 5**.

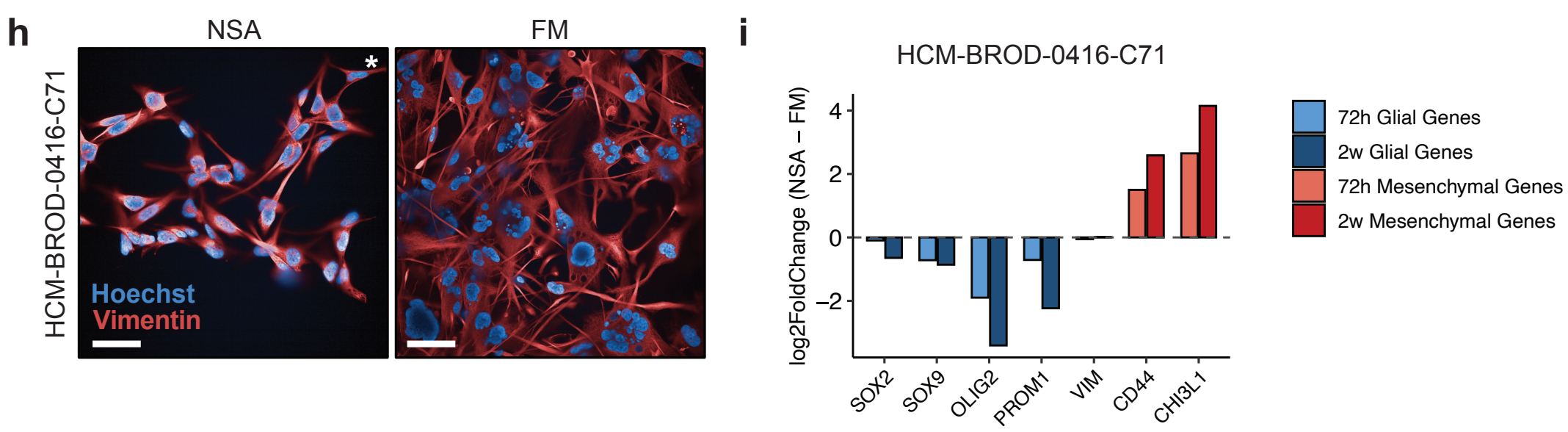

Figure 3: Transcriptional and epigenetic concordance between models and their matched tumors. ***(h)*** *Immunofluorescence of GBM models under native and switched media conditions. Representative images of glioblastoma models HCM-BROD-0416 (baseline NSA culture) after two weeks in neural stem cell medium (NSA) and formulated/conditioned medium (FM/CM). Cells were stained for Vimentin and Hoechst to visualize cytoskeletal organization and nuclei, respectively. Scale bars, 50 µm. Asterisk marks the original media condition of the model. (i****)*** *Lineage-marker expression changes following the media switch. Log2 fold-change in expression of glial (SOX2, SOX9, OLIG2, PROM1) and mesenchymal (VIM, CD44, CHI3L1) markers after switching HCM-BROD-0416 from NSA to FM for 72 hours and two weeks.*

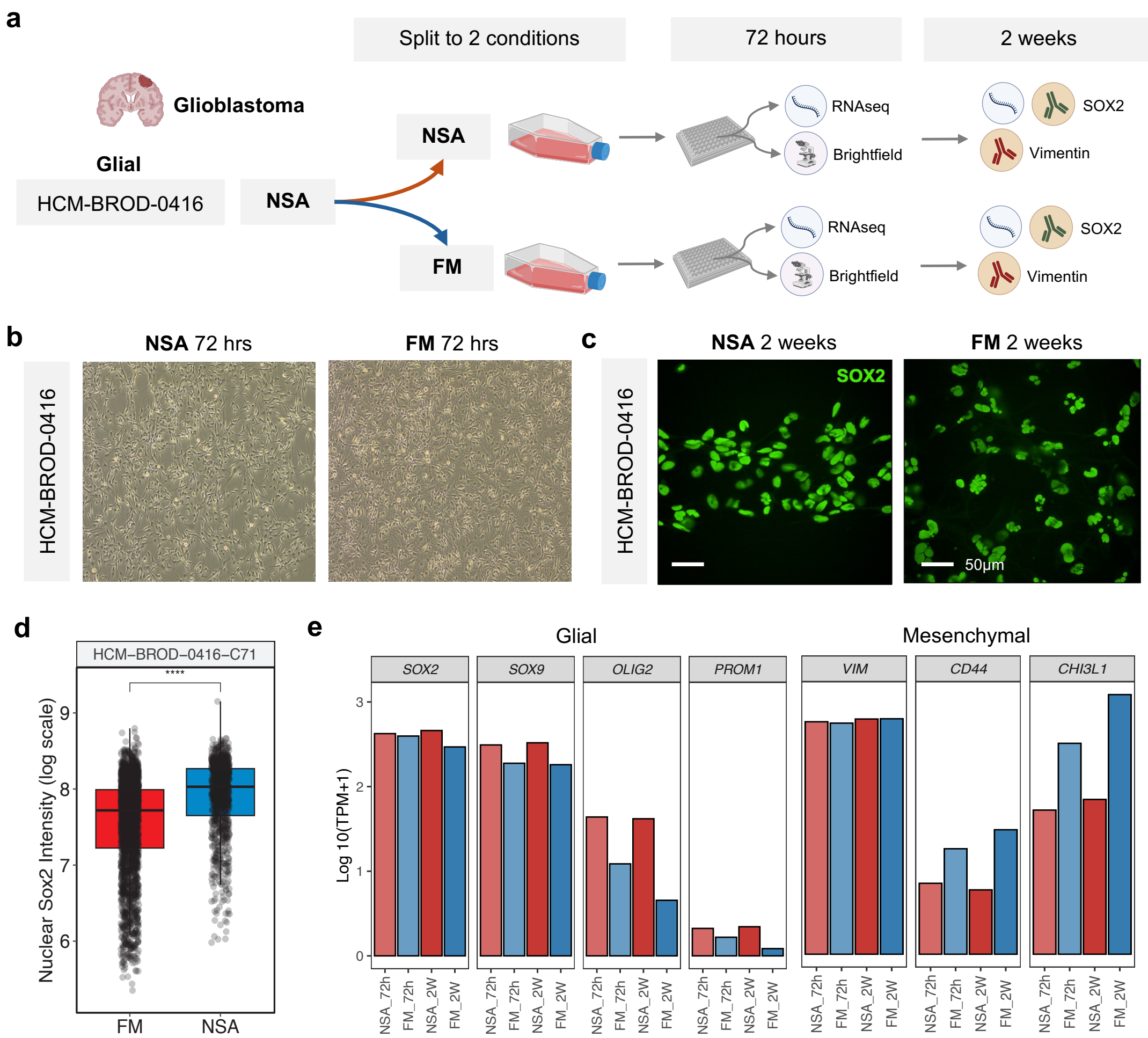

Supplementary Figure 5: Modulation of media-driven transcriptional states in glioblastoma. (a) *Schematic overview of the experimental design. The glioblastoma model HCM-BROD-0416, originally maintained under serum-free NSA conditions, was split and re-cultured in NSA or FM. RNAseq and bright field imaging were performed 72 hours later. RNAseq and SOX2/Vimentin immunostaining were done after a further two weeks of incubation.* ***(b)*** *Representative brightfield images of HCM-BROD-0416 after 72 hours and two weeks in NSA or FM.* ***(c)*** *Immunofluorescence staining of SOX2 after two weeks of culture in NSA or following transition to FM.* ***(d)*** *Quantification of nuclear SOX2 intensity across >10,000 cells per condition.* ***(e)*** *RNA-seq expression of glial (SOX2, SOX9, OLIG2, PROM1) and mesenchymal (VIM, CD44, CHI3L1) lineage markers across media conditions and time points.*

3- *All data availability links should be verified activated at publication time, most of the links do not yet work. For example, I was not able to evaluate this tool because it has not been shared with me: HCMI Explorer Suite (https://appshare.cancer.gov/connect/#/apps/6c6f4b6c-8e55-4410-a49f-dbac2eebeba5) The link in the methods here did not work: https://github.com/D-Harouni/HCMI_Explorer_Suite)*

We fully agree that all data availability and tool-access links must be active and functional at the time of publication. The HCMI Explorer Suite is currently hosted on the NIH AppShare platform

and is maintained under restricted review access; it will be made publicly accessible at the time of publication. Representative screenshots of the interface are provided in **Supplementary Fig. 9**. The tool's full source code, documentation, and usage instructions have been migrated to a dedicated repository within the centralized HCMI GitHub organization (https://github.com/human-cancer-model-initiative/HCMI-Explorer-Suite) and linked as a submodule within the flagship code repository (https://github.com/NCICCGPO/HCMI-Flagship-Manuscript), ensuring long-term versioning, stability, and transparency. This repository is currently private and will be made public at the time of publication.

Similarly, the curated GDC Publication Page—created to centralize validated links to all referenced datasets across modalities—has been approved by NCI and will be publicly released following completion of the NCI release workflow (representative snapshots of the publication page development interface are provided in the rebuttal appendix). In the meantime, all raw data are fully accessible via the HCMI-CMDC project on the GDC Data Portal. Model-level processed genomic data are accessible via cBioPortal. All URLs will be verified and activated prior to publication to ensure full usability.

We have updated the Data Availability section [Article page 55, line 1-13] to clearly specify all access portals and their intended uses, ensuring that readers can readily navigate raw, processed, and interactive datasets as follows:

"To support data access, we maintain a publicly accessible instruction portal (HCMI Page at https://www.cancer.gov/ccg/research/functional-genomics/hcmi/using-hcmi) which serves as the central entry point for the resource. Molecular and genomic characterization data from this study are available through the HCMI-CMDC project on the NCI Genomic Data Commons (GDC) web page (https://portal.gdc.cancer.gov/projects/HCMI-CMDC). Processed molecular datasets (mutation calls, copy-number profiles, clinical annotations) are available through cBioPortal (https://www.cbioportal.org/study/summary?id=pancan_hcmi_2025). Interactive model-level exploration is available through the HCMI Searchable Catalog (https://hcmi-searchable-catalog.nci.nih.gov), and the HCMI Explorer Suite (https://appshare.cancer.gov/content/b8c2812f-6ca5-492d-a9f4-b81107aed8d6/), which offer interactive visualization of clinical timelines and genomic annotations. HCMI patient-derived models are distributed through the ATCC HCMI collection (https://www.atcc.org/hcmi)."

4- *The title says "organoid models" but the HCMI also includes a proportion of 2D models. Consider removing the organoid from the title or adding "cell line" to the title? Also, were the discordant models enriched for organoids or cell lines? In other words, was there any difference in model fidelity based on the 2D vs 3D culture?*

We appreciate the referee's point regarding terminology. To better reflect the diversity of culture formats in the resource, we have revised the title to:

*"**A Compendium of Next-Generation Patient-Derived Models of Diverse Cancer Types**."*

Regarding discordance patterns, we have not observed a consistent enrichment for 2D or 3D models among discordant cases. However, we note that culture format decisions were not systematically randomized or experimentally paired; in many cases, model derivation was performed in only one format per tumor type (e.g., gastroesophageal models were generated exclusively as organoids). As a result, the dataset lacks balanced representation of both formats across most tumor types, limiting the power to directly compare fidelity between 2D and 3D models.

***5-** Are organoid models superior to the 2D models? [Your] group that can answer this important question. If so why? If not, why not?*

We appreciate the importance of this question. Based on our analyses, we do not observe global or uniform improvements in molecular fidelity attributable solely to 3D format across tumor types. Rather, the influence of model format appears to be context-dependent and is often secondary to other variables—most notably culture medium composition. For example, in glioblastoma—the only lineage in our cohort with sufficient numbers of both formats for direct comparison (n≈60) (**Fig. 1d**)—transcriptional fidelity tracked more strongly with medium formulation than with 2D versus 3D growth formats. Other tumor types, such as Wilms tumor and sarcoma, also include both 2D and 3D models but in numbers too limited to support robust format-specific comparisons. By contrast, some of the largest cohorts in this study, including colorectal (COAD) and pancreatic adenocarcinoma (PAAD), are composed almost entirely of 3D organoid models, precluding meaningful direct comparisons of structure within those tumor types.

The two accompanying manuscripts that include HCMI models highlight context-dependent strengths of 3D next-generation formats. First, the DepMap Next-Generation Models study systematically profiled 3D organoids/spheroids and 2D lines with matched WGS/RNA-seq and genome-scale CRISPR screens. They report that 3D models (i) preserve tumor-like transcriptional programs that are frequently silenced in traditional 2D lines, (ii) capture dependencies linked to those programs, and (iii) exhibit differences in gene essentiality attributable to both growth format and culture medium, with overall CRISPR screen quality comparable between formats. These findings argue for complementarity rather than categorical superiority, and they emphasize the need to account for media as a major covariate.

Second, the Sanger organoid biobank demonstrated that patient-derived organoids can recover clinically relevant drug-response phenotypes that were not consistently captured in 2D models, further underscoring the unique value of 3D systems.

Taken together, our data and these resources indicate that organoids are not uniformly "superior" to 2D models; rather, each format provides complementary strengths. Three-dimensional models help to overcome some of the limitations of 2D systems, capturing clinically relevant responses and biological programs that may be attenuated or plateaued in traditional adherent cultures. At the same time, many biomarkers and dependencies are consistently shared across 2D and 3D platforms, underscoring the robustness of key findings across formats. The most informative strategy is therefore to view 2D and 3D resources as complementary: organoids extend discovery beyond the constraints of 2D, while 2D models continue to provide scalability

and historical continuity. Together, these resources enable a more comprehensive view of cancer biology when culture medium and other covariates are carefully controlled.

Other specific comments:

1- *There seem to be a lot of "N/A" values in Extended Data 1, HCMI model sequencing and align. Some added context for why there are so many missing values would be helpful.*

We thank the referee for this helpful observation. The values in **Extended Data Fig. 1** reflect the frequency of sequencing data across tumor, model, and normal samples (trios, pairs, or single models) rather than totals per model. Apparent "N/A" values do not represent missing data but instead indicate where a given sample type or sequencing modality was not generated. This arises from the practical constraints of multi-center model generation and sequencing, including limited input material, sample quality control failures, and the fact that not all samples were eligible for each modality (WGS, WXS, RNA-seq, or methylation). For example, some models were sequenced without matched tumors or normals, and vice versa. To improve clarity, we have now included a detailed legend in the manuscript for **Extended Data Fig. 1** as follows:

"The diagram summarizes sequencing data generated for the HCMI cohort (n = 665 models). Counts are shown for models with available tumor, normal, and model samples, including complete trios (n = 399), pairs of model and normal (n = 233), pairs of model and tumor (n = 14), and models without matched tumor or normal samples (n = 19). For each group, the number of samples profiled by WGS, WXS, RNA-seq, and DNA methylation is indicated."

2- *The legend for Supp Figure 3 should define the population abbreviations used in the labels in the figure. It also appears that the samples are ordered by increasing AFR ancestry, rather than decreasing as stated in the legend, if I understand the figure correctly.*

We thank the referee for pointing this out. We have revised the legend for **Supplementary Fig. 3** to define all population abbreviations in full (continental and population-level) and to correct the description of ancestry-based ordering so that it accurately reflects the direction shown in the figure. The figure legend has been updated in the manuscript accordingly:

"Continental-level ancestry categories: AFR = African, EUR = European, EAS = East Asian, SAS = South Asian, AMR = Admixed American. Population-level abbreviations correspond to 1000 Genomes Project reference populations: CHB = Han Chinese in Beijing, CHS = Southern Han Chinese, JPT = Japanese in Tokyo, KHV = Kinh in Vietnam, BEB = Bengali in Bangladesh, GIH = Gujarati Indian in Houston, ITU = Indian Telugu in the UK, PJL = Punjabi in Lahore, STU = Sri Lankan Tamil in the UK, CEU = Utah Residents with Northern and Western European ancestry, FIN = Finnish in Finland, GBR = British in England and Scotland, IBS = Iberian populations in Spain, TSI = Toscani in Italy, CLM = Colombian in Medellín, PEL = Peruvian in Lima, PUR = Puerto Rican in Puerto Rico, ESN = Esan in Nigeria, GWD = Gambian in Western Division, LWK = Luhya in Kenya, MSL = Mende in Sierra Leone, YRI = Yoruba in Ibadan."

3- *Were germline polymorphisms used to ensure that the model and tumor were derived from the same individual? This could have been clarified in the text and used to further support that the more divergent models were in fact still from the same individual.*

Yes, this was confirmed prior to sequencing. We kindly direct the referee to the following passage in the "Biospecimen processing and QC" section of the methods [page 31, line 29-32], reproduced below:

"All extracted DNA was subjected to a custom Sequenom Single-nucleotide polymorphism (SNP) panel or the AmpFISTR Identifiler (Applied Biosystems, Waltham, MA, USA) to verify that all specimens representing a case were derived from the same patient."

4- *Figure 5g – it is not clear to me whether the data shown in the right two panels (078 and 089) is from the model-only, tumor-only, or both*

We appreciate the referee's attention to the figure. The right two panels (078 and 089) in **Fig. 5g** includes nuclei from both the tumor and corresponding model. In the revised version, we have clarified the figure legend accordingly. The legend now reads:

"PCA showing malignant subpopulations across the entire PAAD cohort (left), and for tumor–model pairs 078 and 089 (right). For the right panels, parental tumors and their matched models are jointly plotted, highlighting preservation of malignant subpopulations albeit at different frequencies."

5- *Line 15-17 on p13 appears incorrect: "Overall the HCMI collection includes models that were derived from patients with 491 treatment events before model generation (naive models) (Extended Data Fig. 9a) and 533 treatment events post model generation (post-treatment models)." If treatment events occurred prior to model generation, then these models should be post-treatment, not naïve.*

We thank the referee for pointing out this inconsistency. This was a typographical error in the original text. The corrected statement [Article page 14, line 21-25] now reads:

*"Overall the HCMI collection includes models that were derived from patients with 491 treatment events after model generation (naive models) (**Extended Data Fig. 9a**) and 533 treatment events before model generation (post-treatment models) (**Extended Data Fig. 9b**)."*

6- *For SNV/indel calling- please clarify how variants were "merged" from the three callers. Were they required to be called by 1,2,or 3 of the callers?*

As detailed in the Methods section under "SNV/indel calling – Consensus method," only variants that were identified by all three variant callers were retained in the final consensus set. This stringent approach was used to maximize specificity and ensure high-confidence variant calls.

7- A summary of the tumor stage of the tumor used to derive each model would have been helpful. I did not see this commented on in the text – are most of these tumors early stage primaries? Some seem to be metastatic and post-progression, so a more clear summary of this in Fig 1 would be helpful. Does this differ from CCLE in a complementary way?

We thank the referee for this helpful suggestion. Information on whether each model was derived from a primary, metastatic, or recurrent tumor is included in **Supplementary Table 1** under model demographics tab. However, we agree that visualizing this information would enhance clarity. Accordingly, in the revised manuscript we have incorporated this distribution into **Fig. 1c** as part of the overall model overview as shown below, and we have added the associated descriptive sentence to the revised manuscript [Article page 5, line 28-30]:

*"Most models were established from primary tumors (n = 394; 59.2%), with additional representation from metastatic lesions (n = 250; 37.6%) (**Fig. 1b**)."*

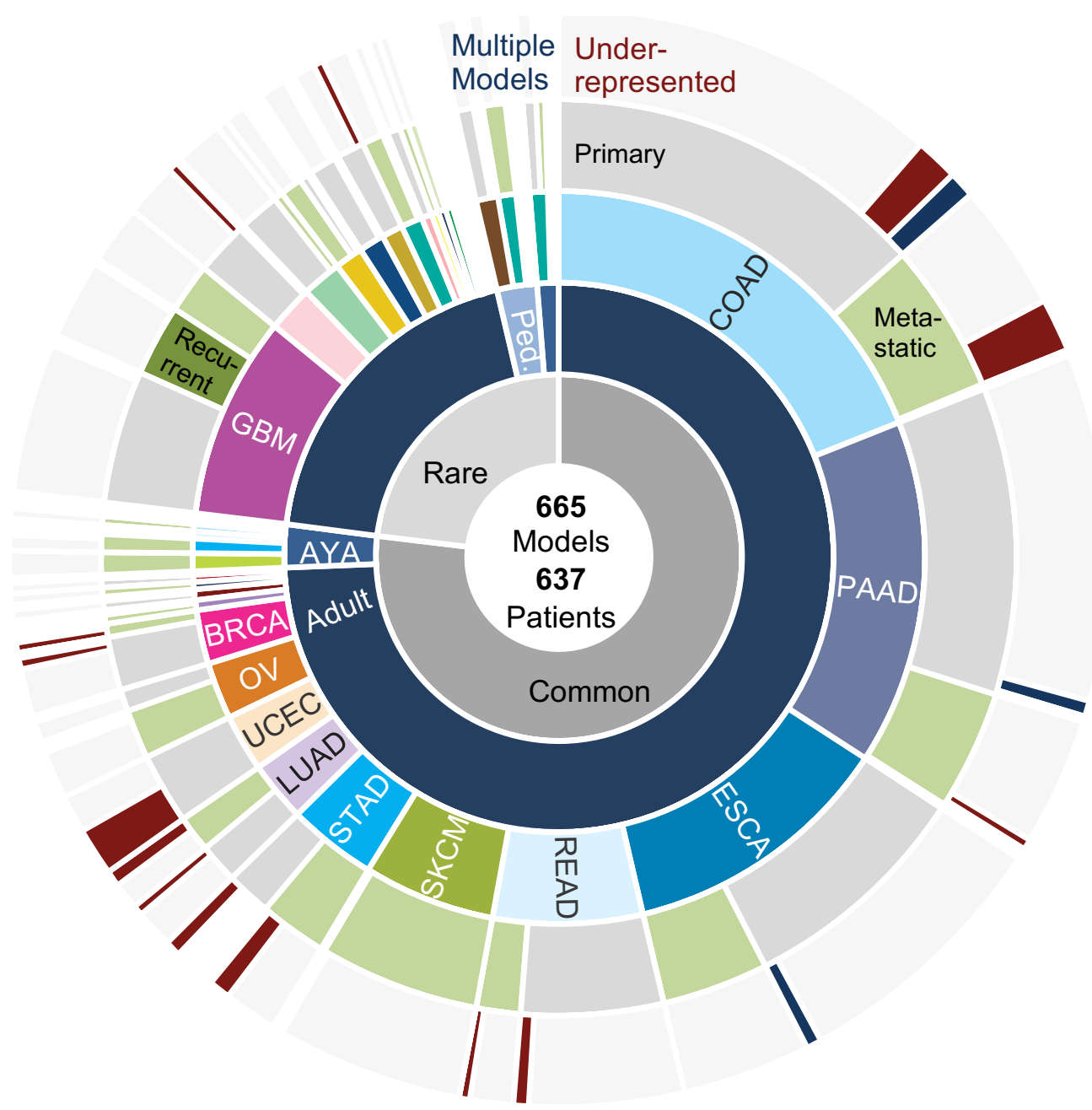

Figure 1: Overview of the HCMI resource and clinical demographics. (b) *The distribution of HCMI models by cancer type and patient demographics. The inner layer classifies HCMI donors by common vs. rare cancers; the second layer groups patients by age category (Pediatric, AYA, and Adult); the third layer groups tumors according to primary HCMI diagnosis, with cancer types coded as in (Supplementary Table 1); the fourth layer maps the tumor status (primary, recurrent, metastatic, and non-malignant), while the outer layer highlights underrepresented patients in red, and patients with multiple models in blue.*

To provide specific context in comparison to CCLE, we added a new panel to Extended Data **Fig. 5** that compares the distribution of primary versus metastatic models between HCMI and CCLE across shared cancer types. This visualization highlights how HCMI complements existing resources by expanding model representation.

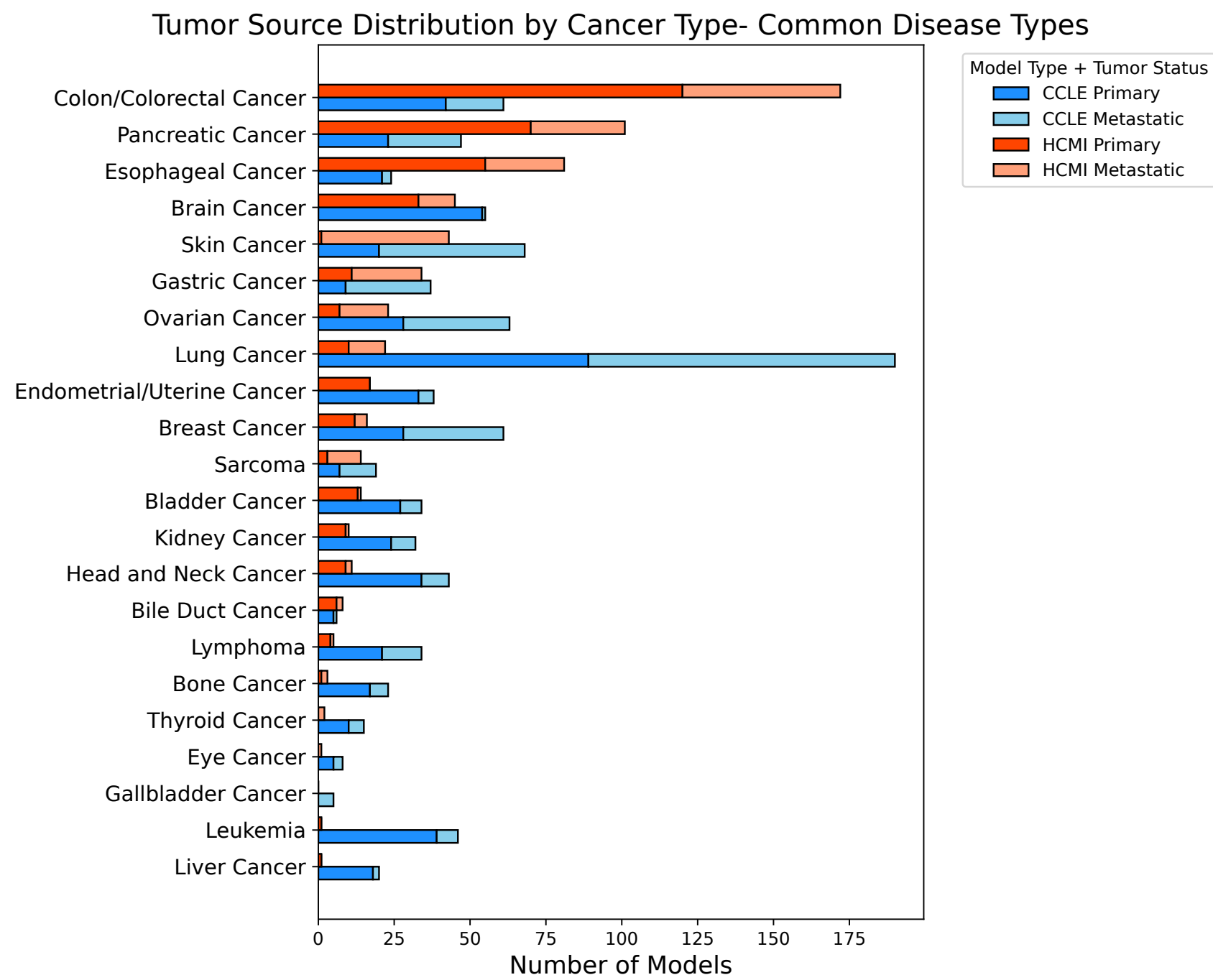

***Extended Data Fig. 5: Comparison of HCMI, TCGA and CCLE. (a)** Stacked bar plot showing the number of models across major disease types in the HCMI (red) and CCLE (blue) collections, stratified by tumor status (primary vs. metastatic). Bar lengths indicate total model counts.*

***8-** In figure 2 the authors do a good job of comparing concordance between tumor and model. Particularly they show that majorly across models there is a significant concordance in mutations and signatures. However a couple of things would be helpful to the readers:*

o Are the VAF of mutations (SNV/INDELs/CNS/SV etc) in models the same as tumors, particularly for driver/tumor suppressors alterations.

There is a general trend that variant allele frequencies (VAFs) tend to be higher in models compared to their matched tumors, especially for SNVs and indels. We believe that a significant portion of this difference is attributable to lower tumor purity in the original samples, which can dilute VAFs, especially for clonal mutations. To better account for this, we computed cancer cell fractions (CCFs) for somatic SNVs and indels by incorporating both sample purity and local copy number. That procedure enabled a more accurate comparison of the clonality of mutations shared between tumors and models. We include below (**Figure**: Variant allele frequency (VAF) and cancer cell fraction (CCF) distributions in tumors and matched models) an analysis comparing the CCFs of shared driver mutations to assess the extent to which these events are clonal and preserved across systems.

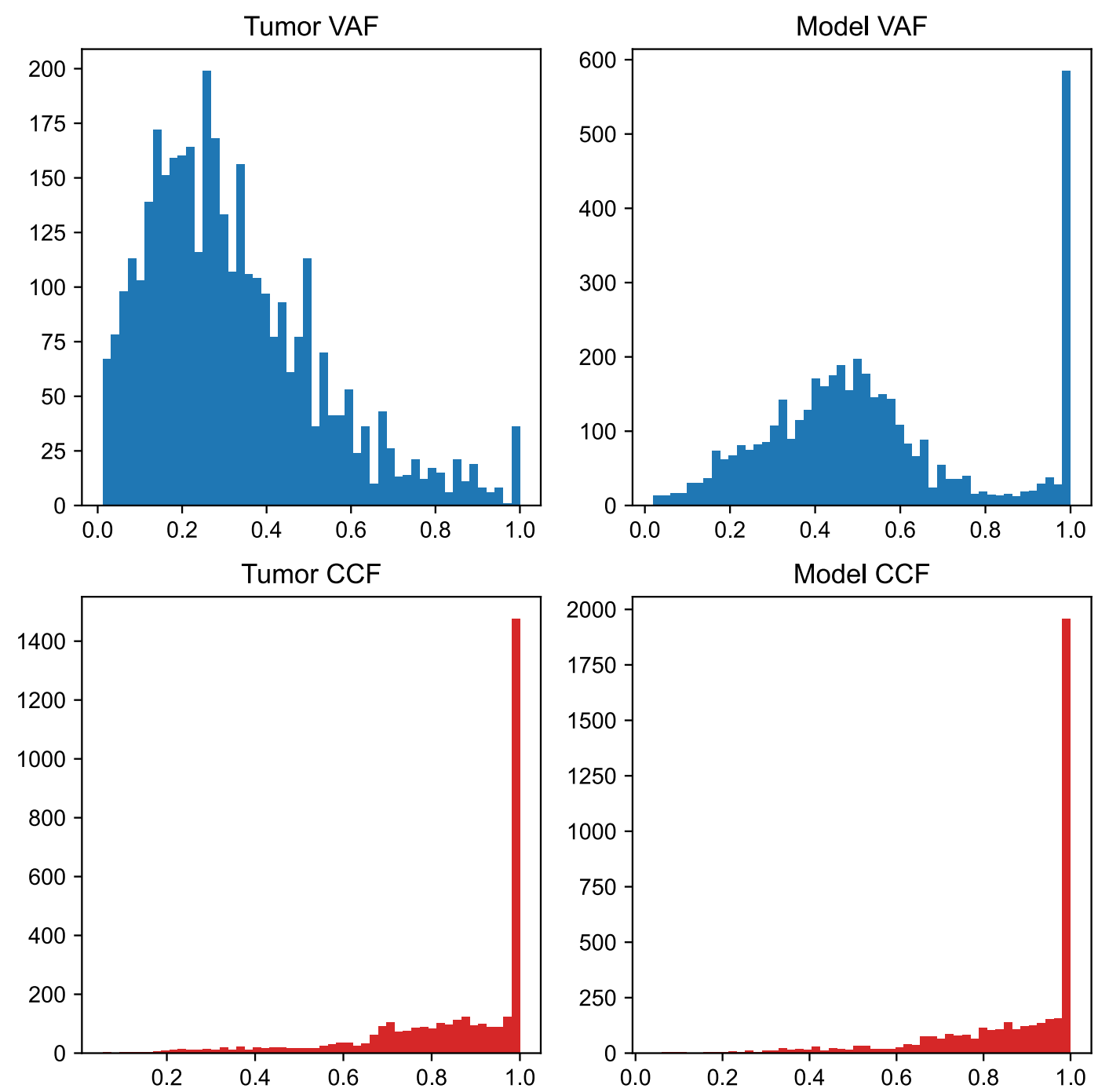

Figure: Variant allele frequency (VAF) and cancer cell fraction (CCF) distributions in tumors and matched models. *Histograms show the distribution of somatic SNV/indel variant allele frequencies (VAFs) in parental tumors (top left) and corresponding models (top right). Cancer cell fraction (CCF) estimates, which adjust for purity and local copy number, display highly similar distributions between tumors (bottom left) and models (bottom right).*

o For drivers/tumor suppressors mutations that are only seen either in models or in tumors have a different VAF. Meaning if a very low VAF of drivers seen in either tumor or model affecting their detection ?

Thank you for this important point. We agree that differences in variant allele frequency (VAF) could influence detection sensitivity. To minimize this effect, we sequenced tumor samples at higher depths than their matched models, thereby increasing our ability to detect low-VAF mutations. As shown in upper panels of the figure below (Variant allele frequency (VAF) and cancer cell fraction (CCF) distributions in tumors and matched models), a substantial proportion of somatic variants occur at low VAFs (<0.1), indicating good sensitivity in the tumor sequencing. Although variants with extremely low VAFs (<0.02) may still be missed, the overall similarity between the tumor and model CCF distributions (lower panels) supports the conclusion that detection sensitivity was generally balanced between tumors and models.

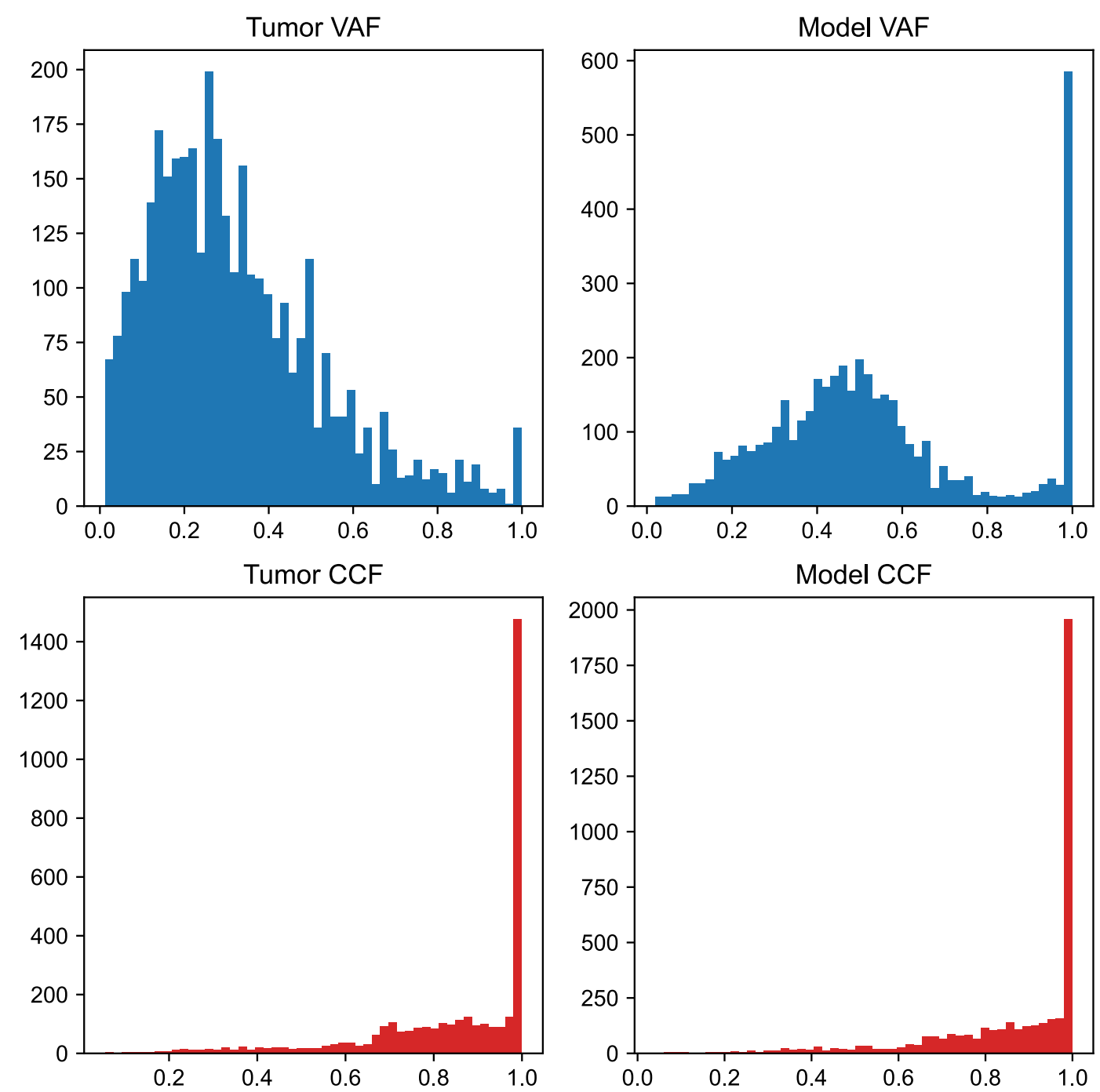

Figure: Variant allele frequency (VAF) and cancer cell fraction (CCF) distributions in tumors and matched models. *Histograms show the distribution of somatic SNV/indel variant allele frequencies (VAFs) in parental tumors (top left) and corresponding models (top right). Cancer cell fraction (CCF) estimates, which adjust for purity and local copy number, display highly similar distributions between tumors (bottom left) and models (bottom right).*

o Also are there any clinical patterns that correlate with discordance? Aka what is driving discordance? Is it genetically defined or more technical issues that could cause the discordance? Note: the reviewer has taken into account the suggestions that low tumor purity and/or FFPE artifacts could be reasons.

We appreciate the referee's question about the potential causes of tumor-model discordance. To further explore whether the observed discrepancies were genetically or technically driven, we compared the fraction of loss-of-heterozygosity (LOH) segments shared between tumor and matched model pairs as a proxy of shared clonal architecture and genome-wide allelic concordance.

As shown in the figure below (LOH fractions in concordant vs. discordant tumor–model pairs) , pairs with 0 shared SNVs ("discordant") exhibited significantly reduced shared LOH fractions compared with non-discordant ("concordant") pairs (rank-sum $P = 4.71 \times 10^{-5}$). This finding indicates that discordant pairs are characterized by broader allelic divergence across the genome, consistent with clonal evolution or subclonal selection during model establishment.

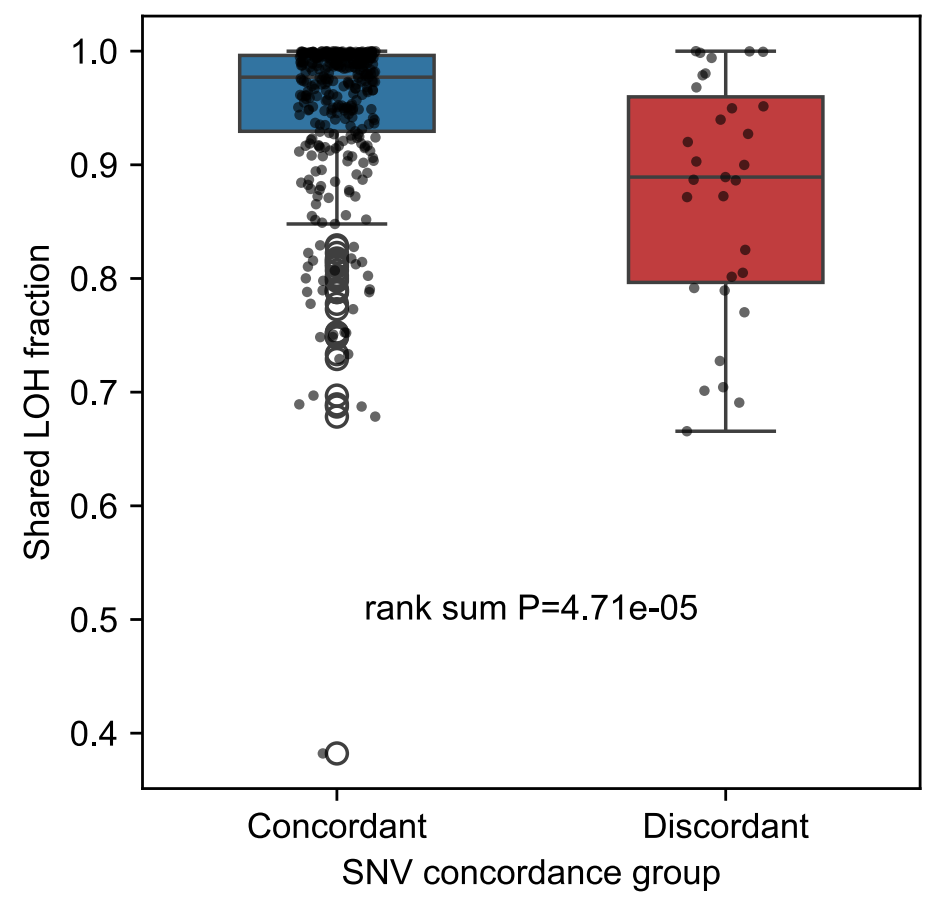

***Figure: Shared LOH fractions in concordant vs. discordant tumor–model pairs.** Shared LOH fractions were compared between tumor/model pairs with 0 SNV concordance ("discordant", red) and pairs with at least 1 SNV concordance ("concordant", blue), P value from Wilcoxon two-sided rank sum test.*

We also highlight in the revised manuscript that, among 420 models with available purity estimates, 406 (96.7 %) showed tumor purity > 80 %, and only 9 exhibited aneuploidy patterns that failed to recapitulate their parental tumors (**Extended Data Fig. 2a–c**). **Extended Data Fig. 2e,f**, and **Supplementary Note 3** highlight that the few highly discordant pairs primarily reflected either a biological subclonal outgrowth or technical challenges with sampling the same tumor for sequencing and model generation. For example, although the two pancreatic adenocarcinoma cases (PAAD) exhibited distinct KRAS G12 variants between tumors and corresponding models, that discrepancy could be attributed to the fact that pancreatic adenocarcinoma can present as a multi-focal and multi-clonal disease, and the sample used for organoid generation was physically nearby but distinct from the one used for model generation (tumor: G12R; model: G12D / G12V). This discrepancy illustrates the importance of directly sequencing a portion of the same sample as was used for model generation.

o Extended Data Fig 2f needs to be cited in the main text (p6 line 31-32)

We thank the referee for noting this oversight. **Extended Data Fig. 2f** is now correctly cited in the main text as indicated.

***9-** It would be helpful overall to include FFPE corrections where possible for FFPE-derived data. For example[,] was a[n] FFPE panel of normals used where possible [to] reduce FFPE artifacts? Correction of copy number calls in a similar way can be performed.*

We thank the referee for this important point. For SNVs and indels, standard pipelines such as Mutect2 were used, including filters designed to reduce FFPE-related artifacts (e.g., oxidative damage and deamination signatures). No additional FFPE-specific panel of normals beyond the default pipeline configuration was used.

For copy number variants, we performed copy number (CN) calling on 9 out of the 12 FFPE-derived WGS samples. However, no explicit FFPE-specific correction (e.g., Tangent normalization) was applied to the CN data. Because no panel of normals is typically used for CN calling, the consensus CN calls reflect standard practice across the contributing centers. Models were retained only if CN profiles passed internal QC thresholds.

10- *The authors should expand upon how they determine mutational signature concordance to give more clarity. Are they only assigning concordance on the basis of presence or absence of mutational signature or on the basis of fraction of mutations with each signature? Or are they generating a per-sample based signature and then assessing cosine similarity in comparing tumor/model pairs?*

We thank the referee for this helpful comment. We generated per-sample mutational signature profiles represented as 96-channel vectors (restricted to 51 non-redundant COSMIC SBS signatures) for both tumors and their matched models. Concordance was assessed by computing the cosine similarity between the two signature profiles, thereby capturing the fractional contributions of all mutational signatures rather than presence/absence alone. To make this clearer, we have revised the Methods section [Article page 36, line 33-38] as follows:

"For each tumor and matched model, we generated per-sample mutational signature profiles represented as exposure vectors across these 51 signatures. Concordance between tumors and their matched models (n = 440 pairs) was quantified in terms of the cosine similarity between their respective signature profiles. As a negative control, we generated random tumor–model pairings of equal number, both within the same tumor type and across different tumor types, and calculated cosine similarities for the randomized pairs."

11- *The link does not work: https://github.com/D-Harouni/HCMI_Explorer_Suite*

We thank the referee for flagging the link. We now consolidate all analysis and development code under a centralized repository at https://github.com/NCICCGPO/HCMI-Flagship-Manuscript, including the HCMI Explorer Suite. The repository is currently private and will be made publicly accessible upon publication of the manuscript.

We want once again to thank the referees for your kind attention to this large, complex study and the extensive responses provided here. This study certainly can't resolve all issues that arise in cancer model generation and use, but we expect that it will shed light on many of the issues and put others front and center for further inquiry. Your questions, comments, and corrections have significantly improved the manuscript in terms of both substance and communication. Thank you.

Sincerely,

Jesse Boehm, on behalf of the HCMI manuscript authors

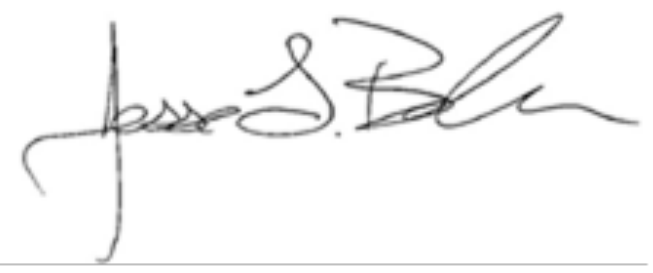

Principal Investigator
Koch Institute for Integrative Cancer Research
Massachusetts Institute of Technology
77 Massachusetts Ave, 76-453
Cambridge, MA 02139
boehm@mit.edu
Chief Science Officer
Break Through Cancer
jsb@breakthroughcancer.org
ph: 1-800-757-9881 x 704

Appendix

- GDC publication page development interface:

A Compendium of Next-Generation Patient-Derived Models for Diverse Cancer Types

GDC

Citation TBD

Development of new therapeutics and validation of pathogenetic mechanisms in cancer require representative laboratory models. However, existing model collections do not yet represent the full spectrum of diversity observed in human cancer. Recent technologies enable more efficient model derivation (e.g., tumor organoids), yet whether such models maintain the molecular states of the originating tumor during long term ex vivo expansion has not been systematically investigated. Here, we present the results of a large-scale international program—the Human Cancer Models Initiative (HCMI)—including 665 patient-derived models from tumors provided by 2,594 donors. The collection enables tumor/model comparisons by whole genome, exome, methylome and transcriptome analyses. It includes 522 models with comprehensive clinical data, 153 models of rare cancers, and 71 models derived from non-European populations. Analysis of 421 matched tumor/model pairs revealed a high degree of genetic (97.8%) and epigenetic (95%) concordance. Single-nucleus RNA sequencing of 16 tumor/model pairs revealed cell differentiation state shifts induced by specific culture medium formulations in glioblastoma. We characterize extrachromosomal DNA-based gene amplification and post-treatment mutational signatures, which provide opportunities to study mechanisms of resistance to therapy. This publicly available collection of models, with accompanying molecular and clinical characterization and integrative software tools, provides the cancer research community with a powerful resource for preclinical investigation of cancer pathogenesis and treatment response.

Supplemental Data

- **GDC Manifests**
  - HCMI-CMDC Controlled-Access Data - Download Manifest (35 Files)
  - HCMI-CMDC Open-Access Data - Download Manifest (17 Files)
- **DNA Whole Genome and Whole Exome Data**
  - nygc_snv_calls.tar.gz - NYGC snv/indel calls (Controlled)
  - broad_snv_calls.tar.gz - Broad snv/indel calls (Controlled)
  - washu_snv_calls.tar.gz - WashU snv/indel calls (Controlled)
  - consensus_snv_calls.tar.gz - Consensus snv/indel calls (Controlled)
  - ascat_cnv_calls.txt - Segmented copy number from Ascat (Controlled)
  - ReMixT_copy_number.tar.gz - ReMixT copy number data compressed tarball (Controlled)
  - cn_obs.bin50k.HCMI-remixt.h5ad.gz - ReMixT copy number anndata table (intermediate file) (Controlled)
  - cn_obs.bin50k.TCGA.h5ad.gz - TCGA copy number anndata table (intermediate file) (Controlled)
  - purple_cnv_calls.txt - Segmented copy number from Purple (Controlled)
  - cn_obs.bin50k.HCMI-consensus.h5ad.gz - Consensus copy number anndata table (intermediate file) (Controlled)
  - nygc_sv.bedpe - NYGC structural variant calls (Controlled)
  - broad_sv.bedpe - Broad Institute structural variant calls (Controlled)
  - washu_sv.bedpe - WashU structural variant calls (Controlled)
  - mskcc_sv.bedpe - MSKCC structural variant calls (Controlled)
  - embl_sv.bedpe - EMBL structural variant calls (Controlled)
  - consensus_sv.bedpe - Consensus structural variants (Controlled)
  - consensus_purity_ploidy.txt - Consensus purity/ploidy Values (Controlled)
  - loh_shared.csv - Shared LOH fraction between tumors and their paired models (Controlled)
  - wgd_status.csv - Whole Genome Doubling status for tumors and models (Controlled)
  - Supp_Table_2_Mutational Signatures.xlsx - Mutational signature (Supplementary Table 2)
  - cnv_drivers.yaml - Curated CNV driver oncogenes (Controlled)
  - snvindel_drivers.csv - SNV/indel driver genes from TCGA (https://pmc.ncbi.nlm.nih.gov/articles/PMC6029450) (Controlled)
  - Supp_Table_10_CNV Drivers.xlsx - CNV drivers (Supplementary Table 10)
  - aa-amplicon-summary-merged.rescued_ecdna.txt - ecDNA calls for both tumors and models (Controlled)
  - hcmi-merged-feature-comparisons.concordant_ecdna.txt - ecDNA calls found to be concordant between tumors and their paired models (Controlled)
- **DNA Methylation Data**
  - template_latent_reporting.xlsx - Latent transcription factor distance template (Controlled)
  - processed.tar.gz - Pre-processed DNA methylation files for TMP subtypes classification (Controlled)
  - HCMI_DNA_methylation_beta_value_matrix.tar.gz - Processed DNA methylation beta value matrix (Controlled)
  - HCMI_TCGA_TARGET_DNA_methylation_beta_value_matrix.qs - HCMI TCGA TARGET merged processed DNA methylation beta value matrix (Controlled)
- **RNA-Seq Data**
  - Supp_Table_3_Transcriptional and epigenetic fidelity.xlsx - Transcriptional and epigenetic model fidelity metrics, TMP and MoMA assignments, GBM gene expression, and GSEA results (Supplementary Table 3)
  - Supp_Table_5_TCGA-MOMA subtypes.xlsx - TCGA MOMA subtype (Supplementary Table 5)

- Supp_Table_6_HCMI-MOMA subtypes.xlsx - HCMI MOMA subtypes (Supplementary Table 6)
- Supp_Table_7_Top50MR for MOMA subtypes - Top50MR for MOMA subtypes (Supplementary Table 7)
- gdc_download_ref.tar.gz - Euclidean and Latent Transcription Factor Distance methods GDC manifests and sample sheets (Controlled)
- key_samples.csv - Key sample pairs for distance and subtype analysis used for Euclidean and Latent Transcription Factor Distance methods (Controlled)
- Supplementary_Table_Celligner_aligned_data.gz - Celligner aligned expression data (Controlled)
- Supplementary_Table_Celligner_cPC_loadings.xlsx - Celligner cPC loadings (Controlled)
- Supplementary_Table_Celligner_pairwise_euclidean_distances_in_70PC.csv - RNA-seq Celligner Distances (Controlled)
- Supp_Table_12_Celligner Model Tumor All-to-All Distances and Lineage Assignment.xlsx - Celligner Model Tumor distances and lineage assignment (Supplementary Table 12)

- **snRNA-Seq Data**
  - Supp_Table_8_snRNAseq_samples.xlsx - snRNAseq samples (Supplementary Table 8)
  - GBM-malignant-reference-signature.tsv - Gene statistics computed from a reference GBM cellular population used for data centering and scaling of GBM snRNA-seq lognormalized data
  - GBM-networks - Gene regulatory network used for protein activity-based PCA projection of GBM samples in Fig. 5
  - PAAD_networks - Gene regulatory network used for protein activity-based analysis of PAAD samples (Fig. 5, Extended Data Fig. 7)
  - PDAC-all-samples-protein-activity-ref-cells_from_model-NES.tsv - VIPER-inferred protein activity matrix for malignant cells across all PAAD snRNA-seq samples. (Note: the suffix indicates that the protein activity matrix was generated using a reference set of cells from models; however, the matrix includes samples from both tumors and models.)
  - PDAC-all-samples-ref-cells_from_model-PCA.csv - PCA coordinates for protein activity visualization of PAAD samples (Fig. 5; Extended Data Fig. 7)
  - demultiplexed_samples_best_gt_thresh-filtered-01 - Directory containing .h5ad files for HCMI snRNAseq demultiplexed samples.
  - filtered_barcodes - Directory containing .tsv files with CellRanger detected barcodes from each multiplexed sequencing run. They can be used as an input to demuxlet for single-sample demultiplexing

  - merged-filtered-01-vcf-trimmed-BAM-sorted - Directory containing multi-sample VCF files to be used as input to Demuxlet (Controlled)
  - Supp_Table_9_DE GBM snRNAseq.xlsx - GBM differential expression snRNAseq (Supplementary Table 9)
- **Metadata Files**
  - Supp_Table_1_ Comprehensive model metadata and patient characteristics.xlsx - Model and patient demographics metadata (Supplementary Table 1)
  - hcmi_cases_days_to_all_events_tx_naive_2024-03-19.xlsx - HCMI days to all events clinical metadata (Controlled)
  - HCMI_AWG_Model-Tumor-Normal_Linkage_v2.0_2.20.2024.txt - Sample pair info that was extracted and subset from HCMI_AWG_Model-Tumor-Normal_Linkage_v2.0_2.20.2024.xlsx used for Euclidean and Latent Transcription Factor Distance methods (Controlled)

Additional Resources

- GDC Data Portal

Instructions for Data Download

Open Access Data

1. Download the appropriate manifest file from the publication page
2. Use the manifest file to download data using the GDC Data Transfer Tool (DTT) or the GDC API
   - GDC DTT (Download, User's Guide)
   - GDC API (User's Guide)

Controlled Access Data

1. Download the appropriate manifest file from the publication page
2. Download a token from the GDC Data Portal
   - GDC Data Portal (Launch, User's Guide)

3. Use the manifest file and token to download data using the GDC DTT or the GDC API
   - GDC DTT (Download, User's Guide)
   - GDC API (User's Guide)

For assistance, please contact the GDC Help Desk: support@nci-gdc.datacommons.io.

KOCH INSTITUTE
for Integrative Cancer Research at MIT
Massachusetts Institute of Technology ● ki.mit.edu
77 Massachusetts Avenue ● 76-453 ● Cambridge, MA 02139 ● boehm@mit.edu

Jesse S. Boehm, PhD
Principal Investigator
Massachusetts Institute of Technology

Response to Referees

Manuscript Revised Title: A Compendium of Next-Generation Patient-Derived Models for Diverse Cancers

Manuscript number: 2025-01-02429C

We sincerely thank the referees for the thorough evaluation of our manuscript and for the positive assessment. We are grateful for the opportunity to revise the manuscript for publication.

We have carefully addressed all referee comments as well as the editorial and production requirements. Below, we summarize the key updates implemented in the revised manuscript and provide a point-by-point response to the remaining referee concerns.

Summary of key revisions

- Ensured full compliance with Nature data and code availability policies
- Restored and validated public access urls to: The HCMI Flagship Manuscript GitHub repository and The HCMI Explorer Suite
- Revised Data Availability and Code Availability statements to align with Nature standards
- Addressed all formatting, figure, and reporting requirements outlined by the editorial office

Referees' comments:

Referee #1 and #2 (Remarks to the Author):

We appreciate the authors' detailed responses and the subsequent improvements to the manuscript. The enhanced documentation significantly strengthens the work's reusability within the research community, and we are pleased with the clarifications provided regarding the cancer biology aspects of the study. That said, we have a few remaining points that require further attention:

-Code: the linked repository (https://github.com/NCICCGPO/HCMI-Flagship-Manuscript) is not available and cannot be peer reviewed.
-The mentioned HCMI explorer is inaccessible

We thank the referees for this positive assessment and for recognizing the value and reusability of the resource. We also thank the referees for highlighting the remaining accessibility issues. We have now restored access to the **HCMI Flagship Manuscript Git repository (https://github.com/NCICCGPO/HCMI-Flagship-Manuscript/)** and verified that the link is functional and accessible for review. We have also ensured that the **HCMI Explorer Suite (https://appshare.cancer.gov/HCMI_Explorer_Suite/)** is publicly accessible and updated the

relevant links in the revised manuscript. The Code Availability and Data Availability statements have been revised accordingly.

Referee #1 (Remarks on code availability):

"it is not accessible and should still be reviewed"

The code is currently open access and available for review on:
https://github.com/NCICCGPO/HCMI-Flagship-Manuscript/

Referee #3 (Remarks to the Author):

"The authors have addressed all raised concerns, and I have no further comments."
We thank the referee for the positive assessment.

Referee #4 and 5 (Remarks to the Author):

"We thank the authors for their thoughtful responses and new data and clarifications added. They have addressed all of our comments."
We thank the referee for the positive assessment.

Sincerely,

Jesse Boehm, on behalf of the HCMI manuscript authors

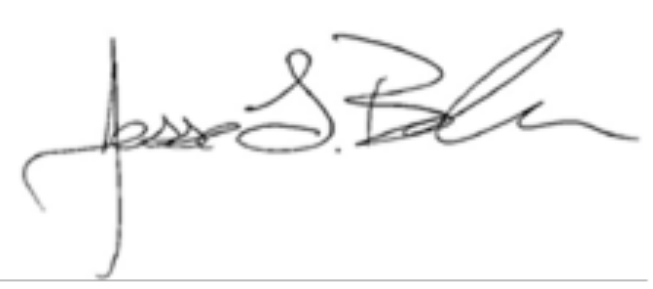

Principal Investigator
Koch Institute for Integrative Cancer Research
Massachusetts Institute of Technology
77 Massachusetts Ave, 76-453
Cambridge, MA 02139
boehm@mit.edu
Chief Science Officer
Break Through Cancer
jsb@breakthroughcancer.org
ph: 1-800-757-9881 x 704
